# A Multi-Scale Computational Study on the Mechanism of *Streptococcus pneumoniae* Nicotinamidase (SpNic)

**DOI:** 10.3390/molecules191015735

**Published:** 2014-09-29

**Authors:** Bogdan F. Ion, Erum Kazim, James W. Gauld

**Affiliations:** Department of Chemistry and Biochemistry, University of Windsor, Windsor, ON N9B 3P4, Canada

**Keywords:** nicotinamidase, thioester enzyme-intermediate, electrostatic protein environment, dispersion, QM/MM, DFT, multi-scale

## Abstract

Nicotinamidase (Nic) is a key zinc-dependent enzyme in NAD metabolism that catalyzes the hydrolysis of nicotinamide to give nicotinic acid. A multi-scale computational approach has been used to investigate the catalytic mechanism, substrate binding and roles of active site residues of Nic from *Streptococcus pneumoniae* (SpNic). In particular, density functional theory (DFT), molecular dynamics (MD) and ONIOM quantum mechanics/molecular mechanics (QM/MM) methods have been employed. The overall mechanism occurs in two stages: (i) formation of a thioester enzyme-intermediate (**IC2**) and (ii) hydrolysis of the thioester bond to give the products. The polar protein environment has a significant effect in stabilizing reaction intermediates and in particular transition states. As a result, both stages effectively occur in one step with Stage 1, formation of **IC2**, being rate limiting barrier with a cost of 53.5 kJ·mol^−1^ with respect to the reactant complex, **RC**. The effects of dispersion interactions on the overall mechanism were also considered but were generally calculated to have less significant effects with the overall mechanism being unchanged. In addition, the active site lysyl (Lys103) is concluded to likely play a role in stabilizing the thiolate of Cys136 during the reaction.

## 1. Introduction

Metal ions often play central roles in protein biochemistry such as for their folding, stabilization, and biochemical function [1,2]. For instance, approximately 40% of all known enzymes require at least one metal ion for their catalytic function [3,4,5]. In such cases the metal may, for example, be central to substrate recognition and binding, e.g., Mg^2+^ in DNA polymerase [6], or redox active within the mechanism, e.g., the oxo-manganese cluster within photosystem II [7].

Zinc is one of the most biologically important metal ions [8], with peptide hydrolases [9,10] being amongst some of the most well-known Zn(II)-containing enzymes. While the coordination of the Zn(II) may vary from 4–6 [2], these enzymes widely share a general acid/base mechanism [11,12]. More specifically, the Zn(II) facilitates formation of a suitable nucleophile via H_2_O activation. In addition, it binds to the carbonyl oxygen of the substrate’s amide bond to be cleaved. This enhances the susceptibility of the bond to nucleophilic attack at its carbon centre, and helps stabilize the tetrahedral intermediate formed during the overall reaction. A general acid subsequently protonates the amide-nitrogen completing amide bond hydrolysis [10]. 

**Scheme 1:**
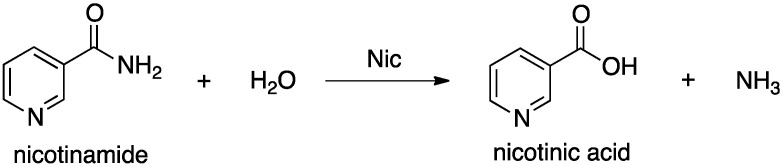
Overall reaction for conversion of nicotinamide to nicotinic acid as catalyzed by nicotinamidase (Nic).

Nicotinamidases (Nic’s) are a family of peptide hydrolases that generally contain a Zn(II) ion [13,14]. They catalyze hydrolysis of the amide bond in nicotinamide (Scheme 1) [15,16], and as such play a key role in the metabolism of the ubiquitous and important enzyme cofactor NAD^+^. While Nic’s are widespread in nature they have not been found within mammals and thus present a potential drug target [14]. Furthermore, they have recently been used in the activation of tuberculosis prodrugs such as pyrazinamide [15,17,18].

Experimentally, Du *et al.* [19] obtained X-ray crystal structures and activity measurements of a Nic from *Pyrococus horikoshii.* Based on their results and comparison with homologous enzymes they concluded that the active site contains a catalytic triad, comprised of a cysteinyl, aspartyl and lysyl, a *cis*-amide bond as part of an oxyanion hole, and a catalytic Zn(II). They proposed that the Cys acts as a nucleophile while the Zn(II) activates a coordinated H_2_O to hydrolyze the resulting enzyme-intermediate thioester bond. Fyfe *et al.* [20] have obtained X-ray crystal structures of a Nic with either product or a product-analogue bound. Importantly, they concluded that the substrate binds to the Zn(II) via its pyridine ring nitrogen (N1). That is, in the family of nicotinamidases, the Zn(II) is atypical and does not bind to the substrate’s amide bond [1,4,21].

Very recently, Sheng *et al.* [22] performed a computational QM/MM study on a nicotinamidase from a yeast species (Pnc1). In Pnc1 the Zn(II) is coordinated to two H_2_O molecules, and monodentately to two histidyl imidazoles, an aspartyl carboxylate as well as the substrate’s N1 centre in an octahedral arrangement. In contrast to that proposed by Du *et al.* [19], they concluded that the hydrolyzing H_2_O comes from the bulk solvent while the active site lysyl helps to stabilize some species along the mechanism [22]. In addition, they suggested that the Zn(II)-binding site acts as a Lewis acid rather than only the Zn(II) ion. For formation of an enzyme-substrate thioester derivative and its subsequent hydrolysis they obtained rate-limiting barriers of approximately 107.5 and 117.6 kJ·mol^−1^, respectively. 

Recently, French *et al.* [15] examined the mechanism of *Streptococcus pneumoniae* Nic (SpNic) via X-ray crystal structures in combination with inhibition and mutagenic studies. In particular, structures were obtained of the apoenzyme, and both native and a C136S mutant with substrate, product or inhibitor bound within the active site [15]. They concluded that in contrast to Pnc1, in SpNic the Zn(II) is coordinated by a single H_2_O, and monodentately via two histidyl imidazoles (His55 and His71), two R-group carboxylates (Asp53 and Glu64), as well as the substrate’s N1 centre. They further suggested that the Zn(II) helps bind and orient the substrate, as well as activating a water for hydrolysis of an intermediate’s thioester bond. Almost simultaneously, French *et al.* [23] performed experimental steady state kinetic and ^18^O isotope exchange studies and suggested that the active site lysyl or Zn(II)-bound H_2_O may have duplicate roles. In particular, either may protonate the substrate’s leaving -NH_2_ group and subsequently activate the incoming water for hydrolysis of the enzyme-intermediate thioester bond.

**Scheme 2:**
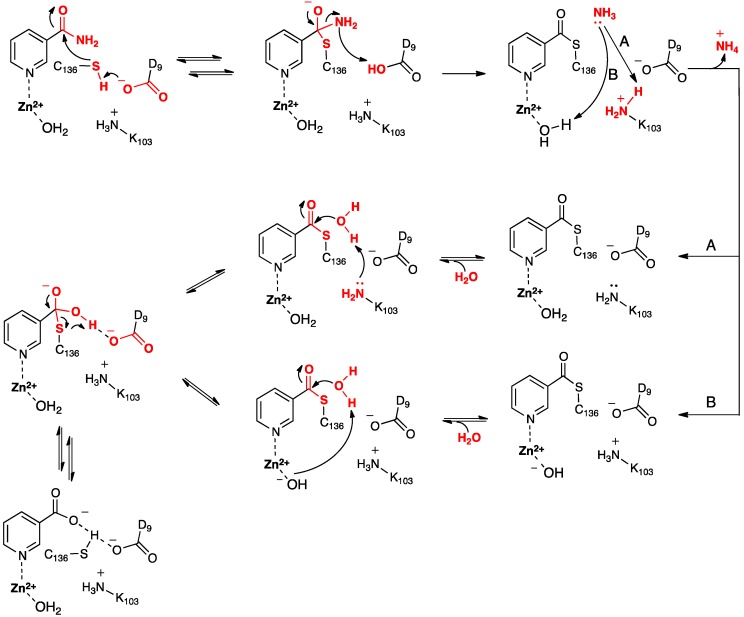
Proposed mechanism(s) for conversion of nicotinamide to nicotinic acid as catalyzed by the nicotinamidase SpNic [23].

Based in part on these findings they proposed the mechanism for SpNic shown in Scheme 2. More specifically, the thiol of the active site cysteinyl (Cys136) is deprotonated by the R-group carboxylate of a nearby aspartyl (Asp9). The now activated cysteinyl thiolate nucleophilically attacks the nicotinamide substrate’s amide carbonyl carbon, forming an enzyme-substrate tetrahedral intermediate. The Asp9-COOH proton is then transferred to the leaving amine (-NH_2_) of the substrate with concomitant collapse of the tetrahedral oxyanion intermediate and release of NH_3_. It is noted that Sheng *et al.* [22] suggested that for Pnc1 C-S bond formation occurs after proton transfer from the aspartyl to the substrate, thus also avoiding formation of a tetrahedral intermediate. Noting the potential duplicate roles of the active site lysyl (Lys103) and Zn(II)-bound H_2_O French *et al.* [23] suggested that the mechanism may proceed via pathway **A** or **B**. In **A**, Lys103 protonates the leaving ammonia and then facilitates attack of an H_2_O at the tetrahedral intermediate’s thioester bond. In **B**, the Zn(II)-bound H_2_O protonates the leaving ammonia and the resulting Zn(II)-OH then activates a bulk solvent water for attack at the intermediate’s thioester bond. It is noted that in the computational study of Sheng *et al.* [22] on Pnc1 neither pathway **A** or **B** were followed. Instead, in Pnc1 the active site lysyl helps stabilize mechanistic intermediates and transition states while Asp8 activates a water molecule for hydrolysis of the thioester bond. This results in formation of a second tetrahedral intermediate that collapses to give nicotinate and a neutral Cys167. 

In this present study, the catalytic mechanism of nicotinamidase from *Streptococcus pneumoniae* (SpNic) has been computationally investigated. More specifically, molecular dynamics (MD) and ONIOM(QM/MM)-based approaches have been complementarily applied to investigate substrate binding, as well as the catalytic mechanism and role of key active site residues. In addition, environmental and dispersion effects on the mechanism have been examined via the application of several DFT methods.

## 2. Results and Discussion 

### 2.1. The Unbound Active Site

As noted in the Introduction the catalytic activity of SpNic has been experimentally shown to depend on the triad comprising Lys103, Asp9, and Cys136 [15,23]. Thus, elucidating the initial protonation state of these residues is important to a fuller understanding of their possible roles in SpNic’s mechanism. For instance, neutralization of both the R-groups of Asp9 and Cys136 (*i.e.*, Asp9-COOH and Cys136-SH) could inhibit the mechanism and in particular formation of the putative thioester intermediate.

In aqueous solution, the proton affinity (PA) of methyl-thiolate, a model for ionized cysteine, is calculated (see Computational Methods) to be 1249.7 kJ·mol^−1^. It is noted that this value is higher than that calculated for H_2_O (1008.5 kJ·mol^−1^) at the same level of theory. However, within the SpNic active site a Cys136 thiolate has a decidedly higher PA of 1504.0 kJ·mol^−1^. This suggests that within the active site environment Cys136 is more likely to be neutral.

The role of Lys103 is somewhat ambiguous. It has been suggested by French *et al.* [15,23] to play the role of an acid in the mechanism while Fyfe *et al.* [20] have alternately proposed that it only acts as an electrostatic stabilizing factor. In aqueous solution, the R-group amine of lysine, modeled as methylamine, is calculated to have a PA of 1192.2 kJ·mol^−1^, which is higher than that calculated for H_2_O in aqueous solution. Within the unbound enzyme active site, the PA of the R-group amine of Lys103 is calculated, at the ONIOM(B3LYP/6-311+G(2df,p)//B3LYP/6-31G(d):AMBER96)-EE level of theory, to increase significantly to 1579.6 kJ·mol^−1^. This suggests that within the unbound active site the R-group of Lys103 likely exists in its protonated form (*i.e.*, Lys103-NH_3_^+^). Furthermore, it appears unlikely to be able to act as a mechanistic acid as suggested by French *et al.* [15,23] but instead may at least in part have a role as an electrostatic stabilizing factor as proposed by Fyfe *et al.* [20]. In the mechanism proposed by French *et al.* [15,23], the Zn(II)-coordinated H_2_O hydrolyzes the thioester bond of a mechanistic intermediate. However, when the Zn(II)-OH_2_ is modified to a Zn(II)-OH, the PA of Lys103-NH_2_ increases even further to 1778.2 kJ·mol^−1^. Thus, it would again appear that in such a scenario Lys103-NH_3_^+^ is unlikely to be a suitable mechanistic acid.

The possible occurrence of a stable complex in which Cys136-SH has transferred its proton onto Asp9-COO^−^ (*i.e.*, Lys103-NH_3_^+^…Asp9-COOH…Cys136-S^−^) was examined. However, no such complex was obtained at the present level of theory, suggesting that nucleophilic attack of the sulfur of Cys136-SH at the carbonyl carbon (C_carb_) of the substrate may occur with concomitant transfer of the thiol proton onto the carboxylate of Asp9.

### 2.2. The Substrate-Bound Active Site

**Figure 1:**
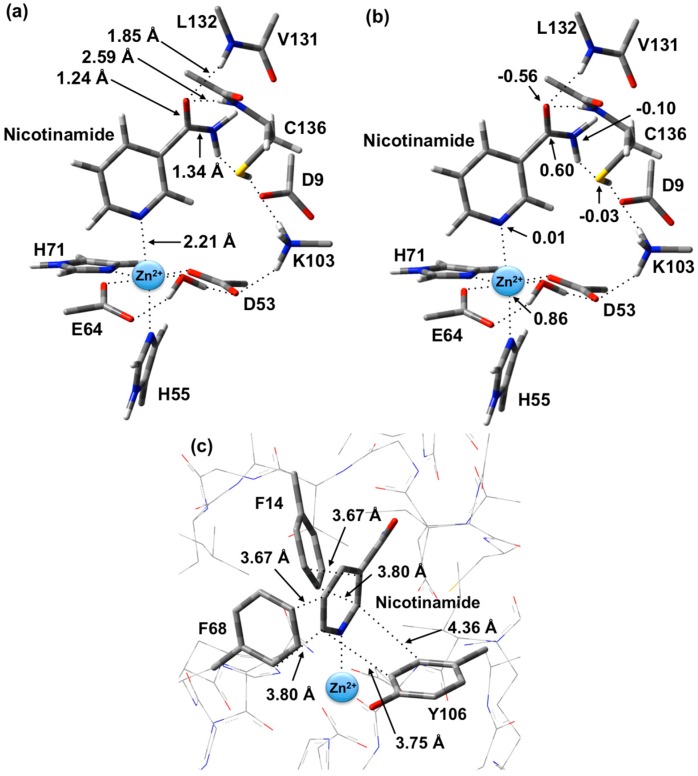
Optimized structure with (**a**) selected distances shown (Angstroms), (**b**) ESP charges, and (**c**) π-interactions in the substrate-bound active site, reactant complex (**RC**), obtained at the ONIOM(B3LYP/6-31G(d):AMBER96)-ME level of theory.

The optimized structure of the preferred substrate-bound active site, the reactant complex (**RC**), is shown in Figure 1. It is noted that the **RC** was overlaid with the X-ray crystal structure (PDB ID: 3094). This comparison shows that there were no significant differences between the two structures (e.g., Zn(II)…N1 distance is 2.27 Å in the PDB structure, whereas in the optimized structure it is 2.21 Å). In agreement with experiment [23], the Zn(II) ion adopts an essentially octahedral geometry. Specifically, it monodentately ligates to the R-group carboxylates of Asp53 and Glu64, which are coordinated *trans* to each other with similar Zn(II)…O_carb_ lengths of 2.01 and 2.03 Å, respectively. In addition, the Zn(II) ligates almost equidistantly to the R-group imidazoles of His55 (2.28 Å) and His71 (2.29 Å). It is noted that the former (His55) is *trans* to the nicotinamide substrate’s Zn(II)-coordination site. The single H_2_O bound to the Zn(II) ion has a Zn(II)…O_W_ distance of 2.12 Å, approximately 0.10 Å longer than the Zn(II)…O_carb_ distances involving Asp53 and Glu64 (see above). However, it should be noted that the water simultaneously forms very strong hydrogen bonds to the non-coordinated R-group carboxylate oxygens of both Asp53 and Glu64; *r*(O_W_H_2_…O_Asp53/Glu64_) = 1.72 and 1.24 Å, respectively. In addition, the same carboxylate oxygen of Asp53 hydrogen bonds to the protonated R-group amine of Lys103 with *r*(Lys103-NH_3_^+^…O_Asp53_) = 1.89 Å. Meanwhile, the nicotinamide substrate is bound via its pyridine N1 centre to the Zn(II) at a distance of 2.21 Å, which is slightly shorter than the Zn(II)…His55/71 coordination bonds.

As can also be seen in Figure 1 the R-groups of the catalytic triad of Lys103, Asp9 and Cys136 form a strong hydrogen bond chain with *r*(Lys103-NH_3_^+^…O_Asp9_) and *r*(O_Asp9_…HS_Cys136_) lengths of 1.59 and 1.86 Å, respectively. The backbone -NH- moieties of Leu132 (1.85 Å) and Cys136 (2.59 Å), which may play a role in stabilizing an oxyanion intermediate, form moderate and quite weak hydrogen bonds respectively with the substrate’s carbonyl oxygen.

The structure of the active site-bound nicotinamide substrate was also compared to that obtained for the substrate in aqueous solution in order to provide insights into the effects of binding. Notably, upon binding the substrate’s amide group C=O bond lengthens slightly by 0.01 Å to 1.24 Å while the C-N bond shortens by 0.02 Å to 1.34 Å. Furthermore, the substrate’s dihedral angle ∠C2-C3-C(O)-N increases on binding by 15.1° to 35.6°.

There are several other interactions between substrate and active site residues that play a role in substrate binding and orientation. In particular, based on observed distances in X-ray crystal structures [15], Phe14, Phe68, and Tyr106 are thought to be involved in π-interactions with the nicotinamide ring. As shown in Figure 1c, these interactions were observed in the optimized structure obtained for **RC**. In particular, the π-π interactions are positioned in an edge-to-face type of arrangement. Both Phe14 and Phe68 aromatic rings are stacked 3.67–3.80 Å away from the nicotinamide substrate’s pyridyl. Meanwhile, the Tyr106 aromatic ring is 3.75–4.36 Å away.

It is also observed that upon binding within the active site, the positive charge on the substrate’s C_carb_ centre decreases slightly by 0.09 from that calculated for the isolated substrate in aqueous solution to 0.60 while the negative charge on O_carb_ is essentially unchanged at −0.56. This would appear to suggest that at least in **RC** formation, the role of the Zn(II) is primarily to facilitate proper binding orientation of the substrate.

### 2.3. Catalytic Mechanism of SpNic

As described in the Introduction it has been proposed [23] that the mechanism occurs in two stages: (i) formation of a thioester covalently cross-linked enzyme-substrate complex with loss of the substrate’s amine group, and (ii) hydrolysis of the enzyme-substrate’s thioester bond and product formation.

#### 2.3.1. Stage 1: Formation of a Thioester Enzyme-Intermediate Complex with Loss of Ammonia

**Figure 2:**
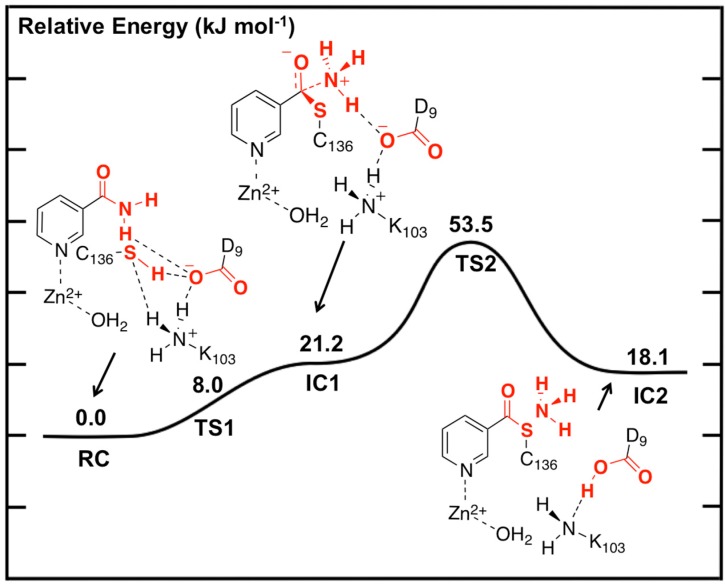
Potential energy surface (kJ·mol^−1^) obtained at the ONIOM(B3LYP/6-311+G(2df,p):AMBER96)-EE//ONIOM(B3LYP/6-31G(d):AMBER96)-ME level of theory for formation of the thioester enzyme-intermediate with concomitant loss of ammonia.

**Figure 3:**
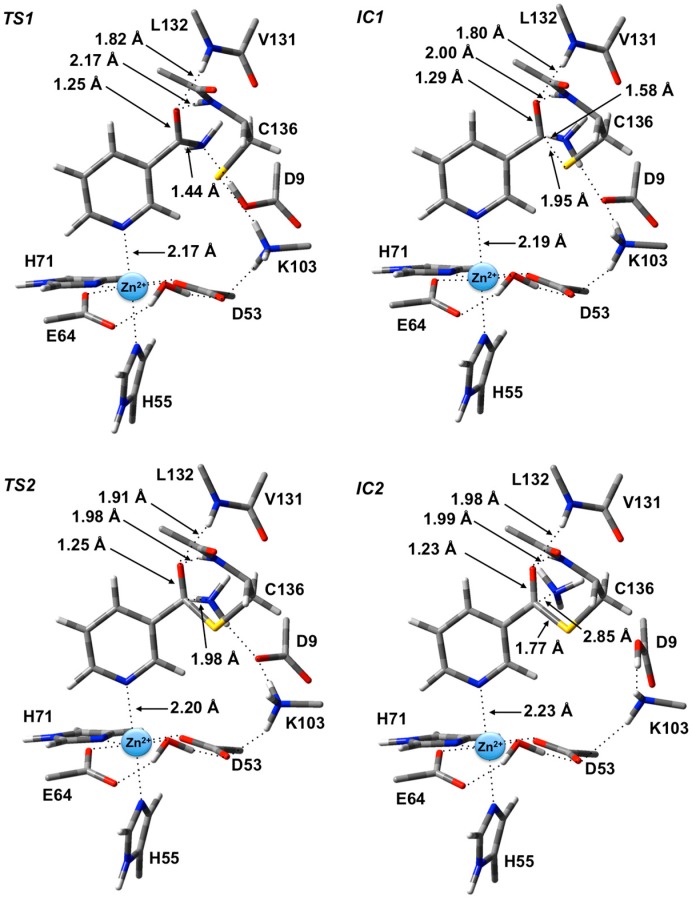
Optimized geometries obtained at the ONIOM(B3LYP/6-31G(d):AMBER96)-ME level of theory of the transition states (**TS1** and **TS2**) and intermediates (**IC1** and **IC2**) for the first stage of the overall mechanism of SpNic.

The potential energy surface obtained, at the ONIOM(B3LYP/6-311+G(2df,p):AMBER96)-EE//ONIOM(B3LYP/6-31G(d):AMBER96)-ME level of theory, for Stage 1 of the overall mechanism of SpNic is shown in Figure 2. The optimized structures of the corresponding intermediates and transition states are shown in Figure 3.

The first step is formation of a tetrahedral thioester enzyme-substrate intermediate. More specifically, the sulfur of Cys136 nucleophilically attacks the nicotinamidase substrate’s carbonyl carbon (C_carb_) while concomitantly the Cys136-SH thiol proton is transferred onto the substrate’s amide group nitrogen. The proton transfer is facilitated directly by the carboxylate of Asp9. Indeed, in **TS1** Asp9 more closely resembles a neutral aspartic acid (Asp9-COOH) indicating that transfer of the proton onto the substrate amide nitrogen occurs late in this step. The resulting tetrahedral intermediate **IC1** lies only 21.2 kJ·mol^−1^ higher in energy than **RC** and is able to reversibly rearrange back to the reactive complex essentially without a barrier. The lower relative energy of **TS1** with respect to **IC1** is a common artifact of the use of single-point calculations and/or empirical corrections on flat PES’s and typically indicates that at the higher level of theory used to obtain relative energies, the reaction likely occurs without a barrier. Interestingly, the environment appears to play a significant role in this step by electrostatically stabilizing both **TS1** and **IC1**. Single point calculations at the same level of theory but without inclusion of the environment’s electrostatic charge (*i.e.*, ONIOM(B3LYP/6-311+G(2df,p):AMBER96)-ME//ONIOM(B3LYP/6-31G(d):AMBER96)-ME level of theory) give a barrier for this step of 68.5 kJ·mol^−1^ while **IC1** lies 49.2 kJ·mol^−1^ higher in energy than **RC**.

**Figure 4:**
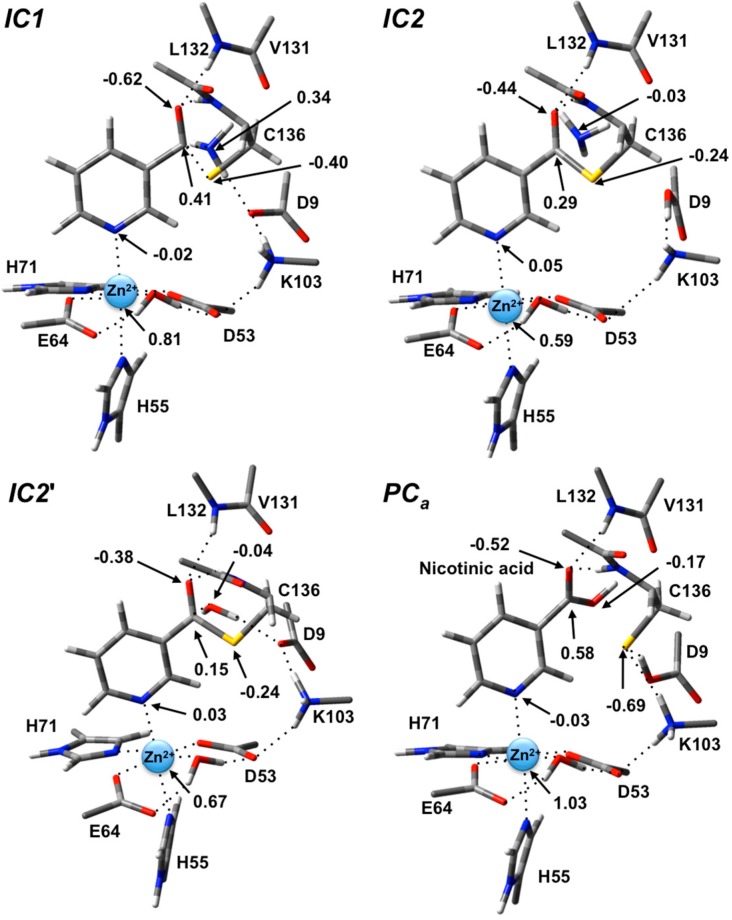
ESP charges for select key species in the SpNic mechanism (H-atom inclusive).

In **IC1** there now exists a weak C_carb_-S_Cys136_ bond as indicated by its length of 1.95 Å while the substrate’s -NH_2_ group is now protonated (Figure 3). Concomitantly, the C_carb_=O bond has lengthened from that observed in **RC** by 0.05 Å to 1.29 Å, while the C_carb_-N bond has lengthened significantly by 0.24 Å to 1.58 Å (Figure 3). There is a slight increase in oxyanionic character of the substrate’s O_carb_ centre to −0.62 (Figure 4). This in part causes both the Leu132/Cys136-NH…O_carb_ hydrogen bonds to shorten significantly to 1.80 and 2.00 Å, respectively (Figure 3). It is noted that the Zn(II) charge (Figure 4) is calculated to have decreased slightly in **IC1** to 0.81 while Zn(II)…N1 distance has shortened slightly by 0.02 Å to 2.19 Å.

The second and final step of Stage 1 is collapse of the tetrahedral intermediate resulting in cleavage of the C_carb_**^…^**NH_3_ bond. For this step we performed detailed scans of the PES. Importantly, an energy maximum (**TS2**) of 53.5 kJ·mol^−1^ with respect to **RC** (Figure 2) was obtained upon elongating the C_carb_**^…^**NH_3_ bond to 1.98 Å at which distance it is effectively cleaved. This is in fact the rate-limiting step of the overall mechanism. The resulting enzyme-intermediate thioester complex **IC2** lies higher in energy than **RC** by 18.1 kJ·mol^−1^ (Figure 2). Within **IC2** the C_carb_**^…^**NH_3_ distance has lengthened even further to 2.85 Å while the C_carb_-S_Cys136_ bond has shortened significantly from that in **IC1** (1.95 Å) to 1.77 Å and now resembles a typical C-S single bond (Figure 3). Furthermore, the C_carb_=O_carb_ bond has also shortened by 0.06 Å to 1.23 Å. In the calculated ESP charges for **IC2** (Figure 4) the positive charge on C_carb_ has decreased to 0.29 while that of O_carb_ is now less negative (*i.e.*, less oxyanion character) at −0.44.

With the ammonia moiety now effectively free in the active site, several possible scenarios exist where water may be made available for the subsequent hydrolysis stage. It has been suggested that the Zn(II)-bound H_2_O may be the required water [15]. However, given its position relative to C_carb_ (*r*(C_carb_…O_water-Zn_) = 5.10 Å) and that it is hydrogen bonded to the nearby carboxylates of Asp53 and Glu64, this would seem unlikely.

Alternately, the cleaved NH_3_ may leave the active site and be replaced by a solvent H_2_O. It has been previously proposed by French *et al.* [15,23] that the cleaved NH_3_ may be protonated by the R-group amine of Lys103. However, based on the optimized structure of **IC2** this would appear unlikely to occur at least directly due to sterics and without some rearrangement of the active site’s hydrogen bonding network. In particular, Lys103 and the leaving NH_3_ are separated by 5.96 Å and with residues and the thioester intermediate between them, make an unlikely proton transfer. Furthermore, the Lys103-NH_2_ group remains hydrogen bonded to both the carboxylate R-groups of Asp9 and Asp53 (Figure 3). Alternatively, however, the NH_3_ may simply be eliminated from the active site and replaced by a H_2_O. This latter option has been used to provide a suitable reactive complex for the second stage of the mechanism.

#### 2.3.2. Stage 2: Hydrolysis of the Thioester Enzyme-Intermediate and Product Formation

**Figure 5:**
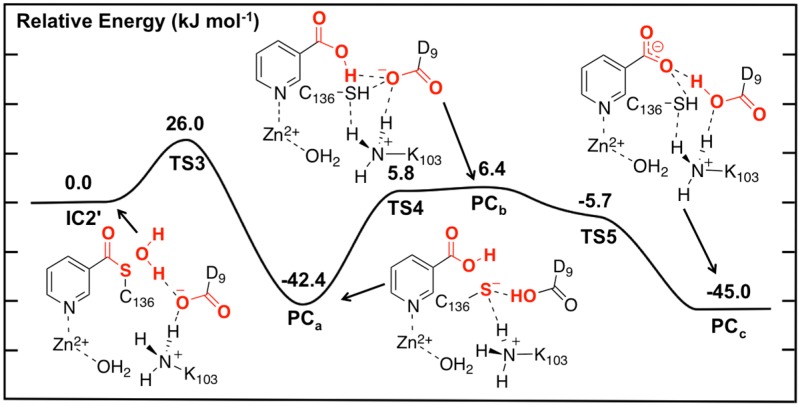
Potential energy surface (kJ·mol^−1^) obtained at the ONIOM(B3LYP/6-311+G(2df,p):AMBER96)-EE//ONIOM(B3LYP/6-31G(d):AMBER96)-ME level of theory for the hydrolysis of the thioester intermediate and product complex formation.

**Figure 6:**
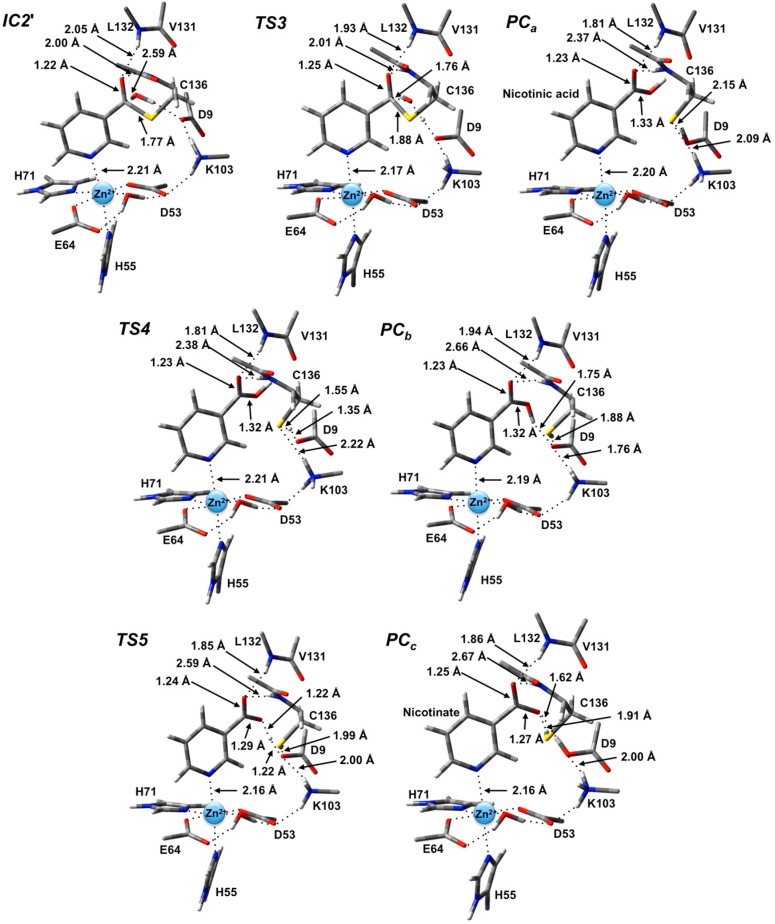
Optimized geometries obtained at the ONIOM(B3LYP/6-31G(d):AMBER96)-ME level of theory of the thioester intermediate + H_2_O (**IC2'**) and other transition states (**TS_x_**, **x** = **3**, **4**, **5**) and product complexes (**PC_x_**, **x** = **a**, **b**, **c**) for the second stage of the overall mechanism of SpNic.

Unfortunately, no present experimental X-ray crystal structures have been obtained with a water bound in an appropriate position for replacing the leaving NH_3_. Hence, the NH_3_ moiety of **IC2** was appropriately substituted by a H_2_O molecule. The resulting potential energy surface obtained for hydrolysis of the thioester intermediate and product formation is given in Figure 5 while the optimized structures of the corresponding intermediates, transition states and product complexes are given in Figure 6.

In the optimized structure of the thioester intermediate **IC2'**, the H_2_O sits such that its oxygen O_w_ is 2.59 Å from C_carb_ with one of its lone pairs directed towards C_carb_ (Figure 6). The most significant changes observed on introducing the water are due to the strong hydrogen bond (1.87 Å) it forms with the R-group carboxylate of Asp9. This induces a change in the hydrogen bond network of the catalytic triad. Specifically, the R-group carboxylate of Asp9 transfers a proton back to the R-group amine of Lys103, which in turn affects the latter hydrogen bond interaction with the Zn(II)-coordinated Asp53. This induces a slight increase of 0.08 in the charge on the Zn(II) centre (Figure 4).

The first step in the second stage is Asp9-facilitated nucleophilic attack of the water’s oxygen (O_w_) at the thioester’s C_carb_ centre with concomitant cleavage of the C_carb_-S_Cys136_ bond and transfer of a water proton onto the R-group carboxylate of Asp9. This exothermic reaction step proceeds with a quite low barrier of just 26.0 kJ·mol^−1^ via **TS3** (Figure 5). The product complex formed, **PC_a_**, lies lower in energy than **IC2'** by 42.4 kJ·mol^−1^. In **PC_a_**, neutral nicotinic acid has been formed and is bound within the active site. It can also be seen that Lys103 plays a stabilizing role by forming a relatively strong hydrogen bond (2.09 Å) with the thiolate of Cys136 (Figure 6). In the computational study of Sheng *et al.* [22] on Pnc1 it was similarly concluded that in that case the active site lysyl (Lys122) aids in stabilizing stationary points along the mechanism, although for SpNic it appears more directly involved in stabilizing active site residues in such species. The formation of **PC_a_** in essence marks successful completion of the catalytic mechanism of SpNic. The nicotinic acid can now be released from the active site while the catalytic triad residues are likely to easily return to their initial starting states.

However, an alternate product complex (**PC_c_**) lies marginally lower in energy than **PC_a_** by 2.6 kJ·mol^−1^ (Figure 5) at the present level of theory (See Computational Methods). Like **PC_a_** the R-group of Asp9 is non-ionized (*i.e.*, Asp9-COOH) while that of Lys103 is protonated (*i.e.*, Lys103-NH_3_^+^) as it can be seen in Figure 6. The differences are that in **PC_c_** the thiol of Cys136 is now neutral (*i.e*., Cys136-SH) while the product is now nicotinate. This rearrangement of the hydrogen bond network can occur in two steps via the intermediate product complex **PC_b_** at a cost of 48.8 kJ·mol^−1^ with respect to **PC_a_**. In **PC_b_**, Cys136 is neutral while Asp9 is ionized, and the there has been a rotation about the C_carb_-OH bond in the nicotinic acid product. Both transition structures **TS4** and **TS5** are calculated to lie lower in energy than **PC_b_** at the level of theory used to obtain the PES in Figure 5, indicating that **PC_b_** likely rearranges without a barrier to either **PC_a_** or **PC_c_**.

### 2.4. Inclusion of Dispersion Effects on the Catalytic Mechanism of SpNic

**Figure 7:**
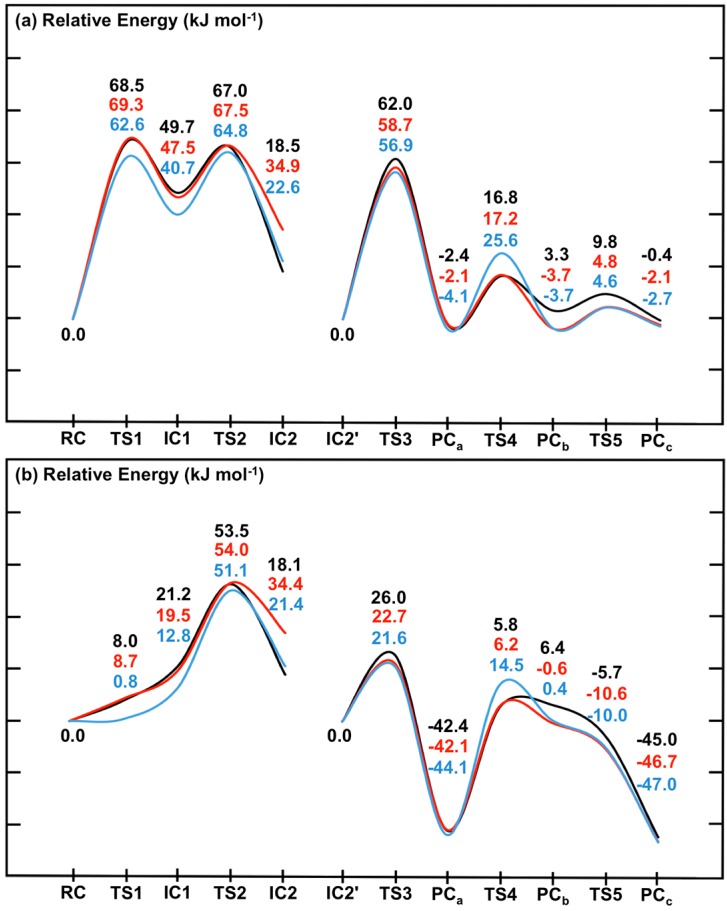
Potential energy surfaces for the overall catalytic mechanism of SpNic obtained at the ONIOM(**DFT_i_**/6-311+G(2df,p):AMBER96)//ONIOM(B3LYP/6-31G(d):AMBER96) level of theory within the (**a**) ME and (**b**) EE formalism. **DFT_i_** = B3LYP (black), B3LYP-D3 (red) or M06 (blue).

Similar to the computational work of Sheng *et al.* [22] on the Pnc1 nicotinamidase, we have used the B3LYP functional to describe the high-layer or reactive region of the QM/MM model. This functional has been and currently continues to be widely used in such studies. However, it is unable to describe dispersion interactions which may be important in enzymatic reactions [24]. Indeed, as noted above for **RC**, in the case of SpNic several non-polar or hydrophobic groups are present in and around the active site and substrate. Fortunately, there are now corrections that can be applied such as those of Grimme [25] and newer functionals such as M06 that better account for such effects. The above PES’s obtained for the mechanism of SpNic were recalculated using such corrections and functionals within both an ME (Figure 7a) and EE (Figure 7b) formalism (*i.e.*, without and with inclusion of the effects of the environment’s polarity, respectively).

First it should be noted that comparison of the corresponding coloured lines in Figure 7a *versus* Figure 7b indicates the effects of including the effects of the electrostatic environment on the entire mechanism of SpNic. The same trends are observed regardless of the functional used. Namely, the electrostatic environment lowers all reaction barriers, *i.e.*, preferentially stabilizes **TS**’s, some even to the extent that they (*i.e.*, **TS1** and **TS5**) essentially do not exist (see above). As a result, Stage 1 of the mechanism becomes a one-step reaction. The smallest decreases of 11.0–11.1 kJ·mol^−1^ are observed for **TS4**, while the largest changes are 60.5–61.8 kJ·mol^−1^ for **TS1**. While the polar environment also lowers the relative energies of almost all intermediate and product complexes, the observed decreases are significantly less than observed for the transition states (**TS**’s). For instance, the smallest decreases are observed for **IC2** and are now just 0.4–1.2 kJ·mol^−1^, while the largest decreases are 44.3–44.6 kJ·mol^−1^ and occur for **PC_c_**. The only exception is **PC_b_** whose energy increases by 3.1–4.1 kJ·mol^−1^. It is noted that Sheng *et al.* [22] concluded that in Pnc1 the Zn(II) binding site acts as a Lewis acid to influence the reaction.

The inclusion of dispersion interaction effects via use of Grimme’s correction (*i.e.*, **DFT_i_** = B3LYP-D3) generally has negligible or only minor effects on the calculated PES, within either the ME or EE formalism (*i.e.*, without or with inclusion of the polar environment effects, respectively). Furthermore, its effects can be to either increase (e.g., **IC2**) or decrease (e.g., **PC_b_**) the relative energy. In particular, the largest increases are 16.3–16.4 kJ·mol^−1^ and are observed for **IC2**. Meanwhile, the most significant decreases at just −6.4–−7.0 kJ·mol^−1^ are obtained for **PC_b_**. Thus, compared to the impact of including the environment’s electrostatic effects (*i.e.*, ME → EE), the inclusion of dispersion effects via Grimme’s corrections (*i.e.*, **DFT_i_** = B3LYP → B3LYP-D3) generally has less significant effects. Importantly, however, the overall mechanism and identity of the rate-limiting barrier (**TS2**) remains unchanged.

For the case in which **DFT_i_** = M06 for the high-layer in the single point calculation (see above) slightly different behaviours are observed between when used within an ME or EE formalism (*i.e.*, without or with inclusion of the environment’s electrostatic effects, respectively). Within the former (Figure 7a), the M06 functional gives the same general overall mechanism for SpNic as obtained for **DFT_i_** = B3LYP. However, now **TS1**, **IC1** and **TS2** have lower energies relative to **RC** by 5.9, 8.5 and 2.2 kJ·mol^−1^, respectively. More significant changes are observed for Stage 2. In particular, while **DFT_i_** = M06 generally gives relative energies in agreement with those obtained using B3LYP-D3, it gives a markedly higher barrier for **TS4** of 14.5 kJ·mol^−1^ (*c.f.*, Figure 5). Indeed, for **DFT_i_** = M06 this reaction step essentially has a higher barrier than for C_carb_-N cleavage via **TS2** though by only 7.5 kJ·mol^−1^, and is thus now the rate-limiting step. In addition, it also predicts notably lower relative energies for **PC_b_** and **TC5**, which are now in fact in good agreement with those obtained using **DFT_i_** = B3LYP-D3 (Figure 7a). When the polar environment is included via use of the EE formalism, some notable changes in the PES upon changing **DFT_i_** to M06 are observed. In particular, as for **DFT_i_** = B3LYP both **TS1** and **IC1** are greatly stabilized by the polar environment but now they are almost thermoneutral with **RC** having relative energies of 0.8 and 12.8 kJ·mol^−1^, respectively (*c.f*., Figure 2). For the second stage, the largest changes are observed for **TS4** which now has a notably higher energy relative to **IC2'** by 8.7 kJ·mol^−1^, while both **PC_b_** and **TS5** are stabilized by 4.3 and 2.0 kJ·mol^−1^, respectively.

## 3. Computational Methods

### 3.1. Molecular Dynamics (MD) Simulations

MD simulations were performed using the Molecular Operating Environment (MOE) [26] program. The X-ray crystal structure of the C136S mutant of homo-tetrameric SpNic complexed with nicotinamide was used as the template structure (PDB ID: 3O94) [15]. As the active site does not contain any monomer interface residues, an appropriate single monomer was selected for further calculations, Ser136 mutated to cysteine, while missing hydrogen atoms were added using the MOE default method. The resulting enzyme-substrate complex was solvated using a 7-Å spherical layer of water molecules. An ellipsoidal potential wall with a scaling constant of 2 was placed around the solvated enzyme-substrate complex, thus ensuring the system lies within the volume of space established by the ellipsoid. A damping functional factor was included to allow the electrostatic and van der Waals potentials to decay smoothly. The solvated complex was optimized using the AMBER99 force field until the root mean square gradient of the total energy was below 0.21 kJ·mol^−1^·Å^−1^. The optimized complex underwent thermal relaxation at constant pressure and temperature. The Nosé-Poincaré thermostat [27] was coupled with the equations of motion and a 2 fs time step was set for numerical integration. The system was annealed by heating it from 150 to 300 K over a 25 ps period, then held at 300 K for a further 25 ps. Then it was heated to 400 K over a 25 ps period, then held at 400 K for a further 325 ps before being cooled down to 300 K over 50 ps. This annealing process was stopped after a further 50 ps interval held at 300 K. The final structure from the trajectory was then optimized using the AMBER99 force field. We have successfully used this MD protocol in studies on other enzymes [28,29].

### 3.2. ONIOM(QM/MM) Calculations

The QM/MM calculations were done using the ONIOM [30,31,32,33,34,35,36,37,38] formalism in the Gaussian 09 [39] suite of programs. Optimized geometries and harmonic vibrational frequencies, to characterize the nature of the stationary points, were obtained at the ONIOM(B3LYP/6-31G(d):AMBER96) level of theory within the mechanical embedding (ME) formalism. That is, the reactive region (high-layer) was described at the B3LYP/6-31G(d) [40,41,42] level of theory while the surrounding protein environment (low-layer) was described using the AMBER96 [43] force field. Relative energies were obtained via single point calculations at the ONIOM(B3LYP/6-311+G(2df,p):AMBER96) level of theory, on the above optimized structures, within an electronic embedding (EE) formalism. The effects of high-layer method choice and dispersion were modeled via single point calculations, on the above optimized structures, at the ONIOM(**DFT_i_**/6-311+G(2df,p):AMBER96) level of theory within both an ME and EE formalism, where **DFT_i_** = B3LYP-D3 or M06. B3LYP-D3 indicates that Grimme’s D3 dispersion [25] scheme as implemented in Gaussian 09 was applied to the B3LYP functional.

**Figure 8:**
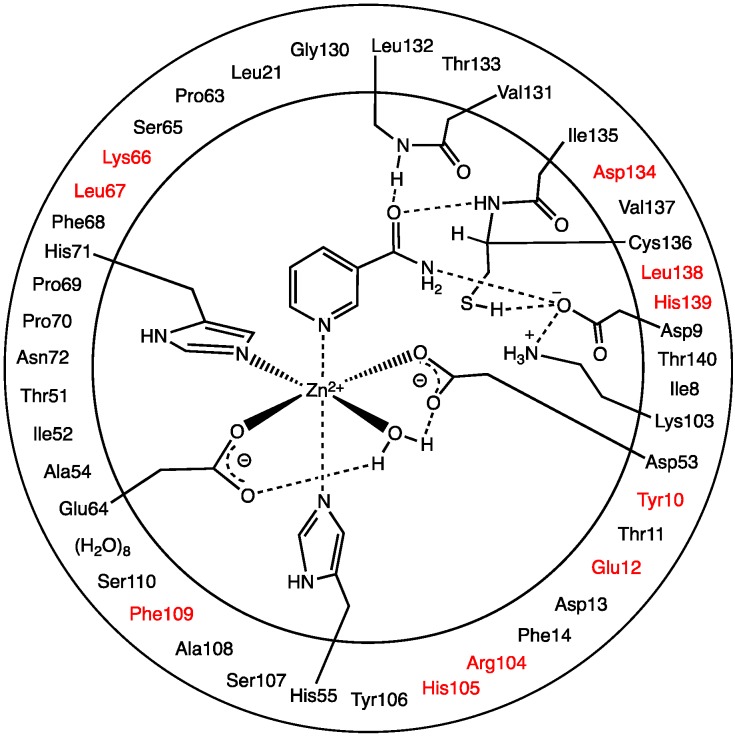
Schematic representation of the substrate-bound active site model used in the QM/MM calculations. The outer circle represents the MM-layer, while those in the inner circle represent the QM-layer. Residues in red were mutated to glycyl (see text).

The QM/MM chemical model was derived from the final optimized MD structure (see above) and is illustrated in Figure 8. As the steric and electrostatic effects of the environment surrounding the active site can have important affects on the mechanism [44], all residues and waters up to 15 Å away from the Zn(II) were included. The QM-region contained the Zn(II) ion, the R-groups of its first coordination sphere residues (His71, Glu64, Asp53 and His55), and its coordinated H_2_O. In addition, the nicotinamide substrate, R-groups of the catalytic triad residues (Lys103, Asp9, and Cys136), and peptide backbones between Val131-Leu132 (α-C_131_-CO-NH-α-C_132_) and Ile135-Cys136 (α-C_135_-CO-NH-α-C_136_) were included. The outer circle in Figure 8 indicates the residues and waters included in the MM-layer in their entirety except those partially in the high-layer, while those in red were mutated to glycyl, *i.e.*, only their backbones were included. The latter was done due to their location at the periphery of the system and to reduce computational cost. The α-carbon of each residue in the MM-layer was held fixed at its final AMBER99 optimized position (see above). This computational approach has been successfully applied in studies on related enzymatic systems [28].

For proton affinity (PA) calculations on isolated species in aqueous solution, optimized geometries were obtained at the B3LYP/6-31G(d) level of theory. Relative energies were obtained via single point calculations on the above structures at the IEFPCM-B3LYP/6-311+G(2df,p) level of theory. IEFPCM indicates polar environment effects were included by use of the Integral Equation Formalism-PCM method with a dielectric constant (ε) of 78.3 [45,46,47,48]. Active site PA’s were calculated at ONIOM(B3LYP/6-311+G(2df,p):AMBER96)-EE level of theory on the above optimized structures.

## 4. Conclusions

Several computational approaches including DFT-small chemical models, molecular dynamics (MD) and ONIOM quantum mechanics/molecular mechanics (QM/MM), have been complementarily applied to the study of a nicotinamidase from *Streptococcus pneumoniae*, referred to as SpNic. Specifically, its catalytic mechanism as well as substrate binding and the role of key active site residues has been investigated.

Initial studies examined the proton affinities of the catalytic triad residues Cys136 and Lys103. It has been suggested that Lys103 may help facilitate proton transfer from the Zn(II)-bound water to give a Zn(II)-bound hydroxyl (*i.e.*, Zn(II)…OH_2_ → Zn(II)…OH^−^). However, the proton affinity of the amine R-group of Lys103 increases in the presence of Zn(II)…OH^−^ suggesting that it is unlikely to be able to participate in such a proton transfer. Rather, Lys103 may play a stabilizing role in the mechanism, in particular for the thiol/thiolate of Cys136.

Using ONIOM(QM/MM) approach within both a mechanical embedding (ME) and electronic embedding (EE) formalism, *i.e.*, without and with inclusion of the environment’s electrostatic effects, the overall two-stage catalytic mechanism of SpNic was elucidated.

It is shown that the electrostatic environment has a significant impact on the overall mechanism. In particular, within an ME formalism, Stage 1, formation of an enzyme-substrate thioester intermediate with loss of NH_3_, occurs via a two-step mechanism. At the ONIOM(B3LYP/6-311+G(2df,p):AMBER96)//ONIOM(B3LYP/6-31G(d):AMBER96) level of theory the first step, formation of a tetrahedral enzyme-substrate intermediate (**IC1**) is rate-determining with a barrier of 68.5 kJ·mol^−1^. However, inclusion of the effects of the polar environment results in stabilization of all transition states and, to a lesser extent, intermediates, along the mechanism with only a few exceptions. Indeed, using the same level of theory but within an EE formalism, Stage 1 becomes a one-step reaction: nucleophilic attack of the sulfur of Cys136 at the substrate’s C_carb_ centre occurs with concomitant cleavage of the C_carb_-NH_2_ bond to give the enzyme-substrate thioester intermediate (**IC2**) with loss of NH_3_. Furthermore, the calculated barrier is now only 53.5 kJ·mol^−1^. In contrast, Stage 2 of the overall mechanism, hydrolysis of the C_carb_-S bond in **IC2'** with formation of the nicotinic acid product essentially occurs in one-step within both the ME and EE formalism with barriers of 62.0 and 26.0 kJ·mol^−1^_,_ respectively.

Dispersion interaction effects were modeled via application of Grimme’s dispersion corrections dispersion [42] to the B3LYP method, *i.e.*, B3LYP-D3. The largest effects were observed for **IC2** which was destabilized by 16.4 kJ·mol^−1^ and the alternate product complex **PC_b_** and **TS5** which were stabilized by 7.0 and 5.0 kJ·mol^−1^, respectively. Notably, their effects are much less in general than those due to polarity of the environment.

Use of the M06 functional, *i.e.*, ONIOM(M06/6-311+G(2df,p):AMBER96)//ONIOM(B3LYP/6-31G(d):AMBER96), within both the ME and EE formalism gave results in reasonable agreement within those obtained using B3LYP and B3LYP-D3. Importantly, the same overall catalytic mechanism for SpNic was obtained.

## Supplementary Materials

Supplementary materials can be accessed at: http://www.mdpi.com/1420-3049/19/10/15735/s1.

## Supplementary Files

Supplementary File 1
