# Supplementary material for: A Multi-Scale Computational Study on the Mechanism of Streptococcus pneumoniae Nicotinamidase (SpNic)
Source: Molecules. 2014 Sep 29;19(10):15735–53. doi: 10.3390/molecules191015735 (PMC6271260; doi:10.3390/molecules191015735)
Supplement: Supplementary File 1 [file molecules-19-15735-s001.pdf]

A Multi-Scale Computational Study on the Mechanism of *Streptococcus pneumoniae* Nicotinamidase (SpNic)

Bogdan F. Ion, Erum Kazim, James W. Gauld

Department of Chemistry and Biochemistry, University of Windsor, Windsor, Ontario, N9B 3P4, Canada

Supporting Information

(Table S1; total pages: 150)

Table S1. Cartesian coordinates and partial charges from the QM/MM optimized structures of each stationary point along the SpNic pathway.

RC

C-CT--0.3662	-1	-10.318774	3.893278	4.028288	L		
C-C-0.5972	0	-8.979322	3.258284	3.784726	L		
O-O--0.5679	0	-8.401332	2.684489	4.700777	L		
H-HC-0.1123	0	-11.055043	3.426341	3.376974	L		
H-HC-0.1123	0	-10.252599	4.957075	3.807034	L		
H-HC-0.1123	0	-10.599066	3.747038	5.069157	L		
N-N--0.415700	0	-8.509410	3.361766	2.548020	L		
C-CT--0.059700	-1	-7.349484	2.632131	2.027858	L		
C-C-0.597300	0	-6.184882	3.589892	1.820878	L		
O-O--0.567900	0	-6.297558	4.543025	1.055571	L		
C-CT-0.130300	0	-7.724518	1.892598	0.718187	L		
C-CT--0.043000	0	-8.891163	0.899032	0.948690	L		
C-CT--0.320400	0	-6.504857	1.143942	0.150558	L		
C-CT--0.066000	0	-9.464938	0.296331	-0.339229	L		
H-H-0.271900	0	-9.053618	3.919328	1.897757	L		
H-H1-0.086900	0	-7.042452	1.879500	2.754052	L		
H-HC-0.018700	0	-8.042920	2.634358	-0.016762	L		
H-HC-0.023600	0	-9.715670	1.410081	1.444322	L		
H-HC-0.023600	0	-8.558752	0.090537	1.600977	L		
H-HC-0.088200	0	-6.151789	0.395545	0.860966	L		
H-HC-0.088200	0	-5.694294	1.841156	-0.065267	L		
H-HC-0.088200	0	-6.761097	0.660221	-0.789845	L		
H-HC-0.018600	0	-9.722821	1.090070	-1.039743	L		
H-HC-0.018600	0	-10.367183	-0.265865	-0.100976	L		
H-HC-0.018600	0	-8.744467	-0.381187	-0.794587	L		
N-N--0.516300	0	-5.086634	3.329853	2.524596	L		
C-CT-0.038100	-1	-3.784538	3.968859	2.394541	L	H-HC	30
C-C-0.536600	0	-3.733867	5.488885	2.130629	L		
O-O--0.581900	0	-2.778894	6.017864	1.561711	L		
C-CT--0.547145	0	-2.996924	3.171448	1.336178	H		
C-C-0.532653	0	-2.496457	1.812920	1.794361	H		
O-O2--0.588507	0	-2.676992	1.426678	2.957374	H		
O-O2--0.625559	0	-1.846973	1.121297	0.903344	H		
H-H-0.293600	0	-5.082886	2.482910	3.079897	L		
H-H1-0.088000	0	-3.263305	3.839911	3.343878	L		
H-HC-0.128435	0	-2.102566	3.745851	1.049319	H		
H-HC-0.183556	0	-3.588548	3.095993	0.418483	H		
N-N--0.415700	0	-4.696600	6.240448	2.646256	L		
C-CT--0.025200	-1	-4.664358	7.686168	2.526339	L		
C-C-0.597300	0	-3.766040	8.311101	3.577075	L		
O-O--0.567900	0	-4.243594	8.950484	4.514808	L		
H-H-0.271900	0	-5.496383	5.752073	3.015974	L		
H-H1-0.069800	0	-5.672613	8.076189	2.658222	L		
H-H1-0.069800	0	-4.314342	7.977867	1.534823	L		
N-N--0.415700	0	-2.461819	8.109420	3.420264	L		
C-CT--0.038900	-1	-1.442663	8.547211	4.369049	L		
C-C-0.597300	0	-0.526483	9.619857	3.771356	L		

O-O--0.567900	0	-0.604305	9.961453	2.590049	L
C-CT-0.365400	0	-0.599410	7.390649	4.946129	L
O-OH--0.676100	0	0.548100	7.177061	4.165119	L
C-CT--0.243800	0	-1.339981	6.066976	5.111023	L
H-H-0.271900	0	-2.191419	7.537668	2.623133	L
H-H1-0.100700	0	-1.946836	9.008982	5.216202	L
H-H1-0.004300	0	-0.258232	7.700202	5.934574	L
H-HC-0.064200	0	-2.256961	6.227043	5.676624	L
H-HC-0.064200	0	-0.703522	5.368903	5.649855	L
H-HC-0.064200	0	-1.578744	5.641440	4.136384	L
H-HO-0.410200	0	1.058545	6.440354	4.566148	L
N-N--0.415700	0	0.376339	10.146971	4.602611	L
C-CT--0.025200	-1	1.422639	11.077455	4.189764	L
C-C-0.597300	0	2.305283	10.590459	3.048639	L
O-O--0.567900	0	2.595740	11.353071	2.132190	L
H-H-0.271900	0	0.412702	9.776266	5.537059	L
H-H1-0.069800	0	2.069149	11.293734	5.039254	L
H-H1-0.069800	0	0.956992	12.010317	3.871155	L
N-N--0.516300	0	2.728314	9.331317	3.118066	L
C-CT-0.038100	-1	3.631304	8.727793	2.139353	L
C-C-0.536600	0	2.995750	8.414482	0.796841	L
O-O--0.581900	0	3.720851	8.075281	-0.132679	L
C-CT--0.030300	0	4.211290	7.416449	2.701126	L
C-C-0.799400	-1	5.562811	7.614310	3.365349	L
O-O2--0.801400	-1	6.142013	8.719237	3.285938	L
O-O2--0.801400	-1	6.023548	6.660081	4.024432	L
H-H-0.293600	0	2.394237	8.738623	3.863363	L
H-H1-0.088000	0	4.434208	9.431228	1.915724	L
H-HC--0.012200	0	4.356785	6.688206	1.901531	L
H-HC--0.012200	0	3.510955	6.973163	3.412265	L
N-N--0.415700	0	1.676140	8.521678	0.678667	L
C-CT--0.002400	-1	0.962132	8.320122	-0.574996	L
C-C-0.597300	0	0.364064	9.612852	-1.129648	L
O-O--0.567900	0	0.319939	9.808287	-2.344143	L
C-CT--0.034300	0	-0.109879	7.234044	-0.403494	L
C-CA-0.011800	0	0.403996	5.889171	0.087991	L
C-CA--0.125600	0	1.063397	5.002360	-0.784002	L
C-CA--0.125600	0	0.189439	5.506507	1.422879	L
C-CA--0.170400	0	1.486570	3.743139	-0.316955	L
C-CA--0.170400	0	0.593965	4.244861	1.887087	L
C-CA--0.107200	0	1.236601	3.358843	1.010696	L
H-H-0.271900	0	1.159490	8.760133	1.513021	L
H-H1-0.097800	0	1.652942	7.957249	-1.335409	L
H-HC-0.029500	0	-0.598765	7.076903	-1.365914	L
H-HC-0.029500	0	-0.871269	7.599101	0.287567	L
H-HA-0.133000	0	1.227074	5.282188	-1.813882	L
H-HA-0.133000	0	-0.332455	6.176244	2.083209	L
H-HA-0.143000	0	1.988449	3.054004	-0.976594	L
H-HA-0.143000	0	0.383712	3.950965	2.905205	L
H-HA-0.129700	0	1.523526	2.378859	1.356796	L
N-N--0.4157	0	-0.072610	10.514396	-0.248154	L
C-CT--0.1490	-1	-0.489875	11.852763	-0.610840	L
H-H-0.2719	0	-0.043099	10.263185	0.734665	L
H-H1-0.0976	0	-0.863311	12.370802	0.273042	L

H-H1-0.0976	0	-1.277949	11.799905	-1.363859	L
H-H1-0.0976	0	0.360739	12.400096	-1.019934	L
C-CT--0.3662	-1	7.972068	8.441410	-0.140280	L
C-C-0.5972	0	7.510083	7.658204	-1.345920	L
O-O--0.5679	0	8.328214	7.330101	-2.201332	L
H-HC-0.1123	0	7.504945	9.423791	-0.144694	L
H-HC-0.1123	0	9.054487	8.542888	-0.171829	L
H-HC-0.1123	0	7.688081	7.908341	0.767903	L
N-N--0.415700	0	6.202193	7.399914	-1.436823	L
C-CT--0.051800	-1	5.552284	6.867745	-2.642907	L
C-C-0.597300	0	4.880065	7.975165	-3.453646	L
O-O--0.567900	0	5.180988	8.184018	-4.628646	L
C-CT--0.110200	0	4.635000	5.668946	-2.316974	L
C-CT-0.353100	0	4.761932	4.540180	-3.362442	L
C-CT--0.412100	0	3.912496	3.337086	-2.971188	L
C-CT--0.412100	0	4.319212	4.944983	-4.770914	L
H-H-0.271900	0	5.600766	7.700434	-0.677235	L
H-H1-0.092200	0	6.346717	6.487933	-3.286587	L
H-HC-0.045700	0	3.597863	5.996707	-2.242107	L
H-HC-0.045700	0	4.922945	5.257318	-1.349273	L
H-HC--0.036100	0	5.802183	4.214179	-3.404339	L
H-HC-0.100000	0	2.854402	3.585479	-3.045380	L
H-HC-0.100000	0	4.143642	3.037492	-1.951128	L
H-HC-0.100000	0	4.135980	2.502427	-3.633037	L
H-HC-0.100000	0	3.317804	5.375047	-4.737686	L
H-HC-0.100000	0	4.317244	4.075032	-5.426460	L
H-HC-0.100000	0	5.011497	5.677385	-5.182525	L
N-N--0.4157	0	3.994775	8.705757	-2.789415	L
C-CT--0.1490	-1	3.342040	9.897025	-3.264810	L
H-H-0.2719	0	3.846851	8.431668	-1.825801	L
H-H1-0.0976	0	4.037234	10.492257	-3.858444	L
H-H1-0.0976	0	2.991213	10.488043	-2.417517	L
H-H1-0.0976	0	2.486071	9.622831	-3.883799	L
C-CT--0.3662	-1	-8.731618	2.342491	8.133372	L
C-C-0.5972	0	-7.850961	1.138740	7.952919	L
O-O--0.5679	0	-7.516019	0.465294	8.920453	L
H-HC-0.1123	0	-9.656461	2.189914	7.580490	L
H-HC-0.1123	0	-8.214166	3.221131	7.751362	L
H-HC-0.1123	0	-8.947313	2.470224	9.192142	L
N-N--0.415700	0	-7.469550	0.883827	6.709513	L
C-CT--0.038900	-1	-6.666835	-0.269133	6.296447	L
C-C-0.597300	0	-5.400596	0.217585	5.614336	L
O-O--0.567900	0	-5.437651	0.645428	4.460353	L
C-CT-0.365400	0	-7.473394	-1.234704	5.414637	L
O-OH--0.676100	0	-8.015549	-0.569959	4.299144	L
C-CT--0.243800	0	-8.631514	-1.848756	6.206563	L
H-H-0.271900	0	-7.808524	1.500863	5.975362	L
H-H1-0.100700	0	-6.366492	-0.833129	7.179323	L
H-H1-0.004300	0	-6.816289	-2.034080	5.069983	L
H-HC-0.064200	0	-8.242814	-2.380175	7.074934	L
H-HC-0.064200	0	-9.183403	-2.546578	5.582992	L
H-HC-0.064200	0	-9.318314	-1.069549	6.535427	L
H-HO-0.410200	0	-7.319899	0.015444	3.966983	L
N-N--0.415700	0	-4.309946	0.212696	6.384658	L

C-CT--0.059700	-1	-3.115011	1.030236	6.164089	L		
C-C-0.597300	0	-1.865938	0.154835	6.093314	L		
O-O--0.567900	0	-1.607360	-0.649392	6.992758	L		
C-CT-0.130300	0	-2.990854	2.106256	7.268279	L		
C-CT--0.043000	0	-4.248651	2.996628	7.428074	L		
C-CT--0.320400	0	-1.754024	2.985036	7.025315	L		
C-CT--0.066000	0	-4.681645	3.780548	6.179900	L		
H-H-0.271900	0	-4.380321	-0.220537	7.291319	L		
H-H1-0.086900	0	-3.220241	1.553909	5.218350	L		
H-HC-0.018700	0	-2.843170	1.594873	8.221417	L		
H-HC-0.023600	0	-4.065492	3.712519	8.230154	L		
H-HC-0.023600	0	-5.085137	2.376652	7.749616	L		
H-HC-0.088200	0	-1.755822	3.346975	5.997189	L		
H-HC-0.088200	0	-0.848435	2.398280	7.183033	L		
H-HC-0.088200	0	-1.742431	3.827800	7.715393	L		
H-HC-0.018600	0	-3.883331	4.442397	5.848467	L		
H-HC-0.018600	0	-5.558673	4.381960	6.419495	L		
H-HC-0.018600	0	-4.942556	3.098784	5.373510	L		
N-N--0.516300	0	-1.113910	0.336808	5.013141	L		
C-CT-0.038100	-1	0.182582	-0.249395	4.726448	L	H-HC	178
C-C-0.536600	0	1.126970	-0.092671	5.923447	L		
O-O--0.581900	0	1.345173	1.001535	6.436211	L		
C-CT--0.520019	0	0.670199	0.395513	3.414965	H		
C-C-0.636404	0	0.995919	-0.571524	2.290349	H		
O-O2--0.608036	0	0.429268	-1.701284	2.222952	H		
O-O2--0.608131	0	1.824105	-0.111368	1.436151	H		
H-H-0.293600	0	-1.510368	0.940532	4.300152	L		
H-H1-0.088000	0	0.025395	-1.317567	4.570729	L		
H-HC-0.132300	0	1.553483	1.005589	3.615926	H		
H-HC-0.188993	0	-0.093602	1.073244	3.018703	H		
N-N--0.415700	0	1.667181	-1.204768	6.389372	L		
C-CT-0.033700	-1	2.567440	-1.255297	7.518582	L		
C-C-0.597300	0	3.788541	-2.073714	7.114728	L		
O-O--0.567900	0	3.836854	-3.299975	7.262121	L		
C-CT--0.182500	0	1.811596	-1.820145	8.729541	L		
H-H-0.271900	0	1.360440	-2.082557	5.984846	L		
H-H1-0.082300	0	2.911456	-0.251154	7.773957	L		
H-HC-0.060300	0	0.971082	-1.167504	8.968850	L		
H-HC-0.060300	0	2.482540	-1.874157	9.586626	L		
H-HC-0.060300	0	1.436099	-2.818948	8.501199	L		
N-N--0.4157	0	4.758719	-1.375955	6.536559	L		
C-CT-0.0188	-1	5.964056	-1.955934	5.988536	L	H-HC	200
C-C-0.5973	0	7.161521	-1.806942	6.936022	L		
O-O--0.5679	0	7.208542	-0.955332	7.826029	L		
C-CT--0.517456	0	6.216694	-1.317460	4.620424	H		
C-CM-0.287351	0	5.348366	-1.798520	3.516449	H		
N-N2--0.586994	0	5.496675	-3.051160	2.937097	H		
C-CM--0.020338	0	4.367281	-1.192836	2.782274	H		
C-CM-0.242458	0	4.630588	-3.142135	1.892519	H		
N-N2--0.505070	0	3.937059	-2.025921	1.769861	H		
H-H-0.2719	0	4.654928	-0.366390	6.483505	L		
H-H1-0.0881	0	5.819205	-3.022386	5.846743	L		
H-HC-0.166355	0	7.272228	-1.479774	4.357014	H		
H-HC-0.174481	0	6.099949	-0.239134	4.755452	H		

H-H-0.335795	0	6.147126	-3.766543	3.254702	H		
H-H4-0.152777	0	3.961717	-0.200339	2.907660	H		
H-H5-0.209475	0	4.534622	-4.008565	1.253136	H		
N-N--0.4157	0	8.166088	-2.645678	6.689493	L		
C-CT--0.1490	-1	9.435536	-2.575021	7.379757	L		
H-H-0.2719	0	8.014713	-3.324565	5.950083	L		
H-H1-0.0976	0	10.108496	-3.344469	7.000139	L		
H-H1-0.0976	0	9.278413	-2.723203	8.449568	L		
H-H1-0.0976	0	9.882779	-1.591810	7.222527	L		
C-CT--0.3662	-1	8.367291	-9.381643	0.905241	L		
C-C-0.5972	0	8.738057	-9.137622	-0.545038	L		
O-O--0.5679	0	9.756688	-8.508092	-0.803206	L		
H-HC-0.1123	0	8.307100	-10.450389	1.096973	L		
H-HC-0.1123	0	7.419410	-8.894379	1.126169	L		
H-HC-0.1123	0	9.139803	-8.943006	1.536779	L		
N-N--0.254800	0	7.927580	-9.607172	-1.510021	L		
C-CT--0.026600	-1	8.168814	-9.365550	-2.934763	L		
C-C-0.589600	0	8.014204	-7.892760	-3.315123	L		
O-O--0.574800	0	8.720846	-7.393768	-4.187820	L		
C-CT--0.007000	0	7.167329	-10.263003	-3.669510	L		
C-CT-0.018900	0	6.010933	-10.403470	-2.678426	L		
C-CT-0.019200	0	6.706833	-10.378221	-1.316850	L		
H-H1-0.064100	0	9.182861	-9.671336	-3.195484	L		
H-HC-0.025300	0	7.618661	-11.241813	-3.838734	L		
H-HC-0.025300	0	6.840465	-9.829927	-4.616179	L		
H-HC-0.021300	0	5.456981	-11.330169	-2.831234	L		
H-HC-0.021300	0	5.345593	-9.543050	-2.765643	L		
H-H1-0.039100	0	6.055034	-9.919418	-0.572030	L		
H-H1-0.039100	0	6.965862	-11.394289	-1.016117	L		
N-N--0.516300	0	7.092613	-7.198467	-2.646971	L		
C-CT-0.039700	-1	6.840269	-5.771249	-2.816200	L		
C-C-0.536600	0	8.098829	-4.905963	-2.683304	L		
O-O--0.581900	0	8.270544	-3.918674	-3.402003	L		
C-CT-0.056000	0	5.739330	-5.336480	-1.843504	L	H-HC	244
C-CT--0.515956	0	5.210263	-3.947438	-2.212774	H		
C-C-0.587828	0	4.112359	-3.421279	-1.296527	H		
O-O2--0.600319	0	4.050331	-2.139399	-1.250359	H		
O-O2--0.589127	0	3.356348	-4.216780	-0.694581	H		
H-H-0.293600	0	6.564159	-7.676541	-1.936344	L		
H-H1-0.110500	0	6.466896	-5.616128	-3.828799	L		
H-HC--0.017300	0	6.130479	-5.324645	-0.824996	L		
H-HC--0.017300	0	4.915943	-6.050587	-1.898317	L		
H-HC-0.167759	0	4.810451	-3.988401	-3.233206	H		
H-HC-0.135230	0	6.032624	-3.223641	-2.214870	H		
N-N--0.415700	0	9.010419	-5.313970	-1.802683	L		
C-CT--0.024900	-1	10.247270	-4.609839	-1.484960	L		
C-C-0.597300	0	11.266316	-4.490332	-2.611152	L		
O-O--0.567900	0	12.239256	-3.747706	-2.479411	L		
C-CT-0.211700	0	10.891431	-5.246494	-0.247474	L		
O-OH--0.654600	0	9.937372	-5.435008	0.787267	L		
H-H-0.271900	0	8.805972	-6.128445	-1.240028	L		
H-H1-0.084300	0	9.977068	-3.587779	-1.215168	L		
H-H1-0.035200	0	11.691906	-4.598910	0.113257	L		
H-H1-0.035200	0	11.316338	-6.214740	-0.516377	L		

H-HO-0.427500	0	9.382545	-4.627016	0.862998	L
N-N--0.415700	0	11.031332	-5.177200	-3.727367	L
C-CT-0.033700	-1	11.780429	-5.009851	-4.965929	L
C-C-0.597300	0	10.955073	-4.432083	-6.119666	L
O-O--0.567900	0	11.399667	-4.414597	-7.266511	L
C-CT--0.182500	0	12.389457	-6.375324	-5.316140	L
H-H-0.271900	0	10.223126	-5.789027	-3.733880	L
H-H1-0.082300	0	12.605630	-4.314602	-4.805740	L
H-HC-0.060300	0	13.003378	-6.729884	-4.487451	L
H-HC-0.060300	0	13.015022	-6.282587	-6.204627	L
H-HC-0.060300	0	11.593996	-7.096148	-5.512081	L
N-N--0.415700	0	9.767396	-3.932384	-5.796126	L
C-CT--0.025200	-1	8.863699	-3.252555	-6.701219	L
C-C-0.597300	0	8.797261	-1.750256	-6.476245	L
O-O--0.567900	0	8.781687	-0.975888	-7.430204	L
H-H-0.271900	0	9.479864	-4.008739	-4.826720	L
H-H1-0.069800	0	7.860186	-3.654940	-6.566652	L
H-H1-0.069800	0	9.161212	-3.428044	-7.735544	L
N-N--0.415700	0	8.754263	-1.346316	-5.209709	L
C-CT--0.002400	-1	8.577797	0.037479	-4.787129	L
C-C-0.597300	0	9.685864	0.469553	-3.820890	L
O-O--0.567900	0	10.297249	-0.382822	-3.168493	L
C-CT--0.034300	0	7.185513	0.160552	-4.141381	L
C-CA-0.011800	0	6.047366	-0.094111	-5.112572	L
C-CA--0.125600	0	5.657054	0.909667	-6.019542	L
C-CA--0.125600	0	5.411492	-1.349574	-5.146983	L
C-CA--0.170400	0	4.647347	0.654614	-6.965366	L
C-CA--0.170400	0	4.397861	-1.602471	-6.088353	L
C-CA--0.107200	0	4.021095	-0.604086	-7.003842	L
H-H-0.271900	0	8.780562	-2.053530	-4.484588	L
H-H1-0.097800	0	8.615296	0.689603	-5.659859	L
H-HC-0.029500	0	7.058625	1.156590	-3.718034	L
H-HC-0.029500	0	7.120105	-0.548452	-3.314444	L
H-HA-0.133000	0	6.142543	1.874663	-6.001540	L
H-HA-0.133000	0	5.712425	-2.126334	-4.459302	L
H-HA-0.143000	0	4.354068	1.425793	-7.663494	L
H-HA-0.143000	0	3.909181	-2.565708	-6.110050	L
H-HA-0.129700	0	3.241279	-0.800537	-7.726520	L
N-N--0.254800	0	9.948923	1.782151	-3.680984	L
C-CT--0.026600	-1	10.812780	2.275385	-2.614918	L
C-C-0.589600	0	10.177570	2.028088	-1.238020	L
O-O--0.574800	0	8.982191	1.725625	-1.160182	L
C-CT--0.007000	0	10.976659	3.774351	-2.898722	L
C-CT-0.018900	0	9.662964	4.145935	-3.584319	L
C-CT-0.019200	0	9.351834	2.897474	-4.409662	L
H-H1-0.064100	0	11.781134	1.778972	-2.669859	L
H-HC-0.025300	0	11.804297	3.924738	-3.593422	L
H-HC-0.025300	0	11.133084	4.359236	-1.990988	L
H-HC-0.021300	0	9.765760	5.033014	-4.210097	L
H-HC-0.021300	0	8.884446	4.295296	-2.833474	L
H-H1-0.039100	0	8.273550	2.792434	-4.520840	L
H-H1-0.039100	0	9.822015	2.978918	-5.390508	L
N-N--0.254800	0	10.943466	2.138162	-0.142323	L
C-CT--0.026600	-1	10.390029	2.121441	1.204575	L

C-C-0.589600	0	9.336431	3.204198	1.440236	L		
O-O--0.574800	0	9.593309	4.384513	1.214683	L		
C-CT--0.007000	0	11.589220	2.305061	2.145466	L		
C-CT-0.018900	0	12.776770	1.820630	1.317227	L		
C-CT-0.019200	0	12.393412	2.240632	-0.098841	L		
H-H1-0.064100	0	9.950574	1.138734	1.381816	L		
H-HC-0.025300	0	11.472729	1.724083	3.060897	L		
H-HC-0.025300	0	11.730746	3.360923	2.383586	L		
H-HC-0.021300	0	12.841869	0.732472	1.369689	L		
H-HC-0.021300	0	13.712009	2.280396	1.637592	L		
H-H1-0.039100	0	12.690850	3.275436	-0.273931	L		
H-H1-0.039100	0	12.870213	1.576402	-0.820410	L		
N-N--0.4157	0	8.169530	2.777705	1.913497	L		
C-CT-0.0188	-1	7.011072	3.604876	2.204911	L	H-HC	334
C-C-0.5973	0	6.144079	3.009801	3.312258	L		
O-O--0.5679	0	6.151598	1.798409	3.530080	L		
C-CT--0.515368	0	6.194635	3.720572	0.889863	H		
C-CM-0.287903	0	5.601222	2.440804	0.378673	H		
N-N2--0.582625	0	6.221756	1.657498	-0.581230	H		
C-CM--0.036893	0	4.482546	1.717152	0.721610	H		
C-CM-0.243454	0	5.489239	0.530135	-0.772191	H		
N-N2--0.523998	0	4.429050	0.531747	0.014514	H		
H-H-0.2719	0	8.035052	1.780387	1.998207	L		
H-H1-0.0881	0	7.342198	4.600138	2.516756	L		
H-HC-0.162301	0	6.852325	4.173841	0.133908	H		
H-HC-0.188340	0	5.399300	4.455238	1.066911	H		
H-H-0.339448	0	7.129455	1.862313	-0.998773	H		
H-H4-0.185957	0	3.720125	1.971364	1.442224	H		
H-H5-0.211295	0	5.759599	-0.258597	-1.458931	H		
N-N--0.415700	0	5.373194	3.871332	3.963779	L		
C-CT-0.014300	-1	4.546261	3.582481	5.126400	L		
C-C-0.597300	0	5.198837	2.628989	6.137539	L		
O-O--0.567900	0	4.673572	1.576536	6.504643	L		
C-CT--0.204100	0	3.082423	3.334900	4.740021	L		
C-C-0.713000	0	2.186079	4.173571	5.633814	L		
N-N--0.919100	0	1.711435	3.658481	6.734043	L		
O-O--0.593100	0	1.941333	5.341811	5.389198	L		
H-H-0.271900	0	5.516080	4.859694	3.747593	L		
H-H1-0.104800	0	4.538455	4.537704	5.656805	L		
H-HC-0.079700	0	2.846532	2.280336	4.835137	L		
H-HC-0.079700	0	2.905451	3.644583	3.710552	L		
H-H-0.419600	0	1.079267	4.217065	7.265740	L		
H-H-0.419600	0	1.778071	2.645516	6.835181	L		
N-N--0.4157	0	6.386038	3.059838	6.577260	L		
C-CT--0.1490	-1	7.292337	2.320582	7.434412	L		
H-H-0.2719	0	6.697669	3.934999	6.179290	L		
H-H1-0.0976	0	8.038122	2.989426	7.863702	L		
H-H1-0.0976	0	7.790723	1.542206	6.853487	L		
H-H1-0.0976	0	6.732249	1.835939	8.237306	L		
C-CT--0.3662	-1	-2.249635	-2.764975	9.534783	L		
C-C-0.5972	0	-2.376222	-3.853316	8.504167	L		
O-O--0.5679	0	-2.922377	-4.914593	8.792445	L		
H-HC-0.1123	0	-1.195365	-2.548677	9.696436	L		
H-HC-0.1123	0	-2.705979	-3.099626	10.464015	L		

H-HC-0.1123	0	-2.756385	-1.871682	9.174616	L		
N-N--0.347900	0	-1.844570	-3.592191	7.313426	L		
C-CT--0.240000	-1	-1.741109	-4.556858	6.212241	L		
C-C-0.734100	0	-0.333440	-4.586311	5.629685	L		
O-O--0.589400	0	0.476233	-3.697264	5.876073	L		
C-CT--0.009400	0	-2.835430	-4.268955	5.164256	L		
C-CT-0.018700	0	-2.623060	-2.967555	4.381336	L	H-HC	379
C-CT--0.464524	0	-3.698092	-2.789854	3.302824	H		
C-CT--0.195159	0	-3.578388	-1.476034	2.540446	H		
N-N3--0.748751	0	-2.337190	-1.383600	1.704141	H		
H-H-0.274700	0	-1.448293	-2.664395	7.179305	L		
H-H1-0.142600	0	-1.925480	-5.553960	6.613005	L		
H-HC-0.036200	0	-3.803909	-4.228459	5.665786	L		
H-HC-0.036200	0	-2.873247	-5.088520	4.453762	L		
H-HC-0.010300	0	-1.641399	-2.978243	3.907717	L		
H-HC-0.010300	0	-2.682514	-2.130285	5.069334	L		
H-HC-0.173903	0	-4.678228	-2.807571	3.788282	H		
H-HC-0.148535	0	-3.661267	-3.643608	2.612141	H		
H-HP-0.236066	0	-3.584107	-0.599584	3.187667	H		
H-HP-0.172509	0	-4.413757	-1.378564	1.835888	H		
H-H-0.437293	0	-2.142702	-0.363004	1.396303	H		
H-H-0.366736	0	-2.464171	-1.926952	0.840286	H		
H-H-0.419451	0	-1.460043	-1.687628	2.166768	H		
N-N--0.415700	0	-0.032395	-5.622559	4.860068	L		
C-CT--0.025200	-1	1.281899	-5.817143	4.256842	L		
C-C-0.597300	0	1.161214	-6.532683	2.910444	L		
O-O--0.567900	0	1.757313	-7.581395	2.674991	L		
H-H-0.271900	0	-0.770800	-6.284448	4.640486	L		
H-H1-0.069800	0	1.904733	-6.417555	4.920078	L		
H-H1-0.069800	0	1.771928	-4.857378	4.089290	L		
N-N--0.415700	0	0.338303	-5.951529	2.042603	L		
C-CT--0.025200	-1	-0.227257	-6.545107	0.834563	L		
C-C-0.597300	0	-1.363576	-5.656597	0.390971	L		
O-O--0.567900	0	-2.240480	-5.375440	1.200167	L		
H-H-0.271900	0	-0.113355	-5.110900	2.377243	L		
H-H1-0.069800	0	-0.612795	-7.540011	1.057253	L		
H-H1-0.069800	0	0.538782	-6.605938	0.061028	L		
N-N--0.4157	0	-1.327819	-5.204720	-0.862202	L		
C-CT--0.0014	-1	-2.156065	-4.110766	-1.374254	L		
C-C-0.5973	0	-3.622399	-4.184483	-0.931628	L		
O-O--0.5679	0	-4.189060	-3.215854	-0.434760	L		
C-CT--0.0152	0	-2.051217	-4.065269	-2.912657	L		
C-CA--0.0011	0	-0.662002	-4.277112	-3.490093	L		
C-CA--0.1906	0	0.362267	-3.343269	-3.241773	L		
C-CA--0.1906	0	-0.397797	-5.413676	-4.281114	L		
C-CA--0.2341	0	1.647974	-3.547500	-3.778559	L		
C-CA--0.2341	0	0.886208	-5.620135	-4.820453	L		
C-C-0.3226	0	1.913271	-4.684520	-4.568276	L		
O-OH--0.5579	0	3.149025	-4.881366	-5.099631	L		
H-H-0.2719	0	-0.514290	-5.429139	-1.417321	L		
H-H1-0.0876	0	-1.739210	-3.186889	-0.978006	L		
H-HC-0.0295	0	-2.414299	-3.101342	-3.262437	L		
H-HC-0.0295	0	-2.715774	-4.820795	-3.332680	L		
H-HA-0.1699	0	0.168521	-2.461974	-2.640410	L		

H-HA-0.1699	0	-1.188144	-6.119544	-4.491411	L
H-HA-0.1656	0	2.435010	-2.835112	-3.585273	L
H-HA-0.1656	0	1.080397	-6.484427	-5.434385	L
H-HO-0.3992	0	3.167585	-5.666890	-5.643985	L
N-N--0.415700	0	-4.218625	-5.367877	-1.091892	L
C-CT--0.024900	-1	-5.554753	-5.658532	-0.593248	L
C-C-0.597300	0	-5.683116	-5.578073	0.925993	L
O-O--0.567900	0	-5.044592	-6.331119	1.670254	L
C-CT-0.211700	0	-6.014519	-7.054618	-1.017816	L
O-OH--0.654600	-1	-7.272984	-7.361075	-0.433052	L
H-H-0.271900	0	-3.642216	-6.108724	-1.448557	L
H-H1-0.084300	0	-6.242748	-4.937702	-1.035354	L
H-H1-0.035200	0	-5.281211	-7.790580	-0.686003	L
H-H1-0.035200	0	-6.090276	-7.096830	-2.104395	L
H-HO-0.427500	-1	-7.529484	-8.233514	-0.741844	L
N-N--0.415700	0	-6.660714	-4.782270	1.353217	L
C-CT-0.033700	-1	-7.153374	-4.689629	2.716301	L
C-C-0.597300	0	-7.503150	-6.018585	3.390969	L
O-O--0.567900	0	-7.427521	-6.126672	4.612967	L
C-CT--0.182500	0	-8.347147	-3.734527	2.723766	L
H-H-0.271900	0	-7.123986	-4.205255	0.657803	L
H-H1-0.082300	0	-6.366195	-4.240107	3.320493	L
H-HC-0.060300	0	-8.062841	-2.768821	2.301813	L
H-HC-0.060300	0	-8.685338	-3.591131	3.745434	L
H-HC-0.060300	0	-9.163908	-4.161566	2.147764	L
N-N--0.415700	0	-7.897998	-7.022606	2.607422	L
C-CT--0.025200	-1	-8.274267	-8.336983	3.113405	L
C-C-0.597300	0	-7.314311	-9.460861	2.743944	L
O-O--0.567900	0	-7.729415	-10.614002	2.652132	L
H-H-0.271900	0	-7.887935	-6.853968	1.607945	L
H-H1-0.069800	0	-9.254153	-8.592916	2.711478	L
H-H1-0.069800	0	-8.362031	-8.320368	4.200002	L
N-N--0.415700	0	-6.030029	-9.147945	2.577343	L
C-CT--0.024900	-1	-4.955744	-10.140997	2.588214	L
C-C-0.597300	0	-4.253805	-10.105439	3.939843	L
O-O--0.567900	0	-3.933357	-9.030313	4.456136	L
C-CT-0.211700	0	-3.965717	-9.847472	1.456453	L
O-OH--0.654600	-1	-3.076126	-10.937283	1.269131	L
H-H-0.271900	0	-5.776542	-8.167689	2.657185	L
H-H1-0.084300	0	-5.367570	-11.136696	2.423097	L
H-H1-0.035200	0	-3.404115	-8.938741	1.679814	L
H-H1-0.035200	0	-4.524743	-9.694031	0.532577	L
H-HO-0.427500	-1	-2.535821	-10.747110	0.498705	L
N-N--0.4157	0	-4.054165	-11.283144	4.530565	L
C-CT--0.1490	-1	-3.517609	-11.430200	5.872468	L
H-H-0.2719	0	-4.332783	-12.102447	4.014374	L
H-H1-0.0976	0	-3.314401	-12.479440	6.086876	L
H-H1-0.0976	0	-4.232458	-11.044576	6.602725	L
H-H1-0.0976	0	-2.590388	-10.859897	5.967298	L
C-CT--0.3662	-1	-12.149536	5.064516	-0.643094	L
C-C-0.5972	0	-10.720308	5.242094	-0.203061	L
O-O--0.5679	0	-10.402121	4.963854	0.947704	L
H-HC-0.1123	0	-12.542899	6.020341	-0.982282	L
H-HC-0.1123	0	-12.735317	4.706155	0.201835	L

H-HC-0.1123	0	-12.185694	4.330815	-1.446293	L		
N-N--0.415700	0	-9.871876	5.694129	-1.121854	L		
C-CT--0.025200	-1	-8.438803	5.860404	-0.894704	L		
C-C-0.597300	0	-7.622775	5.727335	-2.156734	L		
O-O--0.567900	0	-8.132503	6.011204	-3.247122	L		
H-H-0.271900	0	-10.186954	5.862043	-2.065120	L		
H-H1-0.069800	0	-8.125356	5.084955	-0.206141	L		
H-H1-0.069800	0	-8.238913	6.833170	-0.447643	L		
N-N--0.415700	0	-6.362313	5.321020	-1.981035	L	H-H1	489
C-CT--0.530209	-1	-5.353712	5.292698	-3.040570	H		
C-C-0.568093	0	-4.520706	4.026323	-2.934579	H		
O-O--0.528105	0	-4.329029	3.471206	-1.838905	H		
C-CT-0.298500	0	-4.468348	6.562338	-3.001308	L	H-H1	489
C-CT--0.319200	0	-5.279689	7.812110	-3.348873	L		
C-CT--0.319200	0	-3.776239	6.771964	-1.650997	L		
H-H-0.271900	0	-6.063405	5.055098	-1.044432	L		
H-H1-0.184096	0	-5.883396	5.308022	-3.999245	H		
H-HC--0.029700	0	-3.694993	6.463900	-3.761850	L		
H-HC-0.079100	0	-6.007779	8.016334	-2.565636	L		
H-HC-0.079100	0	-5.799678	7.657831	-4.293307	L		
H-HC-0.079100	0	-4.607906	8.664254	-3.443831	L		
H-HC-0.079100	0	-4.510900	6.898748	-0.857369	L		
H-HC-0.079100	0	-3.138339	7.653312	-1.694731	L		
H-HC-0.079100	0	-3.154658	5.908322	-1.413795	L		
N-N--0.610103	0	-3.949413	3.575984	-4.067637	H		
C-CT--0.314060	-1	-4.240469	4.006026	-5.419715	H		
C-C-0.597300	0	-5.363916	3.181476	-6.035357	L	H-H1	505
O-O--0.567900	0	-5.497767	1.980630	-5.799279	L		
C-CT--0.110200	0	-2.967407	3.925466	-6.299167	L	H-H1	505
C-CT-0.353100	0	-1.973748	5.097327	-6.114664	L		
C-CT--0.412100	0	-1.105268	5.009435	-4.858415	L		
C-CT--0.412100	0	-1.021054	5.158174	-7.312546	L		
H-H-0.398207	0	-3.321500	2.775274	-3.975445	H		
H-H1-0.165130	0	-4.559416	5.048384	-5.421999	H		
H-HC-0.045700	0	-3.291507	3.938176	-7.340025	L		
H-HC-0.045700	0	-2.456623	2.977418	-6.138204	L		
H-HC--0.036100	0	-2.533571	6.032917	-6.089667	L		
H-HC-0.100000	0	-0.505802	4.101404	-4.882245	L		
H-HC-0.100000	0	-1.736130	4.991265	-3.971014	L		
H-HC-0.100000	0	-0.451096	5.877823	-4.793227	L		
H-HC-0.100000	0	-0.443635	4.236386	-7.372146	L		
H-HC-0.100000	0	-0.340353	6.001284	-7.195328	L		
H-HC-0.100000	0	-1.589945	5.291666	-8.231154	L		
N-N--0.415700	0	-6.146189	3.829325	-6.896354	L		
C-CT--0.038900	-1	-7.330921	3.237401	-7.529321	L		
C-C-0.597300	0	-7.076536	1.899357	-8.211659	L		
O-O--0.567900	0	-7.944109	1.029161	-8.230444	L		
C-CT-0.365400	-1	-7.969366	4.163233	-8.589126	L		
O-OH--0.676100	-1	-7.909192	5.519601	-8.138459	L		
C-CT--0.243800	0	-9.445934	3.866343	-8.848806	L		
H-H-0.271900	0	-6.013085	4.824831	-6.997483	L		
H-H1-0.100700	0	-8.074529	3.093596	-6.752245	L		
H-H1-0.004300	0	-7.413514	4.089249	-9.524798	L		
H-HC-0.064200	0	-9.556542	2.859825	-9.252252	L		

```
H-HC-0.064200    0   -9.841602    4.575353   -9.575906 L
H-HC-0.064200    0  -10.011711    3.939915   -7.919248 L
H-HO-0.410200   -1   -8.807772    5.853702   -8.102225 L
N-N--0.415700    0   -5.905020    1.771767   -8.832024 L
C-CT--0.025200  -1   -5.521015    0.614437   -9.628337 L
C-C-0.597300     0   -4.617015   -0.388601   -8.941884 L
O-O--0.567900    0   -4.101326   -1.275363   -9.622005 L
H-H-0.271900     0   -5.228272    2.502639   -8.673698 L
H-H1-0.069800    0   -4.999273    0.962497  -10.518748 L
H-H1-0.069800    0   -6.410532    0.077894   -9.958629 L
N-N--0.415700    0   -4.416494   -0.227357   -7.629588 L H-H1    545
C-CT--0.539097  -1   -3.475596   -0.999911   -6.827804 H
C-C-0.572106     0   -4.214758   -1.459572   -5.571651 H
O-O--0.533001    0   -4.948423   -2.441517   -5.651079 H
C-CT-0.130300    0   -2.137422   -0.238246   -6.629857 L H-H1    545
C-CT--0.043000   0   -1.524344    0.256370   -7.961979 L
C-CT--0.320400   0   -1.165697   -1.193942   -5.915336 L
C-CT--0.066000   0   -0.212115    1.037080   -7.809010 L
H-H-0.271900     0   -4.899874    0.531641   -7.159020 L
H-H1-0.180646    0   -3.251006   -1.916522   -7.383320 H
H-HC-0.018700    0   -2.317619    0.629548   -5.999276 L
H-HC-0.023600    0   -2.229615    0.930513   -8.446343 L
H-HC-0.023600    0   -1.357067   -0.594854   -8.623185 L
H-HC-0.088200    0   -0.960703   -2.060218   -6.543224 L
H-HC-0.088200    0   -1.586859   -1.533227   -4.971082 L
H-HC-0.088200    0   -0.232957   -0.688107   -5.680020 L
H-HC-0.018600    0   -0.343496    1.838797   -7.083171 L
H-HC-0.018600    0    0.062387    1.470618   -8.770726 L
H-HC-0.018600    0    0.592327    0.376978   -7.485908 L
N-N--0.611145    0   -4.068413   -0.711579   -4.444315 H
C-CT--0.109296  -1   -4.639724   -1.110257   -3.165690 H
C-C-0.597300     0   -6.165726   -1.120660   -3.173696 L H-H1    564
O-O--0.567900    0   -6.798216   -2.040204   -2.642861 L
C-CT--0.388038   0   -4.119795   -0.282799   -2.007938 H
H-H-0.369964     0   -3.395689    0.045100   -4.410281 H
H-H1-0.178541    0   -4.385917   -2.158129   -2.972191 H
H-H1-0.157964    0   -4.718328   -0.558770   -1.134147 H
H-H1-0.179833    0   -4.247379    0.789207   -2.178325 H
H-HS-0.319500    0   -2.229889    0.259747   -0.695154 H
N-N--0.415700    0   -6.756476   -0.130483   -3.831190 L
C-CT--0.087500  -1   -8.197579   -0.072445   -4.059419 L
C-C-0.597300     0   -8.681567   -1.222685   -4.928762 L
O-O--0.567900    0   -9.609804   -1.932916   -4.544719 L
C-CT-0.298500    0   -8.611722    1.291341   -4.630112 L
C-CT--0.319200   0  -10.130134    1.406495   -4.820193 L
C-CT--0.319200   0   -8.174213    2.456898   -3.729052 L
H-H-0.271900     0   -6.127862    0.541974   -4.263467 L
H-H1-0.096900    0   -8.691450   -0.182801   -3.095950 L
H-HC--0.029700   0   -8.136526    1.395668   -5.596690 L
H-HC-0.079100    0  -10.636773    1.240511   -3.869132 L
H-HC-0.079100    0  -10.474835    0.670338   -5.546276 L
H-HC-0.079100    0  -10.379746    2.398724   -5.195162 L
H-HC-0.079100    0   -8.626292    2.368035   -2.742682 L
H-HC-0.079100    0   -8.466754    3.406933   -4.175350 L
```

H-HC-0.079100	0	-7.089725	2.468807	-3.614064	L
N-N--0.415700	0	-8.055991	-1.421592	-6.087483	L
C-CT--0.025200	-1	-8.405277	-2.483888	-7.026783	L
C-C-0.597300	0	-8.318961	-3.883959	-6.432240	L
O-O--0.567900	0	-9.222731	-4.695642	-6.617746	L
H-H-0.271900	0	-7.276657	-0.813938	-6.303961	L
H-H1-0.069800	0	-7.731967	-2.435085	-7.882326	L
H-H1-0.069800	0	-9.423597	-2.324862	-7.381159	L
N-N--0.415700	0	-7.232754	-4.161914	-5.713842	L
C-CT--0.025200	-1	-7.007721	-5.428830	-5.028631	L
C-C-0.597300	0	-7.930492	-5.691389	-3.849455	L
O-O--0.567900	0	-8.247383	-6.845074	-3.566359	L
H-H-0.271900	0	-6.514481	-3.442329	-5.653992	L
H-H1-0.069800	0	-5.981449	-5.450735	-4.663896	L
H-H1-0.069800	0	-7.133686	-6.243888	-5.741574	L
N-N--0.415700	0	-8.329381	-4.633781	-3.146306	L
C-CT--0.038900	-1	-9.378505	-4.699305	-2.126355	L
C-C-0.597300	0	-10.748743	-4.993235	-2.728108	L
O-O--0.567900	0	-11.491737	-5.827369	-2.211245	L
C-CT-0.365400	0	-9.421091	-3.400625	-1.306649	L
O-OH--0.676100	0	-8.182200	-3.223664	-0.657856	L
C-CT--0.243800	0	-10.501056	-3.423904	-0.225584	L
H-H-0.271900	0	-7.960410	-3.726153	-3.406591	L
H-H1-0.100700	0	-9.148003	-5.516427	-1.442782	L
H-H1-0.004300	0	-9.599078	-2.548944	-1.963277	L
H-HC-0.064200	0	-11.484695	-3.321187	-0.682663	L
H-HC-0.064200	0	-10.347868	-2.596422	0.466590	L
H-HC-0.064200	0	-10.459302	-4.366321	0.318723	L
H-HO-0.410200	0	-7.598550	-2.766814	-1.297321	L
N-N--0.4157	0	-11.098340	-4.286854	-3.804327	L
C-CT--0.1490	-1	-12.331554	-4.475800	-4.539638	L
H-H-0.2719	0	-10.430848	-3.605635	-4.154594	L
H-H1-0.0976	0	-12.404220	-3.726556	-5.328899	L
H-H1-0.0976	0	-13.183363	-4.383572	-3.864415	L
H-H1-0.0976	0	-12.334764	-5.470621	-4.988341	L
N-N2--0.511415	0	1.990885	0.013178	-1.702423	H
C-CA-0.057801	0	0.835895	0.694342	-1.663551	H
C-CA-0.063496	0	0.290110	1.311659	-2.793852	H
C-CA--0.109122	0	0.988464	1.223438	-3.999205	H
C-CA--0.164490	0	2.194600	0.541079	-4.033050	H
C-CA-0.098392	0	2.656132	-0.056504	-2.866439	H
C-C-0.541263	0	-1.055448	1.971720	-2.838726	H
O-O--0.574884	0	-1.685443	1.920797	-3.911187	H
N-N--0.751108	0	-1.491989	2.628590	-1.752104	H
H-H4-0.220889	0	0.338472	0.729287	-0.696964	H
H-HA-0.160434	0	0.564251	1.682498	-4.883954	H
H-HA-0.142191	0	2.774116	0.470939	-4.944902	H
H-H4-0.174502	0	3.576778	-0.628588	-2.847268	H
H-H-0.412468	0	-2.473371	2.924441	-1.734689	H
H-H-0.353801	0	-1.085229	2.465606	-0.836151	H
Zn-ZN-0.974537	0	2.828696	-1.110871	0.005810	H
O-OW--0.834000	0	-2.126326	-7.253582	3.473219	L
H-HW-0.417000	0	-2.587594	-6.669050	2.852517	L
H-HW-0.417000	0	-2.832931	-7.819432	3.826385	L

O-OW--0.783903	0	1.313375	-2.588121	-0.132754	H
H-HW-0.401253	0	0.948764	-2.488196	0.784545	H
H-HW-0.424111	0	1.924178	-3.366840	-0.189701	H
O-OW--0.834000	0	-3.603239	-8.456231	7.198162	L
H-HW-0.417000	0	-3.703318	-7.517413	7.375232	L
H-HW-0.417000	0	-3.747044	-8.532804	6.243025	L
O-OW--0.834000	0	7.172960	-0.013463	1.797977	L
H-HW-0.417000	0	6.866526	0.391490	2.629299	L
H-HW-0.417000	0	6.381795	0.070452	1.243014	L
O-OW--0.834000	0	7.062605	-4.950581	4.750835	L
H-HW-0.417000	0	6.113794	-5.022881	4.980756	L
H-HW-0.417000	0	7.285940	-5.834640	4.456724	L
O-OW--0.834000	0	8.573746	-0.920996	-0.303223	L
H-HW-0.417000	0	8.137240	-0.541965	0.486080	L
H-HW-0.417000	0	8.823373	-0.132658	-0.806069	L
O-OW--0.834000	0	12.601765	-1.051548	-1.925285	L
H-HW-0.417000	0	11.752229	-0.807900	-2.331187	L
H-HW-0.417000	0	12.629134	-2.011025	-2.061922	L
O-OW--0.834000	0	8.286108	-3.283765	0.970211	L
H-HW-0.417000	0	8.470329	-2.522004	0.382224	L
H-HW-0.417000	0	7.581508	-2.940774	1.529354	L
O-OW--0.834000	0	4.371124	-4.920661	5.153550	L
H-HW-0.417000	0	4.100604	-4.540860	6.012326	L
H-HW-0.417000	0	4.067919	-4.251970	4.529148	L
S-SH--0.326142	0	-2.358638	-0.685154	-1.666104	H

TS1

C-CT--0.366200	-1	-10.314430	3.913965	4.029183	L
C-C-0.597200	0	-8.979378	3.271212	3.785151	L
O-O--0.567900	0	-8.407284	2.691573	4.700928	L
H-HC-0.112300	0	-11.053192	3.452873	3.376568	L
H-HC-0.112300	0	-10.241302	4.977721	3.810022	L
H-HC-0.112300	0	-10.596118	3.767508	5.069635	L
N-N--0.415700	0	-8.507621	3.375878	2.549192	L
C-CT--0.059700	-1	-7.345984	2.647952	2.030579	L
C-C-0.597300	0	-6.182870	3.609474	1.828245	L
O-O--0.567900	0	-6.292301	4.557745	1.055791	L
C-CT-0.130300	0	-7.717222	1.906184	0.720931	L
C-CT--0.043000	0	-8.884152	0.912674	0.950484	L
C-CT--0.320400	0	-6.496062	1.156473	0.158114	L
C-CT--0.066000	0	-9.455671	0.308530	-0.337726	L
H-H-0.271900	0	-9.048611	3.936825	1.899351	L
H-H1-0.086900	0	-7.038689	1.896069	2.757157	L
H-HC-0.018700	0	-8.033965	2.646400	-0.016234	L
H-HC-0.023600	0	-9.709683	1.424070	1.443991	L
H-HC-0.023600	0	-8.552690	0.105018	1.604331	L
H-HC-0.088200	0	-6.139025	0.415138	0.874110	L
H-HC-0.088200	0	-5.688002	1.854219	-0.063133	L
H-HC-0.088200	0	-6.749393	0.664133	-0.778501	L
H-HC-0.018600	0	-9.710991	1.101418	-1.040140	L
H-HC-0.018600	0	-10.359081	-0.252255	-0.100648	L
H-HC-0.018600	0	-8.734858	-0.370525	-0.790223	L
N-N--0.516300	0	-5.089200	3.360549	2.543540	L

C-CT-0.038100	-1	-3.780305	3.983455	2.394669	L	H-HC 30
C-C-0.536600	0	-3.710519	5.502590	2.134634	L	
O-O--0.581900	0	-2.735828	6.024279	1.590602	L	
C-CT--0.547145	0	-3.027459	3.167600	1.330104	H	
C-C-0.532653	0	-2.495642	1.853459	1.846489	H	
O-O2--0.588507	0	-2.675934	1.447103	2.981035	H	
O-O2--0.625559	0	-1.739750	1.146493	1.003635	H	
H-H-0.293600	0	-5.088235	2.517180	3.106035	L	
H-H1-0.088000	0	-3.248863	3.849021	3.337803	L	
H-HC-0.128435	0	-2.155135	3.741896	0.986313	H	
H-HC-0.183556	0	-3.651195	3.049533	0.438203	H	
N-N--0.415700	0	-4.682606	6.255548	2.631393	L	
C-CT--0.025200	-1	-4.658200	7.701428	2.520369	L	
C-C-0.597300	0	-3.763159	8.330170	3.572831	L	
O-O--0.567900	0	-4.242114	8.963686	4.513771	L	
H-H-0.271900	0	-5.489858	5.761587	2.977599	L	
H-H1-0.069800	0	-5.668730	8.085524	2.652230	L	
H-H1-0.069800	0	-4.307660	7.996682	1.530190	L	
N-N--0.415700	0	-2.458496	8.139562	3.411351	L	
C-CT--0.038900	-1	-1.435887	8.563883	4.361331	L	
C-C-0.597300	0	-0.532351	9.653712	3.774431	L	
O-O--0.567900	0	-0.632620	10.026130	2.603947	L	
C-CT-0.365400	0	-0.572333	7.394751	4.876379	L	
O-OH--0.676100	0	0.505260	7.161170	4.006794	L	
C-CT--0.243800	0	-1.325432	6.085278	5.091786	L	
H-H-0.271900	0	-2.186140	7.564735	2.616922	L	
H-H1-0.100700	0	-1.931671	8.995094	5.229353	L	
H-H1-0.004300	0	-0.152969	7.695616	5.837033	L	
H-HC-0.064200	0	-2.208160	6.264474	5.704198	L	
H-HC-0.064200	0	-0.674247	5.377840	5.599193	L	
H-HC-0.064200	0	-1.621926	5.658246	4.133903	L	
H-HO-0.410200	0	1.047161	6.436612	4.387395	L	
N-N--0.415700	0	0.389932	10.158241	4.598411	L	
C-CT--0.025200	-1	1.430703	11.092346	4.177549	L	
C-C-0.597300	0	2.304720	10.601906	3.030823	L	
O-O--0.567900	0	2.579458	11.356572	2.103115	L	
H-H-0.271900	0	0.441850	9.773917	5.526604	L	
H-H1-0.069800	0	2.082876	11.311956	5.021850	L	
H-H1-0.069800	0	0.960536	12.023263	3.859671	L	
N-N--0.516300	0	2.738650	9.347023	3.109212	L	
C-CT-0.038100	-1	3.637961	8.738124	2.130854	L	
C-C-0.536600	0	2.998885	8.415993	0.792413	L	
O-O--0.581900	0	3.726095	8.089062	-0.139086	L	
C-CT--0.030300	0	4.217225	7.428176	2.695953	L	
C-C-0.799400	-1	5.569010	7.625694	3.358522	L	
O-O2--0.801400	-1	6.148774	8.730188	3.277212	L	
O-O2--0.801400	-1	6.029319	6.672329	4.019153	L	
H-H-0.293600	0	2.422841	8.762532	3.867793	L	
H-H1-0.088000	0	4.441192	9.439470	1.901682	L	
H-HC--0.012200	0	4.361362	6.697447	1.898340	L	
H-HC--0.012200	0	3.516928	6.988221	3.408744	L	
N-N--0.415700	0	1.676792	8.498281	0.679344	L	
C-CT--0.002400	-1	0.968314	8.327304	-0.582559	L	
C-C-0.597300	0	0.398721	9.634180	-1.131130	L	

O-O--0.567900	0	0.357355	9.832017	-2.345455	L
C-CT--0.034300	0	-0.141578	7.276934	-0.427400	L
C-CA-0.011800	0	0.342792	5.867802	-0.130923	L
C-CA--0.125600	0	0.853030	5.056861	-1.162694	L
C-CA--0.125600	0	0.246005	5.347807	1.171954	L
C-CA--0.170400	0	1.256013	3.738105	-0.884021	L
C-CA--0.170400	0	0.648385	4.031597	1.451162	L
C-CA--0.107200	0	1.152241	3.226142	0.419362	L
H-H-0.271900	0	1.157332	8.716952	1.517381	L
H-H1-0.097800	0	1.653355	7.953409	-1.343299	L
H-HC-0.029500	0	-0.711590	7.240575	-1.356552	L
H-HC-0.029500	0	-0.827374	7.597942	0.358304	L
H-HA-0.133000	0	0.925280	5.445153	-2.168129	L
H-HA-0.133000	0	-0.178036	5.952296	1.955527	L
H-HA-0.143000	0	1.637334	3.101719	-1.665717	L
H-HA-0.143000	0	0.539914	3.632614	2.449577	L
H-HA-0.129700	0	1.443770	2.208195	0.626932	L
N-N--0.415700	0	-0.037448	10.535004	-0.249714	L
C-CT--0.149000	-1	-0.481873	11.860628	-0.624157	L
H-H-0.271900	0	-0.012089	10.287963	0.734290	L
H-H1-0.097600	0	-0.844821	12.386587	0.259482	L
H-H1-0.097600	0	-1.284594	11.784815	-1.359514	L
H-H1-0.097600	0	0.351749	12.414240	-1.058963	L
C-CT--0.366200	-1	7.978354	8.445701	-0.148713	L
C-C-0.597200	0	7.512302	7.665655	-1.353955	L
O-O--0.567900	0	8.328386	7.345790	-2.214533	L
H-HC-0.112300	0	7.502809	9.424051	-0.142918	L
H-HC-0.112300	0	9.059554	8.556791	-0.189123	L
H-HC-0.112300	0	7.707038	7.904974	0.758806	L
N-N--0.415700	0	6.204415	7.402607	-1.440728	L
C-CT--0.051800	-1	5.557519	6.869108	-2.648480	L
C-C-0.597300	0	4.856966	7.953346	-3.465262	L
O-O--0.567900	0	5.077224	8.094013	-4.667863	L
C-CT--0.110200	0	4.670511	5.640752	-2.346115	L
C-CT-0.353100	0	4.970467	4.462220	-3.299568	L
C-CT--0.412100	0	4.067913	3.269810	-3.001169	L
C-CT--0.412100	0	4.757819	4.793808	-4.780840	L
H-H-0.271900	0	5.603815	7.695065	-0.677424	L
H-H1-0.092200	0	6.362931	6.518817	-3.294006	L
H-HC-0.045700	0	3.617384	5.915839	-2.417205	L
H-HC-0.045700	0	4.855809	5.304135	-1.325573	L
H-HC--0.036100	0	6.005313	4.148315	-3.156223	L
H-HC-0.100000	0	3.048619	3.492925	-3.311615	L
H-HC-0.100000	0	4.082474	3.049883	-1.934734	L
H-HC-0.100000	0	4.423724	2.393789	-3.541281	L
H-HC-0.100000	0	3.763240	5.215586	-4.928000	L
H-HC-0.100000	0	4.860936	3.894049	-5.384873	L
H-HC-0.100000	0	5.501064	5.515841	-5.115843	L
N-N--0.415700	0	4.033296	8.734564	-2.780984	L
C-CT--0.149000	-1	3.348779	9.898486	-3.275226	L
H-H-0.271900	0	3.933616	8.501845	-1.801041	L
H-H1-0.097600	0	4.023008	10.491568	-3.894434	L
H-H1-0.097600	0	2.998097	10.503985	-2.438194	L
H-H1-0.097600	0	2.490541	9.591083	-3.875181	L

C-CT--0.366200	-1	-8.727677	2.369211	8.136698	L	
C-C-0.597200	0	-7.869507	1.149189	7.957895	L	
O-O--0.567900	0	-7.555852	0.464343	8.924623	L	
H-HC-0.112300	0	-9.646544	2.241537	7.567745	L	
H-HC-0.112300	0	-8.185904	3.240305	7.771516	L	
H-HC-0.112300	0	-8.957280	2.491163	9.193182	L	
N-N--0.415700	0	-7.480693	0.896671	6.716678	L	
C-CT--0.038900	-1	-6.664417	-0.246537	6.303938	L	
C-C-0.597300	0	-5.408643	0.264435	5.620804	L	
O-O--0.567900	0	-5.470340	0.745820	4.489778	L	
C-CT-0.365400	0	-7.457001	-1.220541	5.419298	L	
O-OH--0.676100	0	-8.007983	-0.562263	4.304306	L	
C-CT--0.243800	0	-8.606510	-1.850751	6.210943	L	
H-H-0.271900	0	-7.804026	1.522766	5.983117	L	
H-H1-0.100700	0	-6.355586	-0.805838	7.186807	L	
H-H1-0.004300	0	-6.789322	-2.010717	5.073933	L	
H-HC-0.064200	0	-8.210393	-2.378732	7.078048	L	
H-HC-0.064200	0	-9.150118	-2.553640	5.586441	L	
H-HC-0.064200	0	-9.302591	-1.080726	6.541970	L	
H-HO-0.410200	0	-7.327325	0.047882	3.984402	L	
N-N--0.415700	0	-4.305243	0.226400	6.371876	L	
C-CT--0.059700	-1	-3.111937	1.050777	6.169074	L	
C-C-0.597300	0	-1.864662	0.174379	6.093086	L	
O-O--0.567900	0	-1.606223	-0.627358	6.995820	L	
C-CT-0.130300	0	-2.996620	2.108242	7.292599	L	
C-CT--0.043000	0	-4.251072	3.003614	7.450502	L	
C-CT--0.320400	0	-1.751474	2.984416	7.085938	L	
C-CT--0.066000	0	-4.652310	3.823738	6.215141	L	
H-H-0.271900	0	-4.355917	-0.252031	7.256496	L	
H-H1-0.086900	0	-3.212243	1.588514	5.231152	L	
H-HC-0.018700	0	-2.866364	1.579495	8.238783	L	
H-HC-0.023600	0	-4.079015	3.696805	8.274658	L	
H-HC-0.023600	0	-5.098461	2.382252	7.738751	L	
H-HC-0.088200	0	-1.733387	3.364108	6.064312	L	
H-HC-0.088200	0	-0.853710	2.387615	7.249784	L	
H-HC-0.088200	0	-1.746976	3.814357	7.791403	L	
H-HC-0.018600	0	-3.845414	4.493522	5.922860	L	
H-HC-0.018600	0	-5.534483	4.419418	6.450064	L	
H-HC-0.018600	0	-4.893113	3.165973	5.383196	L	
N-N--0.516300	0	-1.112960	0.351862	5.011224	L	
C-CT-0.038100	-1	0.184829	-0.232951	4.733251	L	H-HC 178
C-C-0.536600	0	1.116088	-0.077808	5.941968	L	
O-O--0.581900	0	1.326611	1.015755	6.462481	L	
C-CT--0.520019	0	0.703850	0.419352	3.438063	H	
C-C-0.636404	0	0.969668	-0.532456	2.282932	H	
O-O2--0.608036	0	0.350499	-1.634992	2.196083	H	
O-O2--0.608131	0	1.822097	-0.100979	1.444190	H	
H-H-0.293600	0	-1.516138	0.931424	4.281314	L	
H-H1-0.088000	0	0.032628	-1.300753	4.568445	L	
H-HC-0.132300	0	1.634146	0.951256	3.649104	H	
H-HC-0.188993	0	-0.005053	1.169725	3.076911	H	
N-N--0.415700	0	1.662456	-1.189211	6.402918	L	
C-CT-0.033700	-1	2.569464	-1.235423	7.526837	L	
C-C-0.597300	0	3.789423	-2.055232	7.122473	L	

O-O--0.567900	0	3.838362	-3.281155	7.272776	L	
C-CT--0.182500	0	1.820365	-1.794647	8.744631	L	
H-H-0.271900	0	1.360230	-2.067372	5.995852	L	
H-H1-0.082300	0	2.914492	-0.230061	7.776128	L	
H-HC-0.060300	0	0.980790	-1.141177	8.985019	L	
H-HC-0.060300	0	2.495833	-1.843998	9.598403	L	
H-HC-0.060300	0	1.444125	-2.794695	8.523141	L	
N-N--0.415700	0	4.759737	-1.358911	6.542944	L	
C-CT-0.018800	-1	5.965534	-1.940352	5.997541	L	H-HC 200
C-C-0.597300	0	7.161492	-1.793349	6.947474	L	
O-O--0.567900	0	7.208447	-0.942030	7.837772	L	
C-CT--0.517456	0	6.222159	-1.299271	4.631458	H	
C-CM-0.287351	0	5.352558	-1.775793	3.526731	H	
N-N2--0.586994	0	5.494959	-3.030246	2.950150	H	
C-CM--0.020338	0	4.374199	-1.167037	2.791223	H	
C-CM-0.242458	0	4.628532	-3.119759	1.905969	H	
N-N2--0.505070	0	3.939892	-2.000593	1.780885	H	
H-H-0.271900	0	4.655611	-0.349471	6.487946	L	
H-H1-0.088100	0	5.819458	-3.006370	5.853960	L	
H-HC-0.166355	0	7.277229	-1.465403	4.368590	H	
H-HC-0.174481	0	6.109632	-0.220744	4.768945	H	
H-H-0.335795	0	6.143131	-3.747522	3.268339	H	
H-H4-0.152777	0	3.974824	-0.171501	2.912935	H	
H-H5-0.209475	0	4.529225	-3.985440	1.265906	H	
N-N--0.415700	0	8.166325	-2.631974	6.701244	L	
C-CT--0.149000	-1	9.436867	-2.558916	7.389561	L	
H-H-0.271900	0	8.015028	-3.310557	5.961644	L	
H-H1-0.097600	0	10.110225	-3.327946	7.009826	L	
H-H1-0.097600	0	9.281471	-2.706047	8.459771	L	
H-H1-0.097600	0	9.882554	-1.575289	7.230560	L	
C-CT--0.366200	-1	8.364488	-9.375785	0.926514	L	
C-C-0.597200	0	8.735466	-9.134628	-0.524200	L	
O-O--0.567900	0	9.755060	-8.507141	-0.783512	L	
H-HC-0.112300	0	8.303028	-10.444124	1.120075	L	
H-HC-0.112300	0	7.417232	-8.886962	1.146673	L	
H-HC-0.112300	0	9.137552	-8.936976	1.557262	L	
N-N--0.254800	0	7.924244	-9.604715	-1.488292	L	
C-CT--0.026600	-1	8.165651	-9.365997	-2.913492	L	
C-C-0.589600	0	8.011369	-7.894039	-3.297096	L	
O-O--0.574800	0	8.717896	-7.397341	-4.171173	L	
C-CT--0.007000	0	7.163901	-10.264639	-3.646413	L	
C-CT-0.018900	0	6.007137	-10.402036	-2.655327	L	
C-CT-0.019200	0	6.702710	-10.374146	-1.293630	L	
H-H1-0.064100	0	9.179614	-9.672596	-3.173580	L	
H-HC-0.025300	0	7.614722	-11.244110	-3.813147	L	
H-HC-0.025300	0	6.837570	-9.833640	-4.594213	L	
H-HC-0.021300	0	5.452599	-11.328706	-2.806162	L	
H-HC-0.021300	0	5.342405	-9.541370	-2.744707	L	
H-H1-0.039100	0	6.051170	-9.912909	-0.550087	L	
H-H1-0.039100	0	6.960685	-11.389730	-0.990372	L	
N-N--0.516300	0	7.090261	-7.198052	-2.630069	L	
C-CT-0.039700	-1	6.838970	-5.770818	-2.800802	L	
C-C-0.536600	0	8.099273	-4.907328	-2.670685	L	
O-O--0.581900	0	8.273091	-3.922300	-3.391955	L	

```
C-CT-0.056000     0     5.742196   -5.334302   -1.825819 L H-HC 244
C-CT--0.515956    0     5.216485   -3.946880   -2.202492 H
C-C-0.587828      0     4.131650   -3.408636   -1.280112 H
O-O2--0.600319    0     4.073907   -2.127372   -1.246748 H
O-O2--0.589127    0     3.382721   -4.198097   -0.659230 H
H-H-0.293600      0     6.561961   -7.674491   -1.918266 L
H-H1-0.110500     0     6.463610   -5.616334   -3.812776 L
H-HC--0.017300    0     6.137032   -5.318090   -0.808782 L
H-HC--0.017300    0     4.917539   -6.047357   -1.875411 L
H-HC-0.167759     0     4.807492   -3.994472   -3.218715 H
H-HC-0.135230     0     6.041334   -3.226150   -2.217644 H
N-N--0.415700     0     9.009949   -5.313721   -1.788457 L
C-CT--0.024900   -1    10.246699   -4.608946   -1.471826 L
C-C-0.597300      0    11.266773   -4.493362   -2.597600 L
O-O--0.567900     0    12.242132   -3.753860   -2.465883 L
C-CT-0.211700     0    10.889817   -5.242807   -0.232317 L
O-OH--0.654600    0     9.934918   -5.430223    0.801840 L
H-H-0.271900      0     8.804214   -6.125976   -1.223094 L
H-H1-0.084300     0     9.976738   -3.586063   -1.204956 L
H-H1-0.035200     0    11.689493   -4.594039    0.128052 L
H-H1-0.035200     0    11.315674   -6.211275   -0.498931 L
H-HO-0.427500     0     9.378426   -4.623091    0.874743 L
N-N--0.415700     0    11.029985   -5.180048   -3.713551 L
C-CT-0.033700    -1    11.779314   -5.015558   -4.952269 L
C-C-0.597300      0    10.954046   -4.440759   -6.107552 L
O-O--0.567900     0    11.395376   -4.432477   -7.255806 L
C-CT--0.182500    0    12.389076   -6.381696   -5.298683 L
H-H-0.271900      0    10.219653   -5.789065   -3.719867 L
H-H1-0.082300     0    12.604145   -4.319552   -4.793499 L
H-HC-0.060300     0    13.002602   -6.733952   -4.468720 L
H-HC-0.060300     0    13.015160   -6.291007   -6.187009 L
H-HC-0.060300     0    11.593971   -7.103283   -5.493271 L
N-N--0.415700     0     9.769983   -3.933159   -5.783477 L
C-CT--0.025200   -1     8.863324   -3.259657   -6.690210 L
C-C-0.597300      0     8.787733   -1.757486   -6.467759 L
O-O--0.567900     0     8.768751   -0.984565   -7.422916 L
H-H-0.271900      0     9.484894   -4.003713   -4.812886 L
H-H1-0.069800     0     7.862112   -3.667823   -6.555889 L
H-H1-0.069800     0     9.162716   -3.434877   -7.724030 L
N-N--0.415700     0     8.743598   -1.351835   -5.202043 L
C-CT--0.002400   -1     8.579304    0.033716   -4.781589 L
C-C-0.597300      0     9.698559    0.466235   -3.827498 L
O-O--0.567900     0    10.321920   -0.385922   -3.186451 L
C-CT--0.034300    0     7.197865    0.164160   -4.114253 L
C-CA-0.011800     0     6.038483   -0.057354   -5.067670 L
C-CA--0.125600    0     5.683994    0.946274   -5.988990 L
C-CA--0.125600    0     5.334981   -1.276287   -5.061316 L
C-CA--0.170400    0     4.638178    0.729804   -6.904336 L
C-CA--0.170400    0     4.287218   -1.491535   -5.974147 L
C-CA--0.107200    0     3.943298   -0.492537   -6.901338 L
H-H-0.271900      0     8.770362   -2.058209   -4.476055 L
H-H1-0.097800     0     8.608270    0.684391   -5.655662 L
H-HC-0.029500     0     7.090189    1.154851   -3.673532 L
H-HC-0.029500     0     7.136287   -0.555103   -3.295802 L
```

H-HA-0.133000	0	6.223742	1.881733	-6.003771	L	
H-HA-0.133000	0	5.605481	-2.052962	-4.361456	L	
H-HA-0.143000	0	4.371816	1.501459	-7.612628	L	
H-HA-0.143000	0	3.745697	-2.426143	-5.964359	L	
H-HA-0.129700	0	3.137935	-0.661533	-7.602512	L	
N-N--0.254800	0	9.954497	1.779893	-3.681649	L	
C-CT--0.026600	-1	10.815649	2.274090	-2.613328	L	
C-C-0.589600	0	10.180773	2.022866	-1.236856	L	
O-O--0.574800	0	8.985961	1.717615	-1.159526	L	
C-CT--0.007000	0	10.973839	3.774268	-2.894130	L	
C-CT-0.018900	0	9.657762	4.142483	-3.576944	L	
C-CT-0.019200	0	9.351143	2.895205	-4.405707	L	
H-H1-0.064100	0	11.786021	1.781607	-2.668331	L	
H-HC-0.025300	0	11.799911	3.929131	-3.589715	L	
H-HC-0.025300	0	11.129474	4.357834	-1.985413	L	
H-HC-0.021300	0	9.756041	5.031871	-4.200161	L	
H-HC-0.021300	0	8.879532	4.286284	-2.824733	L	
H-H1-0.039100	0	8.273236	2.786423	-4.516399	L	
H-H1-0.039100	0	9.819756	2.981992	-5.386837	L	
N-N--0.254800	0	10.946281	2.135954	-0.140904	L	
C-CT--0.026600	-1	10.393185	2.126739	1.206457	L	
C-C-0.589600	0	9.337847	3.209449	1.434399	L	
O-O--0.574800	0	9.593357	4.388687	1.201375	L	
C-CT--0.007000	0	11.592139	2.318027	2.146053	L	
C-CT-0.018900	0	12.780061	1.829528	1.320752	L	
C-CT-0.019200	0	12.396133	2.240176	-0.097925	L	
H-H1-0.064100	0	9.955080	1.144553	1.389858	L	
H-HC-0.025300	0	11.476435	1.742918	3.065285	L	
H-HC-0.025300	0	11.732383	3.375612	2.377175	L	
H-HC-0.021300	0	12.846264	0.741794	1.380125	L	
H-HC-0.021300	0	13.714875	2.292251	1.638086	L	
H-H1-0.039100	0	12.692644	3.274119	-0.279539	L	
H-H1-0.039100	0	12.873351	1.571846	-0.815471	L	
N-N--0.415700	0	8.171093	2.784581	1.909392	L	
C-CT-0.018800	-1	7.015075	3.613582	2.204623	L	H-HC 334
C-C-0.597300	0	6.153944	3.024595	3.318853	L	
O-O--0.567900	0	6.156359	1.813171	3.536266	L	
C-CT--0.515368	0	6.191656	3.733445	0.893923	H	
C-CM-0.287903	0	5.593136	2.456682	0.382063	H	
N-N2--0.582625	0	6.224909	1.661378	-0.560646	H	
C-CM--0.036893	0	4.464095	1.742919	0.713102	H	
C-CM-0.243454	0	5.488633	0.537604	-0.755417	H	
N-N2--0.523998	0	4.417301	0.551222	0.015812	H	
H-H-0.271900	0	8.036616	1.787720	1.999969	L	
H-H1-0.088100	0	7.350681	4.608135	2.513825	L	
H-HC-0.162301	0	6.846085	4.187595	0.135572	H	
H-HC-0.188340	0	5.398584	4.469080	1.076537	H	
H-H-0.339448	0	7.134911	1.862743	-0.974710	H	
H-H4-0.185957	0	3.685536	2.009086	1.413349	H	
H-H5-0.211295	0	5.763079	-0.258567	-1.432148	H	
N-N--0.415700	0	5.392616	3.891015	3.975848	L	
C-CT-0.014300	-1	4.550549	3.597335	5.126395	L	
C-C-0.597300	0	5.203342	2.648399	6.142724	L	
O-O--0.567900	0	4.682103	1.594508	6.511028	L	

C-CT--0.204100	0	3.102049	3.314584	4.702144	L	
C-C-0.713000	0	2.161896	4.197446	5.501551	L	
N-N--0.919100	0	1.574258	3.713672	6.560804	L	
O-O--0.593100	0	1.975961	5.367858	5.216341	L	
H-H-0.271900	0	5.537755	4.879521	3.761686	L	
H-H1-0.104800	0	4.514843	4.550788	5.658456	L	
H-HC-0.079700	0	2.868775	2.266953	4.859107	L	
H-HC-0.079700	0	2.959209	3.550254	3.647870	L	
H-H-0.419600	0	0.915852	4.295213	7.032415	L	
H-H-0.419600	0	1.625208	2.705001	6.705513	L	
N-N--0.415700	0	6.389137	3.081450	6.583642	L	
C-CT--0.149000	-1	7.296197	2.337875	7.436248	L	
H-H-0.271900	0	6.700451	3.956536	6.185421	L	
H-H1-0.097600	0	8.050230	3.002182	7.858051	L	
H-H1-0.097600	0	7.784214	1.553935	6.853969	L	
H-H1-0.097600	0	6.738340	1.859744	8.244591	L	
C-CT--0.366200	-1	-2.248192	-2.739250	9.546015	L	
C-C-0.597200	0	-2.371451	-3.830879	8.518773	L	
O-O--0.567900	0	-2.918016	-4.891539	8.809233	L	
H-HC-0.112300	0	-1.194331	-2.523172	9.710779	L	
H-HC-0.112300	0	-2.708537	-3.070014	10.474616	L	
H-HC-0.112300	0	-2.752393	-1.846645	9.180481	L	
N-N--0.347900	0	-1.837615	-3.572855	7.328410	L	
C-CT--0.240000	-1	-1.740910	-4.536939	6.226419	L	
C-C-0.734100	0	-0.329485	-4.575428	5.651806	L	
O-O--0.589400	0	0.494030	-3.706429	5.924014	L	
C-CT--0.009400	0	-2.830214	-4.220990	5.176800	L	
C-CT-0.018700	0	-2.561250	-2.976538	4.314041	L	H-HC 379
C-CT--0.464524	0	-3.688954	-2.761172	3.293605	H	
C-CT--0.195159	0	-3.488172	-1.614573	2.303598	H	
N-N3--0.748751	0	-2.327613	-1.856512	1.379614	H	
H-H-0.274700	0	-1.441109	-2.645394	7.191622	L	
H-H1-0.142600	0	-1.941016	-5.532617	6.622636	L	
H-HC-0.036200	0	-3.786130	-4.096642	5.688591	L	
H-HC-0.036200	0	-2.934664	-5.073187	4.512682	L	
H-HC-0.010300	0	-1.617054	-3.099410	3.783413	L	
H-HC-0.010300	0	-2.499896	-2.103894	4.954804	L	
H-HC-0.173903	0	-4.618157	-2.576192	3.840543	H	
H-HC-0.148535	0	-3.833997	-3.696074	2.731455	H	
H-HP-0.236066	0	-3.332629	-0.645062	2.775253	H	
H-HP-0.172509	0	-4.366693	-1.550917	1.649746	H	
H-H-0.437293	0	-2.416518	-1.304910	0.464299	H	
H-H-0.366736	0	-2.360607	-2.841836	1.088092	H	
H-H-0.419451	0	-1.390086	-1.667063	1.799002	H	
N-N--0.415700	0	-0.037494	-5.600416	4.864350	L	
C-CT--0.025200	-1	1.281258	-5.802043	4.272837	L	
C-C-0.597300	0	1.162899	-6.543826	2.939266	L	
O-O--0.567900	0	1.728233	-7.615405	2.733131	L	
H-H-0.271900	0	-0.784490	-6.244206	4.622639	L	
H-H1-0.069800	0	1.902455	-6.388899	4.949594	L	
H-H1-0.069800	0	1.768286	-4.843525	4.089703	L	
N-N--0.415700	0	0.369162	-5.954188	2.051645	L	
C-CT--0.025200	-1	-0.228601	-6.534940	0.851923	L	
C-C-0.597300	0	-1.312086	-5.567529	0.416788	L	

O-O--0.567900	0	-2.092719	-5.113066	1.248914	L
H-H-0.271900	0	-0.058942	-5.096088	2.374851	L
H-H1-0.069800	0	-0.673548	-7.501742	1.086765	L
H-H1-0.069800	0	0.528582	-6.642355	0.074915	L
N-N--0.415700	0	-1.339640	-5.210692	-0.863268	L
C-CT--0.001400	-1	-2.156369	-4.103294	-1.360771	L
C-C-0.597300	0	-3.631880	-4.188767	-0.948028	L
O-O--0.567900	0	-4.233326	-3.214521	-0.512872	L
C-CT--0.015200	0	-2.046890	-4.044528	-2.898074	L
C-CA--0.001100	0	-0.657742	-4.250027	-3.476828	L
C-CA--0.190600	0	0.369902	-3.327341	-3.203784	L
C-CA--0.190600	0	-0.398253	-5.366282	-4.297626	L
C-CA--0.234100	0	1.655446	-3.522610	-3.743688	L
C-CA--0.234100	0	0.885495	-5.564016	-4.840674	L
C-C-0.322600	0	1.916548	-4.640188	-4.562420	L
O-OH--0.557900	0	3.151530	-4.829252	-5.098219	L
H-H-0.271900	0	-0.594691	-5.528253	-1.466475	L
H-H1-0.087600	0	-1.740277	-3.183313	-0.957347	L
H-HC-0.029500	0	-2.409431	-3.077304	-3.238618	L
H-HC-0.029500	0	-2.711116	-4.795855	-3.326377	L
H-HA-0.169900	0	0.178456	-2.460777	-2.581084	L
H-HA-0.169900	0	-1.191877	-6.061754	-4.529297	L
H-HA-0.165600	0	2.443649	-2.816779	-3.529514	L
H-HA-0.165600	0	1.076607	-6.412175	-5.477672	L
H-HO-0.399200	0	3.165044	-5.593452	-5.672275	L
N-N--0.415700	0	-4.210570	-5.383186	-1.067177	L
C-CT--0.024900	-1	-5.555779	-5.648001	-0.576858	L
C-C-0.597300	0	-5.675058	-5.543580	0.940953	L
O-O--0.567900	0	-4.989490	-6.249532	1.689823	L
C-CT-0.211700	0	-6.012753	-7.049174	-0.986437	L
O-OH--0.654600	-1	-7.274872	-7.349388	-0.413657	L
H-H-0.271900	0	-3.626850	-6.137376	-1.381526	L
H-H1-0.084300	0	-6.236817	-4.927902	-1.031290	L
H-H1-0.035200	0	-5.283494	-7.781297	-0.637623	L
H-H1-0.035200	0	-6.077617	-7.105558	-2.073222	L
H-HO-0.427500	-1	-7.531849	-8.222209	-0.720970	L
N-N--0.415700	0	-6.668907	-4.766541	1.365566	L
C-CT-0.033700	-1	-7.153581	-4.672750	2.731221	L
C-C-0.597300	0	-7.451245	-6.015097	3.405470	L
O-O--0.567900	0	-7.253315	-6.162853	4.609181	L
C-CT--0.182500	0	-8.382527	-3.763250	2.737819	L
H-H-0.271900	0	-7.153407	-4.211664	0.666373	L
H-H1-0.082300	0	-6.377987	-4.190101	3.326898	L
H-HC-0.060300	0	-8.131963	-2.786018	2.320813	L
H-HC-0.060300	0	-8.730038	-3.636881	3.758605	L
H-HC-0.060300	0	-9.180789	-4.217422	2.156355	L
N-N--0.415700	0	-7.935804	-6.991023	2.636461	L
C-CT--0.025200	-1	-8.276315	-8.318858	3.134519	L
C-C-0.597300	0	-7.324040	-9.412419	2.659870	L
O-O--0.567900	0	-7.756439	-10.524786	2.367664	L
H-H-0.271900	0	-8.007403	-6.796406	1.644632	L
H-H1-0.069800	0	-9.278132	-8.573372	2.789574	L
H-H1-0.069800	0	-8.288356	-8.331416	4.224722	L
N-N--0.415700	0	-6.024008	-9.120500	2.639405	L

C-CT--0.024900	-1	-4.958774	-10.125458	2.612023	L	
C-C-0.597300	0	-4.231126	-10.092667	3.950649	L	
O-O--0.567900	0	-3.900538	-9.017523	4.460584	L	
C-CT-0.211700	0	-3.992066	-9.848616	1.456930	L	
O-OH--0.654600	-1	-3.079693	-10.924915	1.294092	L	
H-H-0.271900	0	-5.765877	-8.166493	2.869137	L	
H-H1-0.084300	0	-5.386725	-11.116832	2.462629	L	
H-H1-0.035200	0	-3.446461	-8.922364	1.645750	L	
H-H1-0.035200	0	-4.567323	-9.735371	0.537232	L	
H-HO-0.427500	-1	-2.539365	-10.736305	0.523299	L	
N-N--0.415700	0	-4.037695	-11.268695	4.547238	L	
C-CT--0.149000	-1	-3.520988	-11.409919	5.898288	L	
H-H-0.271900	0	-4.319002	-12.089629	4.035133	L	
H-H1-0.097600	0	-3.300345	-12.455696	6.112513	L	
H-H1-0.097600	0	-4.255513	-11.041439	6.617786	L	
H-H1-0.097600	0	-2.606776	-10.822123	6.011027	L	
C-CT--0.366200	-1	-12.145036	5.078349	-0.643971	L	
C-C-0.597200	0	-10.716096	5.258676	-0.203707	L	
O-O--0.567900	0	-10.398563	4.986476	0.948651	L	
H-HC-0.112300	0	-12.538432	6.032196	-0.988594	L	
H-HC-0.112300	0	-12.731279	4.724295	0.202453	L	
H-HC-0.112300	0	-12.180272	4.340238	-1.443165	L	
N-N--0.415700	0	-9.867146	5.706537	-1.124081	L	
C-CT--0.025200	-1	-8.433918	5.871901	-0.897265	L	
C-C-0.597300	0	-7.619452	5.743782	-2.160546	L	
O-O--0.567900	0	-8.127450	6.034625	-3.249823	L	
H-H-0.271900	0	-10.181949	5.870583	-2.068094	L	
H-H1-0.069800	0	-8.120064	5.093394	-0.212090	L	
H-H1-0.069800	0	-8.233616	6.842779	-0.446310	L	
N-N--0.415700	0	-6.360930	5.333095	-1.985551	L	H-H1 489
C-CT--0.530209	-1	-5.349486	5.299088	-3.042549	H	
C-C-0.568093	0	-4.526347	4.034559	-2.924880	H	
O-O--0.528105	0	-4.334470	3.502378	-1.817056	H	
C-CT-0.298500	0	-4.463181	6.568521	-3.004824	L	H-H1 489
C-CT--0.319200	0	-5.266993	7.812665	-3.387742	L	
C-CT--0.319200	0	-3.798121	6.799487	-1.644231	L	
H-H-0.271900	0	-6.065798	5.059935	-1.049655	L	
H-H1-0.184096	0	-5.876598	5.311870	-4.002832	H	
H-HC--0.029700	0	-3.674268	6.457205	-3.747781	L	
H-HC-0.079100	0	-6.012962	8.026505	-2.624090	L	
H-HC-0.079100	0	-5.765897	7.645102	-4.341216	L	
H-HC-0.079100	0	-4.594215	8.664357	-3.479359	L	
H-HC-0.079100	0	-4.548629	6.941444	-0.868143	L	
H-HC-0.079100	0	-3.159453	7.680053	-1.689765	L	
H-HC-0.079100	0	-3.184032	5.938691	-1.379393	L	
N-N--0.610103	0	-3.979682	3.558824	-4.057733	H	
C-CT--0.314060	-1	-4.236772	4.007648	-5.419350	H	
C-C-0.597300	0	-5.356194	3.189004	-6.046860	L	H-H1 505
O-O--0.567900	0	-5.488196	1.987150	-5.817410	L	
C-CT--0.110200	0	-2.941112	3.883469	-6.257314	L	H-H1 505
C-CT-0.353100	0	-1.874592	4.970114	-5.979928	L	
C-CT--0.412100	0	-1.078821	4.766291	-4.687216	L	
C-CT--0.412100	0	-0.862105	4.997914	-7.128666	L	
H-H-0.398207	0	-3.436617	2.697036	-3.951374	H	

```
H-H1-0.165130    0   -4.531922    5.056138   -5.426930 H
H-HC-0.045700    0   -3.225171    3.962602   -7.306982 L
H-HC-0.045700    0   -2.501794    2.895965   -6.114840 L
H-HC--0.036100   0   -2.362385    5.944750   -5.944562 L
H-HC-0.100000    0   -0.622119    3.778512   -4.682785 L
H-HC-0.100000    0   -1.741396    4.854944   -3.828032 L
H-HC-0.100000    0   -0.307474    5.530097   -4.597793 L
H-HC-0.100000    0   -0.344124    4.042030   -7.189111 L
H-HC-0.100000    0   -0.134190    5.790595   -6.955771 L
H-HC-0.100000    0   -1.374932    5.194325   -8.068873 L
N-N--0.415700    0   -6.141034    3.835787   -6.904142 L
C-CT--0.038900  -1   -7.328026    3.237261   -7.527598 L
C-C-0.597300     0   -7.077114    1.899136   -8.211368 L
O-O--0.567900    0   -7.947572    1.031896   -8.232641 L
C-CT-0.365400   -1   -7.966094    4.161654   -8.588887 L
O-OH--0.676100  -1   -7.905178    5.518738   -8.140490 L
C-CT--0.243800   0   -9.443305    3.866596   -8.847328 L
H-H-0.271900     0   -6.016078    4.832601   -7.002328 L
H-H1-0.100700    0   -8.069143    3.095758   -6.747735 L
H-H1-0.004300    0   -7.410854    4.085933   -9.524796 L
H-HC-0.064200    0   -9.555699    2.860293   -9.250767 L
H-HC-0.064200    0   -9.838817    4.576032   -9.574087 L
H-HC-0.064200    0  -10.008143    3.940753   -7.917241 L
H-HO-0.410200   -1   -8.803585    5.853362   -8.104729 L
N-N--0.415700    0   -5.906159    1.770436   -8.832515 L
C-CT--0.025200  -1   -5.519675    0.609863   -9.622406 L
C-C-0.597300     0   -4.590619   -0.371039   -8.939714 L
O-O--0.567900    0   -4.066525   -1.252767   -9.620289 L
H-H-0.271900     0   -5.228613    2.499872   -8.671731 L
H-H1-0.069800    0   -5.018340    0.954802  -10.525628 L
H-H1-0.069800    0   -6.406612    0.056419   -9.931023 L
N-N--0.415700    0   -4.384529   -0.201556   -7.629337 L H-H1 545
C-CT--0.539097  -1   -3.474835   -1.000876   -6.819310 H
C-C-0.572106     0   -4.241436   -1.432543   -5.568086 H
O-O--0.533001    0   -5.022184   -2.377165   -5.678584 H
C-CT-0.130300    0   -2.102769   -0.300348   -6.627999 L H-H1 545
C-CT--0.043000   0   -1.472767    0.157219   -7.965284 L
C-CT--0.320400   0   -1.181760   -1.306444   -5.915847 L
C-CT--0.066000   0   -0.123738    0.874812   -7.824444 L
H-H-0.271900     0   -4.874496    0.551372   -7.155300 L
H-H1-0.180646    0   -3.287458   -1.928860   -7.370537 H
H-HC-0.018700    0   -2.235153    0.577015   -5.999119 L
H-HC-0.023600    0   -2.148052    0.861336   -8.449899 L
H-HC-0.023600    0   -1.349795   -0.704658   -8.622361 L
H-HC-0.088200    0   -1.061542   -2.201872   -6.524528 L
H-HC-0.088200    0   -1.600891   -1.586440   -4.951442 L
H-HC-0.088200    0   -0.205053   -0.872136   -5.722371 L
H-HC-0.018600    0   -0.210368    1.683293   -7.099900 L
H-HC-0.018600    0    0.163614    1.292284   -8.789522 L
H-HC-0.018600    0    0.650605    0.177921   -7.506076 L
N-N--0.611145    0   -4.075776   -0.699828   -4.439643 H
C-CT--0.109296  -1   -4.638608   -1.104405   -3.157056 H
C-C-0.597300     0   -6.166034   -1.109288   -3.155273 L H-H1 564
O-O--0.567900    0   -6.800160   -2.026087   -2.621139 L
```

C-CT--0.388038	0	-4.075248	-0.289104	-1.999273	H
H-H-0.369964	0	-3.325793	-0.018395	-4.373129	H
H-H1-0.178541	0	-4.383390	-2.152223	-2.969159	H
H-H1-0.157964	0	-4.684815	-0.549999	-1.125335	H
H-H1-0.179833	0	-4.218138	0.782368	-2.176767	H
H-HS-0.319500	0	-1.744959	1.521644	0.062925	H
N-N--0.415700	0	-6.755850	-0.122790	-3.818152	L
C-CT--0.087500	-1	-8.196056	-0.066342	-4.052095	L
C-C-0.597300	0	-8.680346	-1.218185	-4.919194	L
O-O--0.567900	0	-9.613931	-1.923064	-4.537654	L
C-CT-0.298500	0	-8.606550	1.296107	-4.628852	L
C-CT--0.319200	0	-10.124662	1.414319	-4.819900	L
C-CT--0.319200	0	-8.166390	2.465283	-3.733505	L
H-H-0.271900	0	-6.122461	0.542833	-4.254054	L
H-H1-0.096900	0	-8.692911	-0.172597	-3.089781	L
H-HC--0.029700	0	-8.130738	1.394771	-5.595813	L
H-HC-0.079100	0	-10.631806	1.254355	-3.868083	L
H-HC-0.079100	0	-10.471028	0.675352	-5.542313	L
H-HC-0.079100	0	-10.371641	2.405249	-5.199926	L
H-HC-0.079100	0	-8.615768	2.380088	-2.745547	L
H-HC-0.079100	0	-8.459307	3.413994	-4.182331	L
H-HC-0.079100	0	-7.081566	2.476588	-3.621355	L
N-N--0.415700	0	-8.051933	-1.422593	-6.075137	L
C-CT--0.025200	-1	-8.405284	-2.482628	-7.015409	L
C-C-0.597300	0	-8.321549	-3.883731	-6.422404	L
O-O--0.567900	0	-9.222826	-4.696904	-6.613614	L
H-H-0.271900	0	-7.260649	-0.827383	-6.283641	L
H-H1-0.069800	0	-7.732672	-2.434362	-7.871495	L
H-H1-0.069800	0	-9.423614	-2.320576	-7.368317	L
N-N--0.415700	0	-7.238517	-4.160183	-5.699304	L
C-CT--0.025200	-1	-7.009054	-5.424952	-5.012480	L
C-C-0.597300	0	-7.928598	-5.685439	-3.830259	L
O-O--0.567900	0	-8.242242	-6.838886	-3.542715	L
H-H-0.271900	0	-6.526698	-3.434612	-5.636501	L
H-H1-0.069800	0	-5.981858	-5.444411	-4.650325	L
H-H1-0.069800	0	-7.136045	-6.241781	-5.723200	L
N-N--0.415700	0	-8.329178	-4.626236	-3.130394	L
C-CT--0.038900	-1	-9.379183	-4.689362	-2.111199	L
C-C-0.597300	0	-10.749078	-4.983934	-2.713194	L
O-O--0.567900	0	-11.491991	-5.817795	-2.195690	L
C-CT-0.365400	0	-9.421445	-3.388692	-1.294760	L
O-OH--0.676100	0	-8.185148	-3.216809	-0.640411	L
C-CT--0.243800	0	-10.506556	-3.404830	-0.218609	L
H-H-0.271900	0	-7.960922	-3.718770	-3.392491	L
H-H1-0.100700	0	-9.149604	-5.505084	-1.425692	L
H-H1-0.004300	0	-9.592318	-2.537560	-1.953998	L
H-HC-0.064200	0	-11.487006	-3.293105	-0.680413	L
H-HC-0.064200	0	-10.349741	-2.579882	0.475836	L
H-HC-0.064200	0	-10.475587	-4.348401	0.324145	L
H-HO-0.410200	0	-7.598757	-2.754380	-1.273804	L
N-N--0.415700	0	-11.098769	-4.278467	-3.789993	L
C-CT--0.149000	-1	-12.332347	-4.468360	-4.524569	L
H-H-0.271900	0	-10.432091	-3.596404	-4.140303	L
H-H1-0.097600	0	-12.405587	-3.719673	-5.314316	L

H-H1-0.097600	0	-13.183840	-4.375803	-3.848996	L
H-H1-0.097600	0	-12.335508	-5.463503	-4.972547	L
N-N2--0.511415	0	1.961733	-0.054695	-1.683948	H
C-CA-0.057801	0	0.711722	0.410016	-1.598988	H
C-CA-0.063496	0	0.028290	0.950386	-2.691955	H
C-CA--0.109122	0	0.698941	1.028651	-3.911639	H
C-CA--0.164490	0	2.008331	0.566567	-3.997011	H
C-CA-0.098392	0	2.600241	0.022741	-2.865528	H
C-C-0.541263	0	-1.374947	1.472210	-2.621187	H
O-O--0.574884	0	-2.035668	1.573264	-3.676027	H
N-N--0.751108	0	-1.636377	2.324674	-1.493989	H
H-H4-0.220889	0	0.230893	0.327411	-0.630840	H
H-HA-0.160434	0	0.179761	1.443348	-4.767577	H
H-HA-0.142191	0	2.566106	0.629538	-4.924399	H
H-H4-0.174502	0	3.605487	-0.382473	-2.888168	H
H-H-0.412468	0	-2.546409	2.787513	-1.621511	H
H-H-0.353801	0	-0.903677	3.032197	-1.379894	H
Zn-ZN-0.974537	0	2.846066	-1.098356	-0.004960	H
O-OW--0.834000	0	-2.165845	-7.198519	3.447840	L
H-HW-0.417000	0	-2.683710	-6.592611	2.898798	L
H-HW-0.417000	0	-2.833934	-7.793349	3.828054	L
O-OW--0.783903	0	1.335492	-2.584302	-0.101512	H
H-HW-0.401253	0	0.954490	-2.473898	0.805072	H
H-HW-0.424111	0	1.947116	-3.362906	-0.148588	H
O-OW--0.834000	0	-3.746455	-8.447514	7.223847	L
H-HW-0.417000	0	-3.798141	-7.504056	7.397632	L
H-HW-0.417000	0	-3.825012	-8.514228	6.260753	L
O-OW--0.834000	0	7.179897	-0.002388	1.808088	L
H-HW-0.417000	0	6.874392	0.396359	2.642680	L
H-HW-0.417000	0	6.389625	0.090960	1.253416	L
O-OW--0.834000	0	7.052788	-4.939001	4.758910	L
H-HW-0.417000	0	6.103276	-5.004882	4.988011	L
H-HW-0.417000	0	7.270584	-5.824879	4.466263	L
O-OW--0.834000	0	8.572073	-0.917517	-0.293965	L
H-HW-0.417000	0	8.138202	-0.541692	0.498462	L
H-HW-0.417000	0	8.819657	-0.126746	-0.794223	L
O-OW--0.834000	0	12.617523	-1.056009	-1.928499	L
H-HW-0.417000	0	11.772132	-0.810720	-2.342041	L
H-HW-0.417000	0	12.640110	-2.016694	-2.057384	L
O-OW--0.834000	0	8.279686	-3.281766	0.976639	L
H-HW-0.417000	0	8.464651	-2.519876	0.389181	L
H-HW-0.417000	0	7.571713	-2.940424	1.532555	L
O-OW--0.834000	0	4.360918	-4.895684	5.157387	L
H-HW-0.417000	0	4.090884	-4.519594	6.018044	L
H-HW-0.417000	0	4.062870	-4.220894	4.536905	L
S-SH--0.326142	0	-2.316004	-0.668758	-1.629003	H

IC1

C-CT--0.3662	-1	-10.308751	3.911350	4.037712	L
C-C-0.5972	0	-8.971022	3.274773	3.790699	L
O-O--0.5679	0	-8.389898	2.705350	4.707656	L
H-HC-0.1123	0	-11.047636	3.444138	3.389597	L
H-HC-0.1123	0	-10.242046	4.974727	3.814714	L
H-HC-0.1123	0	-10.585672	3.766925	5.079743	L

N-N--0.415700	0	-8.506154	3.372834	2.551216	L		
C-CT--0.059700	-1	-7.340831	2.648320	2.036438	L		
C-C-0.597300	0	-6.177708	3.609564	1.832369	L		
O-O--0.567900	0	-6.286977	4.561532	1.064687	L		
C-CT-0.130300	0	-7.698452	1.897338	0.728007	L		
C-CT--0.043000	0	-8.875375	0.913451	0.947038	L		
C-CT--0.320400	0	-6.471776	1.133557	0.193151	L		
C-CT--0.066000	0	-9.432933	0.307106	-0.346095	L		
H-H-0.271900	0	-9.052086	3.927284	1.899903	L		
H-H1-0.086900	0	-7.033799	1.900334	2.767353	L		
H-HC-0.018700	0	-7.998183	2.633253	-0.020536	L		
H-HC-0.023600	0	-9.704295	1.433064	1.426047	L		
H-HC-0.023600	0	-8.558741	0.106899	1.609489	L		
H-HC-0.088200	0	-6.135472	0.393250	0.920052	L		
H-HC-0.088200	0	-5.652732	1.822388	-0.015692	L		
H-HC-0.088200	0	-6.710087	0.636110	-0.744785	L		
H-HC-0.018600	0	-9.679863	1.098917	-1.052578	L		
H-HC-0.018600	0	-10.339493	-0.252290	-0.118010	L		
H-HC-0.018600	0	-8.707806	-0.373146	-0.789692	L		
N-N--0.516300	0	-5.081966	3.352915	2.540473	L		
C-CT-0.038100	-1	-3.773985	3.979262	2.405716	L	H-HC	30
C-C-0.536600	0	-3.699826	5.499217	2.151874	L		
O-O--0.581900	0	-2.719252	6.020476	1.617158	L		
C-CT--0.547145	0	-2.999313	3.173395	1.356369	H		
C-C-0.532653	0	-2.525839	1.808790	1.827711	H		
O-O2--0.588507	0	-2.705491	1.429381	2.989004	H		
O-O2--0.625559	0	-1.901748	1.104073	0.929917	H		
H-H-0.293600	0	-5.080720	2.505547	3.096126	L		
H-H1-0.088000	0	-3.254948	3.842550	3.355280	L		
H-HC-0.128435	0	-2.096351	3.734054	1.070455	H		
H-HC-0.183556	0	-3.597423	3.108525	0.442386	H		
N-N--0.415700	0	-4.674776	6.251330	2.644422	L		
C-CT--0.025200	-1	-4.648296	7.697706	2.542035	L		
C-C-0.597300	0	-3.753738	8.319769	3.598989	L		
O-O--0.567900	0	-4.233146	8.945822	4.544633	L		
H-H-0.271900	0	-5.485546	5.757201	2.982136	L		
H-H1-0.069800	0	-5.658478	8.082520	2.674545	L		
H-H1-0.069800	0	-4.295726	7.997681	1.554006	L		
N-N--0.415700	0	-2.448928	8.132453	3.435364	L		
C-CT--0.038900	-1	-1.425795	8.551597	4.386661	L		
C-C-0.597300	0	-0.520461	9.641741	3.803341	L		
O-O--0.567900	0	-0.619441	10.017237	2.633743	L		
C-CT-0.365400	0	-0.564411	7.379344	4.896788	L		
O-OH--0.676100	0	0.507656	7.142624	4.021509	L		
C-CT--0.243800	0	-1.321174	6.072412	5.114250	L		
H-H-0.271900	0	-2.175793	7.561701	2.638197	L		
H-H1-0.100700	0	-1.920732	8.980594	5.256267	L		
H-H1-0.004300	0	-0.139419	7.677775	5.855729	L		
H-HC-0.064200	0	-2.202516	6.254057	5.728060	L		
H-HC-0.064200	0	-0.670814	5.363691	5.620954	L		
H-HC-0.064200	0	-1.620117	5.645985	4.156837	L		
H-HO-0.410200	0	1.049088	6.417114	4.400987	L		
N-N--0.415700	0	0.401746	10.143189	4.629298	L		
C-CT--0.025200	-1	1.443325	11.077789	4.211327	L		

C-C-0.597300	0	2.316388	10.589631	3.062827	L
O-O--0.567900	0	2.590158	11.345888	2.136174	L
H-H-0.271900	0	0.451898	9.757725	5.557111	L
H-H1-0.069800	0	2.095927	11.294128	5.056146	L
H-H1-0.069800	0	0.974020	12.010132	3.896348	L
N-N--0.516300	0	2.750290	9.334537	3.138511	L
C-CT-0.038100	-1	3.649010	8.727443	2.158494	L
C-C-0.536600	0	3.010261	8.410137	0.818787	L
O-O--0.581900	0	3.738223	8.088866	-0.113932	L
C-CT--0.030300	0	4.226860	7.415231	2.719732	L
C-C-0.799400	-1	5.578538	7.609521	3.383565	L
O-O2--0.801400	-1	6.159407	8.713682	3.305708	L
O-O2--0.801400	-1	6.037681	6.653769	4.041552	L
H-H-0.293600	0	2.436338	8.749414	3.897233	L
H-H1-0.088000	0	4.453050	9.428650	1.931685	L
H-HC--0.012200	0	4.370755	6.687043	1.919741	L
H-HC--0.012200	0	3.525812	6.973582	3.430774	L
N-N--0.415700	0	1.687939	8.489584	0.706293	L
C-CT--0.002400	-1	0.979931	8.327208	-0.557055	L
C-C-0.597300	0	0.415984	9.638356	-1.101031	L
O-O--0.567900	0	0.374638	9.840146	-2.314716	L
C-CT--0.034300	0	-0.137187	7.282766	-0.405969	L
C-CA-0.011800	0	0.334744	5.859748	-0.156818	L
C-CA--0.125600	0	0.771038	5.056768	-1.228097	L
C-CA--0.125600	0	0.294344	5.317058	1.140016	L
C-CA--0.170400	0	1.157608	3.724016	-0.996205	L
C-CA--0.170400	0	0.689056	3.989000	1.374354	L
C-CA--0.107200	0	1.119995	3.191750	0.303035	L
H-H-0.271900	0	1.167971	8.704286	1.545049	L
H-H1-0.097800	0	1.663227	7.952373	-1.319055	L
H-HC-0.029500	0	-0.724419	7.276288	-1.324845	L
H-HC-0.029500	0	-0.805545	7.590035	0.399988	L
H-HA-0.133000	0	0.798091	5.462168	-2.228757	L
H-HA-0.133000	0	-0.079777	5.914113	1.954286	L
H-HA-0.143000	0	1.481173	3.096471	-1.810907	L
H-HA-0.143000	0	0.628951	3.574363	2.370377	L
H-HA-0.129700	0	1.408112	2.165884	0.477759	L
N-N--0.4157	0	-0.019036	10.536923	-0.216919	L
C-CT--0.1490	-1	-0.466795	11.862054	-0.588775	L
H-H-0.2719	0	0.006104	10.287241	0.766418	L
H-H1-0.0976	0	-0.826950	12.386997	0.296608	L
H-H1-0.0976	0	-1.272304	11.785606	-1.321004	L
H-H1-0.0976	0	0.364311	12.417117	-1.026555	L
C-CT--0.3662	-1	7.989928	8.437483	-0.120393	L
C-C-0.5972	0	7.524050	7.665785	-1.330860	L
O-O--0.5679	0	8.339585	7.351650	-2.194011	L
H-HC-0.1123	0	7.507355	9.412284	-0.102731	L
H-HC-0.1123	0	9.070078	8.556947	-0.164684	L
H-HC-0.1123	0	7.726710	7.885974	0.782981	L
N-N--0.415700	0	6.216441	7.402683	-1.417937	L
C-CT--0.051800	-1	5.568444	6.870615	-2.625639	L
C-C-0.597300	0	4.867088	7.955279	-3.440963	L
O-O--0.567900	0	5.075995	8.088380	-4.646428	L
C-CT--0.110200	0	4.677120	5.647298	-2.319674	L

C-CT-0.353100	0	4.989222	4.460074	-3.256567	L
C-CT--0.412100	0	4.077611	3.275996	-2.957251	L
C-CT--0.412100	0	4.797719	4.776612	-4.744309	L
H-H-0.271900	0	5.616616	7.690934	-0.652472	L
H-H1-0.092200	0	6.372823	6.518859	-3.271497	L
H-HC-0.045700	0	3.624888	5.921505	-2.407891	L
H-HC-0.045700	0	4.848619	5.321997	-1.293021	L
H-HC--0.036100	0	6.020910	4.144781	-3.094687	L
H-HC-0.100000	0	3.065977	3.498402	-3.291312	L
H-HC-0.100000	0	4.066867	3.073125	-1.887394	L
H-HC-0.100000	0	4.443799	2.392262	-3.477107	L
H-HC-0.100000	0	3.805748	5.197829	-4.909553	L
H-HC-0.100000	0	4.907834	3.870311	-5.337081	L
H-HC-0.100000	0	5.545736	5.494736	-5.077122	L
N-N--0.4157	0	4.053920	8.743426	-2.751945	L
C-CT--0.1490	-1	3.362881	9.903977	-3.244251	L
H-H-0.2719	0	3.961244	8.513342	-1.770736	L
H-H1-0.0976	0	4.032498	10.499106	-3.866449	L
H-H1-0.0976	0	3.012858	10.508712	-2.406405	L
H-H1-0.0976	0	2.503826	9.592619	-3.840990	L
C-CT--0.3662	-1	-8.724966	2.352976	8.141225	L
C-C-0.5972	0	-7.853008	1.143255	7.959305	L
O-O--0.5679	0	-7.526447	0.463760	8.925499	L
H-HC-0.1123	0	-9.649399	2.209004	7.585371	L
H-HC-0.1123	0	-8.199877	3.228819	7.763209	L
H-HC-0.1123	0	-8.942454	2.479035	9.199809	L
N-N--0.415700	0	-7.468786	0.891310	6.716289	L
C-CT--0.038900	-1	-6.663608	-0.259387	6.301507	L
C-C-0.597300	0	-5.400752	0.234964	5.618518	L
O-O--0.567900	0	-5.447145	0.685296	4.473817	L
C-CT-0.365400	0	-7.467916	-1.224154	5.417021	L
O-OH--0.676100	0	-8.015127	-0.556157	4.305821	L
C-CT--0.243800	0	-8.621668	-1.845645	6.209557	L
H-H-0.271900	0	-7.800651	1.513528	5.983184	L
H-H1-0.100700	0	-6.360782	-0.823901	7.183172	L
H-H1-0.004300	0	-6.808566	-2.019527	5.067412	L
H-HC-0.064200	0	-8.228618	-2.379517	7.074452	L
H-HC-0.064200	0	-9.172971	-2.542658	5.584701	L
H-HC-0.064200	0	-9.310107	-1.070223	6.543855	L
H-HO-0.410200	0	-7.322262	0.033355	3.974655	L
N-N--0.415700	0	-4.304567	0.213662	6.380607	L
C-CT--0.059700	-1	-3.109816	1.034847	6.171710	L
C-C-0.597300	0	-1.862416	0.157780	6.097918	L
O-O--0.567900	0	-1.604606	-0.647087	6.997294	L
C-CT-0.130300	0	-2.991482	2.096950	7.290314	L
C-CT--0.043000	0	-4.245793	2.992824	7.446543	L
C-CT--0.320400	0	-1.746983	2.972111	7.075454	L
C-CT--0.066000	0	-4.656750	3.797929	6.204433	L
H-H-0.271900	0	-4.368170	-0.239916	7.277637	L
H-H1-0.086900	0	-3.212032	1.570835	5.233706	L
H-HC-0.018700	0	-2.858480	1.572771	8.238668	L
H-HC-0.023600	0	-4.069155	3.695559	8.261603	L
H-HC-0.023600	0	-5.090370	2.373631	7.747523	L
H-HC-0.088200	0	-1.732768	3.348040	6.052398	L

H-HC-0.088200	0	-0.848191	2.376474	7.238348	L		
H-HC-0.088200	0	-1.739968	3.804929	7.777550	L		
H-HC-0.018600	0	-3.850383	4.460612	5.894583	L		
H-HC-0.018600	0	-5.534112	4.400208	6.440584	L		
H-HC-0.018600	0	-4.909483	3.129686	5.384219	L		
N-N--0.516300	0	-1.110396	0.338912	5.017771	L		
C-CT-0.038100	-1	0.186208	-0.247869	4.733290	L	H-HC	178
C-C-0.536600	0	1.127652	-0.092891	5.933674	L		
O-O--0.581900	0	1.343292	1.000301	6.451692	L		
C-CT--0.520019	0	0.678734	0.398377	3.423626	H		
C-C-0.636404	0	0.987412	-0.566085	2.290678	H		
O-O2--0.608036	0	0.391125	-1.681186	2.209235	H		
O-O2--0.608131	0	1.832538	-0.121655	1.447764	H		
H-H-0.293600	0	-1.511165	0.934114	4.299710	L		
H-H1-0.088000	0	0.028991	-1.315763	4.574443	L		
H-HC-0.132300	0	1.573270	0.991909	3.623870	H		
H-HC-0.188993	0	-0.074898	1.091584	3.035415	H		
N-N--0.415700	0	1.668274	-1.206570	6.395964	L		
C-CT-0.033700	-1	2.568870	-1.260881	7.524747	L		
C-C-0.597300	0	3.788859	-2.080593	7.119995	L		
O-O--0.567900	0	3.836407	-3.306939	7.266948	L		
C-CT--0.182500	0	1.812411	-1.826170	8.735134	L		
H-H-0.271900	0	1.361070	-2.083079	5.988968	L		
H-H1-0.082300	0	2.914314	-0.257530	7.781452	L		
H-HC-0.060300	0	0.972839	-1.172624	8.975296	L		
H-HC-0.060300	0	2.483377	-1.882134	9.592059	L		
H-HC-0.060300	0	1.435461	-2.824147	8.505616	L		
N-N--0.4157	0	4.759732	-1.383590	6.542136	L		
C-CT-0.0188	-1	5.964797	-1.964630	5.994598	L	H-HC	200
C-C-0.5973	0	7.161283	-1.820347	6.944205	L		
O-O--0.5679	0	7.209639	-0.969864	7.835231	L		
C-CT--0.517456	0	6.221105	-1.321240	4.628846	H		
C-CM-0.287351	0	5.357996	-1.796958	3.517924	H		
N-N2--0.586994	0	5.506215	-3.048667	2.936336	H		
C-CM--0.020338	0	4.387380	-1.184425	2.775065	H		
C-CM-0.242458	0	4.650139	-3.132837	1.882823	H		
N-N2--0.505070	0	3.963281	-2.012837	1.755979	H		
H-H-0.2719	0	4.656464	-0.373937	6.489798	L		
H-H1-0.0881	0	5.818033	-3.030246	5.848928	L		
H-HC-0.166355	0	7.277684	-1.482721	4.368836	H		
H-HC-0.174481	0	6.104144	-0.243355	4.767727	H		
H-H-0.335795	0	6.149550	-3.768330	3.258948	H		
H-H4-0.152777	0	3.984925	-0.190300	2.898419	H		
H-H5-0.209475	0	4.555358	-3.996708	1.239690	H		
N-N--0.4157	0	8.164726	-2.660286	6.696965	L		
C-CT--0.1490	-1	9.435025	-2.590673	7.385998	L		
H-H-0.2719	0	8.012426	-3.337977	5.956643	L		
H-H1-0.0976	0	10.107215	-3.360173	7.005123	L		
H-H1-0.0976	0	9.278902	-2.739596	8.455854	L		
H-H1-0.0976	0	9.882522	-1.607546	7.229044	L		
C-CT--0.3662	-1	8.358294	-9.387455	0.902554	L		
C-C-0.5972	0	8.730037	-9.142394	-0.547311	L		
O-O--0.5679	0	9.749774	-8.514141	-0.804304	L		
H-HC-0.1123	0	8.296574	-10.456317	1.093130	L		

H-HC-0.1123	0	7.410933	-8.899186	1.123523	L		
H-HC-0.1123	0	9.131057	-8.950471	1.534922	L		
N-N--0.254800	0	7.919264	-9.609731	-1.513126	L		
C-CT--0.026600	-1	8.160830	-9.366182	-2.937477	L		
C-C-0.589600	0	8.006515	-7.892863	-3.315537	L		
O-O--0.574800	0	8.713030	-7.393836	-4.188367	L		
C-CT--0.007000	0	7.158986	-10.262096	-3.673591	L		
C-CT-0.018900	0	6.002071	-10.402562	-2.683123	L		
C-CT-0.019200	0	6.697482	-10.379498	-1.321248	L		
H-H1-0.064100	0	9.174792	-9.671896	-3.198593	L		
H-HC-0.025300	0	7.609590	-11.241103	-3.843593	L		
H-HC-0.025300	0	6.832871	-9.827790	-4.619957	L		
H-HC-0.021300	0	5.447287	-11.328555	-2.837185	L		
H-HC-0.021300	0	5.337583	-9.541411	-2.769650	L		
H-H1-0.039100	0	6.045928	-9.920637	-0.576249	L		
H-H1-0.039100	0	6.955131	-11.396179	-1.021416	L		
N-N--0.516300	0	7.086168	-7.198046	-2.645780	L		
C-CT-0.039700	-1	6.837618	-5.770056	-2.814681	L		
C-C-0.536600	0	8.099115	-4.908692	-2.681047	L		
O-O--0.581900	0	8.275119	-3.921684	-3.399101	L		
C-CT-0.056000	0	5.737199	-5.329129	-1.844057	L	H-HC	244
C-CT--0.515956	0	5.221235	-3.936135	-2.217958	H		
C-C-0.587828	0	4.123815	-3.395601	-1.310210	H		
O-O2--0.600319	0	4.057823	-2.114150	-1.288396	H		
O-O2--0.589127	0	3.371914	-4.183906	-0.691620	H		
H-H-0.293600	0	6.556715	-7.675461	-1.935460	L		
H-H1-0.110500	0	6.466204	-5.613936	-3.827855	L		
H-HC--0.017300	0	6.125785	-5.319315	-0.824593	L		
H-HC--0.017300	0	4.908748	-6.037175	-1.901448	L		
H-HC-0.167759	0	4.828222	-3.974602	-3.240839	H		
H-HC-0.135230	0	6.049782	-3.219191	-2.217135	H		
N-N--0.415700	0	9.008954	-5.318751	-1.799756	L		
C-CT--0.024900	-1	10.246005	-4.615425	-1.481094	L		
C-C-0.597300	0	11.266889	-4.498040	-2.605975	L		
O-O--0.567900	0	12.243700	-3.760958	-2.471163	L		
C-CT-0.211700	0	10.888342	-5.252810	-0.242923	L		
O-OH--0.654600	0	9.933203	-5.443767	0.790408	L		
H-H-0.271900	0	8.802582	-6.132867	-1.237277	L		
H-H1-0.084300	0	9.976647	-3.593009	-1.211850	L		
H-H1-0.035200	0	11.687717	-4.604972	0.119773	L		
H-H1-0.035200	0	11.314626	-6.220364	-0.512172	L		
H-HO-0.427500	0	9.374186	-4.638497	0.863831	L		
N-N--0.415700	0	11.029379	-5.180556	-3.724308	L		
C-CT-0.033700	-1	11.779461	-5.013297	-4.962177	L		
C-C-0.597300	0	10.954867	-4.435190	-6.116309	L		
O-O--0.567900	0	11.395233	-4.427026	-7.264952	L		
C-CT--0.182500	0	12.388884	-6.378825	-5.311627	L		
H-H-0.271900	0	10.217659	-5.787703	-3.733098	L		
H-H1-0.082300	0	12.604440	-4.317977	-4.801223	L		
H-HC-0.060300	0	13.001732	-6.733420	-4.482159	L		
H-HC-0.060300	0	13.015561	-6.286192	-6.199331	L		
H-HC-0.060300	0	11.593589	-7.099577	-5.508514	L		
N-N--0.415700	0	9.772519	-3.924648	-5.790621	L		
C-CT--0.025200	-1	8.865802	-3.249445	-6.695974	L		

```
C-C-0.597300    0     8.788514   -1.748166   -6.468348 L
O-O--0.567900   0     8.777140   -0.971536   -7.420689 L
H-H-0.271900    0     9.488140   -3.995570   -4.819832 L
H-H1-0.069800   0     7.865002   -3.659321   -6.563774 L
H-H1-0.069800   0     9.166312   -3.420835   -7.730113 L
N-N--0.415700   0     8.736611   -1.347213   -5.201456 L
C-CT--0.002400  -1    8.584317    0.038575   -4.777775 L
C-C-0.597300    0     9.709923    0.466054   -3.828296 L
O-O--0.567900   0    10.337452   -0.388967   -3.195351 L
C-CT--0.034300  0     7.208885    0.177702   -4.099176 L
C-CA-0.011800   0     6.036969    0.000548   -5.046153 L
C-CA--0.125600  0     5.731297    1.007248   -5.981626 L
C-CA--0.125600  0     5.264075   -1.175014   -5.011588 L
C-CA--0.170400  0     4.665674    0.836337   -6.883439 L
C-CA--0.170400  0     4.196888   -1.344653   -5.911377 L
C-CA--0.107200  0     3.901944   -0.343509   -6.852651 L
H-H-0.271900    0     8.756224   -2.056525   -4.477996 L
H-H1-0.097800   0     8.612280    0.690973   -5.650510 L
H-HC-0.029500   0     7.120984    1.160043   -3.636008 L
H-HC-0.029500   0     7.140843   -0.557157   -3.295197 L
H-HA-0.133000   0     6.322292    1.910559   -6.016954 L
H-HA-0.133000   0     5.491754   -1.952466   -4.297625 L
H-HA-0.143000   0     4.437079    1.609765   -7.602862 L
H-HA-0.143000   0     3.601476   -2.245431   -5.879961 L
H-HA-0.129700   0     3.082629   -0.479050   -7.544513 L
N-N--0.254800   0     9.963616    1.779592   -3.674517 L
C-CT--0.026600  -1   10.822076    2.270380   -2.602147 L
C-C-0.589600    0    10.187613    2.010715   -1.227026 L
O-O--0.574800   0     8.993927    1.700998   -1.151348 L
C-CT--0.007000  0    10.977704    3.772343   -2.874843 L
C-CT-0.018900   0     9.662094    4.141361   -3.557888 L
C-CT-0.019200   0     9.360103    2.897959   -4.394059 L
H-H1-0.064100   0    11.793650    1.780438   -2.658759 L
H-HC-0.025300   0    11.804612    3.932526   -3.568229 L
H-HC-0.025300   0    11.130595    4.351580   -1.962895 L
H-HC-0.021300   0     9.759453    5.034407   -4.176004 L
H-HC-0.021300   0     8.882173    4.279149   -2.806308 L
H-H1-0.039100   0     8.282752    2.788394   -4.508726 L
H-H1-0.039100   0     9.831351    2.990918   -5.373359 L
N-N--0.254800   0    10.952096    2.122604   -0.130127 L
C-CT--0.026600  -1   10.398112    2.112209    1.217039 L
C-C-0.589600    0     9.342918    3.195204    1.444479 L
O-O--0.574800   0     9.597530    4.374067    1.208646 L
C-CT--0.007000  0    11.596408    2.303085    2.157554 L
C-CT-0.018900   0    12.785305    1.816916    1.332245 L
C-CT-0.019200   0    12.401730    2.229007   -0.086107 L
H-H1-0.064100   0     9.959741    1.129956    1.399432 L
H-HC-0.025300   0    11.480702    1.726489    3.075854 L
H-HC-0.025300   0    11.735523    3.360455    2.390358 L
H-HC-0.021300   0    12.852633    0.729175    1.390231 L
H-HC-0.021300   0    13.719461    2.280204    1.650697 L
H-H1-0.039100   0    12.696832    3.263645   -0.266040 L
H-H1-0.039100   0    12.880278    1.562396   -0.804392 L
N-N--0.4157     0     8.177044    2.770885    1.921872 L
```

C-CT-0.0188	-1	7.021105	3.599407	2.218388	L	H-HC	334
C-C-0.5973	0	6.159693	3.007880	3.331048	L		
O-O--0.5679	0	6.159775	1.795686	3.544125	L		
C-CT--0.515368	0	6.196729	3.721866	0.908286	H		
C-CM-0.287903	0	5.597438	2.446954	0.392415	H		
N-N2--0.582625	0	6.236868	1.647983	-0.542174	H		
C-CM--0.036893	0	4.460090	1.739515	0.709216	H		
C-CM-0.243454	0	5.497035	0.528835	-0.747605	H		
N-N2--0.523998	0	4.416203	0.548584	0.010147	H		
H-H-0.2719	0	8.043895	1.774105	2.015277	L		
H-H1-0.0881	0	7.356794	4.593303	2.529609	L		
H-HC-0.162301	0	6.850579	4.178203	0.150675	H		
H-HC-0.188340	0	5.403447	4.456576	1.093191	H		
H-H-0.339448	0	7.149822	1.847911	-0.950492	H		
H-H4-0.185957	0	3.672079	2.009337	1.397629	H		
H-H5-0.211295	0	5.774237	-0.268936	-1.421518	H		
N-N--0.415700	0	5.400147	3.873420	3.991244	L		
C-CT-0.014300	-1	4.555520	3.576977	5.139226	L		
C-C-0.597300	0	5.205712	2.624307	6.153651	L		
O-O--0.567900	0	4.681588	1.571114	6.519767	L		
C-CT--0.204100	0	3.107917	3.298165	4.710201	L		
C-C-0.713000	0	2.169129	4.176363	5.516264	L		
N-N--0.919100	0	1.598693	3.693188	6.585164	L		
O-O--0.593100	0	1.971520	5.344044	5.228204	L		
H-H-0.271900	0	5.547437	4.862398	3.780643	L		
H-H1-0.104800	0	4.520218	4.528830	5.674207	L		
H-HC-0.079700	0	2.873710	2.248827	4.855424	L		
H-HC-0.079700	0	2.967296	3.544538	3.658102	L		
H-H-0.419600	0	0.941326	4.272049	7.061595	L		
H-H-0.419600	0	1.655852	2.684738	6.729014	L		
N-N--0.4157	0	6.392444	3.054117	6.595469	L		
C-CT--0.1490	-1	7.299118	2.308049	7.446332	L		
H-H-0.2719	0	6.705257	3.929343	6.198752	L		
H-H1-0.0976	0	8.051452	2.971548	7.872433	L		
H-H1-0.0976	0	7.789322	1.527421	6.861453	L		
H-H1-0.0976	0	6.740597	1.825517	8.251602	L		
C-CT--0.3662	-1	-2.250970	-2.765933	9.537794	L		
C-C-0.5972	0	-2.379007	-3.852276	8.505232	L		
O-O--0.5679	0	-2.924823	-4.913941	8.792883	L		
H-HC-0.1123	0	-1.196323	-2.552380	9.700619	L		
H-HC-0.1123	0	-2.708975	-3.100996	10.466042	L		
H-HC-0.1123	0	-2.755188	-1.870844	9.178509	L		
N-N--0.347900	0	-1.848500	-3.589641	7.314175	L		
C-CT--0.240000	-1	-1.744262	-4.554346	6.213105	L		
C-C-0.734100	0	-0.334075	-4.588426	5.636458	L		
O-O--0.589400	0	0.480651	-3.706527	5.892612	L		
C-CT--0.009400	0	-2.832310	-4.259585	5.159468	L		
C-CT-0.018700	0	-2.598474	-2.977290	4.348889	L	H-HC	379
C-CT--0.464524	0	-3.690055	-2.792847	3.287570	H		
C-CT--0.195159	0	-3.579514	-1.488698	2.506795	H		
N-N3--0.748751	0	-2.370576	-1.416532	1.620312	H		
H-H-0.274700	0	-1.451876	-2.661917	7.180320	L		
H-H1-0.142600	0	-1.935214	-5.550888	6.612062	L		
H-HC-0.036200	0	-3.799041	-4.191117	5.661304	L		

H-HC-0.036200	0	-2.889322	-5.091493	4.464719	L
H-HC-0.010300	0	-1.626883	-3.021477	3.856587	L
H-HC-0.010300	0	-2.622544	-2.126569	5.022388	L
H-HC-0.173903	0	-4.661394	-2.794798	3.791149	H
H-HC-0.148535	0	-3.678260	-3.654818	2.605846	H
H-HP-0.236066	0	-3.548433	-0.604955	3.143440	H
H-HP-0.172509	0	-4.439550	-1.387927	1.832818	H
H-H-0.437293	0	-2.215243	-0.408108	1.259978	H
H-H-0.366736	0	-2.514250	-2.016621	0.801096	H
H-H-0.419451	0	-1.469802	-1.670330	2.073474	H
N-N--0.415700	0	-0.036552	-5.620923	4.860440	L
C-CT--0.025200	-1	1.277365	-5.816653	4.256877	L
C-C-0.597300	0	1.155189	-6.529544	2.909242	L
O-O--0.567900	0	1.749282	-7.579138	2.672347	L
H-H-0.271900	0	-0.778071	-6.277298	4.634682	L
H-H1-0.069800	0	1.899303	-6.419158	4.919040	L
H-H1-0.069800	0	1.768516	-4.857206	4.090946	L
N-N--0.415700	0	0.333644	-5.945503	2.041874	L
C-CT--0.025200	-1	-0.231994	-6.538011	0.833289	L
C-C-0.597300	0	-1.368613	-5.650941	0.391529	L
O-O--0.567900	0	-2.258154	-5.391964	1.193881	L
H-H-0.271900	0	-0.115289	-5.103323	2.376564	L
H-H1-0.069800	0	-0.616680	-7.533458	1.055082	L
H-H1-0.069800	0	0.533800	-6.597898	0.059451	L
N-N--0.4157	0	-1.319033	-5.180163	-0.853712	L
C-CT--0.0014	-1	-2.156600	-4.097987	-1.372923	L
C-C-0.5973	0	-3.622197	-4.172497	-0.931527	L
O-O--0.5679	0	-4.187979	-3.201358	-0.439965	L
C-CT--0.0152	0	-2.056998	-4.069410	-2.911737	L
C-CA--0.0011	0	-0.664356	-4.240684	-3.493972	L
C-CA--0.1906	0	0.345830	-3.302562	-3.208965	L
C-CA--0.1906	0	-0.384097	-5.338645	-4.332251	L
C-CA--0.2341	0	1.634187	-3.463221	-3.753045	L
C-CA--0.2341	0	0.902313	-5.502122	-4.880569	L
C-C-0.3226	0	1.915518	-4.562497	-4.589757	L
O-OH--0.5579	0	3.152418	-4.718331	-5.131667	L
H-H-0.2719	0	-0.495287	-5.389483	-1.399552	L
H-H1-0.0876	0	-1.751899	-3.159264	-0.994347	L
H-HC-0.0295	0	-2.452325	-3.122042	-3.271083	L
H-HC-0.0295	0	-2.697747	-4.851747	-3.319379	L
H-HA-0.1699	0	0.138958	-2.449874	-2.573456	L
H-HA-0.1699	0	-1.163902	-6.046727	-4.572251	L
H-HA-0.1656	0	2.408024	-2.744307	-3.527886	L
H-HA-0.1656	0	1.109006	-6.336662	-5.530484	L
H-HO-0.3992	0	3.178664	-5.470188	-5.721307	L
N-N--0.415700	0	-4.219884	-5.355177	-1.090787	L
C-CT--0.024900	-1	-5.557795	-5.641673	-0.594754	L
C-C-0.597300	0	-5.686887	-5.566076	0.924753	L
O-O--0.567900	0	-5.055295	-6.327044	1.666920	L
C-CT-0.211700	0	-6.020961	-7.035310	-1.023407	L
O-OH--0.654600	-1	-7.278604	-7.341853	-0.437163	L
H-H-0.271900	0	-3.643331	-6.098451	-1.442000	L
H-H1-0.084300	0	-6.242964	-4.917086	-1.035241	L
H-H1-0.035200	0	-5.288329	-7.773729	-0.695557	L

H-H1-0.035200	0	-6.098699	-7.073627	-2.109960	L		
H-HO-0.427500	-1	-7.536326	-8.213515	-0.747131	L		
N-N--0.415700	0	-6.660176	-4.766299	1.353695	L		
C-CT-0.033700	-1	-7.155820	-4.674595	2.715616	L		
C-C-0.597300	0	-7.510816	-6.003024	3.388628	L		
O-O--0.567900	0	-7.444353	-6.110160	4.611295	L		
C-CT--0.182500	0	-8.347612	-3.716913	2.719979	L		
H-H-0.271900	0	-7.119112	-4.184316	0.659577	L		
H-H1-0.082300	0	-6.369298	-4.226871	3.322259	L		
H-HC-0.060300	0	-8.058281	-2.749906	2.304440	L		
H-HC-0.060300	0	-8.692568	-3.577517	3.739869	L		
H-HC-0.060300	0	-9.161285	-4.139245	2.136102	L		
N-N--0.415700	0	-7.900826	-7.007431	2.603150	L		
C-CT--0.025200	-1	-8.282255	-8.320776	3.107793	L		
C-C-0.597300	0	-7.322427	-9.447866	2.748856	L		
O-O--0.567900	0	-7.736424	-10.602900	2.676810	L		
H-H-0.271900	0	-7.884418	-6.838933	1.603706	L		
H-H1-0.069800	0	-9.259424	-8.575722	2.698744	L		
H-H1-0.069800	0	-8.378904	-8.302200	4.193599	L		
N-N--0.415700	0	-6.040029	-9.135768	2.567456	L		
C-CT--0.024900	-1	-4.966292	-10.129069	2.581156	L		
C-C-0.597300	0	-4.267166	-10.095709	3.934280	L		
O-O--0.567900	0	-3.946555	-9.021646	4.452642	L		
C-CT-0.211700	0	-3.973005	-9.833992	1.452541	L		
O-OH--0.654600	-1	-3.087524	-10.926481	1.261541	L		
H-H-0.271900	0	-5.786161	-8.154398	2.633208	L		
H-H1-0.084300	0	-5.377990	-11.124346	2.413234	L		
H-H1-0.035200	0	-3.408332	-8.928414	1.680863	L		
H-H1-0.035200	0	-4.529725	-9.674173	0.528350	L		
H-HO-0.427500	-1	-2.546738	-10.736134	0.491496	L		
N-N--0.4157	0	-4.068388	-11.274577	4.522972	L		
C-CT--0.1490	-1	-3.530927	-11.424593	5.864135	L		
H-H-0.2719	0	-4.347721	-12.092612	4.005203	L		
H-H1-0.0976	0	-3.324097	-12.473886	6.074718	L		
H-H1-0.0976	0	-4.246950	-11.043987	6.595872	L		
H-H1-0.0976	0	-2.605647	-10.851446	5.960756	L		
C-CT--0.3662	-1	-12.136560	5.091257	-0.632643	L		
C-C-0.5972	0	-10.707418	5.268437	-0.192197	L		
O-O--0.5679	0	-10.389640	4.988615	0.958174	L		
H-HC-0.1123	0	-12.530076	6.047417	-0.970669	L		
H-HC-0.1123	0	-12.722374	4.731731	0.211790	L		
H-HC-0.1123	0	-12.172561	4.358441	-1.436658	L		
N-N--0.415700	0	-9.858293	5.721525	-1.109964	L		
C-CT--0.025200	-1	-8.424581	5.881927	-0.882294	L		
C-C-0.597300	0	-7.610289	5.759864	-2.146502	L		
O-O--0.567900	0	-8.115561	6.060961	-3.234108	L		
H-H-0.271900	0	-10.173522	5.892622	-2.052579	L		
H-H1-0.069800	0	-8.113290	5.097494	-0.202737	L		
H-H1-0.069800	0	-8.221678	6.849080	-0.424522	L		
N-N--0.415700	0	-6.354231	5.341273	-1.973557	L	H-H1	489
C-CT--0.530209	-1	-5.339924	5.312472	-3.028166	H		
C-C-0.568093	0	-4.530756	4.030573	-2.926729	H		
O-O--0.528105	0	-4.363963	3.467688	-1.825648	H		
C-CT-0.298500	0	-4.439415	6.569814	-2.968244	L	H-H1	489

C-CT--0.319200	0	-5.230158	7.829161	-3.327861	L		
C-CT--0.319200	0	-3.773881	6.767445	-1.602910	L		
H-H-0.271900	0	-6.062990	5.061748	-1.038414	L		
H-H1-0.184096	0	-5.862459	5.343348	-3.990202	H		
H-HC--0.029700	0	-3.651867	6.463277	-3.713315	L		
H-HC-0.079100	0	-5.973642	8.036873	-2.560083	L		
H-HC-0.079100	0	-5.730999	7.684621	-4.284096	L		
H-HC-0.079100	0	-4.548446	8.675296	-3.403952	L		
H-HC-0.079100	0	-4.523662	6.897576	-0.823996	L		
H-HC-0.079100	0	-3.131211	7.645753	-1.629186	L		
H-HC-0.079100	0	-3.164611	5.897883	-1.356189	L		
N-N--0.610103	0	-3.964969	3.568498	-4.052480	H		
C-CT--0.314060	-1	-4.227695	4.026890	-5.408389	H		
C-C-0.597300	0	-5.351179	3.212897	-6.034199	L	H-H1	505
O-O--0.567900	0	-5.485858	2.011239	-5.804937	L		
C-CT--0.110200	0	-2.937257	3.897030	-6.253153	L	H-H1	505
C-CT-0.353100	0	-1.854082	4.965389	-5.970766	L		
C-CT--0.412100	0	-1.076080	4.755729	-4.668133	L		
C-CT--0.412100	0	-0.829770	4.964261	-7.109214	L		
H-H-0.398207	0	-3.428715	2.693555	-3.960551	H		
H-H1-0.165130	0	-4.517614	5.076738	-5.410670	H		
H-HC-0.045700	0	-3.224438	3.990988	-7.300784	L		
H-HC-0.045700	0	-2.509949	2.902626	-6.123464	L		
H-HC--0.036100	0	-2.323998	5.949145	-5.947154	L		
H-HC-0.100000	0	-0.656258	3.752084	-4.642118	L		
H-HC-0.100000	0	-1.741313	4.884775	-3.816259	L		
H-HC-0.100000	0	-0.277977	5.492357	-4.586318	L		
H-HC-0.100000	0	-0.327617	3.999067	-7.154227	L		
H-HC-0.100000	0	-0.090084	5.746163	-6.937214	L		
H-HC-0.100000	0	-1.329359	5.159252	-8.056825	L		
N-N--0.415700	0	-6.133202	3.861954	-6.892195	L		
C-CT--0.038900	-1	-7.318905	3.265714	-7.519958	L		
C-C-0.597300	0	-7.067430	1.929093	-8.206351	L		
O-O--0.567900	0	-7.936358	1.060313	-8.226267	L		
C-CT-0.365400	-1	-7.955694	4.193846	-8.578748	L		
O-OH--0.676100	-1	-7.893614	5.549547	-8.126340	L		
C-CT--0.243800	0	-9.432838	3.899955	-8.838605	L		
H-H-0.271900	0	-6.005494	4.858389	-6.990892	L		
H-H1-0.100700	0	-8.061356	3.121989	-6.741729	L		
H-H1-0.004300	0	-7.400091	4.120324	-9.514612	L		
H-HC-0.064200	0	-9.545359	2.894342	-9.243788	L		
H-HC-0.064200	0	-9.827531	4.610848	-9.564396	L		
H-HC-0.064200	0	-9.998290	3.972805	-7.908790	L		
H-HO-0.410200	-1	-8.791703	5.884943	-8.089911	L		
N-N--0.415700	0	-5.897608	1.802557	-8.829972	L		
C-CT--0.025200	-1	-5.512373	0.642724	-9.621846	L		
C-C-0.597300	0	-4.593552	-0.345903	-8.936781	L		
O-O--0.567900	0	-4.084839	-1.238007	-9.615332	L		
H-H-0.271900	0	-5.220910	2.533380	-8.672196	L		
H-H1-0.069800	0	-5.002988	0.988104	-10.520391	L		
H-H1-0.069800	0	-6.400465	0.095664	-9.938490	L		
N-N--0.415700	0	-4.377565	-0.171752	-7.628133	L	H-H1	545
C-CT--0.539097	-1	-3.470133	-0.978240	-6.822772	H		
C-C-0.572106	0	-4.229265	-1.421564	-5.568674	H		

O-O--0.533001	0	-4.970870	-2.399337	-5.660993	H		
C-CT-0.130300	0	-2.090455	-0.294446	-6.644426	L	H-H1	545
C-CT--0.043000	0	-1.454161	0.123787	-7.991155	L		
C-CT--0.320400	0	-1.182837	-1.295210	-5.907510	L		
C-CT--0.066000	0	-0.094703	0.823703	-7.862816	L		
H-H-0.271900	0	-4.857223	0.587402	-7.153758	L		
H-H1-0.180646	0	-3.294672	-1.906910	-7.377252	H		
H-HC-0.018700	0	-2.214091	0.599868	-6.041767	L		
H-HC-0.023600	0	-2.118946	0.827014	-8.491297	L		
H-HC-0.023600	0	-1.343426	-0.753886	-8.629238	L		
H-HC-0.088200	0	-1.077869	-2.208239	-6.492422	L		
H-HC-0.088200	0	-1.604563	-1.543939	-4.935574	L		
H-HC-0.088200	0	-0.198870	-0.871691	-5.726911	L		
H-HC-0.018600	0	-0.166257	1.641569	-7.147031	L		
H-HC-0.018600	0	0.196684	1.224534	-8.833713	L		
H-HC-0.018600	0	0.668881	0.118490	-7.537727	L		
N-N--0.611145	0	-4.091815	-0.671215	-4.448393	H		
C-CT--0.109296	-1	-4.635229	-1.091476	-3.161273	H		
C-C-0.597300	0	-6.162883	-1.085929	-3.135893	L	H-H1	564
O-O--0.567900	0	-6.796713	-1.994558	-2.589085	L		
C-CT--0.388038	0	-4.041894	-0.270950	-2.032454	H		
H-H-0.369964	0	-3.392108	0.071369	-4.395962	H		
H-H1-0.178541	0	-4.382385	-2.142282	-2.987412	H		
H-H1-0.157964	0	-4.518038	-0.591203	-1.098368	H		
H-H1-0.179833	0	-4.255310	0.793393	-2.158125	H		
H-HS-0.319500	0	-1.776502	1.767174	-0.521587	H		
N-N--0.415700	0	-6.752840	-0.104607	-3.808142	L		
C-CT--0.087500	-1	-8.191392	-0.047246	-4.054491	L		
C-C-0.597300	0	-8.669686	-1.200340	-4.922468	L		
O-O--0.567900	0	-9.597209	-1.911763	-4.538942	L		
C-CT-0.298500	0	-8.597017	1.314953	-4.635194	L		
C-CT--0.319200	0	-10.113193	1.432401	-4.841190	L		
C-CT--0.319200	0	-8.165896	2.484535	-3.735870	L		
H-H-0.271900	0	-6.116478	0.550730	-4.254867	L		
H-H1-0.096900	0	-8.695703	-0.152135	-3.095892	L		
H-HC--0.029700	0	-8.111807	1.413640	-5.597307	L		
H-HC-0.079100	0	-10.629684	1.271708	-3.894526	L		
H-HC-0.079100	0	-10.451964	0.693574	-5.567360	L		
H-HC-0.079100	0	-10.357025	2.423330	-5.223242	L		
H-HC-0.079100	0	-8.626702	2.400704	-2.753130	L		
H-HC-0.079100	0	-8.452769	3.433254	-4.188651	L		
H-HC-0.079100	0	-7.082474	2.494535	-3.611413	L		
N-N--0.415700	0	-8.045332	-1.397607	-6.081994	L		
C-CT--0.025200	-1	-8.401922	-2.454600	-7.024973	L		
C-C-0.597300	0	-8.317233	-3.857548	-6.436584	L		
O-O--0.567900	0	-9.219829	-4.668965	-6.628838	L		
H-H-0.271900	0	-7.263363	-0.792703	-6.297019	L		
H-H1-0.069800	0	-7.731852	-2.404078	-7.882930	L		
H-H1-0.069800	0	-9.421217	-2.291011	-7.374408	L		
N-N--0.415700	0	-7.233486	-4.137371	-5.715374	L		
C-CT--0.025200	-1	-7.009276	-5.404163	-5.030212	L		
C-C-0.597300	0	-7.932864	-5.665285	-3.851358	L		
O-O--0.567900	0	-8.253962	-6.818578	-3.571330	L		
H-H-0.271900	0	-6.517034	-3.416345	-5.651862	L		

H-H1-0.069800	0	-5.983175	-5.426751	-4.665032	L
H-H1-0.069800	0	-7.135740	-6.219251	-5.743029	L
N-N--0.415700	0	-8.328672	-4.607881	-3.145635	L
C-CT--0.038900	-1	-9.379716	-4.674793	-2.127616	L
C-C-0.597300	0	-10.749054	-4.967846	-2.731965	L
O-O--0.567900	0	-11.492102	-5.804483	-2.219145	L
C-CT-0.365400	0	-9.424174	-3.379142	-1.303280	L
O-OH--0.676100	0	-8.190148	-3.210481	-0.643959	L
C-CT--0.243800	0	-10.511545	-3.402162	-0.229430	L
H-H-0.271900	0	-7.958520	-3.700020	-3.402602	L
H-H1-0.100700	0	-9.150662	-5.493696	-1.445665	L
H-H1-0.004300	0	-9.593999	-2.524139	-1.957727	L
H-HC-0.064200	0	-11.491980	-3.296425	-0.692610	L
H-HC-0.064200	0	-10.361103	-2.576279	0.465269	L
H-HC-0.064200	0	-10.475057	-4.345450	0.313862	L
H-HO-0.410200	0	-7.603517	-2.739174	-1.270873	L
N-N--0.4157	0	-11.098286	-4.257750	-3.805793	L
C-CT--0.1490	-1	-12.331807	-4.443812	-4.541367	L
H-H-0.2719	0	-10.430439	-3.575217	-4.152828	L
H-H1-0.0976	0	-12.403437	-3.693353	-5.329548	L
H-H1-0.0976	0	-13.183407	-4.351357	-3.865913	L
H-H1-0.0976	0	-12.336480	-5.438021	-4.991425	L
N-N2--0.511415	0	1.946967	-0.021592	-1.688800	H
C-CA-0.057801	0	0.654930	0.320378	-1.611646	H
C-CA-0.063496	0	-0.059365	0.834927	-2.697125	H
C-CA--0.109122	0	0.620500	1.047112	-3.893089	H
C-CA--0.164490	0	1.970960	0.720359	-3.968427	H
C-CA-0.098392	0	2.589489	0.168836	-2.851988	H
C-C-0.541263	0	-1.565083	1.100122	-2.651691	H
O-O--0.574884	0	-2.116632	1.472120	-3.752405	H
N-N--0.751108	0	-1.813034	2.172839	-1.513700	H
H-H4-0.220889	0	0.180350	0.172647	-0.646717	H
H-HA-0.160434	0	0.065512	1.443033	-4.735841	H
H-HA-0.142191	0	2.540806	0.882224	-4.877378	H
H-H4-0.174502	0	3.628194	-0.141623	-2.878186	H
H-H-0.412468	0	-2.764400	2.574101	-1.657606	H
H-H-0.353801	0	-1.145954	2.947996	-1.596958	H
Zn-ZN-0.974537	0	2.856986	-1.091423	-0.013925	H
O-OW--0.834000	0	-2.131071	-7.247835	3.472417	L
H-HW-0.417000	0	-2.585537	-6.663113	2.846528	L
H-HW-0.417000	0	-2.842675	-7.808872	3.822965	L
O-OW--0.783903	0	1.326667	-2.565180	-0.129475	H
H-HW-0.401253	0	0.958448	-2.468199	0.785759	H
H-HW-0.424111	0	1.940277	-3.341932	-0.189199	H
O-OW--0.834000	0	-3.635179	-8.455293	7.197501	L
H-HW-0.417000	0	-3.724945	-7.515233	7.373504	L
H-HW-0.417000	0	-3.771620	-8.530050	6.241069	L
O-OW--0.834000	0	7.198088	-0.014210	1.814099	L
H-HW-0.417000	0	6.888443	0.370486	2.653477	L
H-HW-0.417000	0	6.414680	0.099005	1.253597	L
O-OW--0.834000	0	7.056691	-4.958131	4.749628	L
H-HW-0.417000	0	6.107319	-5.026394	4.978937	L
H-HW-0.417000	0	7.275343	-5.842259	4.452230	L
O-OW--0.834000	0	8.572762	-0.936592	-0.297808	L

```
H-HW-0.417000   0      8.149138   -0.560262    0.499752 L
H-HW-0.417000   0      8.825975   -0.146036   -0.795554 L
O-OW--0.834000  0     12.622518   -1.065654   -1.923067 L
H-HW-0.417000   0     11.781451   -0.817625   -2.343766 L
H-HW-0.417000   0     12.644460   -2.025995   -2.054649 L
O-OW--0.834000  0      8.270287   -3.301423    0.969105 L
H-HW-0.417000   0      8.456052   -2.539686    0.381771 L
H-HW-0.417000   0      7.565671   -2.958140    1.528179 L
O-OW--0.834000  0      4.366073   -4.922064    5.152319 L
H-HW-0.417000   0      4.096580   -4.544244    6.012326 L
H-HW-0.417000   0      4.055456   -4.255509    4.529379 L
S-SH--0.326142  0     -2.240945   -0.557307   -1.886767 H
```

TS2

```
C-CT--0.366200  -1  -10.312375    3.923597    4.029548 L
C-C-0.597200     0   -8.976347    3.283860    3.782839 L
O-O--0.567900    0   -8.397071    2.713697    4.700529 L
H-HC-0.112300    0  -11.052219    3.458054    3.381340 L
H-HC-0.112300    0  -10.242979    4.986748    3.806316 L
H-HC-0.112300    0  -10.589811    3.780033    5.071554 L
N-N--0.415700    0   -8.510913    3.380649    2.543401 L
C-CT--0.059700  -1   -7.343618    2.657464    2.031481 L
C-C-0.597300     0   -6.181932    3.621162    1.828452 L
O-O--0.567900    0   -6.291754    4.574018    1.061819 L
C-CT-0.130300    0   -7.694875    1.901197    0.724487 L
C-CT--0.043000   0   -8.879755    0.925535    0.937800 L
C-CT--0.320400   0   -6.467481    1.125918    0.207920 L
C-CT--0.066000   0   -9.427250    0.313468   -0.356855 L
H-H-0.271900     0   -9.055479    3.935247    1.891117 L
H-H1-0.086900    0   -7.036537    1.912020    2.764882 L
H-HC-0.018700    0   -7.982465    2.634354   -0.031471 L
H-HC-0.023600    0   -9.710850    1.453025    1.404229 L
H-HC-0.023600    0   -8.574691    0.121823    1.609076 L
H-HC-0.088200    0   -6.147016    0.384137    0.940364 L
H-HC-0.088200    0   -5.639181    1.806777    0.009425 L
H-HC-0.088200    0   -6.696572    0.628989   -0.732559 L
H-HC-0.018600    0   -9.666616    1.102202   -1.069260 L
H-HC-0.018600    0  -10.336833   -0.243009   -0.133729 L
H-HC-0.018600    0   -8.699629   -0.370224   -0.790958 L
N-N--0.516300    0   -5.087835    3.367677    2.540010 L
C-CT-0.038100   -1   -3.776387    3.986351    2.403325 L H-HC 30
C-C-0.536600     0   -3.695354    5.504316    2.143616 L
O-O--0.581900    0   -2.711075    6.018786    1.609149 L
C-CT--0.547145   0   -3.003571    3.161579    1.370145 H
C-C-0.532653     0   -2.589983    1.777814    1.847481 H
O-O2--0.588507   0   -2.757396    1.425950    3.020265 H
O-O2--0.625559   0   -2.029538    1.033755    0.943606 H
H-H-0.293600     0   -5.087996    2.518245    3.092898 L
H-H1-0.088000    0   -3.263205    3.853900    3.356662 L
H-HC-0.128435    0   -2.079253    3.697851    1.109426 H
H-HC-0.183556    0   -3.580106    3.115701    0.441830 H
N-N--0.415700    0   -4.670251    6.258676    2.633745 L
C-CT--0.025200  -1   -4.647973    7.705215    2.536826 L
C-C-0.597300     0   -3.754436    8.326363    3.595128 L
```

O-O--0.567900	0	-4.234303	8.950327	4.541907	L
H-H-0.271900	0	-5.481830	5.762401	2.966787	L
H-H1-0.069800	0	-5.659167	8.086970	2.670540	L
H-H1-0.069800	0	-4.295706	8.008449	1.549719	L
N-N--0.415700	0	-2.449511	8.141383	3.430787	L
C-CT--0.038900	-1	-1.426466	8.557785	4.383796	L
C-C-0.597300	0	-0.520177	9.647984	3.802443	L
O-O--0.567900	0	-0.620564	10.027616	2.634311	L
C-CT-0.365400	0	-0.564564	7.381828	4.882255	L
O-OH--0.676100	0	0.480116	7.127472	3.979514	L
C-CT--0.243800	0	-1.331249	6.085699	5.128582	L
H-H-0.271900	0	-2.175275	7.570521	2.634070	L
H-H1-0.100700	0	-1.919668	8.982601	5.256448	L
H-H1-0.004300	0	-0.111830	7.682560	5.827684	L
H-HC-0.064200	0	-2.196079	6.282656	5.760948	L
H-HC-0.064200	0	-0.677739	5.373367	5.625946	L
H-HC-0.064200	0	-1.657384	5.655028	4.182067	L
H-HO-0.410200	0	1.029090	6.405912	4.355141	L
N-N--0.415700	0	0.404259	10.145797	4.628054	L
C-CT--0.025200	-1	1.444579	11.081873	4.209650	L
C-C-0.597300	0	2.317485	10.593246	3.061142	L
O-O--0.567900	0	2.589544	11.347842	2.132674	L
H-H-0.271900	0	0.454388	9.759974	5.555728	L
H-H1-0.069800	0	2.097175	11.299482	5.054153	L
H-H1-0.069800	0	0.974114	12.013441	3.894047	L
N-N--0.516300	0	2.752665	9.338767	3.139048	L
C-CT-0.038100	-1	3.650382	8.728931	2.159920	L
C-C-0.536600	0	3.011912	8.409255	0.820638	L
O-O--0.581900	0	3.740256	8.082596	-0.110010	L
C-CT--0.030300	0	4.225858	7.417041	2.724406	L
C-C-0.799400	-1	5.578066	7.610280	3.387226	L
O-O2--0.801400	-1	6.159777	8.713993	3.309307	L
O-O2--0.801400	-1	6.035970	6.654542	4.046096	L
H-H-0.293600	0	2.443040	8.756431	3.901334	L
H-H1-0.088000	0	4.455493	9.428565	1.932068	L
H-HC--0.012200	0	4.367911	6.686152	1.926545	L
H-HC--0.012200	0	3.524256	6.978851	3.436979	L
N-N--0.415700	0	1.689996	8.493176	0.706151	L
C-CT--0.002400	-1	0.983366	8.329183	-0.557726	L
C-C-0.597300	0	0.413526	9.637786	-1.101772	L
O-O--0.567900	0	0.371031	9.838082	-2.315701	L
C-CT--0.034300	0	-0.123215	7.273134	-0.410905	L
C-CA-0.011800	0	0.364365	5.850250	-0.190148	L
C-CA--0.125600	0	0.821586	5.079731	-1.276652	L
C-CA--0.125600	0	0.317446	5.274071	1.092249	L
C-CA--0.170400	0	1.223412	3.746490	-1.075180	L
C-CA--0.170400	0	0.727871	3.945396	1.296208	L
C-CA--0.107200	0	1.180426	3.181009	0.209668	L
H-H-0.271900	0	1.169288	8.710857	1.543692	L
H-H1-0.097800	0	1.670555	7.962067	-1.320012	L
H-HC-0.029500	0	-0.718438	7.274943	-1.324485	L
H-HC-0.029500	0	-0.786316	7.562568	0.405854	L
H-HA-0.133000	0	0.851970	5.510549	-2.266650	L
H-HA-0.133000	0	-0.071231	5.845190	1.918994	L

H-HA-0.143000	0	1.555341	3.140864	-1.903340	L
H-HA-0.143000	0	0.664539	3.505245	2.281146	L
H-HA-0.129700	0	1.478623	2.153653	0.359366	L
N-N--0.415700	0	-0.020282	10.537844	-0.218600	L
C-CT--0.149000	-1	-0.460848	11.865027	-0.592497	L
H-H-0.271900	0	0.005611	10.290200	0.765221	L
H-H1-0.097600	0	-0.820100	12.392485	0.291761	L
H-H1-0.097600	0	-1.265379	11.791925	-1.326152	L
H-H1-0.097600	0	0.373767	12.415678	-1.029169	L
C-CT--0.366200	-1	7.993058	8.434760	-0.115072	L
C-C-0.597200	0	7.526682	7.661370	-1.323997	L
O-O--0.567900	0	8.342745	7.347786	-2.186907	L
H-HC-0.112300	0	7.516577	9.412608	-0.103054	L
H-HC-0.112300	0	9.074112	8.547260	-0.155906	L
H-HC-0.112300	0	7.723294	7.888523	0.789589	L
N-N--0.415700	0	6.218743	7.398393	-1.411709	L
C-CT--0.051800	-1	5.572637	6.868313	-2.621607	L
C-C-0.597300	0	4.858545	7.944622	-3.436096	L
O-O--0.567900	0	5.035477	8.052494	-4.649189	L
C-CT--0.110200	0	4.701467	5.628289	-2.329982	L
C-CT-0.353100	0	5.080671	4.439241	-3.239367	L
C-CT--0.412100	0	4.188722	3.236303	-2.960364	L
C-CT--0.412100	0	4.935300	4.730794	-4.737993	L
H-H-0.271900	0	5.618465	7.684799	-0.645922	L
H-H1-0.092200	0	6.382211	6.535368	-3.270226	L
H-HC-0.045700	0	3.647457	5.874837	-2.465562	L
H-HC-0.045700	0	4.839194	5.322629	-1.292211	L
H-HC--0.036100	0	6.113157	4.153001	-3.034676	L
H-HC-0.100000	0	3.188504	3.425122	-3.345219	L
H-HC-0.100000	0	4.132373	3.052570	-1.888708	L
H-HC-0.100000	0	4.603096	2.354779	-3.446378	L
H-HC-0.100000	0	3.939039	5.122573	-4.944825	L
H-HC-0.100000	0	5.091228	3.820221	-5.313358	L
H-HC-0.100000	0	5.674161	5.465053	-5.055498	L
N-N--0.415700	0	4.071136	8.753229	-2.741399	L
C-CT--0.149000	-1	3.369740	9.902907	-3.243669	L
H-H-0.271900	0	3.996662	8.540252	-1.754775	L
H-H1-0.097600	0	4.032968	10.496636	-3.873934	L
H-H1-0.097600	0	3.016599	10.513377	-2.411302	L
H-H1-0.097600	0	2.511601	9.578535	-3.834887	L
C-CT--0.366200	-1	-8.733226	2.366207	8.135220	L
C-C-0.597200	0	-7.864309	1.154083	7.954867	L
O-O--0.567900	0	-7.540542	0.474117	8.921703	L
H-HC-0.112300	0	-9.656836	2.224989	7.577288	L
H-HC-0.112300	0	-8.204824	3.240605	7.758469	L
H-HC-0.112300	0	-8.952623	2.492654	9.193352	L
N-N--0.415700	0	-7.479081	0.901193	6.712442	L
C-CT--0.038900	-1	-6.672117	-0.248540	6.298615	L
C-C-0.597300	0	-5.410907	0.248747	5.615416	L
O-O--0.567900	0	-5.462066	0.707218	4.474522	L
C-CT-0.365400	0	-7.474661	-1.214809	5.414275	L
O-OH--0.676100	0	-8.025367	-0.547799	4.304204	L
C-CT--0.243800	0	-8.625479	-1.839949	6.208176	L
H-H-0.271900	0	-7.808757	1.523944	5.978766	L

```
H-H1-0.100700    0   -6.368266   -0.811842    7.180679 L
H-H1-0.004300    0   -6.813664   -2.008191    5.063437 L
H-HC-0.064200    0   -8.229657   -2.373407    7.072056 L
H-HC-0.064200    0   -9.175877   -2.537964    5.583691 L
H-HC-0.064200    0   -9.315387   -1.066553    6.544140 L
H-HO-0.410200    0   -7.335986    0.046461    3.974016 L
N-N--0.415700    0   -4.312330    0.220640    6.373721 L
C-CT--0.059700  -1   -3.117306    1.043132    6.171224 L
C-C-0.597300     0   -1.870865    0.164565    6.097659 L
O-O--0.567900    0   -1.613129   -0.638938    6.998402 L
C-CT-0.130300    0   -3.000852    2.096831    7.298121 L
C-CT--0.043000   0   -4.253566    2.994945    7.454639 L
C-CT--0.320400   0   -1.753145    2.970732    7.095685 L
C-CT--0.066000   0   -4.656244    3.810274    6.216403 L
H-H-0.271900     0   -4.375552   -0.239939    7.267131 L
H-H1-0.086900    0   -3.218489    1.588936    5.240684 L
H-HC-0.018700    0   -2.873733    1.565643    8.243383 L
H-HC-0.023600    0   -4.079175    3.691206    8.275711 L
H-HC-0.023600    0   -5.101262    2.375879    7.746886 L
H-HC-0.088200    0   -1.733406    3.354730    6.075737 L
H-HC-0.088200    0   -0.856512    2.371699    7.258247 L
H-HC-0.088200    0   -1.747474    3.797926    7.804418 L
H-HC-0.018600    0   -3.846709    4.473330    5.915629 L
H-HC-0.018600    0   -5.533328    4.412927    6.452623 L
H-HC-0.018600    0   -4.906511    3.148842    5.389839 L
N-N--0.516300    0   -1.118569    0.343475    5.017849 L
C-CT-0.038100   -1    0.179044   -0.242665    4.736241 L H-HC 178
C-C-0.536600     0    1.119750   -0.086147    5.937184 L
O-O--0.581900    0    1.335794    1.007588    6.454091 L
C-CT--0.520019   0    0.671226    0.402789    3.424718 H
C-C-0.636404     0    0.974345   -0.559405    2.286797 H
O-O2--0.608036   0    0.382406   -1.675173    2.206374 H
O-O2--0.608131   0    1.815068   -0.109638    1.439314 H
H-H-0.293600     0   -1.523227    0.934492    4.298530 L
H-H1-0.088000    0    0.022835   -1.310770    4.577403 L
H-HC-0.132300    0    1.568841    0.992230    3.623903 H
H-HC-0.188993    0   -0.079978    1.101555    3.040997 H
N-N--0.415700    0    1.660225   -1.199681    6.399928 L
C-CT-0.033700   -1    2.558597   -1.255891    7.530332 L
C-C-0.597300     0    3.778260   -2.076676    7.126958 L
O-O--0.567900    0    3.824651   -3.302917    7.275213 L
C-CT--0.182500   0    1.799054   -1.821410    8.738689 L
H-H-0.271900     0    1.351781   -2.075376    5.992092 L
H-H1-0.082300    0    2.904432   -0.252989    7.788291 L
H-HC-0.060300    0    0.959964   -1.166909    8.977955 L
H-HC-0.060300    0    2.468289   -1.879282    9.596838 L
H-HC-0.060300    0    1.421035   -2.818567    8.507333 L
N-N--0.415700    0    4.750753   -1.380558    6.550784 L
C-CT-0.018800   -1    5.955310   -1.962787    6.003363 L H-HC 200
C-C-0.597300     0    7.152230   -1.817355    6.952192 L
O-O--0.567900    0    7.200931   -0.965550    7.841918 L
C-CT--0.517456   0    6.210852   -1.321510    4.636635 H
C-CM-0.287351    0    5.344951   -1.796845    3.527704 H
N-N2--0.586994   0    5.489756   -3.049779    2.947739 H
```

C-CM--0.020338	0	4.373901	-1.183699	2.786040	H	
C-CM-0.242458	0	4.631393	-3.134333	1.896302	H	
N-N2--0.505070	0	3.946267	-2.013303	1.769400	H	
H-H-0.271900	0	4.648451	-0.370805	6.498479	L	
H-H1-0.088100	0	5.808072	-3.028588	5.859368	L	
H-HC-0.166355	0	7.266735	-1.485695	4.375498	H	
H-HC-0.174481	0	6.095780	-0.243354	4.774675	H	
H-H-0.335795	0	6.132275	-3.770094	3.270460	H	
H-H4-0.152777	0	3.973049	-0.188795	2.908497	H	
H-H5-0.209475	0	4.534206	-3.998733	1.254208	H	
N-N--0.415700	0	8.154541	-2.659318	6.707437	L	
C-CT--0.149000	-1	9.423933	-2.590574	7.398186	L	
H-H-0.271900	0	8.002050	-3.338028	5.968080	L	
H-H1-0.097600	0	10.095529	-3.361634	7.019423	L	
H-H1-0.097600	0	9.266044	-2.737724	8.468032	L	
H-H1-0.097600	0	9.873137	-1.608348	7.240475	L	
C-CT--0.366200	-1	8.348021	-9.389909	0.917282	L	
C-C-0.597200	0	8.721629	-9.146697	-0.532429	L	
O-O--0.567900	0	9.742894	-8.520721	-0.788920	L	
H-HC-0.112300	0	8.284240	-10.458505	1.108668	L	
H-HC-0.112300	0	7.401288	-8.899814	1.136895	L	
H-HC-0.112300	0	9.120915	-8.953740	1.550051	L	
N-N--0.254800	0	7.910735	-9.612948	-1.498667	L	
C-CT--0.026600	-1	8.153883	-9.370457	-2.922929	L	
C-C-0.589600	0	8.002085	-7.897044	-3.301644	L	
O-O--0.574800	0	8.711093	-7.398784	-4.172900	L	
C-CT--0.007000	0	7.151286	-10.265191	-3.659467	L	
C-CT-0.018900	0	5.993360	-10.403627	-2.669884	L	
C-CT-0.019200	0	6.687634	-10.380783	-1.307419	L	
H-H1-0.064100	0	9.167600	-9.677810	-3.183061	L	
H-HC-0.025300	0	7.600598	-11.244917	-3.828742	L	
H-HC-0.025300	0	6.826553	-9.830778	-4.606260	L	
H-HC-0.021300	0	5.437492	-11.328970	-2.823941	L	
H-HC-0.021300	0	5.330052	-9.541670	-2.757417	L	
H-H1-0.039100	0	6.036114	-9.920513	-0.563259	L	
H-H1-0.039100	0	6.943499	-11.397670	-1.006760	L	
N-N--0.516300	0	7.080924	-7.201402	-2.633918	L	
C-CT-0.039700	-1	6.833093	-5.773341	-2.803109	L	
C-C-0.536600	0	8.094890	-4.912555	-2.668583	L	
O-O--0.581900	0	8.271679	-3.925522	-3.386365	L	
C-CT-0.056000	0	5.732121	-5.332545	-1.832806	L	H-HC 244
C-CT--0.515956	0	5.212560	-3.941899	-2.209813	H	
C-C-0.587828	0	4.117856	-3.398919	-1.299702	H	
O-O2--0.600319	0	4.060888	-2.117336	-1.271043	H	
O-O2--0.589127	0	3.358605	-4.184982	-0.687137	H	
H-H-0.293600	0	6.549652	-7.678658	-1.924855	L	
H-H1-0.110500	0	6.462278	-5.617150	-3.816504	L	
H-HC--0.017300	0	6.121512	-5.319176	-0.813658	L	
H-HC--0.017300	0	4.905140	-6.042462	-1.887417	L	
H-HC-0.167759	0	4.815812	-3.985645	-3.231118	H	
H-HC-0.135230	0	6.040494	-3.224371	-2.215100	H	
N-N--0.415700	0	9.003801	-5.322864	-1.786476	L	
C-CT--0.024900	-1	10.241140	-4.620422	-1.467169	L	
C-C-0.597300	0	11.262059	-4.502633	-2.591906	L	

O-O--0.567900	0	12.237482	-3.763614	-2.457588	L
C-CT-0.211700	0	10.883278	-5.259309	-0.229659	L
O-OH--0.654600	0	9.928142	-5.451830	0.803389	L
H-H-0.271900	0	8.796229	-6.136706	-1.224051	L
H-H1-0.084300	0	9.972255	-3.598126	-1.197010	L
H-H1-0.035200	0	11.682551	-4.611849	0.133936	L
H-H1-0.035200	0	11.309726	-6.226467	-0.500079	L
H-HO-0.427500	0	9.367533	-4.647665	0.876606	L
N-N--0.415700	0	11.026372	-5.187358	-3.709264	L
C-CT-0.033700	-1	11.777317	-5.021147	-4.946724	L
C-C-0.597300	0	10.955200	-4.440013	-6.101032	L
O-O--0.567900	0	11.399483	-4.426652	-7.248115	L
C-CT--0.182500	0	12.382939	-6.388110	-5.297118	L
H-H-0.271900	0	10.215257	-5.795352	-3.717666	L
H-H1-0.082300	0	12.604272	-4.328397	-4.784847	L
H-HC-0.060300	0	12.994563	-6.745127	-4.467786	L
H-HC-0.060300	0	13.010086	-6.296545	-6.184602	L
H-HC-0.060300	0	11.585654	-7.106456	-5.494772	L
N-N--0.415700	0	9.770507	-3.933484	-5.777626	L
C-CT--0.025200	-1	8.866395	-3.256136	-6.683935	L
C-C-0.597300	0	8.792618	-1.754676	-6.456362	L
O-O--0.567900	0	8.785250	-0.978016	-7.408805	L
H-H-0.271900	0	9.483118	-4.007265	-4.807935	L
H-H1-0.069800	0	7.864424	-3.663468	-6.552655	L
H-H1-0.069800	0	9.167478	-3.428411	-7.717758	L
N-N--0.415700	0	8.738864	-1.353251	-5.189575	L
C-CT--0.002400	-1	8.585565	0.033060	-4.767656	L
C-C-0.597300	0	9.705397	0.460133	-3.811863	L
O-O--0.567900	0	10.326044	-0.394908	-3.172210	L
C-CT--0.034300	0	7.203696	0.175399	-4.103306	L
C-CA-0.011800	0	6.041821	-0.010893	-5.061440	L
C-CA--0.125600	0	5.773540	0.966477	-6.038680	L
C-CA--0.125600	0	5.240916	-1.166283	-4.995559	L
C-CA--0.170400	0	4.716244	0.787612	-6.948674	L
C-CA--0.170400	0	4.182155	-1.343860	-5.903821	L
C-CA--0.107200	0	3.923017	-0.371160	-6.884600	L
H-H-0.271900	0	8.754561	-2.062466	-4.465858	L
H-H1-0.097800	0	8.622792	0.683920	-5.641089	L
H-HC-0.029500	0	7.109995	1.161190	-3.648836	L
H-HC-0.029500	0	7.128979	-0.553566	-3.294584	L
H-HA-0.133000	0	6.387393	1.852887	-6.100292	L
H-HA-0.133000	0	5.438386	-1.921753	-4.249697	L
H-HA-0.143000	0	4.516590	1.538651	-7.699686	L
H-HA-0.143000	0	3.564105	-2.227939	-5.847107	L
H-HA-0.129700	0	3.109226	-0.512409	-7.581804	L
N-N--0.254800	0	9.963016	1.773374	-3.662053	L
C-CT--0.026600	-1	10.823014	2.264403	-2.591235	L
C-C-0.589600	0	10.188515	2.005560	-1.215852	L
O-O--0.574800	0	8.995310	1.694302	-1.139902	L
C-CT--0.007000	0	10.980550	3.765939	-2.865246	L
C-CT-0.018900	0	9.666633	4.135712	-3.551085	L
C-CT-0.019200	0	9.364651	2.891571	-4.386105	L
H-H1-0.064100	0	11.793898	1.773034	-2.647770	L
H-HC-0.025300	0	11.808892	3.924562	-3.557273	L

H-HC-0.025300	0	11.132358	4.345943	-1.953608	L	
H-HC-0.021300	0	9.766112	5.027835	-4.170185	L	
H-HC-0.021300	0	8.885686	4.275376	-2.800949	L	
H-H1-0.039100	0	8.287442	2.784428	-4.504383	L	
H-H1-0.039100	0	9.839662	2.981554	-5.363864	L	
N-N--0.254800	0	10.951960	2.118256	-0.118535	L	
C-CT--0.026600	-1	10.395645	2.108479	1.227662	L	
C-C-0.589600	0	9.340961	3.192667	1.452131	L	
O-O--0.574800	0	9.594968	4.370466	1.210398	L	
C-CT--0.007000	0	11.592569	2.299827	2.169845	L	
C-CT-0.018900	0	12.783241	1.815038	1.346129	L	
C-CT-0.019200	0	12.401283	2.226543	-0.072838	L	
H-H1-0.064100	0	9.956486	1.126592	1.410040	L	
H-HC-0.025300	0	11.476039	1.722763	3.087748	L	
H-HC-0.025300	0	11.730395	3.357225	2.403319	L	
H-HC-0.021300	0	12.851783	0.727382	1.404272	L	
H-HC-0.021300	0	13.716405	2.279427	1.665886	L	
H-H1-0.039100	0	12.695267	3.261562	-0.252417	L	
H-H1-0.039100	0	12.881487	1.560512	-0.790565	L	
N-N--0.415700	0	8.175867	2.770273	1.932802	L	
C-CT-0.018800	-1	7.018773	3.598535	2.225297	L	H-HC 334
C-C-0.597300	0	6.158894	3.009711	3.340393	L	
O-O--0.567900	0	6.159449	1.797936	3.555882	L	
C-CT--0.515368	0	6.194521	3.715294	0.915211	H	
C-CM-0.287903	0	5.598949	2.436648	0.405979	H	
N-N2--0.582625	0	6.247334	1.627654	-0.513422	H	
C-CM--0.036893	0	4.457847	1.733682	0.718440	H	
C-CM-0.243454	0	5.508181	0.507998	-0.716649	H	
N-N2--0.523998	0	4.419339	0.536867	0.029414	H	
H-H-0.271900	0	8.044315	1.773913	2.032611	L	
H-H1-0.088100	0	7.353253	4.593953	2.532926	L	
H-HC-0.162301	0	6.847306	4.170147	0.155786	H	
H-HC-0.188340	0	5.399121	4.448326	1.097331	H	
H-H-0.339448	0	7.160998	1.826806	-0.920690	H	
H-H4-0.185957	0	3.664079	2.010089	1.397523	H	
H-H5-0.211295	0	5.792139	-0.296935	-1.379254	H	
N-N--0.415700	0	5.399664	3.876292	3.999503	L	
C-CT-0.014300	-1	4.550702	3.579352	5.144060	L	
C-C-0.597300	0	5.198939	2.627727	6.160955	L	
O-O--0.567900	0	4.674608	1.574390	6.526342	L	
C-CT--0.204100	0	3.106989	3.293956	4.705371	L	
C-C-0.713000	0	2.158879	4.178298	5.493320	L	
N-N--0.919100	0	1.578334	3.704105	6.560949	L	
O-O--0.593100	0	1.960790	5.342066	5.189934	L	
H-H-0.271900	0	5.548067	4.865039	3.788534	L	
H-H1-0.104800	0	4.509115	4.531033	5.678840	L	
H-HC-0.079700	0	2.872950	2.245748	4.858911	L	
H-HC-0.079700	0	2.974925	3.528836	3.649549	L	
H-H-0.419600	0	0.911253	4.283713	7.022802	L	
H-H-0.419600	0	1.636837	2.696910	6.713019	L	
N-N--0.415700	0	6.384663	3.057818	6.605150	L	
C-CT--0.149000	-1	7.291416	2.309675	7.454178	L	
H-H-0.271900	0	6.698396	3.932362	6.207687	L	
H-H1-0.097600	0	8.044268	2.971988	7.881203	L	

```
H-H1-0.097600    0    7.780977    1.529915    6.867611 L
H-H1-0.097600    0    6.733046    1.825903    8.258815 L
C-CT--0.366200  -1   -2.264034   -2.756532    9.539986 L
C-C-0.597200     0   -2.387926   -3.844802    8.509148 L
O-O--0.567900    0   -2.930125   -4.907873    8.798463 L
H-HC-0.112300    0   -1.210110   -2.541089    9.704895 L
H-HC-0.112300    0   -2.723749   -3.090624   10.467740 L
H-HC-0.112300    0   -2.768758   -1.862824    9.177949 L
N-N--0.347900    0   -1.858745   -3.581962    7.317539 L
C-CT--0.240000  -1   -1.755715   -4.546997    6.216649 L
C-C-0.734100     0   -0.348556   -4.576498    5.632612 L
O-O--0.589400    0    0.463290   -3.689865    5.881019 L
C-CT--0.009400   0   -2.853213   -4.261764    5.172120 L
C-CT-0.018700    0   -2.637075   -2.972224    4.370545 L H-HC 379
C-CT--0.464524   0   -3.740202   -2.793128    3.321187 H
C-CT--0.195159   0   -3.628173   -1.492503    2.535891 H
N-N3--0.748751   0   -2.423604   -1.427401    1.646761 H
H-H-0.274700     0   -1.464574   -2.653392    7.182081 L
H-H1-0.142600    0   -1.938558   -5.543798    6.618774 L
H-HC-0.036200    0   -3.817734   -4.207524    5.679977 L
H-HC-0.036200    0   -2.903423   -5.090041    4.472544 L
H-HC-0.010300    0   -1.669130   -3.005433    3.870115 L
H-HC-0.010300    0   -2.663128   -2.125632    5.049353 L
H-HC-0.173903    0   -4.705728   -2.792961    3.835541 H
H-HC-0.148535    0   -3.733328   -3.657301    2.641635 H
H-HP-0.236066    0   -3.592137   -0.605571    3.168785 H
H-HP-0.172509    0   -4.490427   -1.389575    1.864751 H
H-H-0.437293     0   -2.264986   -0.388616    1.284630 H
H-H-0.366736     0   -2.572974   -2.037669    0.836493 H
H-H-0.419451     0   -1.527139   -1.684672    2.098440 H
N-N--0.415700    0   -0.048683   -5.613125    4.863007 L
C-CT--0.025200  -1    1.266711   -5.812424    4.263671 L
C-C-0.597300     0    1.146336   -6.527334    2.916774 L
O-O--0.567900    0    1.738513   -7.578731    2.683172 L
H-H-0.271900     0   -0.788747   -6.272628    4.641827 L
H-H1-0.069800    0    1.885451   -6.415289    4.928491 L
H-H1-0.069800    0    1.760402   -4.854343    4.097449 L
N-N--0.415700    0    0.329400   -5.941962    2.045999 L
C-CT--0.025200  -1   -0.240201   -6.534469    0.839152 L
C-C-0.597300     0   -1.373020   -5.641454    0.398335 L
O-O--0.567900    0   -2.257271   -5.371195    1.203187 L
H-H-0.271900     0   -0.118242   -5.099242    2.381219 L
H-H1-0.069800    0   -0.629644   -7.527558    1.063182 L
H-H1-0.069800    0    0.524269   -6.599077    0.064402 L
N-N--0.415700    0   -1.325520   -5.176880   -0.849217 L
C-CT--0.001400  -1   -2.161189   -4.094222   -1.369964 L
C-C-0.597300     0   -3.626652   -4.167191   -0.928852 L
O-O--0.567900    0   -4.191300   -3.194423   -0.439374 L
C-CT--0.015200   0   -2.063098   -4.069085   -2.909197 L
C-CA--0.001100   0   -0.671496   -4.240598   -3.493728 L
C-CA--0.190600   0    0.342057   -3.309334   -3.199724 L
C-CA--0.190600   0   -0.395606   -5.330197   -4.344203 L
C-CA--0.234100   0    1.629667   -3.467994   -3.745674 L
C-CA--0.234100   0    0.890223   -5.492301   -4.894492 L
```

```
C-C-0.322600      0    1.907026   -4.559597   -4.593726 L
O-OH--0.557900    0    3.143522   -4.714455   -5.136793 L
H-H-0.271900      0   -0.504278   -5.393497   -1.395871 L
H-H1-0.087600     0   -1.756597   -3.154212   -0.993555 L
H-HC-0.029500     0   -2.459136   -3.122539   -3.270064 L
H-HC-0.029500     0   -2.703975   -4.852559   -3.314450 L
H-HA-0.169900     0    0.137914   -2.463569   -2.555101 L
H-HA-0.169900     0   -1.178236   -6.032521   -4.591938 L
H-HA-0.165600     0    2.405295   -2.753109   -3.513054 L
H-HA-0.165600     0    1.093741   -6.320564   -5.553389 L
H-HO-0.399200     0    3.165776   -5.456340   -5.739109 L
N-N--0.415700     0   -4.225132   -5.349644   -1.087538 L
C-CT--0.024900   -1   -5.564137   -5.635121   -0.593945 L
C-C-0.597300      0   -5.695132   -5.558294    0.925125 L
O-O--0.567900     0   -5.060750   -6.317851    1.666440 L
C-CT-0.211700     0   -6.027909   -7.028617   -1.022381 L
O-OH--0.654600   -1   -7.286275   -7.334012   -0.436971 L
H-H-0.271900      0   -3.647966   -6.093372   -1.436749 L
H-H1-0.084300     0   -6.248033   -4.910104   -1.035749 L
H-H1-0.035200     0   -5.296074   -7.767405   -0.693595 L
H-H1-0.035200     0   -6.104768   -7.067338   -2.108981 L
H-HO-0.427500    -1   -7.544342   -8.205651   -0.746717 L
N-N--0.415700     0   -6.667305   -4.757121    1.353442 L
C-CT-0.033700    -1   -7.164338   -4.665232    2.714551 L
C-C-0.597300      0   -7.517636   -5.994058    3.387642 L
O-O--0.567900     0   -7.446532   -6.102647    4.609926 L
C-CT--0.182500    0   -8.357750   -3.709534    2.716951 L
H-H-0.271900      0   -7.125775   -4.175344    0.658813 L
H-H1-0.082300     0   -6.379508   -4.216178    3.321888 L
H-HC-0.060300     0   -8.068899   -2.741709    2.302942 L
H-HC-0.060300     0   -8.705545   -3.571516    3.736060 L
H-HC-0.060300     0   -9.169267   -4.132582    2.130553 L
N-N--0.415700     0   -7.911441   -6.997374    2.602639 L
C-CT--0.025200   -1   -8.293671   -8.310419    3.107616 L
C-C-0.597300      0   -7.334557   -9.438097    2.748652 L
O-O--0.567900     0   -7.749790  -10.592560    2.674599 L
H-H-0.271900      0   -7.897889   -6.828080    1.603285 L
H-H1-0.069800     0   -9.271050   -8.564814    2.698719 L
H-H1-0.069800     0   -8.390185   -8.291506    4.193431 L
N-N--0.415700     0   -6.051521   -9.127253    2.569775 L
C-CT--0.024900   -1   -4.978526  -10.121310    2.584762 L
C-C-0.597300      0   -4.280221  -10.087709    3.938303 L
O-O--0.567900     0   -3.958738   -9.013568    4.455970 L
C-CT-0.211700     0   -3.984417   -9.827740    1.456493 L
O-OH--0.654600   -1   -3.099181  -10.920715    1.267176 L
H-H-0.271900      0   -5.797869   -8.146060    2.638413 L
H-H1-0.084300     0   -5.390912  -11.116366    2.417232 L
H-H1-0.035200     0   -3.419570   -8.922145    1.684282 L
H-H1-0.035200     0   -4.540487   -9.668633    0.531786 L
H-HO-0.427500    -1   -2.557598  -10.731141    0.497501 L
N-N--0.415700     0   -4.082933  -11.266398    4.527878 L
C-CT--0.149000   -1   -3.546904  -11.416165    5.869639 L
H-H-0.271900      0   -4.363054  -12.084415    4.010510 L
H-H1-0.097600     0   -3.342179  -12.465637    6.081377 L
```

```
H-H1-0.097600     0   -4.262829  -11.033533    6.600416 L
H-H1-0.097600     0   -2.620661  -10.844615    5.966489 L
C-CT--0.366200  -1  -12.135325    5.102405   -0.642983 L
C-C-0.597200      0  -10.706347    5.279910   -0.202022 L
O-O--0.567900     0  -10.389465    5.000057    0.948573 L
H-HC-0.112300     0  -12.528559    6.058225   -0.982265 L
H-HC-0.112300     0  -12.721574    4.743824    0.201550 L
H-HC-0.112300     0  -12.170905    4.368687   -1.446185 L
N-N--0.415700     0   -9.856398    5.733079   -1.119159 L
C-CT--0.025200  -1   -8.422577    5.890340   -0.889834 L
C-C-0.597300      0   -7.606890    5.781563   -2.154190 L
O-O--0.567900     0   -8.108147    6.096446   -3.239706 L
H-H-0.271900      0  -10.170979    5.906043   -2.061609 L
H-H1-0.069800     0   -8.112590    5.097470   -0.219355 L
H-H1-0.069800     0   -8.219046    6.851823   -0.420535 L
N-N--0.415700     0   -6.353973    5.354944   -1.981876 L H-H1 489
C-CT--0.530209  -1   -5.336261    5.317391   -3.032731 H
C-C-0.568093      0   -4.558109    4.017169   -2.929278 H
O-O--0.528105     0   -4.436960    3.427293   -1.844106 H
C-CT-0.298500     0   -4.415968    6.559244   -2.961243 L H-H1 489
C-CT--0.319200    0   -5.186886    7.833394   -3.311587 L
C-CT--0.319200    0   -3.751976    6.733529   -1.592266 L
H-H-0.271900      0   -6.069316    5.062378   -1.048669 L
H-H1-0.184096     0   -5.854799    5.363994   -3.996689 H
H-HC--0.029700    0   -3.628255    6.446814   -3.705171 L
H-HC-0.079100     0   -5.930540    8.044216   -2.544758 L
H-HC-0.079100     0   -5.685784    7.705351   -4.271168 L
H-HC-0.079100     0   -4.492667    8.670251   -3.376691 L
H-HC-0.079100     0   -4.502116    6.866044   -0.814082 L
H-HC-0.079100     0   -3.098315    7.603778   -1.607587 L
H-HC-0.079100     0   -3.155455    5.853168   -1.353101 L
N-N--0.610103     0   -3.965998    3.569375   -4.055570 H
C-CT--0.314060  -1   -4.223068    4.030014   -5.411270 H
C-C-0.597300      0   -5.345967    3.217593   -6.039578 L H-H1 505
O-O--0.567900     0   -5.479723    2.015880   -5.809818 L
C-CT--0.110200    0   -2.931776    3.897698   -6.254635 L H-H1 505
C-CT-0.353100     0   -1.837375    4.951571   -5.961734 L
C-CT--0.412100    0   -1.067557    4.727118   -4.656738 L
C-CT--0.412100    0   -0.807651    4.940822   -7.095350 L
H-H-0.398207      0   -3.491124    2.669464   -3.975770 H
H-H1-0.165130     0   -4.509895    5.080137   -5.410760 H
H-HC-0.045700     0   -3.216255    4.003619   -7.301853 L
H-HC-0.045700     0   -2.514159    2.898308   -6.133068 L
H-HC--0.036100    0   -2.295103    5.941021   -5.936626 L
H-HC-0.100000     0   -0.666607    3.715639   -4.629654 L
H-HC-0.100000     0   -1.733737    4.866320   -3.807262 L
H-HC-0.100000     0   -0.256916    5.449293   -4.571094 L
H-HC-0.100000     0   -0.318119    3.969079   -7.140611 L
H-HC-0.100000     0   -0.058721    5.712541   -6.917464 L
H-HC-0.100000     0   -1.300098    5.145112   -8.044721 L
N-N--0.415700     0   -6.127655    3.866541   -6.897500 L
C-CT--0.038900  -1   -7.313015    3.269964   -7.525206 L
C-C-0.597300      0   -7.061544    1.932933   -8.210897 L
O-O--0.567900     0   -7.930468    1.064128   -8.229863 L
```

```
C-CT-0.365400   -1   -7.948238    4.198002   -8.585019 L
O-OH--0.676100  -1   -7.885596    5.553889   -8.133248 L
C-CT--0.243800   0   -9.425329    3.904813   -8.845919 L
H-H-0.271900     0   -6.000268    4.863190   -6.994694 L
H-H1-0.100700    0   -8.056075    3.127040   -6.747398 L
H-H1-0.004300    0   -7.391911    4.123625   -9.520385 L
H-HC-0.064200    0   -9.538106    2.898952   -9.250422 L
H-HC-0.064200    0   -9.818967    4.615413   -9.572568 L
H-HC-0.064200    0   -9.991531    3.978712   -7.916642 L
H-HO-0.410200   -1   -8.783480    5.889934   -8.097765 L
N-N--0.415700    0   -5.891701    1.805753   -8.834263 L
C-CT--0.025200  -1   -5.506514    0.644633   -9.624197 L
C-C-0.597300     0   -4.602590   -0.352296   -8.931987 L
O-O--0.567900    0   -4.112944   -1.259809   -9.604239 L
H-H-0.271900     0   -5.215067    2.536769   -8.677115 L
H-H1-0.069800    0   -4.985156    0.987888  -10.516656 L
H-H1-0.069800    0   -6.395500    0.105140   -9.951205 L
N-N--0.415700    0   -4.374259   -0.167082   -7.626591 L H-H1 545
C-CT--0.539097  -1   -3.467914   -0.976346   -6.822449 H
C-C-0.572106     0   -4.225496   -1.434822   -5.573939 H
O-O--0.533001    0   -4.945849   -2.427582   -5.662942 H
C-CT-0.130300    0   -2.080756   -0.306634   -6.664327 L H-H1 545
C-CT--0.043000   0   -1.441505    0.060148   -8.024756 L
C-CT--0.320400   0   -1.183750   -1.297231   -5.900753 L
C-CT--0.066000   0   -0.078679    0.757587   -7.920373 L
H-H-0.271900     0   -4.841629    0.601762   -7.155586 L
H-H1-0.180646    0   -3.300164   -1.904623   -7.380851 H
H-HC-0.018700    0   -2.194441    0.608548   -6.095116 L
H-HC-0.023600    0   -2.101711    0.748756   -8.550408 L
H-HC-0.023600    0   -1.335161   -0.840009   -8.631514 L
H-HC-0.088200    0   -1.094506   -2.229041   -6.458063 L
H-HC-0.088200    0   -1.604789   -1.511581   -4.920531 L
H-HC-0.088200    0   -0.192915   -0.883148   -5.737837 L
H-HC-0.018600    0   -0.148490    1.604411   -7.238850 L
H-HC-0.018600    0    0.217485    1.117397   -8.905790 L
H-HC-0.018600    0    0.680688    0.062617   -7.564757 L
N-N--0.611145    0   -4.104894   -0.678685   -4.457880 H
C-CT--0.109296  -1   -4.635986   -1.086830   -3.162138 H
C-C-0.597300     0   -6.163447   -1.076660   -3.127624 L H-H1 564
O-O--0.567900    0   -6.797122   -1.983003   -2.577185 L
C-CT--0.388038   0   -4.017625   -0.239412   -2.061219 H
H-H-0.369964     0   -3.437780    0.089511   -4.425087 H
H-H1-0.178541    0   -4.380538   -2.134656   -2.977470 H
H-H1-0.157964    0   -4.460929   -0.539854   -1.105587 H
H-H1-0.179833    0   -4.219735    0.826617   -2.190732 H
H-HS-0.319500    0   -1.680292    1.913874   -0.504017 H
N-N--0.415700    0   -6.753488   -0.097554   -3.803789 L
C-CT--0.087500  -1   -8.190818   -0.040614   -4.058805 L
C-C-0.597300     0   -8.665721   -1.195897   -4.925368 L
O-O--0.567900    0   -9.591274   -1.909209   -4.540721 L
C-CT-0.298500    0   -8.592905    1.320534   -4.644670 L
C-CT--0.319200   0  -10.107887    1.437809   -4.859443 L
C-CT--0.319200   0   -8.166419    2.492574   -3.746197 L
H-H-0.271900     0   -6.115429    0.554854   -4.251744 L
```

H-H1-0.096900	0	-8.699929	-0.142635	-3.102429	L
H-HC--0.029700	0	-8.102269	1.416731	-5.604243	L
H-HC-0.079100	0	-10.629635	1.279401	-3.915272	L
H-HC-0.079100	0	-10.442771	0.697380	-5.585781	L
H-HC-0.079100	0	-10.349398	2.427905	-5.245083	L
H-HC-0.079100	0	-8.633035	2.411885	-2.765967	L
H-HC-0.079100	0	-8.449512	3.440540	-4.202994	L
H-HC-0.079100	0	-7.083820	2.501617	-3.614908	L
N-N--0.415700	0	-8.042321	-1.392304	-6.085608	L
C-CT--0.025200	-1	-8.400477	-2.449336	-7.028239	L
C-C-0.597300	0	-8.317206	-3.852134	-6.439185	L
O-O--0.567900	0	-9.220759	-4.662599	-6.631052	L
H-H-0.271900	0	-7.263843	-0.784054	-6.303676	L
H-H1-0.069800	0	-7.730604	-2.400006	-7.886432	L
H-H1-0.069800	0	-9.419699	-2.284740	-7.377412	L
N-N--0.415700	0	-7.233941	-4.132651	-5.717464	L
C-CT--0.025200	-1	-7.011625	-5.398857	-5.030774	L
C-C-0.597300	0	-7.936367	-5.658351	-3.852494	L
O-O--0.567900	0	-8.258654	-6.811301	-3.572290	L
H-H-0.271900	0	-6.515053	-3.413938	-5.655360	L
H-H1-0.069800	0	-5.985852	-5.421865	-4.664659	L
H-H1-0.069800	0	-7.137971	-6.214514	-5.742949	L
N-N--0.415700	0	-8.331772	-4.600370	-3.147377	L
C-CT--0.038900	-1	-9.384056	-4.666340	-2.130597	L
C-C-0.597300	0	-10.752900	-4.959112	-2.736203	L
O-O--0.567900	0	-11.496666	-5.795546	-2.224034	L
C-CT-0.365400	0	-9.428893	-3.370682	-1.306293	L
O-OH--0.676100	0	-8.196703	-3.204792	-0.643152	L
C-CT--0.243800	0	-10.519224	-3.391610	-0.235353	L
H-H-0.271900	0	-7.961422	-3.692792	-3.404854	L
H-H1-0.100700	0	-9.156184	-5.485171	-1.448171	L
H-H1-0.004300	0	-9.595162	-2.515239	-1.961050	L
H-HC-0.064200	0	-11.498246	-3.284454	-0.701170	L
H-HC-0.064200	0	-10.369297	-2.565662	0.459410	L
H-HC-0.064200	0	-10.485612	-4.334693	0.308481	L
H-HO-0.410200	0	-7.608612	-2.729633	-1.266024	L
N-N--0.415700	0	-11.100910	-4.249084	-3.810421	L
C-CT--0.149000	-1	-12.333899	-4.434519	-4.547012	L
H-H-0.271900	0	-10.431893	-3.567465	-4.157021	L
H-H1-0.097600	0	-12.404081	-3.684579	-5.335808	L
H-H1-0.097600	0	-13.186020	-4.340779	-3.872397	L
H-H1-0.097600	0	-12.339063	-5.429058	-4.996330	L
N-N2--0.511415	0	1.988191	-0.030976	-1.720870	H
C-CA-0.057801	0	0.684063	0.261095	-1.698788	H
C-CA-0.063496	0	0.000998	0.762631	-2.811162	H
C-CA--0.109122	0	0.726858	1.034053	-3.967987	H
C-CA--0.164490	0	2.090180	0.755942	-3.984704	H
C-CA-0.098392	0	2.674155	0.200564	-2.851703	H
C-C-0.541263	0	-1.498906	0.933814	-2.825333	H
O-O--0.574884	0	-2.070546	1.397477	-3.834102	H
N-N--0.751108	0	-1.649515	2.351432	-1.447048	H
H-H4-0.220889	0	0.170786	0.094459	-0.757426	H
H-HA-0.160434	0	0.207151	1.437019	-4.830009	H
H-HA-0.142191	0	2.696683	0.958676	-4.861342	H

```
H-H4-0.174502    0     3.722675   -0.073733   -2.836224 H
H-H-0.412468     0    -2.561832    2.788335   -1.622681 H
H-H-0.353801     0    -0.921576    3.069750   -1.483772 H
Zn-ZN-0.974537   0     2.856860   -1.091762    0.001961 H
O-OW--0.834000   0    -2.141489   -7.242324    3.473537 L
H-HW-0.417000    0    -2.596197   -6.653663    2.851599 L
H-HW-0.417000    0    -2.853798   -7.801950    3.824887 L
O-OW--0.783903   0     1.316131   -2.551677   -0.133583 H
H-HW-0.401253    0     0.942039   -2.452246    0.780389 H
H-HW-0.424111    0     1.924468   -3.332697   -0.189362 H
O-OW--0.834000   0    -3.643386   -8.445441    7.199944 L
H-HW-0.417000    0    -3.733976   -7.505443    7.375872 L
H-HW-0.417000    0    -3.781519   -8.520625    6.243785 L
O-OW--0.834000   0     7.213358   -0.018694    1.839231 L
H-HW-0.417000    0     6.900943    0.358450    2.680766 L
H-HW-0.417000    0     6.435266    0.108725    1.274434 L
O-OW--0.834000   0     7.045764   -4.957322    4.761231 L
H-HW-0.417000    0     6.096622   -5.026262    4.991302 L
H-HW-0.417000    0     7.265258   -5.841744    4.465294 L
O-OW--0.834000   0     8.575994   -0.947966   -0.280530 L
H-HW-0.417000    0     8.161573   -0.567468    0.519692 L
H-HW-0.417000    0     8.832964   -0.160604   -0.781242 L
O-OW--0.834000   0    12.614032   -1.069243   -1.904681 L
H-HW-0.417000    0    11.771508   -0.822622   -2.323263 L
H-HW-0.417000    0    12.637963   -2.029322   -2.037923 L
O-OW--0.834000   0     8.260911   -3.313119    0.981844 L
H-HW-0.417000    0     8.449716   -2.550852    0.396178 L
H-HW-0.417000    0     7.555769   -2.969344    1.539856 L
O-OW--0.834000   0     4.355655   -4.922237    5.165233 L
H-HW-0.417000    0     4.086668   -4.542897    6.024739 L
H-HW-0.417000    0     4.043274   -4.257447    4.541306 L
S-SH--0.326142   0    -2.214693   -0.529911   -1.926341 H
```

IC2

```
C-CT--0.366200  -1   -10.305405    3.925576    4.025304 L
C-C-0.597200     0    -8.964995    3.289585    3.787734 L
O-O--0.567900    0    -8.386032    2.723173    4.708026 L
H-HC-0.112300    0   -11.039761    3.456079    3.373649 L
H-HC-0.112300    0   -10.238509    4.988521    3.800312 L
H-HC-0.112300    0   -10.588377    3.782881    5.065896 L
N-N--0.415700    0    -8.496667    3.386599    2.550334 L
C-CT--0.059700  -1    -7.336796    2.659152    2.027200 L
C-C-0.597300     0    -6.175434    3.628168    1.833112 L
O-O--0.567900    0    -6.283009    4.583229    1.067865 L
C-CT-0.130300    0    -7.717904    1.928897    0.713063 L
C-CT--0.043000   0    -8.898851    0.949547    0.933902 L
C-CT--0.320400   0    -6.510752    1.163253    0.147634 L
C-CT--0.066000   0    -9.465093    0.346360   -0.357190 L
H-H-0.271900     0    -9.042633    3.940027    1.898250 L
H-H1-0.086900    0    -7.029690    1.901328    2.747543 L
H-HC-0.018700    0    -8.022392    2.677081   -0.021041 L
H-HC-0.023600    0    -9.723418    1.471832    1.417447 L
H-HC-0.023600    0    -8.582803    0.140408    1.593489 L
H-HC-0.088200    0    -6.175932    0.397653    0.848074 L
```

H-HC-0.088200	0	-5.682748	1.846082	-0.043650	L	
H-HC-0.088200	0	-6.757766	0.699928	-0.805063	L	
H-HC-0.018600	0	-9.708220	1.139441	-1.063601	L	
H-HC-0.018600	0	-10.374802	-0.206615	-0.125741	L	
H-HC-0.018600	0	-8.746192	-0.339797	-0.802234	L	
N-N--0.516300	0	-5.090623	3.388148	2.565489	L	
C-CT-0.038100	-1	-3.768263	3.984020	2.402454	L	H-HC 30
C-C-0.536600	0	-3.683552	5.501566	2.150717	L	
O-O--0.581900	0	-2.710804	6.010880	1.592005	L	
C-CT--0.547145	0	-3.068583	3.169141	1.315437	H	
C-C-0.532653	0	-2.891838	1.729682	1.719943	H	
O-O2--0.588507	0	-2.392087	1.405385	2.790091	H	
O-O2--0.625559	0	-3.334091	0.886357	0.806354	H	
H-H-0.293600	0	-5.109892	2.538302	3.114850	L	
H-H1-0.088000	0	-3.219949	3.816581	3.330566	L	
H-HC-0.128435	0	-2.067315	3.595788	1.180088	H	
H-HC-0.183556	0	-3.613072	3.274425	0.375575	H	
N-N--0.415700	0	-4.650266	6.258149	2.653587	L	
C-CT--0.025200	-1	-4.636754	7.703790	2.540111	L	
C-C-0.597300	0	-3.740821	8.337247	3.589077	L	
O-O--0.567900	0	-4.217607	8.983212	4.522436	L	
H-H-0.271900	0	-5.457589	5.761393	2.996598	L	
H-H1-0.069800	0	-5.649240	8.081710	2.675228	L	
H-H1-0.069800	0	-4.290397	7.999224	1.548612	L	
N-N--0.415700	0	-2.437205	8.133603	3.435077	L	
C-CT--0.038900	-1	-1.415407	8.551122	4.389768	L	
C-C-0.597300	0	-0.505368	9.637627	3.807617	L	
O-O--0.567900	0	-0.597786	10.009881	2.636376	L	
C-CT-0.365400	0	-0.554524	7.375641	4.889899	L	
O-OH--0.676100	0	0.478341	7.109156	3.977497	L	
C-CT--0.243800	0	-1.326833	6.086692	5.154043	L	
H-H-0.271900	0	-2.164571	7.552793	2.645485	L	
H-H1-0.100700	0	-1.909198	8.977882	5.261182	L	
H-H1-0.004300	0	-0.090460	7.681223	5.828252	L	
H-HC-0.064200	0	-2.181561	6.293460	5.796971	L	
H-HC-0.064200	0	-0.670909	5.373714	5.647337	L	
H-HC-0.064200	0	-1.668869	5.652084	4.215024	L	
H-HO-0.410200	0	1.029658	6.388866	4.352215	L	
N-N--0.415700	0	0.414755	10.138836	4.635976	L	
C-CT--0.025200	-1	1.458054	11.072769	4.220183	L	
C-C-0.597300	0	2.329352	10.585337	3.070051	L	
O-O--0.567900	0	2.596993	11.338827	2.139335	L	
H-H-0.271900	0	0.457454	9.759681	5.566800	L	
H-H1-0.069800	0	2.111407	11.285719	5.065316	L	
H-H1-0.069800	0	0.990416	12.006803	3.907715	L	
N-N--0.516300	0	2.766073	9.331588	3.147910	L	
C-CT-0.038100	-1	3.662733	8.720305	2.168695	L	
C-C-0.536600	0	3.025207	8.399238	0.828794	L	
O-O--0.581900	0	3.748876	8.045328	-0.096313	L	
C-CT--0.030300	0	4.234860	7.407937	2.736001	L	
C-C-0.799400	-1	5.588757	7.598372	3.395612	L	
O-O2--0.801400	-1	6.171526	8.701642	3.319343	L	
O-O2--0.801400	-1	6.045442	6.641405	4.053543	L	
H-H-0.293600	0	2.460678	8.750343	3.912732	L	

H-H1-0.088000	0	4.469234	9.418303	1.940955	L
H-HC--0.012200	0	4.372675	6.672837	1.941323	L
H-HC--0.012200	0	3.533330	6.974679	3.451698	L
N-N--0.415700	0	1.706845	8.514912	0.707111	L
C-CT--0.002400	-1	0.996739	8.326351	-0.550800	L
C-C-0.597300	0	0.395744	9.620434	-1.097219	L
O-O--0.567900	0	0.333404	9.808303	-2.312243	L
C-CT--0.034300	0	-0.068445	7.236527	-0.388834	L
C-CA-0.011800	0	0.476616	5.854120	-0.072125	L
C-CA--0.125600	0	1.145540	5.108784	-1.061906	L
C-CA--0.125600	0	0.267136	5.284628	1.196642	L
C-CA--0.170400	0	1.564795	3.793862	-0.785686	L
C-CA--0.170400	0	0.686337	3.973339	1.473018	L
C-CA--0.107200	0	1.323050	3.223799	0.473549	L
H-H-0.271900	0	1.190105	8.755900	1.540905	L
H-H1-0.097800	0	1.691093	7.977917	-1.315053	L
H-HC-0.029500	0	-0.628563	7.164233	-1.321584	L
H-HC-0.029500	0	-0.770522	7.540378	0.389236	L
H-HA-0.133000	0	1.309036	5.534784	-2.040917	L
H-HA-0.133000	0	-0.255448	5.842826	1.955437	L
H-HA-0.143000	0	2.030594	3.186596	-1.544394	L
H-HA-0.143000	0	0.484110	3.532476	2.439473	L
H-HA-0.129700	0	1.605106	2.199756	0.661704	L
N-N--0.415700	0	-0.024852	10.527452	-0.214056	L
C-CT--0.149000	-1	-0.444194	11.863567	-0.581974	L
H-H-0.271900	0	0.016378	10.285248	0.770604	L
H-H1-0.097600	0	-0.800732	12.390670	0.303636	L
H-H1-0.097600	0	-1.245675	11.806889	-1.320421	L
H-H1-0.097600	0	0.400677	12.404965	-1.010388	L
C-CT--0.366200	-1	8.006299	8.424913	-0.104439	L
C-C-0.597200	0	7.537529	7.655042	-1.314409	L
O-O--0.567900	0	8.351857	7.352835	-2.183021	L
H-HC-0.112300	0	7.531406	9.403460	-0.089710	L
H-HC-0.112300	0	9.087521	8.535861	-0.146014	L
H-HC-0.112300	0	7.736811	7.877103	0.799375	L
N-N--0.415700	0	6.230161	7.384896	-1.399059	L
C-CT--0.051800	-1	5.585716	6.863776	-2.614128	L
C-C-0.597300	0	4.851732	7.933562	-3.419227	L
O-O--0.567900	0	4.990283	8.022084	-4.638815	L
C-CT--0.110200	0	4.754342	5.592456	-2.352728	L
C-CT-0.353100	0	5.172240	4.438719	-3.291582	L
C-CT--0.412100	0	4.326510	3.200196	-3.031962	L
C-CT--0.412100	0	5.005308	4.752840	-4.783026	L
H-H-0.271900	0	5.630609	7.658353	-0.628016	L
H-H1-0.092200	0	6.400987	6.563206	-3.271289	L
H-HC-0.045700	0	3.695508	5.812718	-2.487907	L
H-HC-0.045700	0	4.899430	5.267614	-1.321854	L
H-HC--0.036100	0	6.215454	4.184710	-3.099742	L
H-HC-0.100000	0	3.304292	3.377532	-3.362728	L
H-HC-0.100000	0	4.327402	2.969350	-1.968838	L
H-HC-0.100000	0	4.741596	2.352277	-3.573493	L
H-HC-0.100000	0	3.995202	5.114800	-4.976110	L
H-HC-0.100000	0	5.188814	3.859992	-5.377898	L
H-HC-0.100000	0	5.717749	5.516667	-5.090816	L

```
N-N--0.415700     0    4.093523    8.761897   -2.716212 L
C-CT--0.149000  -1    3.385937    9.901161   -3.233593 L
H-H-0.271900      0    4.040989    8.568357   -1.723842 L
H-H1-0.097600     0    4.046713   10.491939   -3.869280 L
H-H1-0.097600     0    3.026114   10.518708   -2.409286 L
H-H1-0.097600     0    2.530823    9.564706   -3.822631 L
C-CT--0.366200  -1   -8.729794    2.361698    8.129869 L
C-C-0.597200      0   -7.857060    1.151705    7.952033 L
O-O--0.567900     0   -7.535047    0.470592    8.918536 L
H-HC-0.112300     0   -9.651123    2.218384    7.568648 L
H-HC-0.112300     0   -8.202511    3.237728    7.755365 L
H-HC-0.112300     0   -8.953284    2.486979    9.187278 L
N-N--0.415700     0   -7.467976    0.901385    6.710613 L
C-CT--0.038900  -1   -6.670159   -0.252699    6.291111 L
C-C-0.597300      0   -5.404544    0.246305    5.617400 L
O-O--0.567900     0   -5.444387    0.712841    4.478772 L
C-CT-0.365400     0   -7.485066   -1.199511    5.396438 L
O-OH--0.676100    0   -8.021538   -0.517887    4.288356 L
C-CT--0.243800    0   -8.650036   -1.812147    6.179468 L
H-H-0.271900      0   -7.798750    1.525478    5.978461 L
H-H1-0.100700     0   -6.373911   -0.826565    7.168994 L
H-H1-0.004300     0   -6.838148   -2.001771    5.043567 L
H-HC-0.064200     0   -8.267336   -2.360869    7.039682 L
H-HC-0.064200     0   -9.209813   -2.493928    5.545223 L
H-HC-0.064200     0   -9.328571   -1.030690    6.519816 L
H-HO-0.410200     0   -7.324701    0.069441    3.962013 L
N-N--0.415700     0   -4.309909    0.218244    6.380319 L
C-CT--0.059700  -1   -3.114093    1.035846    6.167118 L
C-C-0.597300      0   -1.867272    0.156801    6.097994 L
O-O--0.567900     0   -1.612670   -0.644959    7.000993 L
C-CT-0.130300     0   -2.993649    2.110642    7.273510 L
C-CT--0.043000    0   -4.245081    3.011061    7.423522 L
C-CT--0.320400    0   -1.746647    2.979601    7.049980 L
C-CT--0.066000    0   -4.648342    3.813988    6.178045 L
H-H-0.271900      0   -4.372312   -0.241792    7.274334 L
H-H1-0.086900     0   -3.205775    1.550871    5.209751 L
H-HC-0.018700     0   -2.861423    1.595597    8.226962 L
H-HC-0.023600     0   -4.068266    3.715577    8.237016 L
H-HC-0.023600     0   -5.092869    2.395783    7.723516 L
H-HC-0.088200     0   -1.728986    3.342308    6.022155 L
H-HC-0.088200     0   -0.850126    2.383164    7.222218 L
H-HC-0.088200     0   -1.738482    3.820929    7.741845 L
H-HC-0.018600     0   -3.840126    4.475992    5.871420 L
H-HC-0.018600     0   -5.527174    4.416677    6.407641 L
H-HC-0.018600     0   -4.895841    3.143408    5.358716 L
N-N--0.516300     0   -1.112883    0.336491    5.018113 L
C-CT-0.038100    -1    0.181804   -0.251190    4.732301 L H-HC 17
C-C-0.536600      0    1.122205   -0.095370    5.933824 L
O-O--0.581900     0    1.334264    0.996784    6.456650 L
C-CT--0.520019    0    0.687281    0.381790    3.420098 H
C-C-0.636404      0    0.993096   -0.593316    2.289679 H
O-O2--0.608036    0    0.429358   -1.713282    2.220538 H
O-O2--0.608131    0    1.824486   -0.122561    1.429331 H
H-H-0.293600      0   -1.500709    0.922263    4.285254 L
```

```
H-H1-0.088000    0    0.019417   -1.318831    4.575548 L
H-HC-0.132300    0    1.591696    0.961383    3.622577 H
H-HC-0.188993    0   -0.054746    1.088828    3.035066 H
N-N--0.415700    0    1.667096   -1.208340    6.391232 L
C-CT-0.033700   -1    2.558990   -1.270080    7.526303 L
C-C-0.597300     0    3.779068   -2.091060    7.124719 L
O-O--0.567900    0    3.825655   -3.317139    7.274905 L
C-CT--0.182500   0    1.791988   -1.840389    8.727684 L
H-H-0.271900     0    1.360918   -2.079689    5.972260 L
H-H1-0.082300    0    2.904667   -0.268602    7.789881 L
H-HC-0.060300    0    0.953524   -1.184860    8.966317 L
H-HC-0.060300    0    2.456720   -1.905020    9.588835 L
H-HC-0.060300    0    1.412208   -2.835053    8.488488 L
N-N--0.415700    0    4.751567   -1.395434    6.547829 L
C-CT-0.018800   -1    5.955868   -1.978255    6.000310 L H-HC 20
C-C-0.597300     0    7.152861   -1.833900    6.949239 L
O-O--0.567900    0    7.203361   -0.980890    7.837731 L
C-CT--0.517456   0    6.212120   -1.338698    4.633954 H
C-CM-0.287351    0    5.352524   -1.824529    3.525520 H
N-N2--0.586994   0    5.511966   -3.075436    2.944465 H
C-CM--0.020338   0    4.373087   -1.222625    2.786869 H
C-CM-0.242458    0    4.651878   -3.170426    1.895465 H
N-N2--0.505070   0    3.954632   -2.056911    1.771001 H
H-H-0.271900     0    4.648523   -0.385854    6.494144 L
H-H1-0.088100    0    5.807868   -3.044015    5.856704 L
H-HC-0.166355    0    7.270081   -1.495766    4.376682 H
H-HC-0.174481    0    6.088091   -0.261176    4.768461 H
H-H-0.335795     0    6.156987   -3.791599    3.271557 H
H-H4-0.152777    0    3.956820   -0.234479    2.911193 H
H-H5-0.209475    0    4.562144   -4.035384    1.253148 H
N-N--0.415700    0    8.153422   -2.678347    6.705867 L
C-CT--0.149000  -1    9.423159   -2.610936    7.396024 L
H-H-0.271900     0    7.999259   -3.358332    5.967960 L
H-H1-0.097600    0   10.092956   -3.384127    7.018428 L
H-H1-0.097600    0    9.265318   -2.755778    8.466187 L
H-H1-0.097600    0    9.874326   -1.629949    7.236276 L
C-CT--0.366200  -1    8.344289   -9.401325    0.906241 L
C-C-0.597200     0    8.719937   -9.158546   -0.543044 L
O-O--0.567900    0    9.744026   -8.536667   -0.798329 L
H-HC-0.112300    0    8.276653  -10.469799    1.096960 L
H-HC-0.112300    0    7.399032   -8.908121    1.125304 L
H-HC-0.112300    0    9.118077   -8.968098    1.539923 L
N-N--0.254800    0    7.907914   -9.620914   -1.510209 L
C-CT--0.026600  -1    8.152135   -9.376988   -2.934042 L
C-C-0.589600     0    8.003545   -7.902648   -3.310405 L
O-O--0.574800    0    8.715495   -7.403652   -4.178911 L
C-CT--0.007000   0    7.147657  -10.268373   -3.672089 L
C-CT-0.018900    0    5.988822  -10.405042   -2.683299 L
C-CT-0.019200    0    6.682265  -10.385075   -1.320349 L
H-H1-0.064100    0    9.165242   -9.686194   -3.194342 L
H-HC-0.025300    0    7.594592  -11.249036   -3.842222 L
H-HC-0.025300    0    6.824523   -9.832085   -4.618567 L
H-HC-0.021300    0    5.430914  -11.328956   -2.838554 L
H-HC-0.021300    0    5.327498   -9.541523   -2.770450 L
```

H-H1-0.039100	0	6.031630	-9.923226	-0.576385	L	
H-H1-0.039100	0	6.934749	-11.402917	-1.020078	L	
N-N--0.516300	0	7.081694	-7.207222	-2.643499	L	
C-CT-0.039700	-1	6.834602	-5.778804	-2.810486	L	
C-C-0.536600	0	8.096628	-4.918628	-2.673756	L	
O-O--0.581900	0	8.274746	-3.930607	-3.389883	L	
C-CT-0.056000	0	5.732896	-5.339792	-1.840203	L	H-HC 24
C-CT--0.515956	0	5.213839	-3.947294	-2.211821	H	
C-C-0.587828	0	4.115278	-3.409807	-1.301277	H	
O-O2--0.600319	0	4.048970	-2.128062	-1.277144	H	
O-O2--0.589127	0	3.363403	-4.199417	-0.685631	H	
H-H-0.293600	0	6.547673	-7.685544	-1.937196	L	
H-H1-0.110500	0	6.464383	-5.620647	-3.823792	L	
H-HC--0.017300	0	6.120950	-5.330648	-0.820485	L	
H-HC--0.017300	0	4.905541	-6.048989	-1.898417	L	
H-HC-0.167759	0	4.821812	-3.985324	-3.235350	H	
H-HC-0.135230	0	6.042041	-3.229775	-2.210821	H	
N-N--0.415700	0	9.004767	-5.330925	-1.791832	L	
C-CT--0.024900	-1	10.243026	-4.630670	-1.471394	L	
C-C-0.597300	0	11.263984	-4.511535	-2.595912	L	
O-O--0.567900	0	12.239493	-3.772761	-2.460625	L	
C-CT-0.211700	0	10.884992	-5.273580	-0.235850	L	
O-OH--0.654600	0	9.930195	-5.468685	0.796980	L	
H-H-0.271900	0	8.795887	-6.145233	-1.230590	L	
H-H1-0.084300	0	9.975440	-3.608752	-1.198543	L	
H-H1-0.035200	0	11.684828	-4.627758	0.129404	L	
H-H1-0.035200	0	11.310711	-6.240227	-0.509245	L	
H-HO-0.427500	0	9.370982	-4.663872	0.873957	L	
N-N--0.415700	0	11.028727	-5.195408	-3.713858	L	
C-CT-0.033700	-1	11.780613	-5.028549	-4.950654	L	
C-C-0.597300	0	10.960595	-4.442872	-6.104086	L	
O-O--0.567900	0	11.406647	-4.425942	-7.250463	L	
C-CT--0.182500	0	12.382597	-6.396409	-5.303783	L	
H-H-0.271900	0	10.217628	-5.803484	-3.723088	L	
H-H1-0.082300	0	12.609406	-4.338430	-4.786981	L	
H-HC-0.060300	0	12.992982	-6.756867	-4.475027	L	
H-HC-0.060300	0	13.010231	-6.304693	-6.190903	L	
H-HC-0.060300	0	11.583400	-7.112159	-5.503148	L	
N-N--0.415700	0	9.775612	-3.936913	-5.780868	L	
C-CT--0.025200	-1	8.872209	-3.258723	-6.687184	L	
C-C-0.597300	0	8.797276	-1.757560	-6.457764	L	
O-O--0.567900	0	8.791616	-0.979357	-7.409068	L	
H-H-0.271900	0	9.486793	-4.013135	-4.811760	L	
H-H1-0.069800	0	7.870338	-3.666814	-6.557447	L	
H-H1-0.069800	0	9.174404	-3.429516	-7.720925	L	
N-N--0.415700	0	8.741292	-1.357895	-5.190597	L	
C-CT--0.002400	-1	8.593434	0.028379	-4.767018	L	
C-C-0.597300	0	9.716663	0.452893	-3.813751	L	
O-O--0.567900	0	10.340460	-0.403643	-3.179384	L	
C-CT--0.034300	0	7.214714	0.173703	-4.096697	L	
C-CA-0.011800	0	6.045248	0.010450	-5.049977	L	
C-CA--0.125600	0	5.814114	0.974799	-6.049713	L	
C-CA--0.125600	0	5.192842	-1.105102	-4.950229	L	
C-CA--0.170400	0	4.743062	0.822399	-6.948172	L	

C-CA--0.170400	0	4.121472	-1.256759	-5.848349	L	
C-CA--0.107200	0	3.898967	-0.297180	-6.850606	L	
H-H-0.271900	0	8.754550	-2.068435	-4.468045	L	
H-H1-0.097800	0	8.630389	0.680104	-5.639676	L	
H-HC-0.029500	0	7.131220	1.154456	-3.629722	L	
H-HC-0.029500	0	7.137896	-0.563982	-3.296153	L	
H-HA-0.133000	0	6.465228	1.831970	-6.136339	L	
H-HA-0.133000	0	5.356870	-1.848991	-4.184589	L	
H-HA-0.143000	0	4.571962	1.563130	-7.716200	L	
H-HA-0.143000	0	3.464030	-2.109864	-5.765845	L	
H-HA-0.129700	0	3.075133	-0.419083	-7.539380	L	
N-N--0.254800	0	9.972679	1.766104	-3.659390	L	
C-CT--0.026600	-1	10.831828	2.254987	-2.586722	L	
C-C-0.589600	0	10.198240	1.992036	-1.212129	L	
O-O--0.574800	0	9.005774	1.679141	-1.137782	L	
C-CT--0.007000	0	10.988644	3.757308	-2.856680	L	
C-CT-0.018900	0	9.674518	4.128060	-3.541423	L	
C-CT-0.019200	0	9.373311	2.886082	-4.379986	L	
H-H1-0.064100	0	11.803172	1.764579	-2.644218	L	
H-HC-0.025300	0	11.816868	3.918208	-3.548323	L	
H-HC-0.025300	0	11.140162	4.334967	-1.943504	L	
H-HC-0.021300	0	9.773302	5.022030	-4.157960	L	
H-HC-0.021300	0	8.893767	4.265063	-2.790657	L	
H-H1-0.039100	0	8.296187	2.778636	-4.498618	L	
H-H1-0.039100	0	9.848176	2.979274	-5.357513	L	
N-N--0.254800	0	10.960458	2.103952	-0.113917	L	
C-CT--0.026600	-1	10.402361	2.094778	1.231765	L	
C-C-0.589600	0	9.348216	3.179945	1.453993	L	
O-O--0.574800	0	9.599924	4.356338	1.202767	L	
C-CT--0.007000	0	11.598219	2.286687	2.175112	L	
C-CT-0.018900	0	12.790050	1.802127	1.352835	L	
C-CT-0.019200	0	12.409600	2.212926	-0.066761	L	
H-H1-0.064100	0	9.962573	1.113205	1.414264	L	
H-HC-0.025300	0	11.480885	1.709833	3.093048	L	
H-HC-0.025300	0	11.735233	3.344241	2.408407	L	
H-HC-0.021300	0	12.858997	0.714523	1.411470	L	
H-HC-0.021300	0	13.722655	2.267015	1.673502	L	
H-H1-0.039100	0	12.703337	3.248003	-0.246381	L	
H-H1-0.039100	0	12.890666	1.546836	-0.783889	L	
N-N--0.415700	0	8.185677	2.759610	1.942184	L	
C-CT-0.018800	-1	7.026393	3.586745	2.229532	L	H-HC 33
C-C-0.597300	0	6.168996	2.998920	3.346906	L	
O-O--0.567900	0	6.175003	1.787960	3.566554	L	
C-CT--0.515368	0	6.205244	3.692041	0.917816	H	
C-CM-0.287903	0	5.622970	2.405068	0.415811	H	
N-N2--0.582625	0	6.267852	1.604328	-0.512989	H	
C-CM--0.036893	0	4.495906	1.688302	0.744668	H	
C-CM-0.243454	0	5.538155	0.475771	-0.705544	H	
N-N2--0.523998	0	4.459056	0.492901	0.054640	H	
H-H-0.271900	0	8.055852	1.763674	2.048832	L	
H-H1-0.088100	0	7.357367	4.584773	2.532391	L	
H-HC-0.162301	0	6.856342	4.148496	0.157955	H	
H-HC-0.188340	0	5.402928	4.418993	1.094837	H	
H-H-0.339448	0	7.177073	1.810163	-0.926583	H	

H-H4-0.185957	0	3.715403	1.952498	1.442934	H	
H-H5-0.211295	0	5.821744	-0.325419	-1.372940	H	
N-N--0.415700	0	5.406567	3.864322	4.003460	L	
C-CT-0.014300	-1	4.556776	3.566245	5.146978	L	
C-C-0.597300	0	5.202903	2.613421	6.164194	L	
O-O--0.567900	0	4.676877	1.560672	6.528710	L	
C-CT--0.204100	0	3.112831	3.283123	4.706889	L	
C-C-0.713000	0	2.165633	4.165898	5.497540	L	
N-N--0.919100	0	1.593233	3.691526	6.569460	L	
O-O--0.593100	0	1.962332	5.328860	5.193927	L	
H-H-0.271900	0	5.552174	4.853100	3.791203	L	
H-H1-0.104800	0	4.515532	4.517411	5.682657	L	
H-HC-0.079700	0	2.877270	2.234767	4.857672	L	
H-HC-0.079700	0	2.980671	3.521083	3.651673	L	
H-H-0.419600	0	0.925637	4.268967	7.032990	L	
H-H-0.419600	0	1.652046	2.683724	6.718504	L	
N-N--0.415700	0	6.388833	3.041863	6.609579	L	
C-CT--0.149000	-1	7.295136	2.291208	7.456936	L	
H-H-0.271900	0	6.703834	3.916092	6.212541	L	
H-H1-0.097600	0	8.047423	2.952279	7.886829	L	
H-H1-0.097600	0	7.785348	1.513515	6.868168	L	
H-H1-0.097600	0	6.736204	1.804545	8.259446	L	
C-CT--0.366200	-1	-2.266051	-2.768729	9.531654	L	
C-C-0.597200	0	-2.375109	-3.859783	8.502942	L	
O-O--0.567900	0	-2.904299	-4.928768	8.794923	L	
H-HC-0.112300	0	-1.215294	-2.535912	9.693036	L	
H-HC-0.112300	0	-2.717590	-3.108820	10.461230	L	
H-HC-0.112300	0	-2.786033	-1.883879	9.169578	L	
N-N--0.347900	0	-1.853248	-3.591699	7.309037	L	
C-CT--0.240000	-1	-1.757684	-4.555588	6.206383	L	
C-C-0.734100	0	-0.368089	-4.557626	5.581475	L	
O-O--0.589400	0	0.431051	-3.653310	5.802551	L	
C-CT--0.009400	0	-2.905067	-4.321800	5.214867	L	
C-CT-0.018700	0	-2.783201	-3.020698	4.419006	L	H-HC 37
C-CT--0.464524	0	-4.033864	-2.855498	3.553152	H	
C-CT--0.195159	0	-4.019112	-1.581278	2.717671	H	
N-N3--0.748751	0	-3.028889	-1.569856	1.624729	H	
H-H-0.274700	0	-1.467211	-2.660245	7.169001	L	
H-H1-0.142600	0	-1.894603	-5.553892	6.622814	L	
H-HC-0.036200	0	-3.844333	-4.315621	5.770946	L	
H-HC-0.036200	0	-2.950287	-5.148498	4.512932	L	
H-HC-0.010300	0	-1.893578	-3.051117	3.788355	L	
H-HC-0.010300	0	-2.717015	-2.178584	5.101865	L	
H-HC-0.173903	0	-4.905960	-2.852465	4.214396	H	
H-HC-0.148535	0	-4.116665	-3.728481	2.889132	H	
H-HP-0.236066	0	-3.842343	-0.693521	3.330485	H	
H-HP-0.172509	0	-4.998322	-1.447340	2.238498	H	
H-H-0.437293	0	-3.144976	-0.098371	1.085775	H	
H-H-0.366736	0	-3.302443	-2.274558	0.926664	H	
H-H-0.419451	0	-2.082446	-1.778845	1.940571	H	
N-N--0.415700	0	-0.056878	-5.608616	4.836119	L	
C-CT--0.025200	-1	1.264572	-5.821410	4.253400	L	
C-C-0.597300	0	1.144907	-6.536999	2.906003	L	
O-O--0.567900	0	1.720250	-7.599925	2.681870	L	

H-H-0.271900	0	-0.789895	-6.281313	4.633071	L
H-H1-0.069800	0	1.869689	-6.430585	4.924878	L
H-H1-0.069800	0	1.769353	-4.868664	4.089892	L
N-N--0.415700	0	0.343747	-5.942132	2.026089	L
C-CT--0.025200	-1	-0.241254	-6.537867	0.827228	L
C-C-0.597300	0	-1.371187	-5.637764	0.388053	L
O-O--0.567900	0	-2.253959	-5.360274	1.193686	L
H-H-0.271900	0	-0.083753	-5.084513	2.350829	L
H-H1-0.069800	0	-0.637112	-7.526222	1.060675	L
H-H1-0.069800	0	0.516506	-6.614106	0.047048	L
N-N--0.415700	0	-1.324968	-5.178646	-0.862122	L
C-CT--0.001400	-1	-2.158858	-4.093140	-1.379875	L
C-C-0.597300	0	-3.629258	-4.177746	-0.955041	L
O-O--0.567900	0	-4.215448	-3.208364	-0.487820	L
C-CT--0.015200	0	-2.054414	-4.048034	-2.917874	L
C-CA--0.001100	0	-0.663579	-4.213907	-3.504355	L
C-CA--0.190600	0	0.352846	-3.295075	-3.185638	L
C-CA--0.190600	0	-0.392929	-5.278859	-4.386988	L
C-CA--0.234100	0	1.638970	-3.440739	-3.737808	L
C-CA--0.234100	0	0.891776	-5.428781	-4.943670	L
C-C-0.322600	0	1.911941	-4.508187	-4.617448	L
O-OH--0.557900	0	3.147077	-4.649898	-5.167175	L
H-H-0.271900	0	-0.504313	-5.396411	-1.408822	L
H-H1-0.087600	0	-1.763817	-3.154609	-0.987663	L
H-HC-0.029500	0	-2.445285	-3.093633	-3.264008	L
H-HC-0.029500	0	-2.697700	-4.822888	-3.335856	L
H-HA-0.169900	0	0.150278	-2.469669	-2.515619	L
H-HA-0.169900	0	-1.178967	-5.970041	-4.654620	L
H-HA-0.165600	0	2.415988	-2.734038	-3.485091	L
H-HA-0.165600	0	1.091586	-6.237926	-5.626978	L
H-HO-0.399200	0	3.165286	-5.370016	-5.795450	L
N-N--0.415700	0	-4.219991	-5.363915	-1.098903	L
C-CT--0.024900	-1	-5.563624	-5.631848	-0.607481	L
C-C-0.597300	0	-5.696331	-5.558130	0.911359	L
O-O--0.567900	0	-5.065257	-6.321381	1.651763	L
C-CT-0.211700	0	-6.032303	-7.021808	-1.041252	L
O-OH--0.654600	-1	-7.287409	-7.329341	-0.453470	L
H-H-0.271900	0	-3.638789	-6.114678	-1.425535	L
H-H1-0.084300	0	-6.240605	-4.900944	-1.050409	L
H-H1-0.035200	0	-5.300658	-7.763834	-0.719305	L
H-H1-0.035200	0	-6.113368	-7.054640	-2.127783	L
H-HO-0.427500	-1	-7.546121	-8.200360	-0.764416	L
N-N--0.415700	0	-6.666068	-4.754607	1.340514	L
C-CT-0.033700	-1	-7.164620	-4.664538	2.701384	L
C-C-0.597300	0	-7.527403	-5.991769	3.372044	L
O-O--0.567900	0	-7.471911	-6.096943	4.595530	L
C-CT--0.182500	0	-8.350821	-3.700069	2.706010	L
H-H-0.271900	0	-7.120203	-4.168322	0.646615	L
H-H1-0.082300	0	-6.381499	-4.224174	3.312967	L
H-HC-0.060300	0	-8.054386	-2.733102	2.295256	L
H-HC-0.060300	0	-8.697754	-3.563059	3.725625	L
H-HC-0.060300	0	-9.165079	-4.115092	2.117726	L
N-N--0.415700	0	-7.911191	-6.997153	2.584929	L
C-CT--0.025200	-1	-8.297517	-8.309162	3.089403	L

```
C-C-0.597300      0   -7.338060   -9.439169    2.739552 L
O-O--0.567900     0   -7.751864  -10.595132    2.681988 L
H-H-0.271900      0   -7.887373   -6.829999    1.585340 L
H-H1-0.069800     0   -9.272436   -8.563223    2.674500 L
H-H1-0.069800     0   -8.401552   -8.288527    4.174478 L
N-N--0.415700     0   -6.056717   -9.128639    2.548695 L
C-CT--0.024900   -1   -4.983777  -10.122467    2.566021 L
C-C-0.597300      0   -4.287716  -10.090160    3.920784 L
O-O--0.567900     0   -3.966664   -9.016616    4.440079 L
C-CT-0.211700     0   -3.987296   -9.827546    1.440088 L
O-OH--0.654600   -1   -3.104498  -10.921992    1.248415 L
H-H-0.271900      0   -5.803039   -8.146662    2.606058 L
H-H1-0.084300     0   -5.395714  -11.117361    2.396426 L
H-H1-0.035200     0   -3.420688   -8.923973    1.671283 L
H-H1-0.035200     0   -4.541629   -9.664245    0.515056 L
H-HO-0.427500    -1   -2.562346  -10.731974    0.479250 L
N-N--0.415700     0   -4.091203  -11.269837    4.508730 L
C-CT--0.149000   -1   -3.555031  -11.422670    5.850039 L
H-H-0.271900      0   -4.371212  -12.086721    3.989549 L
H-H1-0.097600     0   -3.352809  -12.472902    6.060351 L
H-H1-0.097600     0   -4.269721  -11.039133    6.581544 L
H-H1-0.097600     0   -2.627363  -10.853473    5.947117 L
C-CT--0.366200   -1  -12.124877    5.111790   -0.646708 L
C-C-0.597200      0  -10.695320    5.286322   -0.205649 L
O-O--0.567900     0  -10.379067    5.005647    0.945054 L
H-HC-0.112300     0  -12.515916    6.068180   -0.986854 L
H-HC-0.112300     0  -12.711930    4.755075    0.198044 L
H-HC-0.112300     0  -12.161831    4.377385   -1.449228 L
N-N--0.415700     0   -9.844690    5.739431   -1.122388 L
C-CT--0.025200   -1   -8.411278    5.896601   -0.890699 L
C-C-0.597300      0   -7.593165    5.808947   -2.154561 L
O-O--0.567900     0   -8.086758    6.151776   -3.235119 L
H-H-0.271900      0  -10.158864    5.915038   -2.064386 L
H-H1-0.069800     0   -8.099551    5.094781   -0.231397 L
H-H1-0.069800     0   -8.210390    6.851255   -0.406616 L
N-N--0.415700     0   -6.345731    5.367088   -1.985428 L H-H1 48
C-CT--0.530209   -1   -5.324669    5.323632   -3.032806 H
C-C-0.568093      0   -4.586976    4.001569   -2.933567 H
O-O--0.528105     0   -4.530549    3.374312   -1.870713 H
C-CT-0.298500     0   -4.382577    6.547918   -2.945147 L H-H1 48
C-CT--0.319200    0   -5.132907    7.843021   -3.261223 L
C-CT--0.319200    0   -3.704334    6.679838   -1.578755 L
H-H-0.271900      0   -6.071818    5.043733   -1.058895 L
H-H1-0.184096     0   -5.838935    5.391911   -3.998180 H
H-HC--0.029700    0   -3.604637    6.438541   -3.698988 L
H-HC-0.079100     0   -5.865252    8.051292   -2.482807 L
H-HC-0.079100     0   -5.643762    7.743899   -4.217958 L
H-HC-0.079100     0   -4.424281    8.668459   -3.315899 L
H-HC-0.079100     0   -4.446977    6.817217   -0.793904 L
H-HC-0.079100     0   -3.029047    7.533665   -1.582008 L
H-HC-0.079100     0   -3.132181    5.779598   -1.357799 L
N-N--0.610103     0   -3.958696    3.573807   -4.055638 H
C-CT--0.314060   -1   -4.211144    4.038019   -5.412402 H
C-C-0.597300      0   -5.331393    3.227116   -6.045920 L H-H1 50
```

```
O-O--0.567900    0   -5.458460    2.023362   -5.823634 L
C-CT--0.110200   0   -2.917314    3.904680   -6.252992 L H-H1 50
C-CT-0.353100    0   -1.823510    4.960514   -5.964551 L
C-CT--0.412100   0   -1.087581    4.764899   -4.635017 L
C-CT--0.412100   0   -0.768837    4.917875   -7.073584 L
H-H-0.398207     0   -3.545377    2.649894   -3.976720 H
H-H1-0.165130    0   -4.495786    5.088236   -5.409207 H
H-HC-0.045700    0   -3.198383    4.003989   -7.301820 L
H-HC-0.045700    0   -2.499113    2.906258   -6.123439 L
H-HC--0.036100   0   -2.277105    5.952150   -5.974007 L
H-HC-0.100000    0   -0.718231    3.742441   -4.565185 L
H-HC-0.100000    0   -1.775363    4.961443   -3.816223 L
H-HC-0.100000    0   -0.258013    5.467116   -4.557622 L
H-HC-0.100000    0   -0.274516    3.947825   -7.076963 L
H-HC-0.100000    0   -0.026848    5.698231   -6.904484 L
H-HC-0.100000    0   -1.240630    5.088935   -8.039963 L
N-N--0.415700    0   -6.115668    3.878675   -6.899365 L
C-CT--0.038900  -1   -7.300739    3.283334   -7.528712 L
C-C-0.597300     0   -7.050310    1.947166   -8.216439 L
O-O--0.567900    0   -7.920164    1.079486   -8.237390 L
C-CT-0.365400   -1   -7.934565    4.213257   -8.587710 L
O-OH--0.676100  -1   -7.870903    5.568532   -8.134246 L
C-CT--0.243800   0   -9.411817    3.921943   -8.849791 L
H-H-0.271900     0   -5.992518    4.876439   -6.990114 L
H-H1-0.100700    0   -8.044695    3.139912   -6.751819 L
H-H1-0.004300    0   -7.377825    4.139553   -9.522885 L
H-HC-0.064200    0   -9.525458    2.916741   -9.255699 L
H-HC-0.064200    0   -9.804404    4.633905   -9.575671 L
H-HC-0.064200    0   -9.978452    3.995099   -7.920710 L
H-HO-0.410200   -1   -8.768494    5.905361   -8.098809 L
N-N--0.415700    0   -5.880267    1.819660   -8.839426 L
C-CT--0.025200  -1   -5.495589    0.658912   -9.629997 L
C-C-0.597300     0   -4.590367   -0.337623   -8.939012 L
O-O--0.567900    0   -4.088424   -1.235009   -9.615716 L
H-H-0.271900     0   -5.203003    2.549614   -8.680604 L
H-H1-0.069800    0   -4.975594    1.002570  -10.523104 L
H-H1-0.069800    0   -6.384681    0.118963   -9.955981 L
N-N--0.415700    0   -4.373327   -0.162437   -7.630097 L H-H1 54
C-CT--0.539097  -1   -3.459832   -0.967373   -6.829281 H
C-C-0.572106     0   -4.207231   -1.446491   -5.583787 H
O-O--0.533001    0   -4.905025   -2.453978   -5.667194 H
C-CT-0.130300    0   -2.078225   -0.286635   -6.664227 L H-H1 54
C-CT--0.043000   0   -1.455227    0.125946   -8.018971 L
C-CT--0.320400   0   -1.166401   -1.290983   -5.936947 L
C-CT--0.066000   0   -0.082925    0.802924   -7.910306 L
H-H-0.271900     0   -4.848834    0.600002   -7.156701 L
H-H1-0.180646    0   -3.281254   -1.888653   -7.395781 H
H-HC-0.018700    0   -2.194880    0.610092   -6.065642 L
H-HC-0.023600    0   -2.116851    0.840777   -8.506813 L
H-HC-0.023600    0   -1.367355   -0.751505   -8.660887 L
H-HC-0.088200    0   -1.059292   -2.198021   -6.530856 L
H-HC-0.088200    0   -1.588096   -1.552998   -4.968540 L
H-HC-0.088200    0   -0.183468   -0.866067   -5.754059 L
H-HC-0.018600    0   -0.129340    1.618126   -7.189841 L
```

```
H-HC-0.018600    0    0.199104    1.203140   -8.884195 L
H-HC-0.018600    0    0.673461    0.083193   -7.600670 L
N-N--0.611145    0   -4.088872   -0.692682   -4.464961 H
C-CT--0.109296  -1   -4.630155   -1.081337   -3.169476 H
C-C-0.597300     0   -6.157851   -1.089725   -3.155346 L H-H1 56
O-O--0.567900    0   -6.791256   -2.017526   -2.640112 L
C-CT--0.388038   0   -4.046056   -0.202372   -2.070270 H
H-H-0.369964     0   -3.494853    0.128613   -4.454496 H
H-H1-0.178541    0   -4.364275   -2.122192   -2.963798 H
H-H1-0.157964    0   -4.501146   -0.485322   -1.117890 H
H-H1-0.179833    0   -4.234328    0.864428   -2.208120 H
H-HS-0.319500    0   -2.327565    3.243461   -1.366360 H
N-N--0.415700    0   -6.747012   -0.096478   -3.811004 L
C-CT--0.087500  -1   -8.183369   -0.030676   -4.066820 L
C-C-0.597300     0   -8.665226   -1.179152   -4.939453 L
O-O--0.567900    0   -9.602755   -1.882408   -4.564804 L
C-CT-0.298500    0   -8.576535    1.335969   -4.645696 L
C-CT--0.319200   0  -10.090121    1.463552   -4.864375 L
C-CT--0.319200   0   -8.145582    2.500388   -3.739540 L
H-H-0.271900     0   -6.109164    0.577223   -4.224885 L
H-H1-0.096900    0   -8.692893   -0.134538   -3.110757 L
H-HC--0.029700   0   -8.082342    1.434595   -5.603257 L
H-HC-0.079100    0  -10.615438    1.301852   -3.922734 L
H-HC-0.079100    0  -10.426988    0.729710   -5.596453 L
H-HC-0.079100    0  -10.324780    2.457536   -5.244106 L
H-HC-0.079100    0   -8.613820    2.415540   -2.760332 L
H-HC-0.079100    0   -8.422257    3.453037   -4.190461 L
H-HC-0.079100    0   -7.063293    2.501974   -3.605555 L
N-N--0.415700    0   -8.033773   -1.381035   -6.094482 L
C-CT--0.025200  -1   -8.393732   -2.435562   -7.039312 L
C-C-0.597300     0   -8.310258   -3.839237   -6.452709 L
O-O--0.567900    0   -9.212393   -4.650140   -6.649623 L
H-H-0.271900     0   -7.247363   -0.780307   -6.304212 L
H-H1-0.069800    0   -7.724906   -2.384903   -7.898200 L
H-H1-0.069800    0   -9.413383   -2.269822   -7.386729 L
N-N--0.415700    0   -7.228417   -4.120858   -5.729028 L
C-CT--0.025200  -1   -7.008623   -5.388811   -5.044756 L
C-C-0.597300     0   -7.939600   -5.651538   -3.872142 L
O-O--0.567900    0   -8.272254   -6.803995   -3.601927 L
H-H-0.271900     0   -6.508868   -3.403301   -5.660974 L
H-H1-0.069800    0   -5.984835   -5.411593   -4.673011 L
H-H1-0.069800    0   -7.130400   -6.202719   -5.759678 L
N-N--0.415700    0   -8.328596   -4.595850   -3.160649 L
C-CT--0.038900  -1   -9.381859   -4.657660   -2.144894 L
C-C-0.597300     0  -10.751538   -4.945945   -2.750562 L
O-O--0.567900    0  -11.497818   -5.780185   -2.238299 L
C-CT-0.365400    0   -9.420576   -3.359475   -1.323814 L
O-OH--0.676100   0   -8.186753   -3.195537   -0.662708 L
C-CT--0.243800   0  -10.509878   -3.373113   -0.251831 L
H-H-0.271900     0   -7.942769   -3.690671   -3.405489 L
H-H1-0.100700    0   -9.156863   -5.476107   -1.461124 L
H-H1-0.004300    0   -9.584515   -2.505361   -1.980966 L
H-HC-0.064200    0  -11.488922   -3.264026   -0.717128 L
H-HC-0.064200    0  -10.356101   -2.545204    0.439790 L
```

H-HC-0.064200	0	-10.478954	-4.314493	0.295042	L
H-HO-0.410200	0	-7.595295	-2.730790	-1.289885	L
N-N--0.415700	0	-11.097556	-4.235162	-3.825036	L
C-CT--0.149000	-1	-12.330252	-4.420154	-4.562528	L
H-H-0.271900	0	-10.427785	-3.553942	-4.171147	L
H-H1-0.097600	0	-12.399808	-3.669647	-5.350843	L
H-H1-0.097600	0	-13.182739	-4.326674	-3.888351	L
H-H1-0.097600	0	-12.335155	-5.414416	-5.012439	L
N-N2--0.511415	0	2.004377	-0.021485	-1.689655	H
C-CA-0.057801	0	0.685191	0.180531	-1.694375	H
C-CA-0.063496	0	-0.001807	0.656459	-2.817233	H
C-CA--0.109122	0	0.738354	1.022565	-3.942651	H
C-CA--0.164490	0	2.114197	0.833775	-3.930080	H
C-CA-0.098392	0	2.700371	0.280229	-2.795701	H
C-C-0.541263	0	-1.486688	0.688692	-2.899861	H
O-O--0.574884	0	-2.070717	1.339305	-3.764244	H
N-N--0.751108	0	-1.325842	3.188332	-1.541898	H
H-H4-0.220889	0	0.169064	-0.036585	-0.766312	H
H-HA-0.160434	0	0.221785	1.425107	-4.806568	H
H-HA-0.142191	0	2.729285	1.098947	-4.784130	H
H-H4-0.174502	0	3.763095	0.070520	-2.760135	H
H-H-0.412468	0	-1.146099	3.803845	-2.334441	H
H-H-0.353801	0	-0.856594	3.638098	-0.748913	H
Zn-ZN-0.974537	0	2.866047	-1.133212	0.041591	H
O-OW--0.834000	0	-2.140225	-7.252210	3.448209	L
H-HW-0.417000	0	-2.587136	-6.651065	2.832513	L
H-HW-0.417000	0	-2.859984	-7.802545	3.798306	L
O-OW--0.783903	0	1.304322	-2.564694	-0.131695	H
H-HW-0.401253	0	0.926093	-2.458016	0.785333	H
H-HW-0.424111	0	1.901618	-3.352047	-0.183128	H
O-OW--0.834000	0	-3.638869	-8.454620	7.183802	L
H-HW-0.417000	0	-3.715652	-7.513359	7.359411	L
H-HW-0.417000	0	-3.778829	-8.528168	6.227713	L
O-OW--0.834000	0	7.225409	-0.035665	1.858324	L
H-HW-0.417000	0	6.913530	0.352393	2.695236	L
H-HW-0.417000	0	6.440233	0.066906	1.298126	L
O-OW--0.834000	0	7.047359	-4.980318	4.771290	L
H-HW-0.417000	0	6.096393	-5.041867	4.996588	L
H-HW-0.417000	0	7.262948	-5.868605	4.484310	L
O-OW--0.834000	0	8.596069	-0.955189	-0.259804	L
H-HW-0.417000	0	8.179008	-0.577295	0.540375	L
H-HW-0.417000	0	8.845041	-0.166117	-0.762002	L
O-OW--0.834000	0	12.627649	-1.079330	-1.912190	L
H-HW-0.417000	0	11.785548	-0.831579	-2.330977	L
H-HW-0.417000	0	12.648253	-2.039941	-2.042244	L
O-OW--0.834000	0	8.268072	-3.327640	0.985757	L
H-HW-0.417000	0	8.462303	-2.560645	0.408129	L
H-HW-0.417000	0	7.558088	-2.988420	1.540542	L
O-OW--0.834000	0	4.357193	-4.940450	5.168174	L
H-HW-0.417000	0	4.089569	-4.555040	6.025396	L
H-HW-0.417000	0	4.033224	-4.285957	4.539239	L
S-SH--0.326142	0	-2.249448	-0.475547	-1.814336	H

IC2'

```
C-CT--0.3662     -1  -10.301742    3.974122    3.993757 L
C-C-0.5972        0   -8.974319    3.318406    3.749304 L
O-O--0.5679       0   -8.392999    2.763048    4.675196 L
H-HC-0.1123       0  -11.048691    3.507944    3.354347 L
H-HC-0.1123       0  -10.221194    5.033245    3.755340 L
H-HC-0.1123       0  -10.575325    3.847491    5.038785 L
N-N--0.415700     0   -8.521436    3.388197    2.504049 L
C-CT--0.059700   -1   -7.335953    2.687294    2.004522 L
C-C-0.597300      0   -6.195000    3.678708    1.825736 L
O-O--0.567900     0   -6.355751    4.684936    1.140324 L
C-CT-0.130300     0   -7.651571    1.922984    0.694390 L
C-CT--0.043000    0   -8.853416    0.961670    0.880534 L
C-CT--0.320400    0   -6.416728    1.125174    0.236440 L
C-CT--0.066000    0   -9.347888    0.314546   -0.417343 L
H-H-0.271900      0   -9.057716    3.944145    1.846412 L
H-H1-0.086900     0   -7.024883    1.948328    2.742703 L
H-HC-0.018700     0   -7.902500    2.650001   -0.080208 L
H-HC-0.023600     0   -9.701210    1.507734    1.292202 L
H-HC-0.023600     0   -8.585172    0.176085    1.588092 L
H-HC-0.088200     0   -6.150722    0.365290    0.971186 L
H-HC-0.088200     0   -5.559761    1.785808    0.094352 L
H-HC-0.088200     0   -6.601727    0.651898   -0.725651 L
H-HC-0.018600     0   -9.563756    1.084943   -1.156195 L
H-HC-0.018600     0  -10.262845   -0.241030   -0.215619 L
H-HC-0.018600     0   -8.600190   -0.374666   -0.806395 L
N-N--0.516300     0   -5.081603    3.399860    2.498301 L
C-CT-0.038100    -1   -3.766012    4.012431    2.364658 L H-HC      30
C-C-0.536600      0   -3.669788    5.531233    2.125859 L
O-O--0.581900     0   -2.671874    6.045738    1.620245 L
C-CT--0.547145    0   -3.018725    3.213375    1.298191 H
C-C-0.532653      0   -2.758350    1.773961    1.698695 H
O-O2--0.588507    0   -2.572848    1.460004    2.881003 H
O-O2--0.625559    0   -2.697653    0.946995    0.696250 H
H-H-0.293600      0   -5.064928    2.513962    2.986957 L
H-H1-0.088000     0   -3.240778    3.849750    3.306306 L
H-HC-0.128435     0   -2.034673    3.686991    1.160276 H
H-HC-0.183556     0   -3.534712    3.291103    0.337769 H
N-N--0.415700     0   -4.647578    6.290938    2.600254 L
C-CT--0.025200   -1   -4.631196    7.734022    2.469826 L
C-C-0.597300      0   -3.733987    8.382327    3.509575 L
O-O--0.567900     0   -4.209363    9.054415    4.426215 L
H-H-0.271900      0   -5.482545    5.791742    2.866110 L
H-H1-0.069800     0   -5.643266    8.114902    2.598158 L
H-H1-0.069800     0   -4.283439    8.013823    1.474120 L
N-N--0.415700     0   -2.430868    8.165609    3.365525 L
C-CT--0.038900   -1   -1.407518    8.595156    4.309018 L
C-C-0.597300      0   -0.500657    9.679560    3.714881 L
O-O--0.567900     0   -0.603646   10.053358    2.545288 L
C-CT-0.365400     0   -0.556262    7.435492    4.871268 L
O-OH--0.676100    0    0.609271    7.253737    4.107299 L
C-CT--0.243800    0   -1.276875    6.097414    5.002032 L
H-H-0.271900      0   -2.167305    7.562407    2.588502 L
H-H1-0.100700     0   -1.909689    9.046111    5.163035 L
```

H-H1-0.004300	0	-0.234861	7.729641	5.870977	L
H-HC-0.064200	0	-2.211575	6.233791	5.544006	L
H-HC-0.064200	0	-0.643432	5.402476	5.547801	L
H-HC-0.064200	0	-1.481205	5.679251	4.016780	L
H-HO-0.410200	0	1.112976	6.503465	4.491322	L
N-N--0.415700	0	0.424093	10.183965	4.535934	L
C-CT--0.025200	-1	1.467926	11.112725	4.114332	L
C-C-0.597300	0	2.362564	10.615161	2.987765	L
O-O--0.567900	0	2.689731	11.382402	2.087415	L
H-H-0.271900	0	0.487697	9.781519	5.455624	L
H-H1-0.069800	0	2.106970	11.346346	4.964729	L
H-H1-0.069800	0	0.999133	12.037757	3.777956	L
N-N--0.516300	0	2.762356	9.347992	3.054984	L
C-CT-0.038100	-1	3.668820	8.740075	2.082118	L
C-C-0.536600	0	3.035224	8.434790	0.738408	L
O-O--0.581900	0	3.768220	8.129739	-0.195728	L
C-CT--0.030300	0	4.247245	7.428426	2.643872	L
C-C-0.799400	-1	5.594965	7.627546	3.317379	L
O-O2--0.801400	-1	6.178602	8.729590	3.230713	L
O-O2--0.801400	-1	6.051415	6.676129	3.983471	L
H-H-0.293600	0	2.394293	8.749803	3.780454	L
H-H1-0.088000	0	4.473592	9.442006	1.860796	L
H-HC--0.012200	0	4.399187	6.704994	1.841025	L
H-HC--0.012200	0	3.542962	6.981714	3.348648	L
N-N--0.415700	0	1.712425	8.515092	0.625275	L
C-CT--0.002400	-1	1.000119	8.324025	-0.631425	L
C-C-0.597300	0	0.419064	9.622752	-1.190471	L
O-O--0.567900	0	0.403586	9.828425	-2.404161	L
C-CT--0.034300	0	-0.079945	7.246358	-0.473245	L
C-CA-0.011800	0	0.432289	5.883365	-0.037374	L
C-CA--0.125600	0	0.961699	4.977288	-0.976136	L
C-CA--0.125600	0	0.325247	5.496397	1.309287	L
C-CA--0.170400	0	1.363181	3.692898	-0.563561	L
C-CA--0.170400	0	0.720023	4.214259	1.723346	L
C-CA--0.107200	0	1.231337	3.309838	0.780755	L
H-H-0.271900	0	1.190855	8.718513	1.465242	L
H-H1-0.097800	0	1.691197	7.954644	-1.388554	L
H-HC-0.029500	0	-0.582297	7.123125	-1.433255	L
H-HC-0.029500	0	-0.827080	7.594496	0.241616	L
H-HA-0.133000	0	1.016350	5.247338	-2.020117	L
H-HA-0.133000	0	-0.120327	6.174244	2.015312	L
H-HA-0.143000	0	1.711640	2.967467	-1.284115	L
H-HA-0.143000	0	0.580531	3.911040	2.750285	L
H-HA-0.129700	0	1.482690	2.304942	1.077427	L
N-N--0.4157	0	-0.032439	10.523389	-0.315640	L
C-CT--0.1490	-1	-0.437848	11.862048	-0.693073	L
H-H-0.2719	0	-0.030021	10.270052	0.667133	L
H-H1-0.0976	0	-0.817406	12.388477	0.183146	L
H-H1-0.0976	0	-1.218837	11.807124	-1.453327	L
H-H1-0.0976	0	0.420058	12.400801	-1.098108	L
C-CT--0.3662	-1	8.010148	8.420600	-0.192039	L
C-C-0.5972	0	7.546319	7.624238	-1.388085	L
O-O--0.5679	0	8.367545	7.271434	-2.231087	L
H-HC-0.1123	0	7.572745	9.415840	-0.227966	L

H-HC-0.1123	0	9.095494	8.488141	-0.205060	L
H-HC-0.1123	0	7.692862	7.917472	0.722289	L
N-N--0.415700	0	6.235373	7.384452	-1.485335	L
C-CT--0.051800	-1	5.586051	6.839084	-2.685525	L
C-C-0.597300	0	4.918835	7.937785	-3.512949	L
O-O--0.567900	0	5.240190	8.145136	-4.683166	L
C-CT--0.110200	0	4.665004	5.647746	-2.349405	L
C-CT-0.353100	0	4.876150	4.480081	-3.333763	L
C-CT--0.412100	0	4.013962	3.286302	-2.952059	L
C-CT--0.412100	0	4.530025	4.822882	-4.786123	L
H-H-0.271900	0	5.631084	7.707106	-0.736842	L
H-H1-0.092200	0	6.382950	6.453191	-3.322055	L
H-HC-0.045700	0	3.619733	5.959498	-2.356641	L
H-HC-0.045700	0	4.896964	5.287419	-1.346678	L
H-HC--0.036100	0	5.920116	4.166181	-3.288458	L
H-HC-0.100000	0	2.963052	3.506918	-3.140091	L
H-HC-0.100000	0	4.143021	3.053129	-1.897011	L
H-HC-0.100000	0	4.317933	2.423322	-3.539890	L
H-HC-0.100000	0	3.517577	5.224549	-4.840282	L
H-HC-0.100000	0	4.595692	3.929335	-5.405311	L
H-HC-0.100000	0	5.229074	5.561588	-5.174768	L
N-N--0.4157	0	4.020922	8.666698	-2.864007	L
C-CT--0.1490	-1	3.388304	9.872671	-3.330303	L
H-H-0.2719	0	3.856352	8.386944	-1.904772	L
H-H1-0.0976	0	4.092649	10.459689	-3.920971	L
H-H1-0.0976	0	3.048915	10.463095	-2.477989	L
H-H1-0.0976	0	2.526915	9.616615	-3.949545	L
C-CT--0.3662	-1	-8.723867	2.445774	8.110817	L
C-C-0.5972	0	-7.862245	1.227260	7.939153	L
O-O--0.5679	0	-7.555500	0.544460	8.909952	L
H-HC-0.1123	0	-9.640244	2.313271	7.538922	L
H-HC-0.1123	0	-8.183145	3.316301	7.742495	L
H-HC-0.1123	0	-8.956833	2.571965	9.165930	L
N-N--0.415700	0	-7.465900	0.971530	6.700783	L
C-CT--0.038900	-1	-6.668054	-0.186766	6.293799	L
C-C-0.597300	0	-5.400589	0.293766	5.611019	L
O-O--0.567900	0	-5.438362	0.721666	4.458437	L
C-CT-0.365400	0	-7.478287	-1.152354	5.415418	L
O-OH--0.676100	0	-8.037495	-0.488409	4.308000	L
C-CT--0.243800	0	-8.625082	-1.773476	6.218463	L
H-H-0.271900	0	-7.784819	1.594468	5.962127	L
H-H1-0.100700	0	-6.369562	-0.747338	7.179273	L
H-H1-0.004300	0	-6.822147	-1.948361	5.062103	L
H-HC-0.064200	0	-8.224460	-2.307447	7.079731	L
H-HC-0.064200	0	-9.181996	-2.470317	5.598252	L
H-HC-0.064200	0	-9.309970	-0.997456	6.558522	L
H-HO-0.410200	0	-7.343294	0.085592	3.952463	L
N-N--0.415700	0	-4.309246	0.286086	6.380689	L
C-CT--0.059700	-1	-3.111009	1.097577	6.155173	L
C-C-0.597300	0	-1.866287	0.215691	6.093342	L
O-O--0.567900	0	-1.607538	-0.571681	7.008728	L
C-CT-0.130300	0	-2.987761	2.187191	7.245904	L
C-CT--0.043000	0	-4.244145	3.081477	7.394271	L
C-CT--0.320400	0	-1.749322	3.060563	6.995484	L

C-CT--0.066000	0	-4.670311	3.856848	6.138638	L		
H-H-0.271900	0	-4.377839	-0.148117	7.286465	L		
H-H1-0.086900	0	-3.208927	1.605100	5.196635	L		
H-HC-0.018700	0	-2.842587	1.686711	8.205147	L		
H-HC-0.023600	0	-4.062295	3.803174	8.191376	L		
H-HC-0.023600	0	-5.083324	2.465967	7.717245	L		
H-HC-0.088200	0	-1.748941	3.410141	5.962749	L		
H-HC-0.088200	0	-0.846905	2.471618	7.160922	L		
H-HC-0.088200	0	-1.736953	3.911188	7.675601	L		
H-HC-0.018600	0	-3.871133	4.518119	5.808300	L		
H-HC-0.018600	0	-5.550179	4.457977	6.368202	L		
H-HC-0.018600	0	-4.923890	3.169723	5.334612	L		
N-N--0.516300	0	-1.118533	0.374335	5.005733	L		
C-CT-0.038100	-1	0.182552	-0.205101	4.729106	L	H-HC	178
C-C-0.536600	0	1.117386	-0.044298	5.933792	L		
O-O--0.581900	0	1.327170	1.049422	6.452613	L		
C-CT--0.520019	0	0.688225	0.437388	3.420748	H		
C-C-0.636404	0	0.920285	-0.520474	2.257669	H		
O-O2--0.608036	0	0.262690	-1.593603	2.151555	H		
O-O2--0.608131	0	1.792833	-0.107670	1.419820	H		
H-H-0.293600	0	-1.517136	0.950456	4.269334	L		
H-H1-0.088000	0	0.034150	-1.274134	4.567760	L		
H-HC-0.132300	0	1.631069	0.954110	3.616771	H		
H-HC-0.188993	0	-0.020068	1.201508	3.081249	H		
N-N--0.415700	0	1.663882	-1.154095	6.398343	L		
C-CT-0.033700	-1	2.561306	-1.200911	7.530069	L		
C-C-0.597300	0	3.781718	-2.025895	7.137614	L		
O-O--0.567900	0	3.830897	-3.249850	7.304432	L		
C-CT--0.182500	0	1.799715	-1.754182	8.742837	L		
H-H-0.271900	0	1.358861	-2.031137	5.991054	L		
H-H1-0.082300	0	2.907786	-0.195981	7.779041	L		
H-HC-0.060300	0	0.961169	-1.096222	8.974499	L		
H-HC-0.060300	0	2.467741	-1.804765	9.602310	L		
H-HC-0.060300	0	1.420441	-2.752797	8.520024	L		
N-N--0.4157	0	4.752857	-1.336872	6.550627	L		
C-CT-0.0188	-1	5.956247	-1.925647	6.007549	L	H-HC	200
C-C-0.5973	0	7.154863	-1.773235	6.953190	L		
O-O--0.5679	0	7.206057	-0.916372	7.838057	L		
C-CT--0.517456	0	6.207535	-1.301676	4.633189	H		
C-CM-0.287351	0	5.332102	-1.786527	3.536339	H		
N-N2--0.586994	0	5.473962	-3.043432	2.963911	H		
C-CM--0.020338	0	4.350652	-1.181819	2.801925	H		
C-CM-0.242458	0	4.605344	-3.138521	1.922601	H		
N-N2--0.505070	0	3.915460	-2.020007	1.795560	H		
H-H-0.2719	0	4.647801	-0.328278	6.481750	L		
H-H1-0.0881	0	5.807056	-2.992658	5.875589	L		
H-HC-0.166355	0	7.261108	-1.473291	4.367735	H		
H-HC-0.174481	0	6.097972	-0.221840	4.760102	H		
H-H-0.335795	0	6.121555	-3.759149	3.286404	H		
H-H4-0.152777	0	3.947562	-0.187656	2.922058	H		
H-H5-0.209475	0	4.504899	-4.006586	1.285934	H		
N-N--0.4157	0	8.155912	-2.618112	6.713411	L		
C-CT--0.1490	-1	9.424222	-2.548745	7.405877	L		
H-H-0.2719	0	8.001094	-3.301979	5.979250	L		

H-H1-0.0976	0	10.094205	-3.323732	7.032440	L
H-H1-0.0976	0	9.263954	-2.689085	8.476237	L
H-H1-0.0976	0	9.875922	-1.568528	7.242857	L
C-CT--0.3662	-1	8.333934	-9.396141	0.978195	L
C-C-0.5972	0	8.716397	-9.180495	-0.473755	L
O-O--0.5679	0	9.755622	-8.586962	-0.735380	L
H-HC-0.1123	0	8.246903	-10.460356	1.183943	L
H-HC-0.1123	0	7.397117	-8.883111	1.188079	L
H-HC-0.1123	0	9.113776	-8.967193	1.607304	L
N-N--0.254800	0	7.895118	-9.637313	-1.435833	L
C-CT--0.026600	-1	8.138443	-9.406083	-2.861983	L
C-C-0.589600	0	7.997423	-7.933567	-3.247677	L
O-O--0.574800	0	8.715058	-7.442741	-4.116134	L
C-CT--0.007000	0	7.125124	-10.295354	-3.590371	L
C-CT-0.018900	0	5.964730	-10.409305	-2.600389	L
C-CT-0.019200	0	6.657473	-10.379925	-1.237112	L
H-H1-0.064100	0	9.148745	-9.723866	-3.122769	L
H-HC-0.025300	0	7.562137	-11.282298	-3.749502	L
H-HC-0.025300	0	6.806627	-9.866238	-4.541695	L
H-HC-0.021300	0	5.398176	-11.329656	-2.745082	L
H-HC-0.021300	0	5.311314	-9.540920	-2.698127	L
H-H1-0.039100	0	6.013208	-9.897510	-0.500663	L
H-H1-0.039100	0	6.893952	-11.397034	-0.921787	L
N-N--0.516300	0	7.074446	-7.231882	-2.588920	L
C-CT-0.039700	-1	6.824065	-5.805810	-2.769564	L
C-C-0.536600	0	8.084737	-4.941882	-2.642814	L
O-O--0.581900	0	8.263940	-3.962879	-3.370821	L
C-CT-0.056000	0	5.722738	-5.359677	-1.800780	L H-HC 244
C-CT--0.515956	0	5.181196	-3.983263	-2.199641	H
C-C-0.587828	0	4.102975	-3.428500	-1.275359	H
O-O2--0.600319	0	4.066384	-2.146062	-1.239298	H
O-O2--0.589127	0	3.336705	-4.204460	-0.659039	H
H-H-0.293600	0	6.534639	-7.704626	-1.883290	L
H-H1-0.110500	0	6.451346	-5.658246	-3.783596	L
H-HC--0.017300	0	6.118775	-5.322069	-0.784784	L
H-HC--0.017300	0	4.904134	-6.080520	-1.835085	L
H-HC-0.167759	0	4.760797	-4.057006	-3.209895	H
H-HC-0.135230	0	6.002310	-3.259642	-2.241953	H
N-N--0.415700	0	8.992614	-5.345499	-1.756689	L
C-CT--0.024900	-1	10.234629	-4.648625	-1.443778	L
C-C-0.597300	0	11.257184	-4.545809	-2.568563	L
O-O--0.567900	0	12.240459	-3.816621	-2.437005	L
C-CT-0.211700	0	10.873360	-5.285036	-0.203031	L
O-OH--0.654600	0	9.916778	-5.474561	0.829231	L
H-H-0.271900	0	8.778514	-6.150253	-1.184228	L
H-H1-0.084300	0	9.972413	-3.622916	-1.179822	L
H-H1-0.035200	0	11.673172	-4.638020	0.160156	L
H-H1-0.035200	0	11.298611	-6.253583	-0.470418	L
H-HO-0.427500	0	9.365989	-4.663775	0.908872	L
N-N--0.415700	0	11.016503	-5.234207	-3.682478	L
C-CT-0.033700	-1	11.768838	-5.079003	-4.920659	L
C-C-0.597300	0	10.940155	-4.523764	-6.083636	L
O-O--0.567900	0	11.374723	-4.542322	-7.234537	L
C-CT--0.182500	0	12.390723	-6.444226	-5.248769	L

H-H-0.271900	0	10.199970	-5.835211	-3.688664	L
H-H1-0.082300	0	12.587059	-4.373878	-4.768206	L
H-HC-0.060300	0	13.004375	-6.781355	-4.412662	L
H-HC-0.060300	0	13.018654	-6.359142	-6.136297	L
H-HC-0.060300	0	11.601856	-7.174331	-5.436820	L
N-N--0.415700	0	9.762143	-4.000309	-5.762130	L
C-CT--0.025200	-1	8.860416	-3.322340	-6.670473	L
C-C-0.597300	0	8.810301	-1.817988	-6.454858	L
O-O--0.567900	0	8.844475	-1.047899	-7.412021	L
H-H-0.271900	0	9.482090	-4.051101	-4.788767	L
H-H1-0.069800	0	7.853917	-3.714820	-6.528574	L
H-H1-0.069800	0	9.153504	-3.508433	-7.704164	L
N-N--0.415700	0	8.732769	-1.408292	-5.191885	L
C-CT--0.002400	-1	8.586101	-0.017911	-4.779625	L
C-C-0.597300	0	9.712206	0.414396	-3.832830	L
O-O--0.567900	0	10.342037	-0.437082	-3.196911	L
C-CT--0.034300	0	7.207023	0.132044	-4.110526	L
C-CA-0.011800	0	6.044097	-0.046972	-5.068365	L
C-CA--0.125600	0	5.744590	0.960742	-6.005260	L
C-CA--0.125600	0	5.275281	-1.225863	-5.044072	L
C-CA--0.170400	0	4.692533	0.785202	-6.922083	L
C-CA--0.170400	0	4.219377	-1.398002	-5.956605	L
C-CA--0.107200	0	3.932592	-0.397456	-6.901182	L
H-H-0.271900	0	8.719100	-2.113696	-4.464412	L
H-H1-0.097800	0	8.619001	0.626386	-5.658208	L
H-HC-0.029500	0	7.119233	1.117631	-3.654346	L
H-HC-0.029500	0	7.128747	-0.597613	-3.302780	L
H-HA-0.133000	0	6.326748	1.870269	-6.028180	L
H-HA-0.133000	0	5.492412	-2.002485	-4.325495	L
H-HA-0.143000	0	4.465124	1.560711	-7.639593	L
H-HA-0.143000	0	3.621228	-2.296994	-5.927015	L
H-HA-0.129700	0	3.117258	-0.532114	-7.597883	L
N-N--0.254800	0	9.967975	1.728699	-3.688954	L
C-CT--0.026600	-1	10.828283	2.226249	-2.621329	L
C-C-0.589600	0	10.194353	1.977122	-1.243896	L
O-O--0.574800	0	9.002390	1.663037	-1.164902	L
C-CT--0.007000	0	10.984819	3.726188	-2.904693	L
C-CT-0.018900	0	9.668277	4.091480	-3.587846	L
C-CT-0.019200	0	9.364683	2.842803	-4.415429	L
H-H1-0.064100	0	11.799235	1.734867	-2.675168	L
H-HC-0.025300	0	11.810559	3.880905	-3.600674	L
H-HC-0.025300	0	11.139636	4.311714	-1.997104	L
H-HC-0.021300	0	9.764872	4.980529	-4.211807	L
H-HC-0.021300	0	8.889497	4.234282	-2.835969	L
H-H1-0.039100	0	8.287177	2.733741	-4.528511	L
H-H1-0.039100	0	9.835034	2.928913	-5.395756	L
N-N--0.254800	0	10.957805	2.100657	-0.147723	L
C-CT--0.026600	-1	10.402018	2.100658	1.198811	L
C-C-0.589600	0	9.352417	3.190035	1.420247	L
O-O--0.574800	0	9.615506	4.366604	1.182444	L
C-CT--0.007000	0	11.600369	2.291036	2.139535	L
C-CT-0.018900	0	12.788973	1.799442	1.316955	L
C-CT-0.019200	0	12.407261	2.206207	-0.103406	L
H-H1-0.064100	0	9.958769	1.121717	1.386822	L

H-HC-0.025300	0	11.482280	1.717426	3.059396	L		
H-HC-0.025300	0	11.742259	3.348612	2.369645	L		
H-HC-0.021300	0	12.854274	0.711839	1.379527	L		
H-HC-0.021300	0	13.723703	2.262390	1.634165	L		
H-H1-0.039100	0	12.703064	3.239947	-0.287304	L		
H-H1-0.039100	0	12.885663	1.536181	-0.818636	L		
N-N--0.4157	0	8.184257	2.772811	1.897913	L		
C-CT-0.0188	-1	7.028198	3.604402	2.186093	L	H-HC	334
C-C-0.5973	0	6.169081	3.022564	3.305708	L		
O-O--0.5679	0	6.191766	1.815918	3.547195	L		
C-CT--0.515368	0	6.205578	3.710003	0.874684	H		
C-CM-0.287903	0	5.613867	2.424805	0.378475	H		
N-N2--0.582625	0	6.250372	1.617739	-0.550599	H		
C-CM--0.036893	0	4.486079	1.713271	0.716930	H		
C-CM-0.243454	0	5.518593	0.489223	-0.731170	H		
N-N2--0.523998	0	4.443260	0.512683	0.035541	H		
H-H-0.2719	0	8.045869	1.777214	1.995603	L		
H-H1-0.0881	0	7.362411	4.602150	2.486260	L		
H-HC-0.162301	0	6.858979	4.158939	0.112322	H		
H-HC-0.188340	0	5.409383	4.443879	1.049709	H		
H-H-0.339448	0	7.160800	1.816258	-0.965597	H		
H-H4-0.185957	0	3.709026	1.986482	1.415092	H		
H-H5-0.211295	0	5.799045	-0.317818	-1.392573	H		
N-N--0.415700	0	5.394063	3.889696	3.944866	L		
C-CT-0.014300	-1	4.561104	3.612171	5.105733	L		
C-C-0.597300	0	5.208760	2.665863	6.127525	L		
O-O--0.567900	0	4.682819	1.616214	6.501674	L		
C-CT--0.204100	0	3.101202	3.354992	4.708206	L		
C-C-0.713000	0	2.193920	4.228445	5.555814	L		
N-N--0.919100	0	1.636660	3.731134	6.624736	L		
O-O--0.593100	0	2.012420	5.406936	5.305053	L		
H-H-0.271900	0	5.527828	4.875271	3.711821	L		
H-H1-0.104800	0	4.547929	4.571467	5.628375	L		
H-HC-0.079700	0	2.857702	2.305639	4.838542	L		
H-HC-0.079700	0	2.939285	3.625067	3.665141	L		
H-H-0.419600	0	0.992273	4.310771	7.117387	L		
H-H-0.419600	0	1.673787	2.718545	6.741304	L		
N-N--0.4157	0	6.395727	3.097172	6.567509	L		
C-CT--0.1490	-1	7.300401	2.355564	7.424662	L		
H-H-0.2719	0	6.709648	3.968126	6.162235	L		
H-H1-0.0976	0	8.052981	3.021020	7.847048	L		
H-H1-0.0976	0	7.789864	1.570535	6.845095	L		
H-H1-0.0976	0	6.739824	1.879118	8.232076	L		
C-CT--0.3662	-1	-2.263246	-2.677393	9.552962	L		
C-C-0.5972	0	-2.374783	-3.780005	8.536606	L		
O-O--0.5679	0	-2.895748	-4.848943	8.843855	L		
H-HC-0.1123	0	-1.212396	-2.437811	9.703656	L		
H-HC-0.1123	0	-2.706761	-3.009046	10.489300	L		
H-HC-0.1123	0	-2.789514	-1.798808	9.184462	L		
N-N--0.347900	0	-1.859671	-3.521831	7.337861	L		
C-CT--0.240000	-1	-1.759298	-4.494437	6.243412	L		
C-C-0.734100	0	-0.354551	-4.525833	5.653347	L		
O-O--0.589400	0	0.462267	-3.643823	5.901210	L		
C-CT--0.009400	0	-2.866217	-4.223034	5.206922	L		

C-CT-0.018700	0	-2.671046	-2.934789	4.398633	L	H-HC	379
C-CT--0.464524	0	-3.832131	-2.743677	3.417111	H		
C-CT--0.195159	0	-3.760845	-1.444453	2.624449	H		
N-N3--0.748751	0	-2.649623	-1.399642	1.630074	H		
H-H-0.274700	0	-1.479548	-2.589300	7.188487	L		
H-H1-0.142600	0	-1.936066	-5.488551	6.654355	L		
H-HC-0.036200	0	-3.825139	-4.175347	5.725930	L		
H-HC-0.036200	0	-2.917654	-5.053998	4.510497	L		
H-HC-0.010300	0	-1.731889	-2.980583	3.846281	L		
H-HC-0.010300	0	-2.649728	-2.088964	5.078185	L		
H-HC-0.173903	0	-4.765598	-2.744015	3.988081	H		
H-HC-0.148535	0	-3.866347	-3.605390	2.734080	H		
H-HP-0.236066	0	-3.653221	-0.559935	3.253193	H		
H-HP-0.172509	0	-4.679731	-1.321801	2.037430	H		
H-H-0.437293	0	-2.627889	-0.325393	1.149695	H		
H-H-0.366736	0	-2.843596	-2.095229	0.898737	H		
H-H-0.419451	0	-1.711401	-1.575841	2.024270	H		
N-N--0.415700	0	-0.056668	-5.568569	4.891491	L		
C-CT--0.025200	-1	1.260177	-5.780304	4.299243	L		
C-C-0.597300	0	1.135158	-6.505032	2.957983	L		
O-O--0.567900	0	1.715866	-7.565978	2.737238	L		
H-H-0.271900	0	-0.799819	-6.225180	4.671937	L		
H-H1-0.069800	0	1.873196	-6.381900	4.970362	L		
H-H1-0.069800	0	1.759390	-4.826340	4.125950	L		
N-N--0.415700	0	0.324012	-5.920982	2.080021	L		
C-CT--0.025200	-1	-0.249247	-6.526173	0.880940	L		
C-C-0.597300	0	-1.389751	-5.648514	0.434234	L		
O-O--0.567900	0	-2.294422	-5.416236	1.228648	L		
H-H-0.271900	0	-0.109460	-5.063586	2.396976	L		
H-H1-0.069800	0	-0.630832	-7.519907	1.115221	L		
H-H1-0.069800	0	0.512149	-6.593922	0.103413	L		
N-N--0.4157	0	-1.324615	-5.167872	-0.807175	L		
C-CT--0.0014	-1	-2.166711	-4.099703	-1.346339	L		
C-C-0.5973	0	-3.628199	-4.171037	-0.890465	L		
O-O--0.5679	0	-4.180832	-3.210890	-0.365326	L		
C-CT--0.0152	0	-2.076844	-4.108104	-2.887203	L		
C-CA--0.0011	0	-0.688457	-4.283429	-3.479491	L		
C-CA--0.1906	0	0.331250	-3.359876	-3.184131	L		
C-CA--0.1906	0	-0.423058	-5.364501	-4.344037	L		
C-CA--0.2341	0	1.614327	-3.516105	-3.741266	L		
C-CA--0.2341	0	0.858114	-5.525194	-4.905885	L		
C-C-0.3226	0	1.880932	-4.599310	-4.603184	L		
O-OH--0.5579	0	3.112098	-4.754198	-5.158953	L		
H-H-0.2719	0	-0.486559	-5.358542	-1.337631	L		
H-H1-0.0876	0	-1.763444	-3.151540	-0.989473	L		
H-HC-0.0295	0	-2.481928	-3.173018	-3.267345	L		
H-HC-0.0295	0	-2.714600	-4.904464	-3.271219	L		
H-HA-0.1699	0	0.132869	-2.518728	-2.531918	L		
H-HA-0.1699	0	-1.211619	-6.058852	-4.594568	L		
H-HA-0.1656	0	2.390965	-2.801733	-3.508590	L		
H-HA-0.1656	0	1.052752	-6.346448	-5.575709	L		
H-HO-0.3992	0	3.112527	-5.467804	-5.794444	L		
N-N--0.415700	0	-4.235750	-5.347332	-1.060718	L		
C-CT--0.024900	-1	-5.572101	-5.628514	-0.557225	L		

C-C-0.597300	0	-5.701784	-5.550330	0.962285	L
O-O--0.567900	0	-5.091226	-6.327173	1.706374	L
C-CT-0.211700	0	-6.044650	-7.018694	-0.985469	L
O-OH--0.654600	-1	-7.297186	-7.323082	-0.386503	L
H-H-0.271900	0	-3.671283	-6.084914	-1.441416	L
H-H1-0.084300	0	-6.254158	-4.900721	-0.997268	L
H-H1-0.035200	0	-5.310964	-7.760413	-0.667584	L
H-H1-0.035200	0	-6.134068	-7.053065	-2.071270	L
H-HO-0.427500	-1	-7.556908	-8.196635	-0.689398	L
N-N--0.415700	0	-6.665053	-4.736037	1.386499	L
C-CT-0.033700	-1	-7.169387	-4.630200	2.744217	L
C-C-0.597300	0	-7.547958	-5.946425	3.428139	L
O-O--0.567900	0	-7.529661	-6.029659	4.654704	L
C-CT--0.182500	0	-8.345789	-3.653645	2.733419	L
H-H-0.271900	0	-7.104225	-4.142052	0.689487	L
H-H1-0.082300	0	-6.380806	-4.189332	3.352396	L
H-HC-0.060300	0	-8.037968	-2.694791	2.311958	L
H-HC-0.060300	0	-8.695211	-3.499736	3.749637	L
H-HC-0.060300	0	-9.161888	-4.067287	2.146863	L
N-N--0.415700	0	-7.906054	-6.967225	2.648785	L
C-CT--0.025200	-1	-8.305029	-8.270227	3.165890	L
C-C-0.597300	0	-7.338823	-9.410343	2.876120	L
O-O--0.567900	0	-7.738703	-10.572276	2.919737	L
H-H-0.271900	0	-7.857333	-6.814631	1.647631	L
H-H1-0.069800	0	-9.265789	-8.533537	2.724803	L
H-H1-0.069800	0	-8.446443	-8.226541	4.246021	L
N-N--0.415700	0	-6.069412	-9.102471	2.615891	L
C-CT--0.024900	-1	-4.993284	-10.090984	2.655924	L
C-C-0.597300	0	-4.307905	-10.045594	4.015836	L
O-O--0.567900	0	-3.996035	-8.967684	4.531379	L
C-CT-0.211700	0	-3.986474	-9.800011	1.537551	L
O-OH--0.654600	-1	-3.115833	-10.903898	1.343914	L
H-H-0.271900	0	-5.820033	-8.117175	2.600386	L
H-H1-0.084300	0	-5.400131	-11.088414	2.489102	L
H-H1-0.035200	0	-3.411241	-8.903940	1.776345	L
H-H1-0.035200	0	-4.533226	-9.625394	0.610057	L
H-HO-0.427500	-1	-2.574192	-10.721250	0.572606	L
N-N--0.4157	0	-4.103945	-11.220894	4.609886	L
C-CT--0.1490	-1	-3.562767	-11.362901	5.950231	L
H-H-0.2719	0	-4.378495	-12.041631	4.094112	L
H-H1-0.0976	0	-3.343470	-12.409353	6.161869	L
H-H1-0.0976	0	-4.282329	-10.989314	6.682109	L
H-H1-0.0976	0	-2.644221	-10.778612	6.044480	L
C-CT--0.3662	-1	-12.124293	5.119942	-0.687129	L
C-C-0.5972	0	-10.695767	5.307170	-0.248248	L
O-O--0.5679	0	-10.384254	5.048945	0.908870	L
H-HC-0.1123	0	-12.516455	6.068257	-1.047655	L
H-HC-0.1123	0	-12.711240	4.778811	0.163978	L
H-HC-0.1123	0	-12.157014	4.369238	-1.474590	L
N-N--0.415700	0	-9.844257	5.745434	-1.171949	L
C-CT--0.025200	-1	-8.410246	5.899360	-0.941365	L
C-C-0.597300	0	-7.588440	5.835430	-2.204165	L
O-O--0.567900	0	-8.080315	6.200571	-3.278886	L
H-H-0.271900	0	-10.153092	5.905452	-2.118266	L

H-H1-0.069800	0	-8.097635	5.080934	-0.302222	L		
H-H1-0.069800	0	-8.206743	6.841785	-0.434947	L		
N-N--0.415700	0	-6.346337	5.378504	-2.037507	L	H-H1	489
C-CT--0.530209	-1	-5.325934	5.304600	-3.080857	H		
C-C-0.568093	0	-4.665190	3.937377	-2.986784	H		
O-O--0.528105	0	-4.730230	3.259709	-1.960302	H		
C-CT-0.298500	0	-4.332800	6.485122	-2.975430	L	H-H1	489
C-CT--0.319200	0	-5.031545	7.815471	-3.266578	L		
C-CT--0.319200	0	-3.652483	6.560592	-1.606546	L		
H-H-0.271900	0	-6.069787	5.036858	-1.119265	L		
H-H1-0.184096	0	-5.833817	5.406315	-4.047569	H		
H-HC--0.029700	0	-3.559109	6.356477	-3.730939	L		
H-HC-0.079100	0	-5.756034	8.035423	-2.484256	L		
H-HC-0.079100	0	-5.542692	7.754490	-4.226034	L		
H-HC-0.079100	0	-4.289251	8.611648	-3.303078	L		
H-HC-0.079100	0	-4.387638	6.694936	-0.815025	L		
H-HC-0.079100	0	-2.950422	7.391123	-1.587855	L		
H-HC-0.079100	0	-3.104834	5.635326	-1.417834	L		
N-N--0.610103	0	-3.984711	3.531183	-4.089644	H		
C-CT--0.314060	-1	-4.215694	3.996668	-5.449806	H		
C-C-0.597300	0	-5.329588	3.181576	-6.093908	L	H-H1	505
O-O--0.567900	0	-5.455454	1.976006	-5.879518	L		
C-CT--0.110200	0	-2.906988	3.855588	-6.264722	L	H-H1	505
C-CT-0.353100	0	-1.798100	4.879537	-5.920821	L		
C-CT--0.412100	0	-1.020118	4.579361	-4.637132	L		
C-CT--0.412100	0	-0.773760	4.916407	-7.060227	L		
H-H-0.398207	0	-3.590075	2.601291	-4.010219	H		
H-H1-0.165130	0	-4.497946	5.047466	-5.453696	H		
H-HC-0.045700	0	-3.165521	3.986866	-7.315449	L		
H-HC-0.045700	0	-2.512032	2.845765	-6.150256	L		
H-HC--0.036100	0	-2.242440	5.872424	-5.843736	L		
H-HC-0.100000	0	-0.617770	3.568198	-4.666352	L		
H-HC-0.100000	0	-1.681609	4.650130	-3.773576	L		
H-HC-0.100000	0	-0.213263	5.296857	-4.501278	L		
H-HC-0.100000	0	-0.302256	3.939772	-7.160167	L		
H-HC-0.100000	0	-0.010979	5.662887	-6.839728	L		
H-HC-0.100000	0	-1.267260	5.183107	-7.992953	L		
N-N--0.415700	0	-6.118082	3.829148	-6.944247	L		
C-CT--0.038900	-1	-7.307720	3.225719	-7.556632	L		
C-C-0.597300	0	-7.065906	1.886016	-8.242066	L		
O-O--0.567900	0	-7.948454	1.030870	-8.280889	L		
C-CT-0.365400	-1	-7.941683	4.146647	-8.623378	L		
O-OH--0.676100	-1	-7.876479	5.505881	-8.182143	L		
C-CT--0.243800	0	-9.420992	3.860009	-8.881154	L		
H-H-0.271900	0	-5.998944	4.827803	-7.029177	L		
H-H1-0.100700	0	-8.046901	3.090155	-6.773998	L		
H-H1-0.004300	0	-7.386544	4.064339	-9.558821	L		
H-HC-0.064200	0	-9.539749	2.854540	-9.284489	L		
H-HC-0.064200	0	-9.812816	4.571638	-9.607661	L		
H-HC-0.064200	0	-9.984326	3.937451	-7.950422	L		
H-HO-0.410200	-1	-8.773753	5.843781	-8.148951	L		
N-N--0.415700	0	-5.891588	1.746613	-8.853858	L		
C-CT--0.025200	-1	-5.506628	0.581017	-9.635857	L		
C-C-0.597300	0	-4.625579	-0.423147	-8.924781	L		

O-O--0.567900	0	-4.170666	-1.360325	-9.581695	L		
H-H-0.271900	0	-5.204298	2.461897	-8.669805	L		
H-H1-0.069800	0	-4.965533	0.917117	-10.519106	L		
H-H1-0.069800	0	-6.395960	0.050745	-9.976378	L		
N-N--0.415700	0	-4.376879	-0.213828	-7.625694	L	H-H1	545
C-CT--0.539097	-1	-3.469802	-1.021820	-6.822436	H		
C-C-0.572106	0	-4.213549	-1.486888	-5.568863	H		
O-O--0.533001	0	-4.894880	-2.507637	-5.632034	H		
C-CT-0.130300	0	-2.075484	-0.365213	-6.683737	L	H-H1	545
C-CT--0.043000	0	-1.458459	0.015805	-8.050692	L		
C-CT--0.320400	0	-1.179779	-1.384175	-5.956951	L		
C-CT--0.066000	0	-0.091414	0.706757	-7.960760	L		
H-H-0.271900	0	-4.816233	0.575906	-7.160651	L		
H-H1-0.180646	0	-3.311430	-1.952159	-7.380742	H		
H-HC-0.018700	0	-2.167468	0.541572	-6.097323	L		
H-HC-0.023600	0	-2.124225	0.713959	-8.556418	L		
H-HC-0.023600	0	-1.366478	-0.877272	-8.670036	L		
H-HC-0.088200	0	-1.110315	-2.304223	-6.535630	L		
H-HC-0.088200	0	-1.592615	-1.614447	-4.976388	L		
H-HC-0.088200	0	-0.181972	-0.985596	-5.799222	L		
H-HC-0.018600	0	-0.150974	1.554702	-7.279344	L		
H-HC-0.018600	0	0.195896	1.065205	-8.949234	L		
H-HC-0.018600	0	0.668626	0.008989	-7.612265	L		
N-N--0.611145	0	-4.111858	-0.711899	-4.463810	H		
C-CT--0.109296	-1	-4.637005	-1.101950	-3.160734	H		
C-C-0.597300	0	-6.165843	-1.105283	-3.127717	L	H-H1	564
O-O--0.567900	0	-6.798609	-2.033074	-2.609549	L		
C-CT--0.388038	0	-3.999193	-0.247648	-2.076252	H		
H-H-0.369964	0	-3.530233	0.118231	-4.465947	H		
H-H1-0.178541	0	-4.378552	-2.148766	-2.975643	H		
H-H1-0.157964	0	-4.418471	-0.524828	-1.104787	H		
H-H1-0.179833	0	-4.158512	0.827179	-2.198115	H		
H-HS-0.319500	0	-1.891429	2.012492	-0.610596	H		
N-N--0.415700	0	-6.756588	-0.118162	-3.790678	L		
C-CT--0.087500	-1	-8.190129	-0.056356	-4.064392	L		
C-C-0.597300	0	-8.667189	-1.216773	-4.923497	L		
O-O--0.567900	0	-9.608952	-1.913890	-4.546056	L		
C-CT-0.298500	0	-8.573258	1.300257	-4.674552	L		
C-CT--0.319200	0	-10.085317	1.429373	-4.905119	L		
C-CT--0.319200	0	-8.143663	2.485680	-3.795827	L		
H-H-0.271900	0	-6.113851	0.558732	-4.193317	L		
H-H1-0.096900	0	-8.710177	-0.141793	-3.113066	L		
H-HC--0.029700	0	-8.073778	1.374495	-5.631908	L		
H-HC-0.079100	0	-10.616136	1.291440	-3.962865	L		
H-HC-0.079100	0	-10.421034	0.681003	-5.622708	L		
H-HC-0.079100	0	-10.312630	2.415808	-5.308167	L		
H-HC-0.079100	0	-8.619614	2.431766	-2.818664	L		
H-HC-0.079100	0	-8.406972	3.428885	-4.273585	L		
H-HC-0.079100	0	-7.062623	2.482236	-3.651718	L		
N-N--0.415700	0	-8.034099	-1.431613	-6.075336	L		
C-CT--0.025200	-1	-8.405124	-2.487623	-7.015012	L		
C-C-0.597300	0	-8.316919	-3.889046	-6.423718	L		
O-O--0.567900	0	-9.213922	-4.705645	-6.623492	L		
H-H-0.271900	0	-7.239939	-0.839575	-6.283526	L		

H-H1-0.069800	0	-7.743671	-2.440082	-7.879792	L
H-H1-0.069800	0	-9.427742	-2.321427	-7.353154	L
N-N--0.415700	0	-7.237961	-4.162717	-5.692350	L
C-CT--0.025200	-1	-7.020772	-5.424046	-4.995248	L
C-C-0.597300	0	-7.956059	-5.674943	-3.823730	L
O-O--0.567900	0	-8.299518	-6.824015	-3.550877	L
H-H-0.271900	0	-6.520829	-3.442107	-5.623475	L
H-H1-0.069800	0	-5.998381	-5.442247	-4.619282	L
H-H1-0.069800	0	-7.139254	-6.244768	-5.702780	L
N-N--0.415700	0	-8.337623	-4.613708	-3.116394	L
C-CT--0.038900	-1	-9.390854	-4.664892	-2.100005	L
C-C-0.597300	0	-10.760773	-4.958450	-2.702755	L
O-O--0.567900	0	-11.505651	-5.791932	-2.186408	L
C-CT-0.365400	0	-9.427590	-3.360540	-1.289267	L
O-OH--0.676100	0	-8.194972	-3.198740	-0.626378	L
C-CT--0.243800	0	-10.519781	-3.363734	-0.219861	L
H-H-0.271900	0	-7.941717	-3.712082	-3.359938	L
H-H1-0.100700	0	-9.166000	-5.477394	-1.409215	L
H-H1-0.004300	0	-9.586467	-2.510481	-1.952906	L
H-HC-0.064200	0	-11.497392	-3.256030	-0.688337	L
H-HC-0.064200	0	-10.365790	-2.530753	0.465649	L
H-HC-0.064200	0	-10.491600	-4.300609	0.334892	L
H-HO-0.410200	0	-7.603690	-2.728568	-1.250225	L
N-N--0.4157	0	-11.108731	-4.254260	-3.780886	L
C-CT--0.1490	-1	-12.341156	-4.446560	-4.517117	L
H-H-0.2719	0	-10.440145	-3.572710	-4.129496	L
H-H1-0.0976	0	-12.414026	-3.698056	-5.307052	L
H-H1-0.0976	0	-13.193434	-4.354752	-3.842512	L
H-H1-0.0976	0	-12.341695	-5.441876	-4.964583	L
N-N2--0.511415	0	2.026742	-0.084289	-1.739357	H
C-CA-0.057801	0	0.709052	0.121927	-1.764682	H
C-CA-0.063496	0	0.035892	0.555546	-2.912182	H
C-CA--0.109122	0	0.785768	0.887290	-4.039272	H
C-CA--0.164490	0	2.160629	0.691394	-4.006461	H
C-CA-0.098392	0	2.734284	0.172142	-2.850875	H
C-C-0.541263	0	-1.447888	0.599337	-2.994896	H
O-O--0.574884	0	-2.037132	1.279380	-3.823377	H
H-H4-0.220889	0	0.178604	-0.050633	-0.835991	H
H-HA-0.160434	0	0.281294	1.270329	-4.918944	H
H-HA-0.142191	0	2.783914	0.931493	-4.860544	H
H-H4-0.174502	0	3.794880	-0.044617	-2.800950	H
H-H-0.353801	0	-0.827509	3.128869	-0.735058	H
Zn-ZN-0.974537	0	2.867743	-1.109148	0.027207	H
O-OW--0.834000	0	-2.151854	-7.214882	3.529234	L
H-HW-0.417000	0	-2.578451	-6.628256	2.884524	L
H-HW-0.417000	0	-2.885102	-7.748165	3.876875	L
O-OW--0.783903	0	1.295728	-2.544789	-0.132150	H
H-HW-0.401253	0	0.879913	-2.422104	0.760948	H
H-HW-0.424111	0	1.889648	-3.337120	-0.156527	H
O-OW--0.834000	0	-3.713306	-8.395916	7.275157	L
H-HW-0.417000	0	-3.761263	-7.450361	7.437768	L
H-HW-0.417000	0	-3.836756	-8.475638	6.317007	L
O-OW--0.834000	0	7.190253	-0.018876	1.832521	L
H-HW-0.417000	0	6.884920	0.393468	2.660892	L

```
H-HW-0.417000   0      6.403982    0.078627    1.272240 L
O-OW--0.834000  0      7.037736   -4.940590    4.802562 L
H-HW-0.417000   0      6.087495   -4.993455    5.032810 L
H-HW-0.417000   0      7.235816   -5.821987    4.483899 L
O-OW--0.834000  0      8.645045   -0.947844   -0.217515 L
H-HW-0.417000   0      8.181627   -0.569385    0.557191 L
H-HW-0.417000   0      8.854766   -0.161901   -0.742562 L
O-OW--0.834000  0     12.656638   -1.110824   -1.973116 L
H-HW-0.417000   0     11.806299   -0.859927   -2.373301 L
H-HW-0.417000   0     12.657374   -2.074883   -2.076199 L
O-OW--0.834000  0      8.281857   -3.315793    1.021305 L
H-HW-0.417000   0      8.497381   -2.547424    0.452550 L
H-HW-0.417000   0      7.549457   -2.977574    1.546828 L
O-OW--0.834000  0      4.348194   -4.887014    5.203913 L
H-HW-0.417000   0      4.080691   -4.496393    6.058853 L
H-HW-0.417000   0      4.031940   -4.231525    4.571749 L
S-SH--0.326142  0     -2.205117   -0.603117   -1.934323 H
O-OW--0.834000  0     -1.371587    2.523892   -1.267273 H
```

TS3

```
C-CT--0.366200  -1  -10.306119    3.933744    4.023850 L
C-C-0.597200     0   -8.977936    3.280913    3.775973 L
O-O--0.567900    0   -8.400042    2.714402    4.696946 L
H-HC-0.112300    0  -11.051502    3.473234    3.378533 L
H-HC-0.112300    0  -10.225664    4.995115    3.795670 L
H-HC-0.112300    0  -10.581807    3.797050    5.067062 L
N-N--0.415700    0   -8.520942    3.365256    2.533192 L
C-CT--0.059700  -1   -7.339508    2.662939    2.025558 L
C-C-0.597300     0   -6.196625    3.651146    1.848466 L
O-O--0.567900    0   -6.359044    4.661982    1.170209 L
C-CT-0.130300    0   -7.666195    1.908987    0.711860 L
C-CT--0.043000   0   -8.869232    0.949008    0.898282 L
C-CT--0.320400   0   -6.437089    1.112218    0.237845 L
C-CT--0.066000   0   -9.373006    0.310917   -0.400792 L
H-H-0.271900     0   -9.055976    3.928289    1.880607 L
H-H1-0.086900    0   -7.028142    1.918347    2.758021 L
H-HC-0.018700    0   -7.921119    2.642548   -0.055225 L
H-HC-0.023600    0   -9.713356    1.494423    1.318279 L
H-HC-0.023600    0   -8.598904    0.158418    1.599468 L
H-HC-0.088200    0   -6.166505    0.348064    0.966692 L
H-HC-0.088200    0   -5.582296    1.773827    0.090170 L
H-HC-0.088200    0   -6.633327    0.644578   -0.724896 L
H-HC-0.018600    0   -9.588559    1.085927   -1.135007 L
H-HC-0.018600    0  -10.289676   -0.241347   -0.197448 L
H-HC-0.018600    0   -8.630755   -0.380234   -0.796847 L
N-N--0.516300    0   -5.080286    3.365030    2.513020 L
C-CT-0.038100   -1   -3.771439    3.991601    2.391174 L H-HC 30
C-C-0.536600     0   -3.679602    5.513782    2.164892 L
O-O--0.581900    0   -2.678800    6.038006    1.672647 L
C-CT--0.547145   0   -3.004066    3.206507    1.314067 H
C-C-0.532653     0   -2.510152    1.856727    1.778474 H
O-O2--0.588507   0   -2.670320    1.441328    2.924015 H
O-O2--0.625559   0   -1.853678    1.150879    0.878342 H
H-H-0.293600     0   -5.052899    2.474433    2.993656 L
```

H-H1-0.088000	0	-3.248610	3.830757	3.334413	L
H-HC-0.128435	0	-2.105744	3.774066	1.026382	H
H-HC-0.183556	0	-3.591387	3.137076	0.391554	H
N-N--0.415700	0	-4.661684	6.267449	2.638878	L
C-CT--0.025200	-1	-4.642367	7.711161	2.518081	L
C-C-0.597300	0	-3.748116	8.353949	3.564539	L
O-O--0.567900	0	-4.225956	9.010364	4.491208	L
H-H-0.271900	0	-5.498284	5.766012	2.895186	L
H-H1-0.069800	0	-5.654423	8.092282	2.645658	L
H-H1-0.069800	0	-4.292007	7.997135	1.525057	L
N-N--0.415700	0	-2.444040	8.150724	3.413477	L
C-CT--0.038900	-1	-1.418943	8.566832	4.360266	L
C-C-0.597300	0	-0.521482	9.666808	3.780034	L
O-O--0.567900	0	-0.640386	10.068258	2.620974	L
C-CT-0.365400	0	-0.554782	7.398067	4.879680	L
O-OH--0.676100	0	0.573978	7.211607	4.063686	L
C-CT--0.243800	0	-1.282882	6.065982	5.029643	L
H-H-0.271900	0	-2.178703	7.555017	2.631204	L
H-H1-0.100700	0	-1.916840	8.993749	5.229036	L
H-H1-0.004300	0	-0.189403	7.682071	5.867144	L
H-HC-0.064200	0	-2.202571	6.209156	5.595134	L
H-HC-0.064200	0	-0.641801	5.365157	5.558145	L
H-HC-0.064200	0	-1.514345	5.650083	4.049477	L
H-HO-0.410200	0	1.092400	6.463884	4.432183	L
N-N--0.415700	0	0.415125	10.152828	4.598792	L
C-CT--0.025200	-1	1.452444	11.089973	4.178267	L
C-C-0.597300	0	2.344155	10.599547	3.046035	L
O-O--0.567900	0	2.664505	11.370444	2.146426	L
H-H-0.271900	0	0.490301	9.735074	5.510743	L
H-H1-0.069800	0	2.093705	11.322913	5.027181	L
H-H1-0.069800	0	0.977923	12.014097	3.847331	L
N-N--0.516300	0	2.749549	9.333728	3.108121	L
C-CT-0.038100	-1	3.655832	8.732414	2.131248	L
C-C-0.536600	0	3.022424	8.435642	0.785885	L
O-O--0.581900	0	3.757568	8.157017	-0.154075	L
C-CT--0.030300	0	4.237594	7.418318	2.683081	L
C-C-0.799400	-1	5.584451	7.615870	3.359005	L
O-O2--0.801400	-1	6.166313	8.719304	3.278291	L
O-O2--0.801400	-1	6.042786	6.661383	4.019386	L
H-H-0.293600	0	2.388763	8.732658	3.834250	L
H-H1-0.088000	0	4.459287	9.437167	1.913992	L
H-HC--0.012200	0	4.392008	6.702403	1.873960	L
H-HC--0.012200	0	3.534039	6.964282	3.383646	L
N-N--0.415700	0	1.698086	8.491178	0.677935	L
C-CT--0.002400	-1	0.986169	8.327682	-0.583059	L
C-C-0.597300	0	0.425465	9.639804	-1.129433	L
O-O--0.567900	0	0.411332	9.855032	-2.341522	L
C-CT--0.034300	0	-0.125834	7.281434	-0.427985	L
C-CA-0.011800	0	0.352699	5.870729	-0.129346	L
C-CA--0.125600	0	0.757031	5.020342	-1.175793	L
C-CA--0.125600	0	0.335911	5.382736	1.189451	L
C-CA--0.170400	0	1.126195	3.691784	-0.899007	L
C-CA--0.170400	0	0.711330	4.057792	1.468542	L
C-CA--0.107200	0	1.101032	3.210438	0.419697	L

H-H-0.271900	0	1.175109	8.665837	1.523571	L
H-H1-0.097800	0	1.667893	7.948132	-1.343859	L
H-HC-0.029500	0	-0.689012	7.246095	-1.360719	L
H-HC-0.029500	0	-0.814701	7.603317	0.354600	L
H-HA-0.133000	0	0.749044	5.374900	-2.195140	L
H-HA-0.133000	0	-0.022863	6.014807	1.982603	L
H-HA-0.143000	0	1.394583	3.018839	-1.697699	L
H-HA-0.143000	0	0.647110	3.676706	2.476700	L
H-HA-0.129700	0	1.343052	2.177778	0.618577	L
N-N--0.415700	0	-0.027627	10.532750	-0.248211	L
C-CT--0.149000	-1	-0.457366	11.863740	-0.623661	L
H-H-0.271900	0	-0.027729	10.274225	0.733211	L
H-H1-0.097600	0	-0.833167	12.386767	0.256251	L
H-H1-0.097600	0	-1.247468	11.795571	-1.373341	L
H-H1-0.097600	0	0.387121	12.414076	-1.041137	L
C-CT--0.366200	-1	7.996299	8.432729	-0.147245	L
C-C-0.597200	0	7.532259	7.639523	-1.345183	L
O-O--0.567900	0	8.354149	7.285855	-2.187189	L
H-HC-0.112300	0	7.562233	9.429433	-0.182632	L
H-HC-0.112300	0	9.081900	8.496717	-0.158057	L
H-HC-0.112300	0	7.675749	7.929314	0.765795	L
N-N--0.415700	0	6.220629	7.404176	-1.445693	L
C-CT--0.051800	-1	5.573190	6.861676	-2.648296	L
C-C-0.597300	0	4.899234	7.958262	-3.473318	L
O-O--0.567900	0	5.210167	8.162341	-4.646838	L
C-CT--0.110200	0	4.657429	5.662416	-2.321070	L
C-CT-0.353100	0	4.897859	4.483727	-3.287002	L
C-CT--0.412100	0	3.995011	3.306657	-2.943849	L
C-CT--0.412100	0	4.625863	4.823160	-4.756585	L
H-H-0.271900	0	5.615950	7.727884	-0.697976	L
H-H1-0.092200	0	6.372916	6.483393	-3.285494	L
H-HC-0.045700	0	3.609777	5.964721	-2.356340	L
H-HC-0.045700	0	4.867184	5.314901	-1.308943	L
H-HC--0.036100	0	5.933526	4.154691	-3.189877	L
H-HC-0.100000	0	2.963933	3.544940	-3.200766	L
H-HC-0.100000	0	4.053057	3.085383	-1.879160	L
H-HC-0.100000	0	4.314505	2.428700	-3.501655	L
H-HC-0.100000	0	3.625686	5.245245	-4.859731	L
H-HC-0.100000	0	4.701198	3.924411	-5.366648	L
H-HC-0.100000	0	5.355948	5.545607	-5.118197	L
N-N--0.415700	0	4.005500	8.687007	-2.818604	L
C-CT--0.149000	-1	3.370319	9.895449	-3.274452	L
H-H-0.271900	0	3.848364	8.406391	-1.858686	L
H-H1-0.097600	0	4.072130	10.485998	-3.864582	L
H-H1-0.097600	0	3.035213	10.480896	-2.417038	L
H-H1-0.097600	0	2.506698	9.643147	-3.891994	L
C-CT--0.366200	-1	-8.723401	2.384392	8.131190	L
C-C-0.597200	0	-7.861133	1.167452	7.951734	L
O-O--0.567900	0	-7.553784	0.479054	8.918411	L
H-HC-0.112300	0	-9.639239	2.255469	7.557618	L
H-HC-0.112300	0	-8.182682	3.257605	7.769296	L
H-HC-0.112300	0	-8.957233	2.503181	9.186963	L
N-N--0.415700	0	-7.464829	0.919564	6.711718	L
C-CT--0.038900	-1	-6.664560	-0.234509	6.297963	L

```
C-C-0.597300      0   -5.398762    0.250189    5.614689 L
O-O--0.567900     0   -5.440597    0.681982    4.463925 L
C-CT-0.365400     0   -7.472233   -1.199439    5.416484 L
O-OH--0.676100    0   -8.034757   -0.532649    4.312453 L
C-CT--0.243800    0   -8.615779   -1.828320    6.218094 L
H-H-0.271900      0   -7.784455    1.546283    5.976645 L
H-H1-0.100700     0   -6.363320   -0.798048    7.180596 L
H-H1-0.004300     0   -6.812923   -1.991254    5.058825 L
H-HC-0.064200     0   -8.212240   -2.363562    7.077206 L
H-HC-0.064200     0   -9.170026   -2.525444    5.595830 L
H-HC-0.064200     0   -9.303594   -1.056310    6.561379 L
H-HO-0.410200     0   -7.342222    0.045565    3.960174 L
N-N--0.415700     0   -4.305912    0.239313    6.382559 L
C-CT--0.059700   -1   -3.109610    1.056176    6.164584 L
C-C-0.597300      0   -1.863797    0.178008    6.089070 L
O-O--0.567900     0   -1.603485   -0.619940    6.994949 L
C-CT-0.130300     0   -2.988840    2.131688    7.269520 L
C-CT--0.043000    0   -4.245157    3.025441    7.422328 L
C-CT--0.320400    0   -1.748602    3.007410    7.037029 L
C-CT--0.066000    0   -4.666243    3.813509    6.172730 L
H-H-0.271900      0   -4.373550   -0.200724    7.285472 L
H-H1-0.086900     0   -3.211972    1.578134    5.215873 L
H-HC-0.018700     0   -2.848841    1.618995    8.223070 L
H-HC-0.023600     0   -4.065860    3.738980    8.227312 L
H-HC-0.023600     0   -5.085941    2.407339    7.735976 L
H-HC-0.088200     0   -1.742214    3.370613    6.008994 L
H-HC-0.088200     0   -0.848169    2.414908    7.200417 L
H-HC-0.088200     0   -1.739261    3.848869    7.728504 L
H-HC-0.018600     0   -3.864397    4.475583    5.850306 L
H-HC-0.018600     0   -5.544926    4.415000    6.405838 L
H-HC-0.018600     0   -4.920185    3.134475    5.361721 L
N-N--0.516300     0   -1.115910    0.349472    5.003019 L
C-CT-0.038100    -1    0.185138   -0.233179    4.729166 L H-HC 17
C-C-0.536600      0    1.118417   -0.078321    5.936269 L
O-O--0.581900     0    1.326063    1.012883    6.462222 L
C-CT--0.520019    0    0.695706    0.410638    3.426239 H
C-C-0.636404      0    0.956521   -0.550734    2.277143 H
O-O2--0.608036    0    0.320309   -1.644744    2.184450 H
O-O2--0.608131    0    1.817626   -0.132931    1.440583 H
H-H-0.293600      0   -1.516388    0.930367    4.271015 L
H-H1-0.088000     0    0.033136   -1.301441    4.567955 L
H-HC-0.132300     0    1.624854    0.947727    3.629362 H
H-HC-0.188993     0   -0.019352    1.155372    3.062528 H
N-N--0.415700     0    1.666100   -1.190693    6.394178 L
C-CT-0.033700    -1    2.567110   -1.241234    7.523002 L
C-C-0.597300      0    3.788602   -2.061506    7.124034 L
O-O--0.567900     0    3.841499   -3.285984    7.285776 L
C-CT--0.182500    0    1.810389   -1.801173    8.735784 L
H-H-0.271900      0    1.363021   -2.066929    5.983714 L
H-H1-0.082300     0    2.912245   -0.236747    7.775663 L
H-HC-0.060300     0    0.970639   -1.146482    8.972261 L
H-HC-0.060300     0    2.480982   -1.853062    9.593172 L
H-HC-0.060300     0    1.433412   -2.800055    8.510393 L
N-N--0.415700     0    4.756904   -1.367716    6.537892 L
```

```
C-CT-0.018800   -1    5.962273   -1.951947    5.994375 L H-HC 20
C-C-0.597300     0    7.158725   -1.806308    6.944064 L
O-O--0.567900    0    7.207571   -0.956626    7.835957 L
C-CT--0.517456   0    6.219195   -1.317694    4.625451 H
C-CM-0.287351    0    5.350925   -1.797942    3.521116 H
N-N2--0.586994   0    5.495261   -3.052271    2.944196 H
C-CM--0.020338   0    4.373720   -1.189690    2.783939 H
C-CM-0.242458    0    4.631266   -3.142446    1.898279 H
N-N2--0.505070   0    3.942232   -2.023344    1.772247 H
H-H-0.271900     0    4.649472   -0.359071    6.473609 L
H-H1-0.088100    0    5.813959   -3.017969    5.853913 L
H-HC-0.166355    0    7.274598   -1.484394    4.364156 H
H-HC-0.174481    0    6.106363   -0.238940    4.758845 H
H-H-0.335795     0    6.141623   -3.769519    3.266072 H
H-H4-0.152777    0    3.973302   -0.194581    2.905239 H
H-H5-0.209475    0    4.533419   -4.008308    1.258196 H
N-N--0.415700    0    8.161921   -2.646932    6.697759 L
C-CT--0.149000  -1    9.432050   -2.577583    7.387085 L
H-H-0.271900     0    8.008419   -3.325163    5.958162 L
H-H1-0.097600    0   10.103072   -3.348897    7.007957 L
H-H1-0.097600    0    9.275041   -2.723730    8.457152 L
H-H1-0.097600    0    9.880623   -1.595327    7.227790 L
C-CT--0.366200  -1    8.348637   -9.389873    0.921055 L
C-C-0.597200     0    8.729052   -9.163669   -0.529802 L
O-O--0.567900    0    9.765974   -8.564895   -0.788603 L
H-HC-0.112300    0    8.265072  -10.455655    1.120017 L
H-HC-0.112300    0    7.410457   -8.881081    1.135145 L
H-HC-0.112300    0    9.127758   -8.962616    1.552207 L
N-N--0.254800    0    7.908530   -9.617328   -1.493998 L
C-CT--0.026600  -1    8.150870   -9.378200   -2.919003 L
C-C-0.589600     0    8.006147   -7.904211   -3.297659 L
O-O--0.574800    0    8.721371   -7.407836   -4.164913 L
C-CT--0.007000   0    7.139661  -10.266393   -3.651630 L
C-CT-0.018900    0    5.979859  -10.388318   -2.661916 L
C-CT-0.019200    0    6.673059  -10.364452   -1.298778 L
H-H1-0.064100    0    9.161894   -9.692172   -3.181603 L
H-HC-0.025300    0    7.579172  -11.251393   -3.815857 L
H-HC-0.025300    0    6.819794   -9.833307   -4.600692 L
H-HC-0.021300    0    5.415454  -11.309248   -2.811248 L
H-HC-0.021300    0    5.324371   -9.520968   -2.754851 L
H-H1-0.039100    0    6.027786   -9.887816   -0.559451 L
H-H1-0.039100    0    6.912490  -11.382601   -0.989085 L
N-N--0.516300    0    7.082763   -7.207580   -2.634213 L
C-CT-0.039700   -1    6.830922   -5.780576   -2.805257 L
C-C-0.536600     0    8.091666   -4.917146   -2.675369 L
O-O--0.581900    0    8.269889   -3.934592   -3.398836 L
C-CT-0.056000    0    5.733449   -5.343116   -1.831559 L H-HC 24
C-CT--0.515956   0    5.203428   -3.960341   -2.218790 H
C-C-0.587828     0    4.122210   -3.415033   -1.296973 H
O-O2--0.600319   0    4.076935   -2.133558   -1.260305 H
O-O2--0.589127   0    3.362768   -4.197971   -0.680055 H
H-H-0.293600     0    6.545421   -7.684834   -1.929735 L
H-H1-0.110500    0    6.455888   -5.626607   -3.817477 L
H-HC--0.017300   0    6.129320   -5.318195   -0.815095 L
```

H-HC--0.017300	0	4.911300	-6.059382	-1.875412	L
H-HC-0.167759	0	4.790927	-4.017934	-3.233031	H
H-HC-0.135230	0	6.027142	-3.238871	-2.244273	H
N-N--0.415700	0	9.000082	-5.323876	-1.791245	L
C-CT--0.024900	-1	10.240462	-4.625637	-1.474889	L
C-C-0.597300	0	11.263290	-4.516405	-2.598925	L
O-O--0.567900	0	12.246981	-3.788615	-2.462801	L
C-CT-0.211700	0	10.879665	-5.265359	-0.236109	L
O-OH--0.654600	0	9.922766	-5.460304	0.794854	L
H-H-0.271900	0	8.787200	-6.131331	-1.222117	L
H-H1-0.084300	0	9.975992	-3.601480	-1.207173	L
H-H1-0.035200	0	11.678055	-4.618186	0.129915	L
H-H1-0.035200	0	11.306844	-6.232154	-0.506731	L
H-HO-0.427500	0	9.370182	-4.650987	0.876899	L
N-N--0.415700	0	11.021927	-5.197188	-3.717391	L
C-CT-0.033700	-1	11.773268	-5.033756	-4.955072	L
C-C-0.597300	0	10.943112	-4.473733	-6.114706	L
O-O--0.567900	0	11.375815	-4.487713	-7.266377	L
C-CT--0.182500	0	12.397809	-6.395922	-5.290791	L
H-H-0.271900	0	10.205062	-5.797646	-3.727171	L
H-H1-0.082300	0	12.590130	-4.327828	-4.799077	L
H-HC-0.060300	0	13.012388	-6.736364	-4.456707	L
H-HC-0.060300	0	13.025315	-6.304757	-6.178014	L
H-HC-0.060300	0	11.610352	-7.126573	-5.482589	L
N-N--0.415700	0	9.765812	-3.951382	-5.789130	L
C-CT--0.025200	-1	8.861061	-3.271691	-6.693119	L
C-C-0.597300	0	8.805555	-1.768610	-6.469840	L
O-O--0.567900	0	8.833646	-0.993349	-7.422983	L
H-H-0.271900	0	9.487452	-4.006872	-4.815548	L
H-H1-0.069800	0	7.856096	-3.668572	-6.552625	L
H-H1-0.069800	0	9.154058	-3.451307	-7.727982	L
N-N--0.415700	0	8.730345	-1.365978	-5.204626	L
C-CT--0.002400	-1	8.582707	0.021457	-4.783274	L
C-C-0.597300	0	9.714228	0.450618	-3.840878	L
O-O--0.567900	0	10.352417	-0.403463	-3.216888	L
C-CT--0.034300	0	7.210505	0.161704	-4.098100	L
C-CA-0.011800	0	6.035807	-0.012046	-5.042060	L
C-CA--0.125600	0	5.708552	1.011528	-5.951807	L
C-CA--0.125600	0	5.281365	-1.200194	-5.029759	L
C-CA--0.170400	0	4.641489	0.843689	-6.852533	L
C-CA--0.170400	0	4.211322	-1.365454	-5.926868	L
C-CA--0.107200	0	3.896064	-0.348275	-6.844051	L
H-H-0.271900	0	8.721623	-2.075520	-4.481167	L
H-H1-0.097800	0	8.605100	0.672022	-5.657603	L
H-HC-0.029500	0	7.125425	1.143005	-3.632245	L
H-HC-0.029500	0	7.143540	-0.574662	-3.295388	L
H-HA-0.133000	0	6.280524	1.927633	-5.965471	L
H-HA-0.133000	0	5.521749	-1.989158	-4.332566	L
H-HA-0.143000	0	4.392286	1.631576	-7.549046	L
H-HA-0.143000	0	3.625351	-2.272754	-5.908114	L
H-HA-0.129700	0	3.070565	-0.478281	-7.529646	L
N-N--0.254800	0	9.963727	1.764721	-3.685007	L
C-CT--0.026600	-1	10.822665	2.256769	-2.613518	L
C-C-0.589600	0	10.188951	1.997922	-1.237729	L

O-O--0.574800	0	8.996890	1.683176	-1.160667	L	
C-CT--0.007000	0	10.975204	3.758955	-2.886994	L	
C-CT-0.018900	0	9.656191	4.125763	-3.564664	L	
C-CT-0.019200	0	9.353807	2.882313	-4.400569	L	
H-H1-0.064100	0	11.794869	1.768236	-2.670439	L	
H-HC-0.025300	0	11.798997	3.920304	-3.583778	L	
H-HC-0.025300	0	11.130750	4.338658	-1.975800	L	
H-HC-0.021300	0	9.749170	5.019507	-4.182435	L	
H-HC-0.021300	0	8.878715	4.261256	-2.810097	L	
H-H1-0.039100	0	8.276258	2.770289	-4.510501	L	
H-H1-0.039100	0	9.820137	2.977554	-5.381968	L	
N-N--0.254800	0	10.953022	2.115599	-0.141141	L	
C-CT--0.026600	-1	10.398875	2.108706	1.206093	L	
C-C-0.589600	0	9.347104	3.194733	1.433630	L	
O-O--0.574800	0	9.609566	4.373658	1.206977	L	
C-CT--0.007000	0	11.597875	2.296437	2.146502	L	
C-CT-0.018900	0	12.785865	1.808799	1.320771	L	
C-CT-0.019200	0	12.402560	2.221398	-0.097485	L	
H-H1-0.064100	0	9.957571	1.128070	1.389925	L	
H-HC-0.025300	0	11.481077	1.719169	3.064239	L	
H-HC-0.025300	0	11.739307	3.353202	2.380538	L	
H-HC-0.021300	0	12.851691	0.720971	1.378711	L	
H-HC-0.021300	0	13.720704	2.270817	1.639014	L	
H-H1-0.039100	0	12.697946	3.255968	-0.277329	L	
H-H1-0.039100	0	12.880553	1.554504	-0.815916	L	
N-N--0.415700	0	8.178093	2.772225	1.904675	L	
C-CT-0.018800	-1	7.023299	3.601503	2.203927	L	H-HC 33
C-C-0.597300	0	6.167906	3.013134	3.322558	L	
O-O--0.567900	0	6.187750	1.804415	3.553551	L	
C-CT--0.515368	0	6.195091	3.721888	0.896369	H	
C-CM-0.287903	0	5.598482	2.444171	0.385904	H	
N-N2--0.582625	0	6.238119	1.643978	-0.547251	H	
C-CM--0.036893	0	4.467363	1.731495	0.712891	H	
C-CM-0.243454	0	5.505671	0.518072	-0.740561	H	
N-N2--0.523998	0	4.428549	0.535074	0.023099	H	
H-H-0.271900	0	8.039469	1.775656	1.991922	L	
H-H1-0.088100	0	7.360007	4.596131	2.511522	L	
H-HC-0.162301	0	6.846642	4.178150	0.136673	H	
H-HC-0.188340	0	5.401470	4.455814	1.082255	H	
H-H-0.339448	0	7.149353	1.845749	-0.958803	H	
H-H4-0.185957	0	3.680232	2.002835	1.401698	H	
H-H5-0.211295	0	5.785776	-0.281898	-1.410593	H	
N-N--0.415700	0	5.398330	3.877706	3.972217	L	
C-CT-0.014300	-1	4.557936	3.588736	5.125011	L	
C-C-0.597300	0	5.207375	2.638995	6.142921	L	
O-O--0.567900	0	4.684246	1.586385	6.512473	L	
C-CT--0.204100	0	3.106386	3.315005	4.706006	L	
C-C-0.713000	0	2.176450	4.199153	5.516034	L	
N-N--0.919100	0	1.579457	3.709606	6.567101	L	
O-O--0.593100	0	2.010783	5.377248	5.251339	L	
H-H-0.271900	0	5.533106	4.865176	3.747734	L	
H-H1-0.104800	0	4.529243	4.543619	5.654885	L	
H-HC-0.079700	0	2.867114	2.267300	4.855465	L	
H-HC-0.079700	0	2.960011	3.559500	3.654159	L	

```
H-H-0.419600      0     0.919930    4.292740    7.034890 L
H-H-0.419600      0     1.616053    2.698663    6.697426 L
N-N--0.415700     0     6.393890    3.069838    6.584492 L
C-CT--0.149000   -1     7.300576    2.323207    7.435118 L
H-H-0.271900      0     6.706367    3.943176    6.183321 L
H-H1-0.097600     0     8.057187    2.985228    7.855683 L
H-H1-0.097600     0     7.784840    1.538330    6.850966 L
H-H1-0.097600     0     6.742491    1.846065    8.243842 L
C-CT--0.366200   -1    -2.253919   -2.736796    9.540265 L
C-C-0.597200      0    -2.371277   -3.831125    8.515259 L
O-O--0.567900     0    -2.894735   -4.900873    8.815409 L
H-HC-0.112300     0    -1.201456   -2.508725    9.697442 L
H-HC-0.112300     0    -2.704999   -3.070894   10.472109 L
H-HC-0.112300     0    -2.769876   -1.850416    9.175798 L
N-N--0.347900     0    -1.856072   -3.566064    7.317959 L
C-CT--0.240000   -1    -1.749105   -4.534127    6.220099 L
C-C-0.734100      0    -0.336189   -4.573539    5.649535 L
O-O--0.589400     0     0.487069   -3.704178    5.921105 L
C-CT--0.009400    0    -2.834628   -4.242690    5.163294 L
C-CT-0.018700     0    -2.597338   -2.966136    4.345117 L H-HC 37
C-CT--0.464524    0    -3.700318   -2.778946    3.296459 H
C-CT--0.195159    0    -3.599729   -1.476625    2.512427 H
N-N3--0.748751    0    -2.407829   -1.426747    1.598557 H
H-H-0.274700      0    -1.474145   -2.633238    7.175219 L
H-H1-0.142600     0    -1.942714   -5.529064    6.621279 L
H-HC-0.036200     0    -3.801454   -4.168622    5.664163 L
H-HC-0.036200     0    -2.893818   -5.078310    4.473245 L
H-HC-0.010300     0    -1.631451   -3.020965    3.842520 L
H-HC-0.010300     0    -2.606991   -2.112350    5.014928 L
H-HC-0.173903     0    -4.665767   -2.777822    3.810990 H
H-HC-0.148535     0    -3.699728   -3.641722    2.615300 H
H-HP-0.236066     0    -3.546199   -0.592170    3.145347 H
H-HP-0.172509     0    -4.471999   -1.371195    1.855922 H
H-H-0.437293      0    -2.282066   -0.462655    1.183024 H
H-H-0.366736      0    -2.559556   -2.075526    0.817744 H
H-H-0.419451      0    -1.486039   -1.634302    2.045682 H
N-N--0.415700     0    -0.043208   -5.602682    4.867420 L
C-CT--0.025200   -1     1.271217   -5.804141    4.266847 L
C-C-0.597300      0     1.145152   -6.519174    2.920815 L
O-O--0.567900     0     1.731561   -7.574766    2.689644 L
H-H-0.271900      0    -0.789738   -6.250316    4.632920 L
H-H1-0.069800     0     1.890408   -6.407398    4.930788 L
H-H1-0.069800     0     1.764796   -4.846264    4.098878 L
N-N--0.415700     0     0.327818   -5.933241    2.049933 L
C-CT--0.025200   -1    -0.239077   -6.532848    0.845228 L
C-C-0.597300      0    -1.384246   -5.661515    0.399852 L
O-O--0.567900     0    -2.293992   -5.444716    1.191931 L
H-H-0.271900      0    -0.111124   -5.081625    2.374446 L
H-H1-0.069800     0    -0.614262   -7.530872    1.071510 L
H-H1-0.069800     0     0.524811   -6.590533    0.069321 L
N-N--0.415700     0    -1.318459   -5.168813   -0.836740 L
C-CT--0.001400   -1    -2.161661   -4.096710   -1.367035 L
C-C-0.597300      0    -3.624918   -4.172336   -0.915920 L
O-O--0.567900     0    -4.183011   -3.214333   -0.392665 L
```

C-CT--0.015200	0	-2.064817	-4.084626	-2.906626	L
C-CA--0.001100	0	-0.672757	-4.258519	-3.490048	L
C-CA--0.190600	0	0.338337	-3.320449	-3.208545	L
C-CA--0.190600	0	-0.394772	-5.357007	-4.328310	L
C-CA--0.234100	0	1.625510	-3.480954	-3.755614	L
C-CA--0.234100	0	0.889812	-5.520082	-4.880952	L
C-C-0.322600	0	1.903787	-4.579885	-4.593889	L
O-OH--0.557900	0	3.137518	-4.736383	-5.143429	L
H-H-0.271900	0	-0.476897	-5.348207	-1.365766	L
H-H1-0.087600	0	-1.758521	-3.153322	-0.999075	L
H-HC-0.029500	0	-2.463151	-3.142304	-3.275995	L
H-HC-0.029500	0	-2.704167	-4.872719	-3.304855	L
H-HA-0.169900	0	0.130665	-2.464866	-2.576187	L
H-HA-0.169900	0	-1.176499	-6.063432	-4.565933	L
H-HA-0.165600	0	2.396818	-2.757979	-3.533603	L
H-HA-0.165600	0	1.093867	-6.354167	-5.531829	L
H-HO-0.399200	0	3.146838	-5.467801	-5.758243	L
N-N--0.415700	0	-4.228807	-5.349983	-1.088631	L
C-CT--0.024900	-1	-5.564186	-5.635334	-0.584661	L
C-C-0.597300	0	-5.693030	-5.565793	0.935361	L
O-O--0.567900	0	-5.082015	-6.346719	1.674743	L
C-CT-0.211700	0	-6.034808	-7.023855	-1.020312	L
O-OH--0.654600	-1	-7.286517	-7.333550	-0.422605	L
H-H-0.271900	0	-3.660544	-6.086504	-1.465681	L
H-H1-0.084300	0	-6.247781	-4.906336	-1.020250	L
H-H1-0.035200	0	-5.299823	-7.766193	-0.706898	L
H-H1-0.035200	0	-6.124713	-7.052353	-2.106250	L
H-HO-0.427500	-1	-7.545053	-8.205766	-0.730330	L
N-N--0.415700	0	-6.656998	-4.754954	1.364715	L
C-CT-0.033700	-1	-7.161061	-4.658386	2.723364	L
C-C-0.597300	0	-7.535241	-5.979710	3.400002	L
O-O--0.567900	0	-7.513251	-6.070963	4.625906	L
C-CT--0.182500	0	-8.340955	-3.685933	2.718244	L
H-H-0.271900	0	-7.097082	-4.157426	0.671329	L
H-H1-0.082300	0	-6.373312	-4.217583	3.333606	L
H-HC-0.060300	0	-8.037104	-2.724065	2.300804	L
H-HC-0.060300	0	-8.689835	-3.537722	3.735525	L
H-HC-0.060300	0	-9.156256	-4.099894	2.130831	L
N-N--0.415700	0	-7.894279	-6.995914	2.615071	L
C-CT--0.025200	-1	-8.290760	-8.302537	3.124914	L
C-C-0.597300	0	-7.323011	-9.439400	2.827600	L
O-O--0.567900	0	-7.721231	-10.602162	2.863772	L
H-H-0.271900	0	-7.848029	-6.837039	1.614793	L
H-H1-0.069800	0	-9.251502	-8.564781	2.683154	L
H-H1-0.069800	0	-8.431278	-8.265339	4.205409	L
N-N--0.415700	0	-6.054094	-9.128039	2.569144	L
C-CT--0.024900	-1	-4.976476	-10.115162	2.602617	L
C-C-0.597300	0	-4.290159	-10.076652	3.962253	L
O-O--0.567900	0	-3.979161	-9.001319	4.483625	L
C-CT-0.211700	0	-3.970978	-9.816324	1.485152	L
O-OH--0.654600	-1	-3.098539	-10.917630	1.284883	L
H-H-0.271900	0	-5.806217	-8.142274	2.559723	L
H-H1-0.084300	0	-5.381980	-11.112216	2.430374	L
H-H1-0.035200	0	-3.397147	-8.920561	1.728443	L

H-H1-0.035200	0	-4.518705	-9.637590	0.559017	L		
H-HO-0.427500	-1	-2.557645	-10.729735	0.514313	L		
N-N--0.415700	0	-4.084319	-11.254983	4.549594	L		
C-CT--0.149000	-1	-3.542009	-11.403621	5.888767	L		
H-H-0.271900	0	-4.358451	-12.073201	4.029616	L		
H-H1-0.097600	0	-3.318920	-12.450623	6.093614	L		
H-H1-0.097600	0	-4.262565	-11.037281	6.623326	L		
H-H1-0.097600	0	-2.625482	-10.816720	5.986392	L		
C-CT--0.366200	-1	-12.133249	5.103409	-0.649345	L		
C-C-0.597200	0	-10.704997	5.290522	-0.209327	L		
O-O--0.567900	0	-10.392614	5.027254	0.946428	L		
H-HC-0.112300	0	-12.526923	6.053012	-1.004803	L		
H-HC-0.112300	0	-12.719679	4.756798	0.199905	L		
H-HC-0.112300	0	-12.164801	4.356854	-1.440800	L		
N-N--0.415700	0	-9.854516	5.734507	-1.131075	L		
C-CT--0.025200	-1	-8.420578	5.890083	-0.901309	L		
C-C-0.597300	0	-7.601641	5.827657	-2.166009	L		
O-O--0.567900	0	-8.095525	6.195449	-3.238876	L		
H-H-0.271900	0	-10.164347	5.897954	-2.076507	L		
H-H1-0.069800	0	-8.105856	5.072295	-0.262480	L		
H-H1-0.069800	0	-8.217842	6.832974	-0.395462	L		
N-N--0.415700	0	-6.358708	5.371956	-2.002996	L	H-H1	48
C-CT--0.530209	-1	-5.336599	5.312371	-3.045967	H		
C-C-0.568093	0	-4.654288	3.956753	-2.954036	H		
O-O--0.528105	0	-4.691460	3.298090	-1.911700	H		
C-CT-0.298500	0	-4.361903	6.508505	-2.938984	L	H-H1	48
C-CT--0.319200	0	-5.075631	7.826161	-3.250705	L		
C-CT--0.319200	0	-3.700123	6.607088	-1.562263	L		
H-H-0.271900	0	-6.079290	5.027321	-1.086622	L		
H-H1-0.184096	0	-5.845915	5.409680	-4.012329	H		
H-HC--0.029700	0	-3.577453	6.384557	-3.684065	L		
H-HC-0.079100	0	-5.813724	8.042266	-2.480071	L		
H-HC-0.079100	0	-5.573196	7.749971	-4.216244	L		
H-HC-0.079100	0	-4.344628	8.632853	-3.284449	L		
H-HC-0.079100	0	-4.447243	6.737730	-0.781392	L		
H-HC-0.079100	0	-3.013006	7.449726	-1.542181	L		
H-HC-0.079100	0	-3.141662	5.692137	-1.356859	L		
N-N--0.610103	0	-3.986307	3.550219	-4.062143	H		
C-CT--0.314060	-1	-4.225753	4.019736	-5.423019	H		
C-C-0.597300	0	-5.339992	3.206554	-6.069517	L	H-H1	50
O-O--0.567900	0	-5.465984	1.999971	-5.863610	L		
C-CT--0.110200	0	-2.921115	3.887479	-6.245173	L	H-H1	50
C-CT-0.353100	0	-1.835060	4.942701	-5.927305	L		
C-CT--0.412100	0	-1.047569	4.681566	-4.641092	L		
C-CT--0.412100	0	-0.816895	4.986235	-7.071660	L		
H-H-0.398207	0	-3.541806	2.637828	-3.972338	H		
H-H1-0.165130	0	-4.512462	5.069937	-5.422537	H		
H-HC-0.045700	0	-3.188510	3.994063	-7.296515	L		
H-HC-0.045700	0	-2.504540	2.888430	-6.114772	L		
H-HC--0.036100	0	-2.302526	5.925925	-5.863921	L		
H-HC-0.100000	0	-0.618072	3.681828	-4.660769	L		
H-HC-0.100000	0	-1.711505	4.749443	-3.779631	L		
H-HC-0.100000	0	-0.260236	5.423468	-4.521670	L		
H-HC-0.100000	0	-0.320910	4.020698	-7.158754	L		

```
H-HC-0.100000    0   -0.072457    5.755709   -6.868201 L
H-HC-0.100000    0   -1.321415    5.224143   -8.006401 L
N-N--0.415700    0   -6.129116    3.857989   -6.916515 L
C-CT--0.038900  -1   -7.317818    3.255981   -7.532380 L
C-C-0.597300     0   -7.075199    1.921025   -8.226567 L
O-O--0.567900    0   -7.959384    1.068084   -8.275468 L
C-CT-0.365400   -1   -7.953857    4.181989   -8.593478 L
O-OH--0.676100  -1   -7.890514    5.538783   -8.144528 L
C-CT--0.243800   0   -9.433129    3.895768   -8.852225 L
H-H-0.271900     0   -6.011585    4.857127   -6.996537 L
H-H1-0.100700    0   -8.056561    3.114549   -6.750391 L
H-H1-0.004300    0   -7.399139    4.105916   -9.529704 L
H-HC-0.064200    0   -9.551338    2.893140   -9.262676 L
H-HC-0.064200    0   -9.826205    4.612089   -9.573420 L
H-HC-0.064200    0   -9.995849    3.966015   -7.920552 L
H-HO-0.410200   -1   -8.788295    5.875082   -8.108880 L
N-N--0.415700    0   -5.899220    1.784737   -8.835839 L
C-CT--0.025200  -1   -5.513833    0.626016   -9.627730 L
C-C-0.597300     0   -4.606351   -0.367876   -8.934710 L
O-O--0.567900    0   -4.113965   -1.273270   -9.608952 L
H-H-0.271900     0   -5.210485    2.495730   -8.640995 L
H-H1-0.069800    0   -4.993668    0.971254  -10.519985 L
H-H1-0.069800    0   -6.401760    0.084132   -9.953194 L
N-N--0.415700    0   -4.382037   -0.185141   -7.628078 L H-H1 54
C-CT--0.539097  -1   -3.472826   -0.989661   -6.824700 H
C-C-0.572106     0   -4.227082   -1.439712   -5.570220 H
O-O--0.533001    0   -4.952311   -2.429967   -5.654263 H
C-CT-0.130300    0   -2.095793   -0.301082   -6.644589 L H-H1 54
C-CT--0.043000   0   -1.465696    0.131914   -7.989578 L
C-CT--0.320400   0   -1.183367   -1.306347   -5.919886 L
C-CT--0.066000   0   -0.110786    0.839961   -7.858243 L
H-H-0.271900     0   -4.845181    0.584426   -7.152262 L
H-H1-0.180646    0   -3.293286   -1.916415   -7.381207 H
H-HC-0.018700    0   -2.220644    0.586829   -6.031654 L
H-HC-0.023600    0   -2.136167    0.834961   -8.482327 L
H-HC-0.023600    0   -1.350915   -0.739952   -8.634754 L
H-HC-0.088200    0   -1.078202   -2.214877   -6.511042 L
H-HC-0.088200    0   -1.602396   -1.560357   -4.947647 L
H-HC-0.088200    0   -0.201001   -0.880221   -5.737206 L
H-HC-0.018600    0   -0.187919    1.653385   -7.137777 L
H-HC-0.018600    0    0.177428    1.248288   -8.826887 L
H-HC-0.018600    0    0.657584    0.138301   -7.536876 L
N-N--0.611145    0   -4.099277   -0.681769   -4.454080 H
C-CT--0.109296  -1   -4.637727   -1.092524   -3.162842 H
C-C-0.597300     0   -6.166914   -1.107965   -3.145405 L H-H1 56
O-O--0.567900    0   -6.797910   -2.043413   -2.638219 L
C-CT--0.388038   0   -4.038126   -0.267693   -2.038529 H
H-H-0.369964     0   -3.428522    0.083097   -4.413494 H
H-H1-0.178541    0   -4.377438   -2.141632   -2.987573 H
H-H1-0.157964    0   -4.519722   -0.581359   -1.104892 H
H-H1-0.179833    0   -4.202428    0.807176   -2.150760 H
H-HS-0.319500    0   -1.763347    1.639176   -0.247085 H
N-N--0.415700    0   -6.758265   -0.113513   -3.795299 L
C-CT--0.087500  -1   -8.193013   -0.047355   -4.058419 L
```

C-C-0.597300	0	-8.675810	-1.198720	-4.927032	L
O-O--0.567900	0	-9.624657	-1.891347	-4.558615	L
C-CT-0.298500	0	-8.577584	1.315570	-4.653245	L
C-CT--0.319200	0	-10.091054	1.450250	-4.870966	L
C-CT--0.319200	0	-8.138834	2.491755	-3.766462	L
H-H-0.271900	0	-6.114606	0.567926	-4.190035	L
H-H1-0.096900	0	-8.707204	-0.141678	-3.104673	L
H-HC--0.029700	0	-8.085427	1.397435	-5.613842	L
H-HC-0.079100	0	-10.614940	1.305264	-3.925909	L
H-HC-0.079100	0	-10.433971	0.708905	-5.592424	L
H-HC-0.079100	0	-10.319020	2.440729	-5.263604	L
H-HC-0.079100	0	-8.607143	2.429087	-2.786120	L
H-HC-0.079100	0	-8.404343	3.439811	-4.233254	L
H-HC-0.079100	0	-7.056567	2.485449	-3.630942	L
N-N--0.415700	0	-8.037277	-1.411904	-6.076038	L
C-CT--0.025200	-1	-8.405966	-2.462073	-7.022745	L
C-C-0.597300	0	-8.317350	-3.867112	-6.440129	L
O-O--0.567900	0	-9.212374	-4.684250	-6.646495	L
H-H-0.271900	0	-7.232232	-0.829845	-6.270877	L
H-H1-0.069800	0	-7.743121	-2.408398	-7.886068	L
H-H1-0.069800	0	-9.428188	-2.294599	-7.361426	L
N-N--0.415700	0	-7.238816	-4.143184	-5.709509	L
C-CT--0.025200	-1	-7.015821	-5.407805	-5.020594	L
C-C-0.597300	0	-7.950299	-5.668421	-3.850497	L
O-O--0.567900	0	-8.292146	-6.819652	-3.584921	L
H-H-0.271900	0	-6.527061	-3.417871	-5.634464	L
H-H1-0.069800	0	-5.993319	-5.424225	-4.644889	L
H-H1-0.069800	0	-7.131642	-6.224668	-5.733007	L
N-N--0.415700	0	-8.332891	-4.612129	-3.136505	L
C-CT--0.038900	-1	-9.385359	-4.668905	-2.119670	L
C-C-0.597300	0	-10.755380	-4.960257	-2.723131	L
O-O--0.567900	0	-11.498633	-5.797891	-2.211201	L
C-CT-0.365400	0	-9.421888	-3.367478	-1.304180	L
O-OH--0.676100	0	-8.187700	-3.205767	-0.643843	L
C-CT--0.243800	0	-10.511998	-3.375669	-0.232787	L
H-H-0.271900	0	-7.935985	-3.709260	-3.374346	L
H-H1-0.100700	0	-9.159477	-5.484188	-1.432510	L
H-H1-0.004300	0	-9.583352	-2.515461	-1.964741	L
H-HC-0.064200	0	-11.490472	-3.266196	-0.699061	L
H-HC-0.064200	0	-10.356902	-2.545653	0.456075	L
H-HC-0.064200	0	-10.482691	-4.315029	0.317624	L
H-HO-0.410200	0	-7.597106	-2.738513	-1.270222	L
N-N--0.415700	0	-11.105233	-4.249994	-3.796777	L
C-CT--0.149000	-1	-12.337441	-4.441403	-4.533764	L
H-H-0.271900	0	-10.439026	-3.564365	-4.141966	L
H-H1-0.097600	0	-12.413843	-3.686811	-5.317567	L
H-H1-0.097600	0	-13.189669	-4.358467	-3.857945	L
H-H1-0.097600	0	-12.334220	-5.433045	-4.989297	L
N-N2--0.511415	0	1.989543	-0.067750	-1.687943	H
C-CA-0.057801	0	0.689001	0.232789	-1.627240	H
C-CA-0.063496	0	-0.021897	0.740023	-2.716390	H
C-CA--0.109122	0	0.671522	0.999465	-3.895331	H
C-CA--0.164490	0	2.031666	0.712344	-3.953544	H
C-CA-0.098392	0	2.646264	0.157348	-2.837619	H

C-C-0.541263	0	-1.523322	0.925119	-2.694925	H
O-O--0.574884	0	-2.085243	1.395306	-3.704080	H
H-H4-0.220889	0	0.202194	0.064355	-0.673692	H
H-HA-0.160434	0	0.125797	1.399912	-4.741942	H
H-HA-0.142191	0	2.611014	0.908107	-4.849361	H
H-H4-0.174502	0	3.693216	-0.124031	-2.851844	H
H-H-0.353801	0	-1.166287	2.834457	-1.462066	H
Zn-ZN-0.974537	0	2.871618	-1.109680	-0.001980	H
O-OW--0.834000	0	-2.139358	-7.240315	3.491682	L
H-HW-0.417000	0	-2.569235	-6.655750	2.847279	L
H-HW-0.417000	0	-2.869676	-7.778369	3.838344	L
O-OW--0.783903	0	1.319983	-2.575376	-0.125144	H
H-HW-0.401253	0	0.937552	-2.471439	0.781483	H
H-HW-0.424111	0	1.931991	-3.354320	-0.174701	H
O-OW--0.834000	0	-3.708768	-8.445201	7.231759	L
H-HW-0.417000	0	-3.758467	-7.500663	7.399731	L
H-HW-0.417000	0	-3.828457	-8.519373	6.272700	L
O-OW--0.834000	0	7.183747	-0.013757	1.818749	L
H-HW-0.417000	0	6.879282	0.390527	2.651429	L
H-HW-0.417000	0	6.395808	0.086538	1.261279	L
O-OW--0.834000	0	7.044992	-4.961730	4.772212	L
H-HW-0.417000	0	6.094311	-5.013468	5.001083	L
H-HW-0.417000	0	7.241453	-5.842320	4.450383	L
O-OW--0.834000	0	8.634622	-0.931453	-0.237265	L
H-HW-0.417000	0	8.170506	-0.560477	0.540765	L
H-HW-0.417000	0	8.844442	-0.140059	-0.754097	L
O-OW--0.834000	0	12.663366	-1.084254	-1.989766	L
H-HW-0.417000	0	11.814883	-0.830874	-2.392330	L
H-HW-0.417000	0	12.663216	-2.047988	-2.095847	L
O-OW--0.834000	0	8.282143	-3.305836	0.993525	L
H-HW-0.417000	0	8.493061	-2.534782	0.426750	L
H-HW-0.417000	0	7.550154	-2.972062	1.522635	L
O-OW--0.834000	0	4.355104	-4.906425	5.170371	L
H-HW-0.417000	0	4.086391	-4.523048	6.028220	L
H-HW-0.417000	0	4.040607	-4.245143	4.543355	L
S-SH--0.326142	0	-2.246507	-0.607442	-1.869454	H
O-OW--0.834000	0	-1.733625	2.049715	-1.357269	H

PC$_a$

C-CT--0.3662	-1	-10.306290	3.912184	4.022624	L
C-C-0.5972	0	-8.979366	3.253767	3.780943	L
O-O--0.5679	0	-8.418707	2.662423	4.695814	L
H-HC-0.1123	0	-11.047659	3.464822	3.363534	L
H-HC-0.1123	0	-10.217308	4.976017	3.809510	L
H-HC-0.1123	0	-10.594269	3.763664	5.060919	L
N-N--0.415700	0	-8.504957	3.360360	2.547347	L
C-CT--0.059700	-1	-7.339685	2.642009	2.023926	L
C-C-0.597300	0	-6.186208	3.617088	1.840365	L
O-O--0.567900	0	-6.323806	4.592316	1.107538	L
C-CT-0.130300	0	-7.716642	1.928899	0.701654	L
C-CT--0.043000	0	-8.845433	0.893797	0.936992	L
C-CT--0.320400	0	-6.491538	1.239196	0.080228	L
C-CT--0.066000	0	-9.428752	0.299040	-0.349897	L
H-H-0.271900	0	-9.033422	3.937513	1.901741	L

```
H-H1-0.086900   0    -7.028709    1.879676    2.737940 L
H-HC-0.018700   0    -8.077195    2.679581   -0.004086 L
H-HC-0.023600   0    -9.673436    1.368413    1.462209 L
H-HC-0.023600   0    -8.472404    0.083380    1.564685 L
H-HC-0.088200   0    -6.083256    0.493824    0.763789 L
H-HC-0.088200   0    -5.715428    1.971125   -0.149842 L
H-HC-0.088200   0    -6.760173    0.765350   -0.861430 L
H-HC-0.018600   0    -9.708003    1.097221   -1.037183 L
H-HC-0.018600   0   -10.318980   -0.279467   -0.106425 L
H-HC-0.018600   0    -8.703408   -0.361892   -0.821734 L
N-N--0.516300   0    -5.083745    3.357184    2.538492 L
C-CT-0.038100   -1   -3.773508    3.977631    2.382609 L H-HC      30
C-C-0.536600    0    -3.706015    5.495448    2.113025 L
O-O--0.581900   0    -2.738934    6.024171    1.566997 L
C-CT--0.547145  0    -3.020636    3.137721    1.319361 H
C-C-0.532653    0    -2.520053    1.828011    1.877301 H
O-O2--0.588507  0    -2.589272    1.560474    3.062004 H
O-O2--0.625559  0    -1.873102    0.971892    1.074761 H
H-H-0.293600    0    -5.064112    2.496129    3.071411 L
H-H1-0.088000   0    -3.241908    3.860071    3.327239 L
H-HC-0.128435   0    -2.126139    3.687271    0.987886 H
H-HC-0.183556   0    -3.628609    3.027439    0.416469 H
N-N--0.415700   0    -4.670022    6.250623    2.623556 L
C-CT--0.025200  -1   -4.650727    7.695968    2.502133 L
C-C-0.597300    0    -3.749515    8.334782    3.542135 L
O-O--0.567900   0    -4.224017    9.004066    4.461516 L
H-H-0.271900    0    -5.479119    5.751460    2.956880 L
H-H1-0.069800   0    -5.661433    8.077794    2.637663 L
H-H1-0.069800   0    -4.307764    7.984124    1.507094 L
N-N--0.415700   0    -2.446650    8.115921    3.395647 L
C-CT--0.038900  -1   -1.426765    8.561336    4.338840 L
C-C-0.597300    0    -0.520791    9.644478    3.741365 L
O-O--0.567900   0    -0.624088   10.014572    2.570773 L
C-CT-0.365400   0    -0.581016    7.411329    4.932150 L
O-OH--0.676100  0     0.615735    7.246885    4.213413 L
C-CT--0.243800  0    -1.290316    6.064521    5.041654 L
H-H-0.271900    0    -2.183545    7.518201    2.613839 L
H-H1-0.100700   0    -1.935177    9.023751    5.182850 L
H-H1-0.004300   0    -0.299433    7.710461    5.942323 L
H-HC-0.064200   0    -2.229257    6.187260    5.579263 L
H-HC-0.064200   0    -0.654844    5.367963    5.583528 L
H-HC-0.064200   0    -1.488188    5.655400    4.051077 L
H-HO-0.410200   0     1.108953    6.489704    4.597470 L
N-N--0.415700   0     0.397772   10.156924    4.564457 L
C-CT--0.025200  -1    1.440059   11.089011    4.148011 L
C-C-0.597300    0     2.339332   10.597240    3.023014 L
O-O--0.567900   0     2.673172   11.372594    2.131929 L
H-H-0.271900    0     0.464616    9.748297    5.481259 L
H-H1-0.069800   0     2.076152   11.321893    5.000793 L
H-H1-0.069800   0     0.969715   12.013618    3.812791 L
N-N--0.516300   0     2.735870    9.328216    3.080752 L
C-CT-0.038100   -1    3.645268    8.730659    2.103866 L
C-C-0.536600    0     3.016368    8.443798    0.753964 L
O-O--0.581900   0     3.750786    8.160686   -0.185959 L
```

C-CT--0.030300	0	4.231322	7.417423	2.653392	L
C-C-0.799400	-1	5.577159	7.620206	3.332003	L
O-O2--0.801400	-1	6.157024	8.724458	3.248168	L
O-O2--0.801400	-1	6.037865	6.667997	3.994022	L
H-H-0.293600	0	2.362446	8.721921	3.797458	L
H-H1-0.088000	0	4.447379	9.438178	1.891280	L
H-HC--0.012200	0	4.389752	6.704937	1.842083	L
H-HC--0.012200	0	3.528500	6.958379	3.351787	L
N-N--0.415700	0	1.693749	8.519862	0.643010	L
C-CT--0.002400	-1	0.973328	8.315219	-0.606581	L
C-C-0.597300	0	0.383646	9.610304	-1.166163	L
O-O--0.567900	0	0.366493	9.819254	-2.379380	L
C-CT--0.034300	0	-0.104061	7.238841	-0.420738	L
C-CA-0.011800	0	0.413199	5.915706	0.118778	L
C-CA--0.125600	0	1.038881	4.982840	-0.728201	L
C-CA--0.125600	0	0.235196	5.602195	1.476188	L
C-CA--0.170400	0	1.464711	3.744053	-0.213460	L
C-CA--0.170400	0	0.640351	4.360086	1.989098	L
C-CA--0.107200	0	1.249328	3.427213	1.137451	L
H-H-0.271900	0	1.173890	8.704616	1.488112	L
H-H1-0.097800	0	1.658704	7.938279	-1.364965	L
H-HC-0.029500	0	-0.581314	7.052174	-1.383928	L
H-HC-0.029500	0	-0.873729	7.621166	0.251433	L
H-HA-0.133000	0	1.151187	5.197661	-1.779523	L
H-HA-0.133000	0	-0.274243	6.304846	2.109547	L
H-HA-0.143000	0	1.907376	3.005043	-0.861619	L
H-HA-0.143000	0	0.438720	4.106042	3.018405	L
H-HA-0.129700	0	1.512171	2.450826	1.509569	L
N-N--0.4157	0	-0.071113	10.506610	-0.287891	L
C-CT--0.1490	-1	-0.476354	11.848685	-0.653537	L
H-H-0.2719	0	-0.064974	10.246263	0.693082	L
H-H1-0.0976	0	-0.864257	12.364300	0.225419	L
H-H1-0.0976	0	-1.250675	11.800448	-1.420971	L
H-H1-0.0976	0	0.383959	12.393414	-1.045213	L
C-CT--0.3662	-1	7.983740	8.433337	-0.178728	L
C-C-0.5972	0	7.522531	7.637275	-1.377057	L
O-O--0.5679	0	8.343663	7.292844	-2.223661	L
H-HC-0.1123	0	7.516479	9.415537	-0.194734	L
H-HC-0.1123	0	9.066164	8.533095	-0.208994	L
H-HC-0.1123	0	7.696052	7.910094	0.734070	L
N-N--0.415700	0	6.213528	7.386573	-1.470647	L
C-CT--0.051800	-1	5.560604	6.852489	-2.673573	L
C-C-0.597300	0	4.920194	7.976640	-3.488406	L
O-O--0.567900	0	5.307088	8.245331	-4.626210	L
C-CT--0.110200	0	4.614093	5.679929	-2.328400	L
C-CT-0.353100	0	4.557642	4.616704	-3.446500	L
C-CT--0.412100	0	3.762197	3.393595	-3.003596	L
C-CT--0.412100	0	3.912802	5.112555	-4.743302	L
H-H-0.271900	0	5.609327	7.706959	-0.720948	L
H-H1-0.092200	0	6.346314	6.443673	-3.310076	L
H-HC-0.045700	0	3.611404	6.046930	-2.106983	L
H-HC-0.045700	0	4.990043	5.188900	-1.430606	L
H-HC--0.036100	0	5.573587	4.286016	-3.667304	L
H-HC-0.100000	0	2.704899	3.643013	-2.919045	L

H-HC-0.100000	0	4.123157	3.038162	-2.040054	L		
H-HC-0.100000	0	3.881093	2.594906	-3.732985	L		
H-HC-0.100000	0	2.936747	5.549601	-4.528839	L		
H-HC-0.100000	0	3.788023	4.282383	-5.437518	L		
H-HC-0.100000	0	4.548790	5.857466	-5.216729	L		
N-N--0.4157	0	3.970424	8.658260	-2.861938	L		
C-CT--0.1490	-1	3.351811	9.881040	-3.304115	L		
H-H-0.2719	0	3.770831	8.343273	-1.919723	L		
H-H1-0.0976	0	4.064709	10.476130	-3.876227	L		
H-H1-0.0976	0	3.013373	10.454027	-2.439581	L		
H-H1-0.0976	0	2.491361	9.647564	-3.933341	L		
C-CT--0.3662	-1	-8.716392	2.374803	8.131688	L		
C-C-0.5972	0	-7.869055	1.146969	7.954278	L		
O-O--0.5679	0	-7.577796	0.450254	8.919886	L		
H-HC-0.1123	0	-9.621064	2.268768	7.536055	L		
H-HC-0.1123	0	-8.153850	3.244195	7.794914	L		
H-HC-0.1123	0	-8.972059	2.482386	9.183602	L		
N-N--0.415700	0	-7.465811	0.901345	6.716344	L		
C-CT--0.038900	-1	-6.655043	-0.244659	6.302083	L		
C-C-0.597300	0	-5.398643	0.258150	5.614858	L		
O-O--0.567900	0	-5.459484	0.720352	4.476500	L		
C-CT-0.365400	0	-7.453642	-1.220141	5.424189	L		
O-OH--0.676100	0	-8.015851	-0.565738	4.312715	L		
C-CT--0.243800	0	-8.595761	-1.851421	6.225879	L		
H-H-0.271900	0	-7.776809	1.533786	5.982817	L		
H-H1-0.100700	0	-6.344792	-0.802236	7.185417	L		
H-H1-0.004300	0	-6.787943	-2.010098	5.074767	L		
H-HC-0.064200	0	-8.191918	-2.375468	7.091752	L		
H-HC-0.064200	0	-9.141535	-2.558075	5.607366	L		
H-HC-0.064200	0	-9.291379	-1.082025	6.559256	L		
H-HO-0.410200	0	-7.337237	0.041303	3.981759	L		
N-N--0.415700	0	-4.297730	0.231278	6.370581	L		
C-CT--0.059700	-1	-3.102476	1.051852	6.161887	L		
C-C-0.597300	0	-1.858248	0.169923	6.088979	L		
O-O--0.567900	0	-1.609280	-0.637496	6.990376	L		
C-CT-0.130300	0	-2.980872	2.112890	7.280869	L		
C-CT--0.043000	0	-4.239424	3.000254	7.452505	L		
C-CT--0.320400	0	-1.745205	2.996610	7.051841	L		
C-CT--0.066000	0	-4.666488	3.808986	6.218151	L		
H-H-0.271900	0	-4.350454	-0.235536	7.260741	L		
H-H1-0.086900	0	-3.210217	1.590717	5.229059	L		
H-HC-0.018700	0	-2.833736	1.588570	8.226981	L		
H-HC-0.023600	0	-4.059412	3.700361	8.269033	L		
H-HC-0.023600	0	-5.077537	2.374626	7.758257	L		
H-HC-0.088200	0	-1.746339	3.371088	6.027745	L		
H-HC-0.088200	0	-0.840623	2.406765	7.201289	L		
H-HC-0.088200	0	-1.734726	3.830403	7.752386	L		
H-HC-0.018600	0	-3.868934	4.481792	5.908065	L		
H-HC-0.018600	0	-5.548675	4.400521	6.463088	L		
H-HC-0.018600	0	-4.915953	3.144375	5.394081	L		
N-N--0.516300	0	-1.099517	0.348546	5.013915	L		
C-CT-0.038100	-1	0.192911	-0.235042	4.725743	L	H-HC	178
C-C-0.536600	0	1.123865	-0.083646	5.934424	L		
O-O--0.581900	0	1.328762	1.006160	6.463846	L		

C-CT--0.520019	0	0.713193	0.423815	3.434708	H		
C-C-0.636404	0	0.940021	-0.508747	2.253301	H		
O-O2--0.608036	0	0.331217	-1.615688	2.166330	H		
O-O2--0.608131	0	1.768959	-0.055941	1.398892	H		
H-H-0.293600	0	-1.484780	0.950704	4.292358	L		
H-H1-0.088000	0	0.041522	-1.301735	4.553924	L		
H-HC-0.132300	0	1.666856	0.914504	3.645350	H		
H-HC-0.188993	0	0.028333	1.211004	3.107079	H		
N-N--0.415700	0	1.676441	-1.193320	6.391558	L		
C-CT-0.033700	-1	2.579692	-1.232703	7.519210	L		
C-C-0.597300	0	3.802821	-2.052646	7.124290	L		
O-O--0.567900	0	3.858125	-3.276528	7.290364	L		
C-CT--0.182500	0	1.826823	-1.786431	8.737234	L		
H-H-0.271900	0	1.378100	-2.070992	5.981041	L		
H-H1-0.082300	0	2.923472	-0.226050	7.764879	L		
H-HC-0.060300	0	0.985975	-1.132117	8.970836	L		
H-HC-0.060300	0	2.499241	-1.830808	9.593595	L		
H-HC-0.060300	0	1.451861	-2.787671	8.519131	L		
N-N--0.4157	0	4.769777	-1.359207	6.536071	L		
C-CT-0.0188	-1	5.974396	-1.940996	5.988444	L	H-HC	200
C-C-0.5973	0	7.172465	-1.792326	6.935435	L		
O-O--0.5679	0	7.218827	-0.946114	7.830850	L		
C-CT--0.517456	0	6.221795	-1.299796	4.620195	H		
C-CM-0.287351	0	5.331873	-1.758176	3.522879	H		
N-N2--0.586994	0	5.451505	-3.013558	2.943499	H		
C-CM--0.020338	0	4.357005	-1.134207	2.793750	H		
C-CM-0.242458	0	4.578614	-3.088363	1.903899	H		
N-N2--0.505070	0	3.904961	-1.959301	1.783548	H		
H-H-0.2719	0	4.658847	-0.351234	6.466366	L		
H-H1-0.0881	0	5.827692	-3.006940	5.846348	L		
H-HC-0.166355	0	7.271007	-1.480204	4.343456	H		
H-HC-0.174481	0	6.128012	-0.220346	4.763837	H		
H-H-0.335795	0	6.096645	-3.737137	3.252498	H		
H-H4-0.152777	0	3.973422	-0.132933	2.920695	H		
H-H5-0.209475	0	4.465101	-3.951718	1.263037	H		
N-N--0.4157	0	8.178807	-2.627868	6.685462	L		
C-CT--0.1490	-1	9.446775	-2.557514	7.378731	L		
H-H-0.2719	0	8.028216	-3.302995	5.942724	L		
H-H1-0.0976	0	10.120979	-3.325747	6.999038	L		
H-H1-0.0976	0	9.287145	-2.707388	8.447898	L		
H-H1-0.0976	0	9.893125	-1.573525	7.223820	L		
C-CT--0.3662	-1	8.368030	-9.386184	0.929218	L		
C-C-0.5972	0	8.745843	-9.161433	-0.522567	L		
O-O--0.5679	0	9.779784	-8.558777	-0.784036	L		
H-HC-0.1123	0	8.289607	-10.451881	1.130680	L		
H-HC-0.1123	0	7.427914	-8.881048	1.143376	L		
H-HC-0.1123	0	9.146003	-8.954023	1.558495	L		
N-N--0.254800	0	7.926160	-9.620562	-1.484869	L		
C-CT--0.026600	-1	8.166030	-9.383485	-2.910634	L		
C-C-0.589600	0	8.013390	-7.911208	-3.292793	L		
O-O--0.574800	0	8.721285	-7.416065	-4.166676	L		
C-CT--0.007000	0	7.158548	-10.278354	-3.640190	L		
C-CT-0.018900	0	6.000071	-10.402714	-2.649246	L		
C-CT-0.019200	0	6.694382	-10.372911	-1.286804	L		

H-H1-0.064100	0	9.178378	-9.692972	-3.173505	L		
H-HC-0.025300	0	7.602491	-11.261776	-3.801951	L		
H-HC-0.025300	0	6.836088	-9.849368	-4.590234	L		
H-HC-0.021300	0	5.439285	-11.326268	-2.795982	L		
H-HC-0.021300	0	5.341094	-9.538165	-2.743583	L		
H-H1-0.039100	0	6.047716	-9.897687	-0.547797	L		
H-H1-0.039100	0	6.938839	-11.389305	-0.975264	L		
N-N--0.516300	0	7.091794	-7.214267	-2.626663	L		
C-CT-0.039700	-1	6.839994	-5.787898	-2.803516	L		
C-C-0.536600	0	8.099473	-4.922873	-2.677656	L		
O-O--0.581900	0	8.273963	-3.942148	-3.404418	L		
C-CT-0.056000	0	5.739386	-5.339474	-1.832048	L	H-HC	244
C-CT--0.515956	0	5.223724	-3.939494	-2.189274	H		
C-C-0.587828	0	4.120134	-3.405422	-1.280138	H		
O-O2--0.600319	0	4.046132	-2.123381	-1.252313	H		
O-O2--0.589127	0	3.371715	-4.198518	-0.664354	H		
H-H-0.293600	0	6.560834	-7.688065	-1.915099	L		
H-H1-0.110500	0	6.465108	-5.637208	-3.816251	L		
H-HC--0.017300	0	6.127678	-5.339213	-0.812387	L		
H-HC--0.017300	0	4.908099	-6.043830	-1.892312	L		
H-HC-0.167759	0	4.832749	-3.966363	-3.213642	H		
H-HC-0.135230	0	6.053192	-3.223611	-2.179272	H		
N-N--0.415700	0	9.009749	-5.325669	-1.793546	L		
C-CT--0.024900	-1	10.248993	-4.624091	-1.479504	L		
C-C-0.597300	0	11.270584	-4.515644	-2.604767	L		
O-O--0.567900	0	12.252969	-3.785648	-2.471703	L		
C-CT-0.211700	0	10.890042	-5.258975	-0.239266	L		
O-OH--0.654600	0	9.933616	-5.450746	0.792651	L		
H-H-0.271900	0	8.798602	-6.130956	-1.220727	L		
H-H1-0.084300	0	9.982589	-3.599706	-1.214483	L		
H-H1-0.035200	0	11.688087	-4.609811	0.123989	L		
H-H1-0.035200	0	11.317725	-6.226376	-0.506863	L		
H-HO-0.427500	0	9.385710	-4.638216	0.876131	L		
N-N--0.415700	0	11.029199	-5.199465	-3.721410	L		
C-CT-0.033700	-1	11.778681	-5.037391	-4.960448	L		
C-C-0.597300	0	10.945173	-4.483908	-6.120964	L		
O-O--0.567900	0	11.377048	-4.499039	-7.272891	L		
C-CT--0.182500	0	12.407717	-6.398356	-5.292581	L		
H-H-0.271900	0	10.213164	-5.800891	-3.728716	L		
H-H1-0.082300	0	12.593183	-4.328030	-4.807612	L		
H-HC-0.060300	0	13.024513	-6.733891	-4.458142	L		
H-HC-0.060300	0	13.033898	-6.307667	-6.180796	L		
H-HC-0.060300	0	11.622705	-7.132439	-5.481208	L		
N-N--0.415700	0	9.765994	-3.965388	-5.795871	L		
C-CT--0.025200	-1	8.861528	-3.284263	-6.699239	L		
C-C-0.597300	0	8.813922	-1.780085	-6.480609	L		
O-O--0.567900	0	8.832811	-1.008569	-7.436861	L		
H-H-0.271900	0	9.488306	-4.019898	-4.822080	L		
H-H1-0.069800	0	7.855011	-3.675592	-6.554423	L		
H-H1-0.069800	0	9.150491	-3.468595	-7.734403	L		
N-N--0.415700	0	8.751897	-1.372955	-5.215923	L		
C-CT--0.002400	-1	8.579584	0.012685	-4.796493	L		
C-C-0.597300	0	9.696198	0.447885	-3.840572	L		
O-O--0.567900	0	10.322461	-0.402993	-3.199824	L		

C-CT--0.034300	0	7.192734	0.135519	-4.138855	L		
C-CA-0.011800	0	6.046826	-0.123186	-5.099814	L		
C-CA--0.125600	0	5.626391	0.888077	-5.984635	L		
C-CA--0.125600	0	5.431583	-1.388473	-5.145245	L		
C-CA--0.170400	0	4.608976	0.629964	-6.921251	L		
C-CA--0.170400	0	4.410217	-1.644593	-6.077208	L		
C-CA--0.107200	0	4.005151	-0.639158	-6.972538	L		
H-H-0.271900	0	8.752876	-2.079417	-4.489657	L		
H-H1-0.097800	0	8.607926	0.662428	-5.671379	L		
H-HC-0.029500	0	7.067718	1.131963	-3.716049	L		
H-HC-0.029500	0	7.134899	-0.571801	-3.309854	L		
H-HA-0.133000	0	6.090674	1.863113	-5.953612	L		
H-HA-0.133000	0	5.752410	-2.168667	-4.470871	L		
H-HA-0.143000	0	4.288375	1.408813	-7.598505	L		
H-HA-0.143000	0	3.934292	-2.614005	-6.104153	L		
H-HA-0.129700	0	3.216047	-0.836057	-7.684856	L		
N-N--0.254800	0	9.953267	1.761400	-3.698615	L		
C-CT--0.026600	-1	10.818058	2.256730	-2.634231	L		
C-C-0.589600	0	10.183408	2.012381	-1.256228	L		
O-O--0.574800	0	8.989096	1.706501	-1.176272	L		
C-CT--0.007000	0	10.979016	3.755523	-2.920739	L		
C-CT-0.018900	0	9.660884	4.124664	-3.599160	L		
C-CT-0.019200	0	9.347244	2.875334	-4.422276	L		
H-H1-0.064100	0	11.786874	1.761387	-2.689194	L		
H-HC-0.025300	0	11.802440	3.905965	-3.620383	L		
H-HC-0.025300	0	11.139826	4.341763	-2.014659	L		
H-HC-0.021300	0	9.758587	5.011702	-4.225803	L		
H-HC-0.021300	0	8.886228	4.273052	-2.844110	L		
H-H1-0.039100	0	8.268459	2.767435	-4.525343	L		
H-H1-0.039100	0	9.809465	2.958459	-5.406712	L		
N-N--0.254800	0	10.950204	2.128733	-0.161644	L		
C-CT--0.026600	-1	10.398719	2.116525	1.186167	L		
C-C-0.589600	0	9.348121	3.201526	1.423237	L		
O-O--0.574800	0	9.614276	4.382363	1.211135	L		
C-CT--0.007000	0	11.599837	2.297599	2.125245	L		
C-CT-0.018900	0	12.784964	1.809940	1.295578	L		
C-CT-0.019200	0	12.400275	2.229262	-0.120310	L		
H-H1-0.064100	0	9.956903	1.135382	1.366057	L		
H-HC-0.025300	0	11.483072	1.717162	3.040980	L		
H-HC-0.025300	0	11.744690	3.353037	2.363090	L		
H-HC-0.021300	0	12.847803	0.721709	1.349080	L		
H-HC-0.021300	0	13.721638	2.268093	1.614001	L		
H-H1-0.039100	0	12.698744	3.263522	-0.296826	L		
H-H1-0.039100	0	12.875081	1.563492	-0.841808	L		
N-N--0.4157	0	8.176879	2.776140	1.886901	L		
C-CT-0.0188	-1	7.021665	3.605737	2.184360	L	H-HC	334
C-C-0.5973	0	6.156611	3.012907	3.294114	L		
O-O--0.5679	0	6.179781	1.804282	3.525625	L		
C-CT--0.515368	0	6.203063	3.729779	0.870827	H		
C-CM-0.287903	0	5.599000	2.457228	0.354008	H		
N-N2--0.582625	0	6.214174	1.669911	-0.606757	H		
C-CM--0.036893	0	4.471988	1.744477	0.692027	H		
C-CM-0.243454	0	5.470639	0.550616	-0.802368	H		
N-N2--0.523998	0	4.408692	0.561079	-0.017705	H		

H-H-0.2719	0	8.033993	1.779124	1.960846	L		
H-H1-0.0881	0	7.356857	4.599071	2.497741	L		
H-HC-0.162301	0	6.862940	4.181858	0.115838	H		
H-HC-0.188340	0	5.413499	4.469771	1.051222	H		
H-H-0.339448	0	7.125542	1.866118	-1.020685	H		
H-H4-0.185957	0	3.708065	2.005928	1.408192	H		
H-H5-0.211295	0	5.732591	-0.239352	-1.490841	H		
N-N--0.415700	0	5.379885	3.874753	3.938638	L		
C-CT-0.014300	-1	4.559537	3.595288	5.108181	L		
C-C-0.597300	0	5.214396	2.645309	6.121436	L		
O-O--0.567900	0	4.693386	1.592215	6.492921	L		
C-CT--0.204100	0	3.091710	3.349882	4.734120	L		
C-C-0.713000	0	2.199658	4.205309	5.616947	L		
N-N--0.919100	0	1.652081	3.682548	6.678576	L		
O-O--0.593100	0	2.019178	5.391231	5.402104	L		
H-H-0.271900	0	5.510123	4.861284	3.708083	L		
H-H1-0.104800	0	4.557706	4.553771	5.632476	L		
H-HC-0.079700	0	2.849029	2.298547	4.847123	L		
H-HC-0.079700	0	2.911654	3.642820	3.700456	L		
H-H-0.419600	0	1.013046	4.251857	7.189536	L		
H-H-0.419600	0	1.683880	2.667039	6.766605	L		
N-N--0.4157	0	6.401275	3.078555	6.559622	L		
C-CT--0.1490	-1	7.306895	2.339688	7.418103	L		
H-H-0.2719	0	6.711077	3.952168	6.156796	L		
H-H1-0.0976	0	8.060475	3.006407	7.836670	L		
H-H1-0.0976	0	7.794873	1.552206	6.840611	L		
H-H1-0.0976	0	6.747165	1.866621	8.228057	L		
C-CT--0.3662	-1	-2.236532	-2.732040	9.545136	L		
C-C-0.5972	0	-2.345996	-3.832204	8.525954	L		
O-O--0.5679	0	-2.867667	-4.902206	8.829060	L		
H-HC-0.1123	0	-1.186385	-2.486851	9.692110	L		
H-HC-0.1123	0	-2.674342	-3.068610	10.482356	L		
H-HC-0.1123	0	-2.768819	-1.855224	9.181045	L		
N-N--0.347900	0	-1.829461	-3.570261	7.328801	L		
C-CT--0.240000	-1	-1.732259	-4.535956	6.228463	L		
C-C-0.734100	0	-0.321282	-4.573902	5.651701	L		
O-O--0.589400	0	0.507437	-3.712940	5.932316	L		
C-CT--0.009400	0	-2.832271	-4.234356	5.190096	L		
C-CT-0.018700	0	-2.563892	-2.999769	4.318367	L	H-HC	379
C-CT--0.464524	0	-3.724317	-2.765828	3.341875	H		
C-CT--0.195159	0	-3.505156	-1.638990	2.335975	H		
N-N3--0.748751	0	-2.379901	-1.944576	1.391668	H		
H-H-0.274700	0	-1.451442	-2.636071	7.182551	L		
H-H1-0.142600	0	-1.924663	-5.530715	6.630305	L		
H-HC-0.036200	0	-3.781877	-4.101792	5.711618	L		
H-HC-0.036200	0	-2.945291	-5.092368	4.534820	L		
H-HC-0.010300	0	-1.641047	-3.147210	3.757012	L		
H-HC-0.010300	0	-2.459826	-2.126257	4.952426	L		
H-HC-0.173903	0	-4.626784	-2.546963	3.919954	H		
H-HC-0.148535	0	-3.913919	-3.701033	2.793400	H		
H-HP-0.236066	0	-3.296986	-0.674223	2.797704	H		
H-HP-0.172509	0	-4.396481	-1.540052	1.704449	H		
H-H-0.437293	0	-2.526343	-1.453764	0.432388	H		
H-H-0.366736	0	-2.408134	-2.950027	1.177419	H		

H-H-0.419451	0	-1.434969	-1.704911	1.759570	H
N-N--0.415700	0	-0.031322	-5.598130	4.862803	L
C-CT--0.025200	-1	1.288105	-5.805149	4.274743	L
C-C-0.597300	0	1.162703	-6.551345	2.943998	L
O-O--0.567900	0	1.706674	-7.636496	2.749473	L
H-H-0.271900	0	-0.780492	-6.238036	4.617873	L
H-H1-0.069800	0	1.907340	-6.391635	4.953502	L
H-H1-0.069800	0	1.776783	-4.848273	4.087275	L
N-N--0.415700	0	0.382690	-5.954478	2.047797	L
C-CT--0.025200	-1	-0.224684	-6.544152	0.856437	L
C-C-0.597300	0	-1.318388	-5.589704	0.418897	L
O-O--0.567900	0	-2.120440	-5.164415	1.246810	L
H-H-0.271900	0	-0.021746	-5.079417	2.355728	L
H-H1-0.069800	0	-0.661639	-7.512177	1.100953	L
H-H1-0.069800	0	0.526761	-6.653854	0.074184	L
N-N--0.4157	0	-1.333538	-5.219725	-0.858432	L
C-CT--0.0014	-1	-2.153899	-4.116313	-1.359174	L
C-C-0.5973	0	-3.632621	-4.205204	-0.956842	L
O-O--0.5679	0	-4.244640	-3.231421	-0.536809	L
C-CT--0.0152	0	-2.029300	-4.029643	-2.892809	L
C-CA--0.0011	0	-0.640380	-4.263145	-3.461172	L
C-CA--0.1906	0	0.387666	-3.329280	-3.230335	L
C-CA--0.1906	0	-0.381147	-5.417941	-4.227018	L
C-CA--0.2341	0	1.673146	-3.551342	-3.760633	L
C-CA--0.2341	0	0.902756	-5.642058	-4.759626	L
C-C-0.3226	0	1.934069	-4.705912	-4.525809	L
O-OH--0.5579	0	3.168761	-4.920453	-5.053580	L
H-H-0.2719	0	-0.569724	-5.511199	-1.451418	L
H-H1-0.0876	0	-1.748160	-3.192346	-0.949563	L
H-HC-0.0295	0	-2.357248	-3.041156	-3.209691	L
H-HC-0.0295	0	-2.712247	-4.749673	-3.344864	L
H-HA-0.1699	0	0.196051	-2.432365	-2.649101	L
H-HA-0.1699	0	-1.176182	-6.121897	-4.424605	L
H-HA-0.1656	0	2.460962	-2.836170	-3.580828	L
H-HA-0.1656	0	1.093604	-6.519265	-5.355575	L
H-HO-0.3992	0	3.176593	-5.708079	-5.594383	L
N-N--0.415700	0	-4.206403	-5.402557	-1.061856	L
C-CT--0.024900	-1	-5.552900	-5.659039	-0.569599	L
C-C-0.597300	0	-5.673599	-5.561208	0.948296	L
O-O--0.567900	0	-5.005212	-6.285782	1.695428	L
C-CT-0.211700	0	-6.016556	-7.055589	-0.986195	L
O-OH--0.654600	-1	-7.272118	-7.359856	-0.401829	L
H-H-0.271900	0	-3.618143	-6.159132	-1.361154	L
H-H1-0.084300	0	-6.229854	-4.933743	-1.021838	L
H-H1-0.035200	0	-5.284413	-7.791544	-0.651781	L
H-H1-0.035200	0	-6.093370	-7.102411	-2.072672	L
H-HO-0.427500	-1	-7.529485	-8.233205	-0.707310	L
N-N--0.415700	0	-6.659894	-4.775078	1.373394	L
C-CT-0.033700	-1	-7.147828	-4.677412	2.737981	L
C-C-0.597300	0	-7.468419	-6.011710	3.417183	L
O-O--0.567900	0	-7.333075	-6.136721	4.632242	L
C-CT--0.182500	0	-8.362158	-3.748579	2.740788	L
H-H-0.271900	0	-7.130618	-4.206821	0.675252	L
H-H1-0.082300	0	-6.367420	-4.204875	3.335491	L

H-HC-0.060300	0	-8.097852	-2.778940	2.314461	L		
H-HC-0.060300	0	-8.703702	-3.607027	3.761535	L		
H-HC-0.060300	0	-9.169423	-4.195450	2.166444	L		
N-N--0.415700	0	-7.906090	-7.004740	2.641964	L		
C-CT--0.025200	-1	-8.270788	-8.322593	3.148961	L		
C-C-0.597300	0	-7.316797	-9.438376	2.737973	L		
O-O--0.567900	0	-7.742170	-10.578995	2.568658	L		
H-H-0.271900	0	-7.936723	-6.824593	1.645010	L		
H-H1-0.069800	0	-9.261411	-8.575716	2.772351	L		
H-H1-0.069800	0	-8.325840	-8.314663	4.237838	L		
N-N--0.415700	0	-6.024574	-9.132493	2.628276	L		
C-CT--0.024900	-1	-4.953955	-10.130670	2.627085	L		
C-C-0.597300	0	-4.241271	-10.091219	3.973361	L		
O-O--0.567900	0	-3.922640	-9.014088	4.486626	L		
C-CT-0.211700	0	-3.973393	-9.846951	1.484884	L		
O-OH--0.654600	-1	-3.076083	-10.932859	1.309090	L		
H-H-0.271900	0	-5.767930	-8.160090	2.766786	L		
H-H1-0.084300	0	-5.372155	-11.125205	2.471627	L		
H-H1-0.035200	0	-3.416906	-8.931335	1.692416	L		
H-H1-0.035200	0	-4.539234	-9.710596	0.562468	L		
H-HO-0.427500	-1	-2.536361	-10.745763	0.537504	L		
N-N--0.4157	0	-4.038225	-11.267103	4.566982	L		
C-CT--0.1490	-1	-3.513659	-11.409260	5.914540	L		
H-H-0.2719	0	-4.312443	-12.088118	4.051352	L		
H-H1-0.0976	0	-3.273119	-12.452384	6.119732	L		
H-H1-0.0976	0	-4.252158	-11.059566	6.639317	L		
H-H1-0.0976	0	-2.609990	-10.805525	6.028196	L		
C-CT--0.3662	-1	-12.140566	5.068186	-0.651171	L		
C-C-0.5972	0	-10.714045	5.259523	-0.207357	L		
O-O--0.5679	0	-10.403783	5.012887	0.952870	L		
H-HC-0.1123	0	-12.532487	6.014108	-1.018317	L		
H-HC-0.1123	0	-12.730518	4.731146	0.199476	L		
H-HC-0.1123	0	-12.169655	4.313288	-1.434804	L		
N-N--0.415700	0	-9.862279	5.688893	-1.133876	L		
C-CT--0.025200	-1	-8.429539	5.860694	-0.908992	L		
C-C-0.597300	0	-7.610806	5.690730	-2.165280	L		
O-O--0.567900	0	-8.129431	5.944687	-3.259956	L		
H-H-0.271900	0	-10.170012	5.826181	-2.084380	L		
H-H1-0.069800	0	-8.117978	5.103402	-0.200469	L		
H-H1-0.069800	0	-8.228060	6.844590	-0.487936	L		
N-N--0.415700	0	-6.343957	5.298981	-1.986213	L	H-H1	489
C-CT--0.530209	-1	-5.346938	5.283488	-3.055749	H		
C-C-0.568093	0	-4.446850	4.065073	-2.935940	H		
O-O--0.528105	0	-4.128327	3.586310	-1.827709	H		
C-CT-0.298500	0	-4.519107	6.593449	-3.062241	L	H-H1	489
C-CT--0.319200	0	-5.378336	7.793869	-3.466216	L		
C-CT--0.319200	0	-3.843202	6.891291	-1.719760	L		
H-H-0.271900	0	-6.023234	5.057105	-1.050242	L		
H-H1-0.184096	0	-5.886291	5.243295	-4.007660	H		
H-HC--0.029700	0	-3.736862	6.497109	-3.814387	L		
H-HC-0.079100	0	-6.116098	8.002514	-2.693581	L		
H-HC-0.079100	0	-5.887804	7.576774	-4.403827	L		
H-HC-0.079100	0	-4.739667	8.666263	-3.596883	L		
H-HC-0.079100	0	-4.585532	7.025754	-0.935090	L		

H-HC-0.079100	0	-3.233160	7.789206	-1.801660	L		
H-HC-0.079100	0	-3.192986	6.060137	-1.441724	L		
N-N--0.610103	0	-3.973457	3.548150	-4.077767	H		
C-CT--0.314060	-1	-4.236450	3.987420	-5.431093	H		
C-C-0.597300	0	-5.353324	3.164070	-6.062021	L	H-H1	505
O-O--0.567900	0	-5.487276	1.960966	-5.840192	L		
C-CT--0.110200	0	-2.943832	3.892282	-6.279237	L	H-H1	505
C-CT-0.353100	0	-1.922686	5.031677	-6.047956	L		
C-CT--0.412100	0	-1.090020	4.902534	-4.771989	L		
C-CT--0.412100	0	-0.935730	5.078334	-7.219272	L		
H-H-0.398207	0	-3.390260	2.714993	-3.988252	H		
H-H1-0.165130	0	-4.547715	5.031672	-5.438159	H		
H-HC-0.045700	0	-3.242131	3.930739	-7.326952	L		
H-HC-0.045700	0	-2.461402	2.928488	-6.120085	L		
H-HC--0.036100	0	-2.455233	5.983196	-6.026593	L		
H-HC-0.100000	0	-0.537587	3.964666	-4.776870	L		
H-HC-0.100000	0	-1.740869	4.910165	-3.897820	L		
H-HC-0.100000	0	-0.396315	5.736849	-4.683943	L		
H-HC-0.100000	0	-0.383366	4.140713	-7.269830	L		
H-HC-0.100000	0	-0.235027	5.899925	-7.072150	L		
H-HC-0.100000	0	-1.474711	5.236575	-8.151520	L		
N-N--0.415700	0	-6.139642	3.811702	-6.917238	L		
C-CT--0.038900	-1	-7.329512	3.213593	-7.535338	L		
C-C-0.597300	0	-7.084870	1.876284	-8.223761	L		
O-O--0.567900	0	-7.967785	1.021933	-8.269131	L		
C-CT-0.365400	-1	-7.968313	4.136116	-8.597814	L		
O-OH--0.676100	-1	-7.906820	5.494021	-8.151983	L		
C-CT--0.243800	0	-9.447170	3.846313	-8.854803	L		
H-H-0.271900	0	-6.012931	4.808782	-7.007880	L		
H-H1-0.100700	0	-8.068275	3.073789	-6.753095	L		
H-H1-0.004300	0	-7.414159	4.058766	-9.534259	L		
H-HC-0.064200	0	-9.563557	2.842346	-9.262508	L		
H-HC-0.064200	0	-9.842079	4.559962	-9.577645	L		
H-HC-0.064200	0	-10.009405	3.917945	-7.922943	L		
H-HO-0.410200	-1	-8.805143	5.828852	-8.116102	L		
N-N--0.415700	0	-5.908337	1.739374	-8.832103	L		
C-CT--0.025200	-1	-5.523289	0.582039	-9.626760	L		
C-C-0.597300	0	-4.627346	-0.421814	-8.931994	L		
O-O--0.567900	0	-4.146155	-1.336394	-9.601863	L		
H-H-0.271900	0	-5.220610	2.452370	-8.640646	L		
H-H1-0.069800	0	-4.993843	0.928554	-10.513045	L		
H-H1-0.069800	0	-6.412200	0.047734	-9.961932	L		
N-N--0.415700	0	-4.396953	-0.233387	-7.627880	L	H-H1	545
C-CT--0.539097	-1	-3.476419	-1.023811	-6.822361	H		
C-C-0.572106	0	-4.234689	-1.451256	-5.567252	H		
O-O--0.533001	0	-5.029880	-2.382495	-5.680235	H		
C-CT-0.130300	0	-2.109914	-0.308700	-6.651245	L	H-H1	545
C-CT--0.043000	0	-1.491057	0.128848	-8.000897	L		
C-CT--0.320400	0	-1.171513	-1.288328	-5.925221	L		
C-CT--0.066000	0	-0.161503	0.885394	-7.879397	L		
H-H-0.271900	0	-4.855031	0.541979	-7.157025	L		
H-H1-0.180646	0	-3.286501	-1.953369	-7.370080	H		
H-HC-0.018700	0	-2.251488	0.579236	-6.040212	L		
H-HC-0.023600	0	-2.182149	0.803024	-8.504964	L		

```
H-HC-0.023600   0    -1.346354   -0.747001   -8.634551 L
H-HC-0.088200   0    -1.031115   -2.188724   -6.521496 L
H-HC-0.088200   0    -1.584861   -1.562520   -4.956865 L
H-HC-0.088200   0    -0.206484   -0.827581   -5.733051 L
H-HC-0.018600   0    -0.276605    1.722432   -7.191583 L
H-HC-0.018600   0     0.124093    1.269017   -8.858859 L
H-HC-0.018600   0     0.627792    0.223537   -7.524694 L
N-N--0.611145   0    -4.053057   -0.725705   -4.433659 H
C-CT--0.109296  -1   -4.637112   -1.120445   -3.158988 H
C-C-0.597300    0    -6.163693   -1.169160   -3.203033 L H-H1    564
O-O--0.567900   0    -6.792455   -2.117361   -2.716553 L
C-CT--0.388038  0    -4.175913   -0.259157   -1.986476 H
H-H-0.369964    0    -3.273092   -0.083391   -4.360333 H
H-H1-0.178541   0    -4.353724   -2.153602   -2.932026 H
H-H1-0.157964   0    -4.845834   -0.527497   -1.161631 H
H-H1-0.179833   0    -4.349557    0.800256   -2.203744 H
H-HS-0.319500   0    -2.147207    0.904677    0.102879 H
N-N--0.415700   0    -6.755424   -0.159874   -3.828404 L
C-CT--0.087500  -1   -8.195185   -0.083427   -4.053003 L
C-C-0.597300    0    -8.696173   -1.224540   -4.926543 L
O-O--0.567900   0    -9.653842   -1.906555   -4.560752 L
C-CT-0.298500   0    -8.589955    1.285646   -4.623722 L
C-CT--0.319200  0   -10.107733    1.425651   -4.804539 L
C-CT--0.319200  0    -8.128615    2.445838   -3.728539 L
H-H-0.271900    0    -6.118992    0.530827   -4.220143 L
H-H1-0.096900   0    -8.688453   -0.188735   -3.089342 L
H-HC--0.029700  0    -8.119557    1.379271   -5.594101 L
H-HC-0.079100   0   -10.610310    1.270161   -3.849644 L
H-HC-0.079100   0   -10.468803    0.693857   -5.526912 L
H-HC-0.079100   0   -10.342534    2.421365   -5.179721 L
H-HC-0.079100   0    -8.572972    2.366014   -2.737938 L
H-HC-0.079100   0    -8.410049    3.398629   -4.175335 L
H-HC-0.079100   0    -7.042721    2.443201   -3.622567 L
N-N--0.415700   0    -8.052109   -1.446713   -6.070649 L
C-CT--0.025200  -1   -8.407222   -2.505167   -7.011660 L
C-C-0.597300    0    -8.321705   -3.905812   -6.418496 L
O-O--0.567900   0    -9.219915   -4.722055   -6.614016 L
H-H-0.271900    0    -7.235728   -0.877435   -6.256868 L
H-H1-0.069800   0    -7.735031   -2.456057   -7.868061 L
H-H1-0.069800   0    -9.425930   -2.342788   -7.363126 L
N-N--0.415700   0    -7.240098   -4.179946   -5.692333 L
C-CT--0.025200  -1   -7.009795   -5.443989   -5.004428 L
C-C-0.597300    0    -7.938381   -5.707341   -3.830047 L
O-O--0.567900   0    -8.267579   -6.859759   -3.554420 L
H-H-0.271900    0    -6.531974   -3.450635   -5.623592 L
H-H1-0.069800   0    -5.985610   -5.457565   -4.633421 L
H-H1-0.069800   0    -7.126811   -6.261162   -5.716315 L
N-N--0.415700   0    -8.328791   -4.649645   -3.123248 L
C-CT--0.038900  -1   -9.377419   -4.702655   -2.102566 L
C-C-0.597300    0   -10.749405   -4.993868   -2.701247 L
O-O--0.567900   0   -11.492547   -5.827154   -2.182381 L
C-CT-0.365400   0    -9.409170   -3.396363   -1.294990 L
O-OH--0.676100  0    -8.166536   -3.223448   -0.652397 L
C-CT--0.243800  0   -10.486941   -3.406033   -0.211450 L
```

H-H-0.271900	0	-7.936784	-3.747002	-3.371222	L
H-H1-0.100700	0	-9.149787	-5.515399	-1.413033	L
H-H1-0.004300	0	-9.583398	-2.549178	-1.958640	L
H-HC-0.064200	0	-11.469919	-3.291103	-0.666969	L
H-HC-0.064200	0	-10.321743	-2.580718	0.480512	L
H-HC-0.064200	0	-10.455917	-4.348629	0.332941	L
H-HO-0.410200	0	-7.578430	-2.783339	-1.299148	L
N-N--0.4157	0	-11.100345	-4.288931	-3.778236	L
C-CT--0.1490	-1	-12.332538	-4.485671	-4.513909	L
H-H-0.2719	0	-10.435824	-3.604877	-4.129611	L
H-H1-0.0976	0	-12.411190	-3.733568	-5.299934	L
H-H1-0.0976	0	-13.184744	-4.402771	-3.838053	L
H-H1-0.0976	0	-12.327157	-5.478555	-4.966714	L
N-N2--0.511415	0	1.935025	0.045264	-1.709328	H
C-CA-0.057801	0	0.761748	0.692050	-1.669526	H
C-CA-0.063496	0	0.172197	1.235813	-2.815549	H
C-CA--0.109122	0	0.846387	1.123906	-4.031056	H
C-CA--0.164490	0	2.077076	0.483424	-4.064017	H
C-CA-0.098392	0	2.579339	-0.051038	-2.885751	H
C-C-0.541263	0	-1.184888	1.849651	-2.847486	H
O-O--0.574884	0	-1.866666	1.754782	-3.866964	H
H-H4-0.220889	0	0.273306	0.762572	-0.703839	H
H-HA-0.160434	0	0.391535	1.529594	-4.926752	H
H-HA-0.142191	0	2.638562	0.393655	-4.985175	H
H-H4-0.174502	0	3.517421	-0.594756	-2.865613	H
H-H-0.353801	0	-2.467081	2.874081	-1.844701	H
Zn-ZN-0.974537	0	2.806776	-1.058897	-0.020609	H
O-OW--0.834000	0	-2.157943	-7.210545	3.463264	L
H-HW-0.417000	0	-2.651863	-6.596258	2.901014	L
H-HW-0.417000	0	-2.846310	-7.781831	3.842181	L
O-OW--0.783903	0	1.329919	-2.561218	-0.130807	H
H-HW-0.401253	0	0.929992	-2.440856	0.767715	H
H-HW-0.424111	0	1.938939	-3.342242	-0.161591	H
O-OW--0.834000	0	-3.811182	-8.461474	7.252830	L
H-HW-0.417000	0	-3.819937	-7.516169	7.424248	L
H-HW-0.417000	0	-3.873135	-8.525399	6.288070	L
O-OW--0.834000	0	7.144788	-0.007909	1.769668	L
H-HW-0.417000	0	6.843005	0.410191	2.596878	L
H-HW-0.417000	0	6.351997	0.075309	1.215906	L
O-OW--0.834000	0	7.036436	-4.943712	4.740652	L
H-HW-0.417000	0	6.087686	-4.994735	4.976571	L
H-HW-0.417000	0	7.226213	-5.819143	4.401436	L
O-OW--0.834000	0	8.646113	-0.915050	-0.250545	L
H-HW-0.417000	0	8.153076	-0.545055	0.510411	L
H-HW-0.417000	0	8.846339	-0.125851	-0.774253	L
O-OW--0.834000	0	12.658699	-1.076639	-2.013454	L
H-HW-0.417000	0	11.800146	-0.826867	-2.396396	L
H-HW-0.417000	0	12.659697	-2.040571	-2.117296	L
O-OW--0.834000	0	8.308704	-3.285199	0.989799	L
H-HW-0.417000	0	8.519486	-2.514069	0.422719	L
H-HW-0.417000	0	7.574188	-2.952090	1.515579	L
O-OW--0.834000	0	4.347378	-4.876065	5.153395	L
H-HW-0.417000	0	4.085831	-4.502642	6.017819	L
H-HW-0.417000	0	4.046532	-4.196837	4.538426	L

S-SH--0.326142	0	-2.439684	-0.557031	-1.448185	H	
O-OW--0.834000	0	-1.511792	2.577632	-1.789161	H	

TS4

C-CT--0.366200	-1	-10.305510	3.904795	4.028475	L	
C-C-0.597200	0	-8.980705	3.243119	3.785691	L	
O-O--0.567900	0	-8.421942	2.648792	4.699668	L	
H-HC-0.112300	0	-11.048066	3.460994	3.368331	L	
H-HC-0.112300	0	-10.213265	4.968789	3.817553	L	
H-HC-0.112300	0	-10.594056	3.755014	5.066420	L	
N-N--0.415700	0	-8.506506	3.351183	2.552289	L	
C-CT--0.059700	-1	-7.338927	2.637417	2.027970	L	
C-C-0.597300	0	-6.188774	3.617652	1.848734	L	
O-O--0.567900	0	-6.333100	4.601347	1.128386	L	
C-CT-0.130300	0	-7.712748	1.928927	0.702964	L	
C-CT--0.043000	0	-8.837726	0.888907	0.934659	L	
C-CT--0.320400	0	-6.484930	1.246614	0.079622	L	
C-CT--0.066000	0	-9.415915	0.293210	-0.353946	L	
H-H-0.271900	0	-9.033798	3.931055	1.908249	L	
H-H1-0.086900	0	-7.026122	1.873691	2.739675	L	
H-HC-0.018700	0	-8.075749	2.681165	0.000163	L	
H-HC-0.023600	0	-9.668649	1.359506	1.458903	L	
H-HC-0.023600	0	-8.462524	0.079254	1.562067	L	
H-HC-0.088200	0	-6.078145	0.495685	0.758046	L	
H-HC-0.088200	0	-5.707540	1.980673	-0.140378	L	
H-HC-0.088200	0	-6.750961	0.781718	-0.867155	L	
H-HC-0.018600	0	-9.697297	1.091002	-1.040761	L	
H-HC-0.018600	0	-10.304128	-0.289348	-0.112932	L	
H-HC-0.018600	0	-8.686543	-0.363962	-0.824847	L	
N-N--0.516300	0	-5.083394	3.354180	2.539928	L	
C-CT-0.038100	-1	-3.772911	3.973140	2.387861	L	H-HC 30
C-C-0.536600	0	-3.701146	5.490695	2.121597	L	
O-O--0.581900	0	-2.734688	6.016700	1.572385	L	
C-CT--0.547145	0	-3.013072	3.121581	1.334415	H	
C-C-0.532653	0	-2.592093	1.727493	1.803663	H	
O-O2--0.588507	0	-2.713450	1.417273	2.995653	H	
O-O2--0.625559	0	-2.023870	0.932307	0.941241	H	
H-H-0.293600	0	-5.057356	2.481102	3.053328	L	
H-H1-0.088000	0	-3.243810	3.841722	3.332109	L	
H-HC-0.128435	0	-2.081422	3.643447	1.065052	H	
H-HC-0.183556	0	-3.588984	3.087020	0.405385	H	
N-N--0.415700	0	-4.664434	6.245960	2.633659	L	
C-CT--0.025200	-1	-4.650660	7.691202	2.511863	L	
C-C-0.597300	0	-3.748634	8.332280	3.549704	L	
O-O--0.567900	0	-4.222219	9.006446	4.466014	L	
H-H-0.271900	0	-5.474488	5.743826	2.960616	L	
H-H1-0.069800	0	-5.662284	8.069793	2.649629	L	
H-H1-0.069800	0	-4.310274	7.979794	1.516089	L	
N-N--0.415700	0	-2.446098	8.109307	3.405483	L	
C-CT--0.038900	-1	-1.426634	8.554875	4.349256	L	
C-C-0.597300	0	-0.520474	9.638202	3.752417	L	
O-O--0.567900	0	-0.623349	10.008868	2.581990	L	
C-CT-0.365400	0	-0.581377	7.405602	4.944254	L	
O-OH--0.676100	0	0.616300	7.241161	4.227202	L	

C-CT--0.243800	0	-1.290884	6.059027	5.054190	L
H-H-0.271900	0	-2.183763	7.508623	2.625658	L
H-H1-0.100700	0	-1.935736	9.017570	5.192694	L
H-H1-0.004300	0	-0.301052	7.705549	5.954538	L
H-HC-0.064200	0	-2.230122	6.182587	5.591066	L
H-HC-0.064200	0	-0.655900	5.362929	5.597267	L
H-HC-0.064200	0	-1.488277	5.649340	4.063756	L
H-HO-0.410200	0	1.108044	6.482832	4.610923	L
N-N--0.415700	0	0.397373	10.150742	4.576210	L
C-CT--0.025200	-1	1.439799	11.083195	4.161113	L
C-C-0.597300	0	2.339579	10.593010	3.035878	L
O-O--0.567900	0	2.674520	11.369955	2.146585	L
H-H-0.271900	0	0.464250	9.739894	5.492015	L
H-H1-0.069800	0	2.075559	11.315142	5.014394	L
H-H1-0.069800	0	0.969517	12.008176	3.826842	L
N-N--0.516300	0	2.735539	9.323715	3.091555	L
C-CT-0.038100	-1	3.645142	8.727581	2.113955	L
C-C-0.536600	0	3.016550	8.443279	0.763346	L
O-O--0.581900	0	3.750926	8.160876	-0.176884	L
C-CT--0.030300	0	4.231228	7.413764	2.662084	L
C-C-0.799400	-1	5.577323	7.615963	3.340580	L
O-O2--0.801400	-1	6.157018	8.720399	3.257988	L
O-O2--0.801400	-1	6.038238	6.663041	4.001426	L
H-H-0.293600	0	2.361126	8.716228	3.806761	L
H-H1-0.088000	0	4.447229	9.435495	1.902638	L
H-HC--0.012200	0	4.389382	6.702134	1.849966	L
H-HC--0.012200	0	3.528579	6.954022	3.360223	L
N-N--0.415700	0	1.694086	8.521586	0.652115	L
C-CT--0.002400	-1	0.972980	8.314948	-0.596703	L
C-C-0.597300	0	0.379097	9.608721	-1.155223	L
O-O--0.567900	0	0.361460	9.818509	-2.368306	L
C-CT--0.034300	0	-0.098366	7.232704	-0.410626	L
C-CA-0.011800	0	0.425598	5.915175	0.136791	L
C-CA--0.125600	0	1.067137	4.985316	-0.701663	L
C-CA--0.125600	0	0.239679	5.604502	1.493730	L
C-CA--0.170400	0	1.499377	3.751732	-0.179408	L
C-CA--0.170400	0	0.649673	4.366920	2.013621	L
C-CA--0.107200	0	1.272610	3.436077	1.169940	L
H-H-0.271900	0	1.174406	8.705976	1.497406	L
H-H1-0.097800	0	1.659357	7.941452	-1.355830	L
H-HC-0.029500	0	-0.570967	7.039053	-1.374736	L
H-HC-0.029500	0	-0.872747	7.613121	0.257173	L
H-HA-0.133000	0	1.186778	5.198516	-1.752459	L
H-HA-0.133000	0	-0.279838	6.305375	2.120865	L
H-HA-0.143000	0	1.955801	3.015069	-0.820747	L
H-HA-0.143000	0	0.441114	4.114736	3.041927	L
H-HA-0.129700	0	1.537836	2.462089	1.546774	L
N-N--0.415700	0	-0.076091	10.504115	-0.276125	L
C-CT--0.149000	-1	-0.477226	11.848254	-0.639336	L
H-H-0.271900	0	-0.069165	10.242632	0.704549	L
H-H1-0.097600	0	-0.865972	12.362566	0.240010	L
H-H1-0.097600	0	-1.249828	11.803826	-1.408727	L
H-H1-0.097600	0	0.385348	12.391873	-1.027564	L
C-CT--0.366200	-1	7.983419	8.433592	-0.169439	L

C-C-0.597200	0	7.522121	7.637049	-1.367594	L
O-O--0.567900	0	8.343301	7.294252	-2.214823	L
H-HC-0.112300	0	7.515183	9.415332	-0.185173	L
H-HC-0.112300	0	9.065725	8.534389	-0.200394	L
H-HC-0.112300	0	7.696747	7.910063	0.743512	L
N-N--0.415700	0	6.213293	7.384694	-1.460907	L
C-CT--0.051800	-1	5.560255	6.855334	-2.665896	L
C-C-0.597300	0	4.923315	7.983851	-3.476929	L
O-O--0.567900	0	5.317504	8.259794	-4.610534	L
C-CT--0.110200	0	4.613658	5.681920	-2.325243	L
C-CT-0.353100	0	4.514804	4.650381	-3.470035	L
C-CT--0.412100	0	3.767265	3.399099	-3.022223	L
C-CT--0.412100	0	3.790763	5.170098	-4.714802	L
H-H-0.271900	0	5.609296	7.704663	-0.710855	L
H-H1-0.092200	0	6.345791	6.447690	-3.303332	L
H-HC-0.045700	0	3.621602	6.049957	-2.062014	L
H-HC-0.045700	0	5.014738	5.164557	-1.453522	L
H-HC--0.036100	0	5.522566	4.344621	-3.754834	L
H-HC-0.100000	0	2.709861	3.622127	-2.884321	L
H-HC-0.100000	0	4.179086	3.027292	-2.085639	L
H-HC-0.100000	0	3.869601	2.622396	-3.777324	L
H-HC-0.100000	0	2.821541	5.584730	-4.434684	L
H-HC-0.100000	0	3.639452	4.356741	-5.423538	L
H-HC-0.100000	0	4.387388	5.937175	-5.203291	L
N-N--0.415700	0	3.966989	8.658977	-2.853487	L
C-CT--0.149000	-1	3.350952	9.884303	-3.292633	L
H-H-0.271900	0	3.764462	8.339965	-1.913161	L
H-H1-0.097600	0	4.065498	10.480372	-3.861681	L
H-H1-0.097600	0	3.012093	10.454853	-2.426643	L
H-H1-0.097600	0	2.490931	9.654230	-3.923697	L
C-CT--0.366200	-1	-8.714958	2.362798	8.135556	L
C-C-0.597200	0	-7.869698	1.133692	7.956901	L
O-O--0.567900	0	-7.579792	0.435198	8.921673	L
H-HC-0.112300	0	-9.618979	2.259628	7.538419	L
H-HC-0.112300	0	-8.150301	3.231717	7.801104	L
H-HC-0.112300	0	-8.971848	2.468882	9.187310	L
N-N--0.415700	0	-7.466280	0.889503	6.718838	L
C-CT--0.038900	-1	-6.653415	-0.254200	6.302645	L
C-C-0.597300	0	-5.399293	0.253427	5.616300	L
O-O--0.567900	0	-5.466904	0.726319	4.483386	L
C-CT-0.365400	0	-7.450407	-1.229790	5.423454	L
O-OH--0.676100	0	-8.014922	-0.575181	4.313317	L
C-CT--0.243800	0	-8.590358	-1.865022	6.225106	L
H-H-0.271900	0	-7.776106	1.523372	5.986008	L
H-H1-0.100700	0	-6.341616	-0.812270	7.185083	L
H-H1-0.004300	0	-6.783468	-2.017854	5.072508	L
H-HC-0.064200	0	-8.184505	-2.389751	7.089623	L
H-HC-0.064200	0	-9.135151	-2.571682	5.605804	L
H-HC-0.064200	0	-9.287295	-1.097731	6.560571	L
H-HO-0.410200	0	-7.339354	0.037155	3.985326	L
N-N--0.415700	0	-4.295423	0.220082	6.367751	L
C-CT--0.059700	-1	-3.101053	1.042998	6.163611	L
C-C-0.597300	0	-1.854051	0.165173	6.097484	L
O-O--0.567900	0	-1.592967	-0.626264	7.009584	L

C-CT-0.130300	0	-2.984792	2.105273	7.282276	L	
C-CT--0.043000	0	-4.244549	2.991385	7.450215	L	
C-CT--0.320400	0	-1.748536	2.989765	7.057680	L	
C-CT--0.066000	0	-4.665426	3.803812	6.216357	L	
H-H-0.271900	0	-4.347073	-0.253831	7.254159	L	
H-H1-0.086900	0	-3.200892	1.579500	5.225962	L	
H-HC-0.018700	0	-2.840665	1.581434	8.229125	L	
H-HC-0.023600	0	-4.069091	3.688994	8.269866	L	
H-HC-0.023600	0	-5.083856	2.364392	7.749680	L	
H-HC-0.088200	0	-1.747689	3.366472	6.034519	L	
H-HC-0.088200	0	-0.843450	2.400637	7.208058	L	
H-HC-0.088200	0	-1.740361	3.822264	7.759837	L	
H-HC-0.018600	0	-3.868605	4.481831	5.916065	L	
H-HC-0.018600	0	-5.552384	4.390018	6.456846	L	
H-HC-0.018600	0	-4.904720	3.141899	5.387234	L	
N-N--0.516300	0	-1.107262	0.330982	5.012359	L	
C-CT-0.038100	-1	0.194376	-0.241716	4.725606	L	H-HC 17
C-C-0.536600	0	1.133006	-0.086360	5.927302	L	
O-O--0.581900	0	1.341201	1.004224	6.452647	L	
C-CT--0.520019	0	0.676818	0.428194	3.423487	H	
C-C-0.636404	0	0.951447	-0.508142	2.259977	H	
O-O2--0.608036	0	0.339673	-1.614242	2.155639	H	
O-O2--0.608131	0	1.805027	-0.063567	1.426017	H	
H-H-0.293600	0	-1.516495	0.905646	4.279873	L	
H-H1-0.088000	0	0.049658	-1.309007	4.551637	L	
H-HC-0.132300	0	1.589422	0.993070	3.625837	H	
H-HC-0.188993	0	-0.068061	1.149484	3.069945	H	
N-N--0.415700	0	1.682483	-1.196900	6.386695	L	
C-CT-0.033700	-1	2.581591	-1.242323	7.517645	L	
C-C-0.597300	0	3.804582	-2.061498	7.120818	L	
O-O--0.567900	0	3.859635	-3.285663	7.285016	L	
C-CT--0.182500	0	1.824117	-1.801499	8.730281	L	
H-H-0.271900	0	1.379273	-2.073351	5.977116	L	
H-H1-0.082300	0	2.925028	-0.236879	7.768732	L	
H-HC-0.060300	0	0.983030	-1.147611	8.964275	L	
H-HC-0.060300	0	2.493582	-1.850755	9.588693	L	
H-HC-0.060300	0	1.449009	-2.801295	8.505910	L	
N-N--0.415700	0	4.771378	-1.367117	6.533375	L	
C-CT-0.018800	-1	5.976241	-1.948310	5.985691	L	H-HC 20
C-C-0.597300	0	7.174020	-1.800777	6.933312	L	
O-O--0.567900	0	7.219353	-0.956781	7.830846	L	
C-CT--0.517456	0	6.225495	-1.307255	4.618002	H	
C-CM-0.287351	0	5.342471	-1.771628	3.517944	H	
N-N2--0.586994	0	5.475683	-3.023259	2.933113	H	
C-CM--0.020338	0	4.361741	-1.154406	2.791469	H	
C-CM-0.242458	0	4.603500	-3.102509	1.893081	H	
N-N2--0.505070	0	3.918759	-1.979845	1.777499	H	
H-H-0.271900	0	4.660535	-0.358996	6.465351	L	
H-H1-0.088100	0	5.829854	-3.014222	5.843026	L	
H-HC-0.166355	0	7.276744	-1.481829	4.345327	H	
H-HC-0.174481	0	6.124737	-0.228310	4.760094	H	
H-H-0.335795	0	6.123728	-3.743865	3.243296	H	
H-H4-0.152777	0	3.965067	-0.159016	2.923928	H	
H-H5-0.209475	0	4.497194	-3.965676	1.250979	H	

```
N-N--0.415700    0    8.180506   -2.635899    6.682342 L
C-CT--0.149000  -1    9.448854   -2.565958    7.374888 L
H-H-0.271900     0    8.030282   -3.309538    5.938182 L
H-H1-0.097600    0   10.124238   -3.331331    6.991524 L
H-H1-0.097600    0    9.290299   -2.720531    8.443539 L
H-H1-0.097600    0    9.893242   -1.580494    7.223712 L
C-CT--0.366200  -1    8.370440   -9.387169    0.917430 L
C-C-0.597200     0    8.747983   -9.160524   -0.534124 L
O-O--0.567900    0    9.781475   -8.556823   -0.794944 L
H-HC-0.112300    0    8.292493  -10.453143    1.117615 L
H-HC-0.112300    0    7.430137   -8.882693    1.132299 L
H-HC-0.112300    0    9.148315   -8.955447    1.547129 L
N-N--0.254800    0    7.928392   -9.618846   -1.496893 L
C-CT--0.026600  -1    8.168040   -9.379966   -2.922395 L
C-C-0.589600     0    8.015059   -7.907221   -3.302587 L
O-O--0.574800    0    8.722744   -7.410852   -4.175941 L
C-CT--0.007000   0    7.160695  -10.274135   -3.653004 L
C-CT-0.018900    0    6.002507  -10.400357   -2.661963 L
C-CT-0.019200    0    6.697158  -10.372291   -1.299660 L
H-H1-0.064100    0    9.180433   -9.688889   -3.185757 L
H-HC-0.025300    0    7.604951  -11.257160   -3.816310 L
H-HC-0.025300    0    6.837845   -9.843880   -4.602341 L
H-HC-0.021300    0    5.442012  -11.323895   -2.809908 L
H-HC-0.021300    0    5.343208   -9.535899   -2.754875 L
H-H1-0.039100    0    6.050387   -9.898659   -0.559726 L
H-H1-0.039100    0    6.942353  -11.389024   -0.989811 L
N-N--0.516300    0    7.093446   -7.211227   -2.635434 L
C-CT-0.039700   -1    6.841487   -5.784704   -2.810895 L
C-C-0.536600     0    8.100738   -4.919571   -2.683952 L
O-O--0.581900    0    8.274381   -3.937469   -3.409036 L
C-CT-0.056000    0    5.739785   -5.336821   -1.839432 L H-HC 24
C-CT--0.515956   0    5.220698   -3.937727   -2.196963 H
C-C-0.587828     0    4.114296   -3.403861   -1.289608 H
O-O2--0.600319   0    4.045044   -2.121335   -1.256109 H
O-O2--0.589127   0    3.359093   -4.196237   -0.682061 H
H-H-0.293600     0    6.562700   -7.685992   -1.924365 L
H-H1-0.110500    0    6.467061   -5.633234   -3.823688 L
H-HC--0.017300   0    6.128011   -5.335322   -0.819752 L
H-HC--0.017300   0    4.909684   -6.042620   -1.898951 L
H-HC-0.167759    0    4.830535   -3.966296   -3.221658 H
H-HC-0.135230    0    6.048982   -3.220569   -2.187045 H
N-N--0.415700    0    9.011343   -5.323397   -1.800654 L
C-CT--0.024900  -1   10.250453   -4.621960   -1.485864 L
C-C-0.597300     0   11.272019   -4.512249   -2.611013 L
O-O--0.567900    0   12.254243   -3.782204   -2.477059 L
C-CT-0.211700    0   10.891360   -5.257730   -0.246015 L
O-OH--0.654600   0    9.934733   -5.450652    0.785563 L
H-H-0.271900     0    8.800371   -6.129599   -1.229056 L
H-H1-0.084300    0    9.983821   -3.597857   -1.219956 L
H-H1-0.035200    0   11.689176   -4.608688    0.117961 L
H-H1-0.035200    0   11.319349   -6.224791   -0.514358 L
H-HO-0.427500    0    9.386567   -4.638442    0.869921 L
N-N--0.415700    0   11.030699   -5.194817   -3.728439 L
C-CT-0.033700   -1   11.779839   -5.030925   -4.967453 L
```

C-C-0.597300	0	10.946105	-4.475804	-6.127009	L
O-O--0.567900	0	11.378335	-4.488581	-7.278828	L
C-CT--0.182500	0	12.408816	-6.391398	-5.301712	L
H-H-0.271900	0	10.214414	-5.795891	-3.736368	L
H-H1-0.082300	0	12.594373	-4.321780	-4.813777	L
H-HC-0.060300	0	13.025813	-6.728141	-4.467909	L
H-HC-0.060300	0	13.034786	-6.299411	-6.189942	L
H-HC-0.060300	0	11.623768	-7.125214	-5.491224	L
N-N--0.415700	0	9.766272	-3.959071	-5.801378	L
C-CT--0.025200	-1	8.862248	-3.276174	-6.703869	L
C-C-0.597300	0	8.815809	-1.772239	-6.483284	L
O-O--0.567900	0	8.836338	-0.999486	-7.438510	L
H-H-0.271900	0	9.488402	-4.015047	-4.827728	L
H-H1-0.069800	0	7.855438	-3.666915	-6.559481	L
H-H1-0.069800	0	9.150999	-3.459425	-7.739285	L
N-N--0.415700	0	8.752925	-1.366676	-5.218109	L
C-CT--0.002400	-1	8.580018	0.018484	-4.797204	L
C-C-0.597300	0	9.695602	0.452663	-3.839774	L
O-O--0.567900	0	10.320975	-0.398895	-3.199031	L
C-CT--0.034300	0	7.192029	0.140648	-4.141975	L
C-CA-0.011800	0	6.048050	-0.119151	-5.105057	L
C-CA--0.125600	0	5.630860	0.890351	-5.993429	L
C-CA--0.125600	0	5.431591	-1.383906	-5.149027	L
C-CA--0.170400	0	4.615090	0.631224	-6.931582	L
C-CA--0.170400	0	4.412063	-1.641135	-6.082721	L
C-CA--0.107200	0	4.009721	-0.637230	-6.981016	L
H-H-0.271900	0	8.752299	-2.074181	-4.492857	L
H-H1-0.097800	0	8.609629	0.669059	-5.671419	L
H-HC-0.029500	0	7.065665	1.137163	-3.719723	L
H-HC-0.029500	0	7.133251	-0.566568	-3.312985	L
H-HA-0.133000	0	6.096282	1.864870	-5.963773	L
H-HA-0.133000	0	5.749968	-2.162848	-4.472091	L
H-HA-0.143000	0	4.296886	1.408809	-7.611391	L
H-HA-0.143000	0	3.935137	-2.610082	-6.108436	L
H-HA-0.129700	0	3.221726	-0.834845	-7.694366	L
N-N--0.254800	0	9.952824	1.766000	-3.696663	L
C-CT--0.026600	-1	10.818388	2.260304	-2.632526	L
C-C-0.589600	0	10.184540	2.014265	-1.254519	L
O-O--0.574800	0	8.990641	1.707200	-1.174470	L
C-CT--0.007000	0	10.979482	3.759355	-2.917642	L
C-CT-0.018900	0	9.661819	4.129211	-3.596574	L
C-CT-0.019200	0	9.348391	2.880495	-4.420687	L
H-H1-0.064100	0	11.787097	1.764853	-2.688513	L
H-HC-0.025300	0	11.803343	3.910383	-3.616643	L
H-HC-0.025300	0	11.139761	4.344803	-2.010955	L
H-HC-0.021300	0	9.760060	5.016649	-4.222567	L
H-HC-0.021300	0	8.886786	4.277233	-2.841843	L
H-H1-0.039100	0	8.269660	2.773232	-4.525045	L
H-H1-0.039100	0	9.811879	2.963741	-5.404520	L
N-N--0.254800	0	10.951306	2.129662	-0.159910	L
C-CT--0.026600	-1	10.399468	2.115527	1.187746	L
C-C-0.589600	0	9.349081	3.200465	1.426095	L
O-O--0.574800	0	9.615033	4.381414	1.214433	L
C-CT--0.007000	0	11.600468	2.295334	2.127257	L

```
C-CT-0.018900    0   12.786078    1.810112    1.296793 L
C-CT-0.019200    0   12.401214    2.231663   -0.118381 L
H-H1-0.064100    0    9.957530    1.134185    1.366230 L
H-HC-0.025300    0   11.483957    1.712972    3.041801 L
H-HC-0.025300    0   11.744670    3.350364    2.367305 L
H-HC-0.021300    0   12.849772    0.721836    1.348336 L
H-HC-0.021300    0   13.722362    2.268425    1.616131 L
H-H1-0.039100    0   12.698588    3.266572   -0.292940 L
H-H1-0.039100    0   12.876736    1.567728   -0.841099 L
N-N--0.415700    0    8.178259    2.774659    1.890247 L
C-CT-0.018800   -1    7.022299    3.603063    2.188048 L H-HC 33
C-C-0.597300     0    6.158368    3.008519    3.297749 L
O-O--0.567900    0    6.185116    1.800158    3.530171 L
C-CT--0.515368   0    6.203535    3.726522    0.874575 H
C-CM-0.287903    0    5.601948    2.453130    0.357079 H
N-N2--0.582625   0    6.219684    1.666421   -0.602591 H
C-CM--0.036893   0    4.475702    1.738942    0.694062 H
C-CM-0.243454    0    5.478483    0.545966   -0.799066 H
N-N2--0.523998   0    4.415085    0.555655   -0.016200 H
H-H-0.271900     0    8.036073    1.777502    1.963489 L
H-H1-0.088100    0    7.356482    4.596626    2.501781 L
H-HC-0.162301    0    6.862714    4.180143    0.119892 H
H-HC-0.188340    0    5.412413    4.464718    1.055243 H
H-H-0.339448     0    7.131319    1.864047   -1.015295 H
H-H4-0.185957    0    3.710987    1.998328    1.409847 H
H-H5-0.211295    0    5.742977   -0.244529   -1.485882 H
N-N--0.415700    0    5.379199    3.868602    3.941562 L
C-CT-0.014300   -1    4.560477    3.588801    5.112111 L
C-C-0.597300     0    5.215333    2.637957    6.124593 L
O-O--0.567900    0    4.694465    1.584359    6.494904 L
C-CT--0.204100   0    3.092007    3.346461    4.740959 L
C-C-0.713000     0    2.203964    4.197651    5.632199 L
N-N--0.919100    0    1.671476    3.674293    6.701284 L
O-O--0.593100    0    2.012938    5.381577    5.415988 L
H-H-0.271900     0    5.509214    4.855100    3.710840 L
H-H1-0.104800    0    4.560870    4.547171    5.636660 L
H-HC-0.079700    0    2.850090    2.294179    4.846718 L
H-HC-0.079700    0    2.909818    3.646948    3.709950 L
H-H-0.419600     0    1.031531    4.239591    7.215601 L
H-H-0.419600     0    1.707339    2.658480    6.783952 L
N-N--0.415700    0    6.401881    3.071143    6.563747 L
C-CT--0.149000  -1    7.308260    2.330879    7.420264 L
H-H-0.271900     0    6.711839    3.944605    6.160702 L
H-H1-0.097600    0    8.063187    2.996628    7.837954 L
H-H1-0.097600    0    7.794507    1.543302    6.841436 L
H-H1-0.097600    0    6.749419    1.857850    8.230844 L
C-CT--0.366200  -1   -2.234201   -2.744758    9.542300 L
C-C-0.597200     0   -2.341535   -3.844955    8.523169 L
O-O--0.567900    0   -2.864263   -4.914887    8.824520 L
H-HC-0.112300    0   -1.184313   -2.500984    9.693307 L
H-HC-0.112300    0   -2.676079   -3.080427   10.477941 L
H-HC-0.112300    0   -2.763931   -1.867205    9.176203 L
N-N--0.347900    0   -1.822754   -3.582260    7.327350 L
C-CT--0.240000  -1   -1.730009   -4.544683    6.223448 L
```

```
C-C-0.734100      0   -0.323345   -4.574441    5.635858 L
O-O--0.589400     0    0.500000   -3.704696    5.904802 L
C-CT--0.009400    0   -2.840372   -4.246841    5.198087 L
C-CT-0.018700     0   -2.604170   -2.974094    4.377252 L H-HC 37
C-CT--0.464524    0   -3.753043   -2.750444    3.386902 H
C-CT--0.195159    0   -3.518722   -1.625417    2.385856 H
N-N3--0.748751    0   -2.363865   -1.950620    1.474613 H
H-H-0.274700      0   -1.442356   -2.648785    7.183522 L
H-H1-0.142600     0   -1.913583   -5.541538    6.624309 L
H-HC-0.036200     0   -3.793594   -4.160867    5.722779 L
H-HC-0.036200     0   -2.923830   -5.083542    4.511685 L
H-HC-0.010300     0   -1.664257   -3.067811    3.833281 L
H-HC-0.010300     0   -2.548624   -2.120239    5.042705 L
H-HC-0.173903     0   -4.663512   -2.535737    3.952677 H
H-HC-0.148535     0   -3.930130   -3.687790    2.836514 H
H-HP-0.236066     0   -3.314131   -0.646528    2.827015 H
H-HP-0.172509     0   -4.391370   -1.539882    1.727085 H
H-H-0.437293      0   -2.508169   -1.498922    0.536202 H
H-H-0.366736      0   -2.362385   -2.964676    1.296840 H
H-H-0.419451      0   -1.425444   -1.668818    1.839984 H
N-N--0.415700     0   -0.029956   -5.602778    4.853655 L
C-CT--0.025200   -1    1.290338   -5.811125    4.267916 L
C-C-0.597300      0    1.164602   -6.561776    2.939300 L
O-O--0.567900     0    1.699070   -7.652827    2.751752 L
H-H-0.271900      0   -0.776579   -6.247916    4.615010 L
H-H1-0.069800     0    1.908853   -6.395860    4.948837 L
H-H1-0.069800     0    1.779390   -4.854948    4.077832 L
N-N--0.415700     0    0.395179   -5.960365    2.037112 L
C-CT--0.025200   -1   -0.222698   -6.546315    0.848898 L
C-C-0.597300      0   -1.307022   -5.577887    0.416216 L
O-O--0.567900     0   -2.089060   -5.127355    1.250972 L
H-H-0.271900      0   -0.001497   -5.081390    2.343925 L
H-H1-0.069800     0   -0.669891   -7.508844    1.096527 L
H-H1-0.069800     0    0.524284   -6.664702    0.063676 L
N-N--0.415700     0   -1.334428   -5.221804   -0.864829 L
C-CT--0.001400   -1   -2.152501   -4.116146   -1.363645 L
C-C-0.597300      0   -3.628168   -4.202602   -0.952231 L
O-O--0.567900     0   -4.231104   -3.230425   -0.514420 L
C-CT--0.015200    0   -2.031793   -4.026878   -2.897470 L
C-CA--0.001100    0   -0.643695   -4.259738   -3.468143 L
C-CA--0.190600    0    0.383597   -3.324138   -3.240705 L
C-CA--0.190600    0   -0.384216   -5.415682   -4.232138 L
C-CA--0.234100    0    1.668696   -3.545703   -3.772153 L
C-CA--0.234100    0    0.899331   -5.639402   -4.765857 L
C-C-0.322600      0    1.929941   -4.701660   -4.535110 L
O-OH--0.557900    0    3.164283   -4.916062   -5.063818 L
H-H-0.271900      0   -0.581923   -5.529000   -1.464073 L
H-H1-0.087600     0   -1.745543   -3.192408   -0.952637 L
H-HC-0.029500     0   -2.359141   -3.037282   -3.211835 L
H-HC-0.029500     0   -2.715995   -4.745608   -3.349652 L
H-HA-0.169900     0    0.191765   -2.426304   -2.660954 L
H-HA-0.169900     0   -1.178856   -6.120686   -4.427486 L
H-HA-0.165600     0    2.455840   -2.829153   -3.594696 L
H-HA-0.165600     0    1.090415   -6.517531   -5.360339 L
```

H-HO-0.399200	0	3.172049	-5.704643	-5.603197	L
N-N--0.415700	0	-4.206199	-5.397473	-1.068944	L
C-CT--0.024900	-1	-5.551194	-5.660302	-0.575538	L
C-C-0.597300	0	-5.670691	-5.561615	0.942346	L
O-O--0.567900	0	-4.994776	-6.281302	1.687548	L
C-CT-0.211700	0	-6.013401	-7.057333	-0.992255	L
O-OH--0.654600	-1	-7.270144	-7.361569	-0.409598	L
H-H-0.271900	0	-3.621570	-6.149663	-1.385880	L
H-H1-0.084300	0	-6.230145	-4.936248	-1.026677	L
H-H1-0.035200	0	-5.281365	-7.792758	-0.656482	L
H-H1-0.035200	0	-6.088798	-7.104498	-2.078841	L
H-HO-0.427500	-1	-7.527415	-8.234594	-0.716083	L
N-N--0.415700	0	-6.658494	-4.778000	1.368630	L
C-CT-0.033700	-1	-7.145921	-4.682814	2.733364	L
C-C-0.597300	0	-7.459236	-6.020002	3.410374	L
O-O--0.567900	0	-7.309221	-6.151474	4.623004	L
C-CT--0.182500	0	-8.364882	-3.760094	2.737699	L
H-H-0.271900	0	-7.131683	-4.211084	0.671079	L
H-H1-0.082300	0	-6.367536	-4.207068	3.330857	L
H-HC-0.060300	0	-8.105348	-2.788539	2.312751	L
H-HC-0.060300	0	-8.706948	-3.621654	3.758704	L
H-HC-0.060300	0	-9.170044	-4.210130	2.162886	L
N-N--0.415700	0	-7.906823	-7.008907	2.635476	L
C-CT--0.025200	-1	-8.268303	-8.328643	3.140157	L
C-C-0.597300	0	-7.314801	-9.441397	2.719446	L
O-O--0.567900	0	-7.741627	-10.578821	2.533361	L
H-H-0.271900	0	-7.947002	-6.824370	1.639704	L
H-H1-0.069800	0	-9.260640	-8.581359	2.767783	L
H-H1-0.069800	0	-8.317395	-8.324332	4.229348	L
N-N--0.415700	0	-6.021375	-9.136775	2.621073	L
C-CT--0.024900	-1	-4.951259	-10.135615	2.615803	L
C-C-0.597300	0	-4.236767	-10.098286	3.961224	L
O-O--0.567900	0	-3.917359	-9.021817	4.475391	L
C-CT-0.211700	0	-3.972531	-9.851449	1.472188	L
O-OH--0.654600	-1	-3.073407	-10.935972	1.296666	L
H-H-0.271900	0	-5.765570	-8.166489	2.774379	L
H-H1-0.084300	0	-5.370533	-11.129661	2.460094	L
H-H1-0.035200	0	-3.417343	-8.934733	1.678326	L
H-H1-0.035200	0	-4.539657	-9.716922	0.550296	L
H-HO-0.427500	-1	-2.533792	-10.747886	0.525245	L
N-N--0.415700	0	-4.033746	-11.274780	4.553612	L
C-CT--0.149000	-1	-3.510433	-11.417874	5.901596	L
H-H-0.271900	0	-4.308381	-12.095302	4.037411	L
H-H1-0.097600	0	-3.271486	-12.461363	6.106810	L
H-H1-0.097600	0	-4.248959	-11.067264	6.625904	L
H-H1-0.097600	0	-2.605997	-10.815433	6.016031	L
C-CT--0.366200	-1	-12.140442	5.066045	-0.643762	L
C-C-0.597200	0	-10.714095	5.257937	-0.199689	L
O-O--0.567900	0	-10.404038	5.010751	0.960442	L
H-HC-0.112300	0	-12.532659	6.011809	-1.010984	L
H-HC-0.112300	0	-12.730384	4.728798	0.206812	L
H-HC-0.112300	0	-12.169066	4.311094	-1.427352	L
N-N--0.415700	0	-9.862379	5.688129	-1.125922	L
C-CT--0.025200	-1	-8.429558	5.859401	-0.901033	L

C-C-0.597300	0	-7.610569	5.689122	-2.157101	L	
O-O--0.567900	0	-8.129602	5.942471	-3.251783	L	
H-H-0.271900	0	-10.170261	5.825730	-2.076304	L	
H-H1-0.069800	0	-8.118594	5.101315	-0.193209	L	
H-H1-0.069800	0	-8.227502	6.842903	-0.479330	L	
N-N--0.415700	0	-6.343168	5.298514	-1.978273	L	H-H1 48
C-CT--0.530209	-1	-5.347094	5.285182	-3.048790	H	
C-C-0.568093	0	-4.445865	4.066332	-2.932583	H	
O-O--0.528105	0	-4.129941	3.577972	-1.829806	H	
C-CT-0.298500	0	-4.521604	6.596439	-3.056015	L	H-H1 48
C-CT--0.319200	0	-5.383078	7.795667	-3.458743	L	
C-CT--0.319200	0	-3.845334	6.893973	-1.713709	L	
H-H-0.271900	0	-6.021691	5.057271	-1.042461	L	
H-H1-0.184096	0	-5.887665	5.245397	-4.000195	H	
H-HC--0.029700	0	-3.739749	6.501530	-3.808692	L	
H-HC-0.079100	0	-6.120667	8.002621	-2.685482	L	
H-HC-0.079100	0	-5.892834	7.578222	-4.396112	L	
H-HC-0.079100	0	-4.745956	8.669203	-3.589281	L	
H-HC-0.079100	0	-4.587497	7.029296	-0.929025	L	
H-HC-0.079100	0	-3.234570	7.791384	-1.795865	L	
H-HC-0.079100	0	-3.195948	6.062132	-1.435534	L	
N-N--0.610103	0	-3.966003	3.555520	-4.076110	H	
C-CT--0.314060	-1	-4.236666	3.992083	-5.425780	H	
C-C-0.597300	0	-5.353239	3.167525	-6.056358	L	H-H1 50
O-O--0.567900	0	-5.485894	1.964102	-5.834998	L	
C-CT--0.110200	0	-2.949001	3.907946	-6.282998	L	H-H1 50
C-CT-0.353100	0	-1.936065	5.055681	-6.056422	L	
C-CT--0.412100	0	-1.091080	4.928146	-4.788501	L	
C-CT--0.412100	0	-0.960114	5.116978	-7.236257	L	
H-H-0.398207	0	-3.371891	2.731074	-3.986871	H	
H-H1-0.165130	0	-4.553182	5.034833	-5.430586	H	
H-HC-0.045700	0	-3.254191	3.945442	-7.328799	L	
H-HC-0.045700	0	-2.457703	2.948100	-6.128092	L	
H-HC--0.036100	0	-2.476860	6.002276	-6.025987	L	
H-HC-0.100000	0	-0.528783	3.996273	-4.803759	L	
H-HC-0.100000	0	-1.734593	4.924111	-3.908792	L	
H-HC-0.100000	0	-0.405494	5.769314	-4.701710	L	
H-HC-0.100000	0	-0.399556	4.184815	-7.296390	L	
H-HC-0.100000	0	-0.265721	5.944307	-7.091322	L	
H-HC-0.100000	0	-1.508898	5.274854	-8.162828	L	
N-N--0.415700	0	-6.140294	3.816415	-6.910342	L	
C-CT--0.038900	-1	-7.329833	3.220287	-7.530614	L	
C-C-0.597300	0	-7.084249	1.883697	-8.220040	L	
O-O--0.567900	0	-7.966050	1.028133	-8.263874	L	
C-CT-0.365400	-1	-7.968879	4.143969	-8.591934	L	
O-OH--0.676100	-1	-7.907539	5.501356	-8.144506	L	
C-CT--0.243800	0	-9.447574	3.853703	-8.849194	L	
H-H-0.271900	0	-6.013171	4.813705	-6.997991	L	
H-H1-0.100700	0	-8.068993	3.079233	-6.748953	L	
H-H1-0.004300	0	-7.414718	4.067795	-9.528468	L	
H-HC-0.064200	0	-9.563461	2.849903	-9.257478	L	
H-HC-0.064200	0	-9.842719	4.567650	-9.571615	L	
H-HC-0.064200	0	-10.009918	3.924558	-7.917338	L	
H-HO-0.410200	-1	-8.805908	5.836012	-8.108137	L	

N-N--0.415700	0	-5.907997	1.747851	-8.829129	L	
C-CT--0.025200	-1	-5.523441	0.591468	-9.625329	L	
C-C-0.597300	0	-4.639370	-0.421458	-8.927641	L	
O-O--0.567900	0	-4.163741	-1.339229	-9.597004	L	
H-H-0.271900	0	-5.220624	2.461266	-8.637891	L	
H-H1-0.069800	0	-4.984417	0.938397	-10.505671	L	
H-H1-0.069800	0	-6.413030	0.064504	-9.970207	L	
N-N--0.415700	0	-4.409088	-0.235382	-7.622942	L	H-H1 54
C-CT--0.539097	-1	-3.476043	-1.017391	-6.823040	H	
C-C-0.572106	0	-4.217472	-1.459712	-5.563007	H	
O-O--0.533001	0	-4.982538	-2.416952	-5.656560	H	
C-CT-0.130300	0	-2.119133	-0.284640	-6.652811	L	H-H1 54
C-CT--0.043000	0	-1.510069	0.166844	-8.002296	L	
C-CT--0.320400	0	-1.164928	-1.252695	-5.931830	L	
C-CT--0.066000	0	-0.189278	0.938574	-7.880791	L	
H-H-0.271900	0	-4.862881	0.542852	-7.152760	L	
H-H1-0.180646	0	-3.275954	-1.942679	-7.374070	H	
H-HC-0.018700	0	-2.272071	0.599337	-6.038951	L	
H-HC-0.023600	0	-2.210808	0.835377	-8.500545	L	
H-HC-0.023600	0	-1.356969	-0.703970	-8.640902	L	
H-HC-0.088200	0	-1.011812	-2.148757	-6.531459	L	
H-HC-0.088200	0	-1.572516	-1.536507	-4.963592	L	
H-HC-0.088200	0	-0.207055	-0.777551	-5.739079	L	
H-HC-0.018600	0	-0.310989	1.767951	-7.184828	L	
H-HC-0.018600	0	0.086779	1.334171	-8.858211	L	
H-HC-0.018600	0	0.609646	0.283164	-7.535697	L	
N-N--0.611145	0	-4.048402	-0.723787	-4.431738	H	
C-CT--0.109296	-1	-4.636342	-1.118521	-3.159665	H	
C-C-0.597300	0	-6.162210	-1.171247	-3.207408	L	H-H1 56
O-O--0.567900	0	-6.790018	-2.121721	-2.724153	L	
C-CT--0.388038	0	-4.200772	-0.243311	-1.988424	H	
H-H-0.369964	0	-3.319460	-0.022263	-4.378101	H	
H-H1-0.178541	0	-4.351965	-2.150562	-2.928506	H	
H-H1-0.157964	0	-4.858282	-0.520534	-1.158765	H	
H-H1-0.179833	0	-4.371477	0.815189	-2.202627	H	
H-HS-0.319500	0	-2.377872	0.485605	-0.286897	H	
N-N--0.415700	0	-6.754736	-0.159785	-3.829029	L	
C-CT--0.087500	-1	-8.194659	-0.080969	-4.052083	L	
C-C-0.597300	0	-8.695682	-1.220832	-4.927245	L	
O-O--0.567900	0	-9.651858	-1.904768	-4.561351	L	
C-CT-0.298500	0	-8.588122	1.289180	-4.620993	L	
C-CT--0.319200	0	-10.105625	1.430548	-4.802629	L	
C-CT--0.319200	0	-8.126105	2.447776	-3.724205	L	
H-H-0.271900	0	-6.119046	0.533908	-4.216409	L	
H-H1-0.096900	0	-8.687653	-0.187423	-3.088475	L	
H-HC--0.029700	0	-8.117043	1.383718	-5.590936	L	
H-HC-0.079100	0	-10.608947	1.274097	-3.848276	L	
H-HC-0.079100	0	-10.466769	0.700066	-5.526291	L	
H-HC-0.079100	0	-10.339418	2.427017	-5.176458	L	
H-HC-0.079100	0	-8.572083	2.368092	-2.734342	L	
H-HC-0.079100	0	-8.405421	3.401320	-4.170700	L	
H-HC-0.079100	0	-7.040394	2.443386	-3.616495	L	
N-N--0.415700	0	-8.052267	-1.440549	-6.072411	L	
C-CT--0.025200	-1	-8.406649	-2.499246	-7.013575	L	

C-C-0.597300	0	-8.319222	-3.899756	-6.420654	L
O-O--0.567900	0	-9.217767	-4.715877	-6.615193	L
H-H-0.271900	0	-7.240633	-0.865714	-6.261546	L
H-H1-0.069800	0	-7.735192	-2.449292	-7.870509	L
H-H1-0.069800	0	-9.425811	-2.337983	-7.364280	L
N-N--0.415700	0	-7.236609	-4.174432	-5.695818	L
C-CT--0.025200	-1	-7.008581	-5.440231	-5.009959	L
C-C-0.597300	0	-7.937164	-5.705129	-3.835957	L
O-O--0.567900	0	-8.265990	-6.857967	-3.561615	L
H-H-0.271900	0	-6.525423	-3.448010	-5.626594	L
H-H1-0.069800	0	-5.984376	-5.455855	-4.639035	L
H-H1-0.069800	0	-7.126418	-6.255945	-5.723380	L
N-N--0.415700	0	-8.327726	-4.648438	-3.127882	L
C-CT--0.038900	-1	-9.376012	-4.702671	-2.106978	L
C-C-0.597300	0	-10.748226	-4.993061	-2.705528	L
O-O--0.567900	0	-11.491337	-5.826712	-2.187200	L
C-CT-0.365400	0	-9.407428	-3.397422	-1.297804	L
O-OH--0.676100	0	-8.164273	-3.225299	-0.656106	L
C-CT--0.243800	0	-10.484481	-3.408868	-0.213573	L
H-H-0.271900	0	-7.935678	-3.745599	-3.375157	L
H-H1-0.100700	0	-9.148167	-5.516303	-1.418568	L
H-H1-0.004300	0	-9.582215	-2.549374	-1.960196	L
H-HC-0.064200	0	-11.467605	-3.291791	-0.668223	L
H-HC-0.064200	0	-10.318075	-2.585598	0.480538	L
H-HC-0.064200	0	-10.454119	-4.352934	0.328265	L
H-HO-0.410200	0	-7.576688	-2.784759	-1.302867	L
N-N--0.415700	0	-11.099278	-4.287170	-3.781840	L
C-CT--0.149000	-1	-12.331414	-4.483275	-4.517756	L
H-H-0.271900	0	-10.434390	-3.603310	-4.132877	L
H-H1-0.097600	0	-12.410188	-3.730304	-5.302932	L
H-H1-0.097600	0	-13.183637	-4.401273	-3.841808	L
H-H1-0.097600	0	-12.325867	-5.475655	-4.971658	L
N-N2--0.511415	0	1.931669	0.070874	-1.697308	H
C-CA-0.057801	0	0.768648	0.739407	-1.668797	H
C-CA-0.063496	0	0.200458	1.285562	-2.825657	H
C-CA--0.109122	0	0.880701	1.155609	-4.036246	H
C-CA--0.164490	0	2.100770	0.496445	-4.054633	H
C-CA-0.098392	0	2.583078	-0.039866	-2.867837	H
C-C-0.541263	0	-1.156071	1.898170	-2.866561	H
O-O--0.574884	0	-1.844490	1.774697	-3.880583	H
H-H4-0.220889	0	0.267557	0.827502	-0.708611	H
H-HA-0.160434	0	0.439187	1.562846	-4.938083	H
H-HA-0.142191	0	2.670159	0.392411	-4.969412	H
H-H4-0.174502	0	3.511444	-0.599681	-2.838013	H
H-H-0.353801	0	-2.450585	2.886530	-1.830047	H
Zn-ZN-0.974537	0	2.817029	-1.055780	-0.010349	H
O-OW--0.834000	0	-2.159989	-7.215046	3.451881	L
H-HW-0.417000	0	-2.662445	-6.598878	2.899801	L
H-HW-0.417000	0	-2.842143	-7.791688	3.834134	L
O-OW--0.783903	0	1.321112	-2.544029	-0.145562	H
H-HW-0.401253	0	0.927685	-2.422449	0.757365	H
H-HW-0.424111	0	1.926855	-3.327564	-0.178771	H
O-OW--0.834000	0	-3.802961	-8.470074	7.242135	L
H-HW-0.417000	0	-3.815601	-7.525060	7.414990	L

```
H-HW-0.417000    0    -3.866866   -8.532902    6.277471 L
O-OW--0.834000   0     7.150419   -0.010100    1.772723 L
H-HW-0.417000    0     6.848811    0.406384    2.600816 L
H-HW-0.417000    0     6.357836    0.075161    1.218977 L
O-OW--0.834000   0     7.041937   -4.951895    4.737730 L
H-HW-0.417000    0     6.092149   -5.000335    4.970004 L
H-HW-0.417000    0     7.232102   -5.829852    4.405340 L
O-OW--0.834000   0     8.647501   -0.915887   -0.252023 L
H-HW-0.417000    0     8.156910   -0.545768    0.510409 L
H-HW-0.417000    0     8.848641   -0.126513   -0.775126 L
O-OW--0.834000   0    12.656862   -1.073583   -2.012462 L
H-HW-0.417000    0    11.798296   -0.823677   -2.395288 L
H-HW-0.417000    0    12.658934   -2.037233   -2.118806 L
O-OW--0.834000   0     8.307280   -3.286308    0.986463 L
H-HW-0.417000    0     8.519210   -2.515482    0.419394 L
H-HW-0.417000    0     7.574561   -2.951241    1.513659 L
O-OW--0.834000   0     4.351492   -4.880643    5.143516 L
H-HW-0.417000    0     4.088683   -4.507058    6.007450 L
H-HW-0.417000    0     4.054415   -4.200374    4.527850 L
S-SH--0.326142   0    -2.461620   -0.526087   -1.457262 H
O-OW--0.834000   0    -1.492129    2.596162   -1.800287 H
```

PC$_b$

```
C-CT--0.3662    -1  -10.314404    3.908782    4.022547 L
C-C-0.5972       0    -8.983992    3.258714    3.777156 L
O-O--0.5679      0    -8.409130    2.685477    4.695695 L
H-HC-0.1123      0   -11.056943    3.448919    3.373447 L
H-HC-0.1123      0   -10.235308    4.970974    3.797574 L
H-HC-0.1123      0   -10.593383    3.768736    5.064438 L
N-N--0.415700    0    -8.521259    3.351551    2.537621 L
C-CT--0.059700  -1    -7.347460    2.641875    2.022276 L
C-C-0.597300     0    -6.201878    3.624877    1.840792 L
O-O--0.567900    0    -6.366102    4.635909    1.164093 L
C-CT-0.130300    0    -7.696623    1.900481    0.707623 L
C-CT--0.043000   0    -8.898156    0.940372    0.904593 L
C-CT--0.320400   0    -6.478442    1.108011    0.204028 L
C-CT--0.066000   0    -9.415284    0.301131   -0.388999 L
H-H-0.271900     0    -9.055042    3.918476    1.887086 L
H-H1-0.086900    0    -7.036414    1.891734    2.749254 L
H-HC-0.018700    0    -7.963420    2.641449   -0.048214 L
H-HC-0.023600    0    -9.737763    1.486288    1.333053 L
H-HC-0.023600    0    -8.621349    0.149795    1.603258 L
H-HC-0.088200    0    -6.204172    0.328206    0.914698 L
H-HC-0.088200    0    -5.623486    1.769624    0.057537 L
H-HC-0.088200    0    -6.693831    0.662725   -0.765232 L
H-HC-0.018600    0    -9.634728    1.075017   -1.123364 L
H-HC-0.018600    0   -10.331808   -0.248043   -0.176374 L
H-HC-0.018600    0    -8.678817   -0.393663   -0.789654 L
N-N--0.516300    0    -5.082633    3.333980    2.499206 L
C-CT-0.038100   -1    -3.781436    3.977237    2.383375 L H-HC      30
C-C-0.536600     0    -3.710131    5.502225    2.167944 L
O-O--0.581900    0    -2.722608    6.038106    1.665980 L
C-CT--0.547145   0    -3.006864    3.225842    1.273849 H
C-C-0.532653     0    -2.420997    1.890147    1.681079 H
```

O-O2--0.588507	0	-2.571917	1.432468	2.823406	H
O-O2--0.625559	0	-1.726058	1.266354	0.761861	H
H-H-0.293600	0	-5.054716	2.445742	2.982045	L
H-H1-0.088000	0	-3.246582	3.807371	3.318083	L
H-HC-0.128435	0	-2.144840	3.832070	0.954947	H
H-HC-0.183556	0	-3.619735	3.139675	0.369367	H
N-N--0.415700	0	-4.690855	6.252709	2.647435	L
C-CT--0.025200	-1	-4.658975	7.695325	2.508444	L
C-C-0.597300	0	-3.763624	8.339707	3.552686	L
O-O--0.567900	0	-4.241536	9.002235	4.475124	L
H-H-0.271900	0	-5.528725	5.757038	2.909033	L
H-H1-0.069800	0	-5.668158	8.085905	2.629677	L
H-H1-0.069800	0	-4.305198	7.970302	1.513332	L
N-N--0.415700	0	-2.459716	8.129662	3.406005	L
C-CT--0.038900	-1	-1.435299	8.558179	4.346835	L
C-C-0.597300	0	-0.524790	9.637949	3.750532	L
O-O--0.567900	0	-0.621262	10.004380	2.578168	L
C-CT-0.365400	0	-0.592866	7.403184	4.932462	L
O-OH--0.676100	0	0.598220	7.230530	4.206415	L
C-CT--0.243800	0	-1.307748	6.060959	5.047701	L
H-H-0.271900	0	-2.196717	7.534277	2.622759	L
H-H1-0.100700	0	-1.939418	9.020098	5.193758	L
H-H1-0.004300	0	-0.302740	7.700703	5.940703	L
H-HC-0.064200	0	-2.249255	6.190206	5.579523	L
H-HC-0.064200	0	-0.675967	5.367907	5.598012	L
H-HC-0.064200	0	-1.499740	5.646239	4.058676	L
H-HO-0.410200	0	1.089303	6.473978	4.593958	L
N-N--0.415700	0	0.391286	10.151190	4.575684	L
C-CT--0.025200	-1	1.431340	11.086375	4.160166	L
C-C-0.597300	0	2.332739	10.598125	3.035187	L
O-O--0.567900	0	2.668717	11.375550	2.146815	L
H-H-0.271900	0	0.452465	9.749010	5.495667	L
H-H1-0.069800	0	2.066322	11.320667	5.013391	L
H-H1-0.069800	0	0.958603	12.009903	3.825280	L
N-N--0.516300	0	2.728131	9.328792	3.090001	L
C-CT-0.038100	-1	3.636982	8.731299	2.112714	L
C-C-0.536600	0	3.006096	8.439068	0.764560	L
O-O--0.581900	0	3.738719	8.141514	-0.172617	L
C-CT--0.030300	0	4.221513	7.418016	2.664271	L
C-C-0.799400	-1	5.568820	7.619149	3.339396	L
O-O2--0.801400	-1	6.148604	8.723573	3.257300	L
O-O2--0.801400	-1	6.029527	6.665977	4.000027	L
H-H-0.293600	0	2.352885	8.722003	3.805095	L
H-H1-0.088000	0	4.439174	9.438163	1.898081	L
H-HC--0.012200	0	4.376740	6.702155	1.855319	L
H-HC--0.012200	0	3.519356	6.962273	3.365596	L
N-N--0.415700	0	1.684076	8.525067	0.651740	L
C-CT--0.002400	-1	0.965393	8.319742	-0.598671	L
C-C-0.597300	0	0.368630	9.612966	-1.155657	L
O-O--0.567900	0	0.353629	9.824532	-2.368321	L
C-CT--0.034300	0	-0.098707	7.228445	-0.422380	L
C-CA-0.011800	0	0.428314	5.901245	0.099925	L
C-CA--0.125600	0	1.057800	4.982703	-0.760325	L
C-CA--0.125600	0	0.252004	5.566298	1.452661	L

```
C-CA--0.170400  0      1.490217    3.738677   -0.263473 L
C-CA--0.170400  0      0.660972    4.317434    1.946628 L
C-CA--0.107200  0      1.274878    3.399253    1.082186 L
H-H-0.271900    0      1.163941    8.724306    1.493523 L
H-H1-0.097800   0      1.655247    7.953938   -1.358399 L
H-HC-0.029500   0     -0.570559    7.047873   -1.389001 L
H-HC-0.029500   0     -0.873324    7.595066    0.252876 L
H-HA-0.133000   0      1.173277    5.216183   -1.807735 L
H-HA-0.133000   0     -0.256601    6.258608    2.098535 L
H-HA-0.143000   0      1.947622    3.017697   -0.920980 L
H-HA-0.143000   0      0.462367    4.051257    2.973582 L
H-HA-0.129700   0      1.547389    2.420495    1.442306 L
N-N--0.4157     0     -0.087623   10.507743   -0.276564 L
C-CT--0.1490   -1     -0.484574   11.853155   -0.640451 L
H-H-0.2719      0     -0.083605   10.246018    0.704035 L
H-H1-0.0976     0     -0.872723   12.368940    0.238280 L
H-H1-0.0976     0     -1.256644   11.810743   -1.410551 L
H-H1-0.0976     0      0.379774   12.394286   -1.028228 L
C-CT--0.3662   -1      7.975745    8.437787   -0.169819 L
C-C-0.5972      0      7.514306    7.640000   -1.366707 L
O-O--0.5679     0      8.336335    7.289723   -2.209992 L
H-HC-0.1123     0      7.530846    9.429816   -0.202818 L
H-HC-0.1123     0      9.060493    8.513253   -0.185603 L
H-HC-0.1123     0      7.664194    7.930602    0.744234 L
N-N--0.415700   0      6.204294    7.395511   -1.463722 L
C-CT--0.051800 -1      5.553032    6.860517   -2.667337 L
C-C-0.597300    0      4.902544    7.979150   -3.481649 L
O-O--0.567900   0      5.272158    8.236694   -4.627607 L
C-CT--0.110200  0      4.618280    5.677752   -2.329332 L
C-CT-0.353100   0      4.651124    4.581290   -3.415092 L
C-CT--0.412100  0      3.834114    3.366403   -2.992512 L
C-CT--0.412100  0      4.098447    5.033024   -4.769239 L
H-H-0.271900    0      5.599364    7.717481   -0.715346 L
H-H1-0.092200   0      6.342860    6.462892   -3.305815 L
H-HC-0.045700   0      3.596653    6.027791   -2.178307 L
H-HC-0.045700   0      4.953460    5.225229   -1.395806 L
H-HC--0.036100  0      5.682779    4.253810   -3.552157 L
H-HC-0.100000   0      2.772557    3.612855   -2.983732 L
H-HC-0.100000   0      4.134877    3.039841   -1.998755 L
H-HC-0.100000   0      4.006272    2.549942   -3.690836 L
H-HC-0.100000   0      3.103324    5.460467   -4.640021 L
H-HC-0.100000   0      4.037892    4.183625   -5.448561 L
H-HC-0.100000   0      4.757274    5.776896   -5.212808 L
N-N--0.4157     0      3.966311    8.670836   -2.845874 L
C-CT--0.1490   -1      3.344063    9.889837   -3.293555 L
H-H-0.2719      0      3.775230    8.362377   -1.899882 L
H-H1-0.0976     0      4.055276   10.484476   -3.868229 L
H-H1-0.0976     0      3.003013   10.465657   -2.431950 L
H-H1-0.0976     0      2.484624    9.650881   -3.922231 L
C-CT--0.3662   -1     -8.724860    2.365316    8.129466 L
C-C-0.5972      0     -7.850212    1.157049    7.950234 L
O-O--0.5679     0     -7.529272    0.476627    8.918070 L
H-HC-0.1123     0     -9.642589    2.224086    7.561834 L
H-HC-0.1123     0     -8.195867    3.242984    7.761091 L
```

H-HC-0.1123	0	-8.954060	2.486013	9.186076	L		
N-N--0.415700	0	-7.459609	0.905780	6.708875	L		
C-CT--0.038900	-1	-6.663081	-0.251206	6.296140	L		
C-C-0.597300	0	-5.392255	0.223156	5.614185	L		
O-O--0.567900	0	-5.419457	0.624606	4.451313	L		
C-CT-0.365400	0	-7.474072	-1.216047	5.417281	L		
O-OH--0.676100	0	-8.029625	-0.551983	4.308225	L		
C-CT--0.243800	0	-8.623670	-1.835445	6.217512	L		
H-H-0.271900	0	-7.790507	1.525729	5.973177	L		
H-H1-0.100700	0	-6.365319	-0.815131	7.179772	L		
H-H1-0.004300	0	-6.817884	-2.013033	5.065265	L		
H-HC-0.064200	0	-8.226081	-2.368666	7.080662	L		
H-HC-0.064200	0	-9.178981	-2.532719	5.596181	L		
H-HC-0.064200	0	-9.309046	-1.058492	6.554523	L		
H-HO-0.410200	0	-7.333338	0.020469	3.954888	L		
N-N--0.415700	0	-4.306986	0.234148	6.392541	L		
C-CT--0.059700	-1	-3.110605	1.045808	6.158323	L		
C-C-0.597300	0	-1.862185	0.170643	6.087969	L		
O-O--0.567900	0	-1.601486	-0.628294	6.992250	L		
C-CT-0.130300	0	-2.982897	2.141237	7.242336	L		
C-CT--0.043000	0	-4.242696	3.029844	7.396284	L		
C-CT--0.320400	0	-1.751235	3.019031	6.974623	L		
C-CT--0.066000	0	-4.688418	3.789441	6.137675	L		
H-H-0.271900	0	-4.384081	-0.177521	7.308274	L		
H-H1-0.086900	0	-3.214817	1.548499	5.196681	L		
H-HC-0.018700	0	-2.826266	1.646746	8.202874	L		
H-HC-0.023600	0	-4.055792	3.760851	8.183696	L		
H-HC-0.023600	0	-5.074397	2.412745	7.735409	L		
H-HC-0.088200	0	-1.762854	3.362692	5.939972	L		
H-HC-0.088200	0	-0.843108	2.437141	7.134073	L		
H-HC-0.088200	0	-1.736535	3.874104	7.649160	L		
H-HC-0.018600	0	-3.895594	4.448628	5.788267	L		
H-HC-0.018600	0	-5.566321	4.391353	6.372670	L		
H-HC-0.018600	0	-4.952068	3.092359	5.345370	L		
N-N--0.516300	0	-1.114793	0.344961	5.001859	L		
C-CT-0.038100	-1	0.185053	-0.238641	4.720621	L	H-HC	178
C-C-0.536600	0	1.124946	-0.083125	5.921459	L		
O-O--0.581900	0	1.335203	1.008751	6.442800	L		
C-CT--0.520019	0	0.678975	0.410451	3.415405	H		
C-C-0.636404	0	1.000038	-0.557125	2.292703	H		
O-O2--0.608036	0	0.411858	-1.678066	2.215861	H		
O-O2--0.608131	0	1.844742	-0.113594	1.449940	H		
H-H-0.293600	0	-1.505264	0.943460	4.279247	L		
H-H1-0.088000	0	0.030957	-1.306657	4.562244	L		
H-HC-0.132300	0	1.568063	1.009865	3.621250	H		
H-HC-0.188993	0	-0.077939	1.097474	3.021790	H		
N-N--0.415700	0	1.671560	-1.194370	6.382867	L		
C-CT-0.033700	-1	2.571592	-1.240332	7.512853	L		
C-C-0.597300	0	3.795465	-2.057481	7.114865	L		
O-O--0.567900	0	3.852693	-3.281087	7.281500	L		
C-CT--0.182500	0	1.815570	-1.800851	8.725799	L		
H-H-0.271900	0	1.368776	-2.072544	5.976435	L		
H-H1-0.082300	0	2.914369	-0.234887	7.764764	L		
H-HC-0.060300	0	0.973779	-1.148311	8.960914	L		

H-HC-0.060300	0	2.485754	-1.849641	9.583716	L		
H-HC-0.060300	0	1.441781	-2.801191	8.501606	L		
N-N--0.4157	0	4.759429	-1.362926	6.522219	L		
C-CT-0.0188	-1	5.966532	-1.946028	5.981410	L	H-HC	200
C-C-0.5973	0	7.161479	-1.799833	6.933119	L		
O-O--0.5679	0	7.207180	-0.952190	7.827082	L		
C-CT--0.517456	0	6.227477	-1.314896	4.612766	H		
C-CM-0.287351	0	5.359994	-1.802851	3.512422	H		
N-N2--0.586994	0	5.507448	-3.058195	2.938680	H		
C-CM--0.020338	0	4.371729	-1.203655	2.784032	H		
C-CM-0.242458	0	4.633655	-3.157949	1.902145	H		
N-N2--0.505070	0	3.936196	-2.043774	1.779823	H		
H-H-0.2719	0	4.649604	-0.354771	6.454324	L		
H-H1-0.0881	0	5.819343	-3.012358	5.841980	L		
H-HC-0.166355	0	7.283401	-1.480614	4.353386	H		
H-HC-0.174481	0	6.112071	-0.236040	4.742050	H		
H-H-0.335795	0	6.159478	-3.771307	3.258446	H		
H-H4-0.152777	0	3.964829	-0.211680	2.907402	H		
H-H5-0.209475	0	4.536008	-4.026984	1.266546	H		
N-N--0.4157	0	8.166904	-2.637560	6.685491	L		
C-CT--0.1490	-1	9.438800	-2.564364	7.371175	L		
H-H-0.2719	0	8.014745	-3.315074	5.945008	L		
H-H1-0.0976	0	10.111506	-3.332873	6.989334	L		
H-H1-0.0976	0	9.285486	-2.712000	8.441565	L		
H-H1-0.0976	0	9.883394	-1.580356	7.211451	L		
C-CT--0.3662	-1	8.361372	-9.383358	0.911212	L		
C-C-0.5972	0	8.739326	-9.156511	-0.540167	L		
O-O--0.5679	0	9.774172	-8.554866	-0.800493	L		
H-HC-0.1123	0	8.280869	-10.449309	1.110537	L		
H-HC-0.1123	0	7.422206	-8.876893	1.126441	L		
H-HC-0.1123	0	9.140259	-8.953961	1.541216	L		
N-N--0.254800	0	7.918888	-9.612747	-1.503204	L		
C-CT--0.026600	-1	8.159822	-9.374866	-2.928656	L		
C-C-0.589600	0	8.013316	-7.901470	-3.309307	L		
O-O--0.574800	0	8.727326	-7.404639	-4.177225	L		
C-CT--0.007000	0	7.149405	-10.265325	-3.659642	L		
C-CT-0.018900	0	5.990862	-10.388759	-2.668645	L		
C-CT-0.019200	0	6.685445	-10.362711	-1.306269	L		
H-H1-0.064100	0	9.171068	-9.687930	-3.191516	L		
H-HC-0.025300	0	7.590526	-11.249629	-3.823738	L		
H-HC-0.025300	0	6.827767	-9.833418	-4.608641	L		
H-HC-0.021300	0	5.428024	-11.310842	-2.816784	L		
H-HC-0.021300	0	5.333690	-9.522672	-2.761446	L		
H-H1-0.039100	0	6.039876	-9.887306	-0.566412	L		
H-H1-0.039100	0	6.927668	-11.380162	-0.996441	L		
N-N--0.516300	0	7.089064	-7.205974	-2.646343	L		
C-CT-0.039700	-1	6.833478	-5.779554	-2.816254	L		
C-C-0.536600	0	8.091607	-4.912572	-2.687755	L		
O-O--0.581900	0	8.265061	-3.929377	-3.411495	L		
C-CT-0.056000	0	5.736461	-5.349085	-1.838661	L	H-HC	244
C-CT--0.515956	0	5.199522	-3.965973	-2.214173	H		
C-C-0.587828	0	4.113126	-3.438193	-1.287716	H		
O-O2--0.600319	0	4.062400	-2.156370	-1.233411	H		
O-O2--0.589127	0	3.355112	-4.230355	-0.683483	H		

H-H-0.293600	0	6.553924	-7.684240	-1.940887	L
H-H1-0.110500	0	6.455397	-5.625435	-3.827311	L
H-HC--0.017300	0	6.133808	-5.329589	-0.822626	L
H-HC--0.017300	0	4.916541	-6.067768	-1.885237	L
H-HC-0.167759	0	4.787475	-4.017159	-3.229206	H
H-HC-0.135230	0	6.019104	-3.239627	-2.231758	H
N-N--0.415700	0	9.002461	-5.317477	-1.805158	L
C-CT--0.024900	-1	10.242226	-4.617471	-1.490083	L
C-C-0.597300	0	11.263234	-4.506369	-2.615515	L
O-O--0.567900	0	12.244234	-3.774603	-2.481863	L
C-CT-0.211700	0	10.883351	-5.255252	-0.251404	L
O-OH--0.654600	0	9.927274	-5.448871	0.780614	L
H-H-0.271900	0	8.792286	-6.125655	-1.236049	L
H-H1-0.084300	0	9.976320	-3.593622	-1.222526	L
H-H1-0.035200	0	11.681791	-4.607159	0.112888	L
H-H1-0.035200	0	11.310625	-6.222231	-0.521193	L
H-HO-0.427500	0	9.375601	-4.638958	0.862711	L
N-N--0.415700	0	11.022877	-5.189685	-3.732681	L
C-CT-0.033700	-1	11.772354	-5.025379	-4.971469	L
C-C-0.597300	0	10.939942	-4.466876	-6.130227	L
O-O--0.567900	0	11.374319	-4.474807	-7.281264	L
C-CT--0.182500	0	12.398305	-6.386685	-5.307963	L
H-H-0.271900	0	10.208591	-5.793620	-3.740509	L
H-H1-0.082300	0	12.588482	-4.318307	-4.816748	L
H-HC-0.060300	0	13.014726	-6.726093	-4.474819	L
H-HC-0.060300	0	13.024272	-6.294698	-6.196198	L
H-HC-0.060300	0	11.611631	-7.118517	-5.498439	L
N-N--0.415700	0	9.758915	-3.952576	-5.804919	L
C-CT--0.025200	-1	8.855261	-3.269861	-6.707948	L
C-C-0.597300	0	8.806986	-1.766016	-6.487075	L
O-O--0.567900	0	8.826222	-0.992841	-7.441939	L
H-H-0.271900	0	9.479305	-4.011792	-4.831963	L
H-H1-0.069800	0	7.848676	-3.661627	-6.564798	L
H-H1-0.069800	0	9.145079	-3.452466	-7.743188	L
N-N--0.415700	0	8.743943	-1.360907	-5.221856	L
C-CT--0.002400	-1	8.572824	0.024180	-4.800249	L
C-C-0.597300	0	9.690740	0.457925	-3.845169	L
O-O--0.567900	0	10.318049	-0.393889	-3.206731	L
C-CT--0.034300	0	7.187609	0.145515	-4.138999	L
C-CA-0.011800	0	6.038010	-0.103402	-5.098031	L
C-CA--0.125600	0	5.623689	0.912908	-5.980069	L
C-CA--0.125600	0	5.409897	-1.362602	-5.141678	L
C-CA--0.170400	0	4.597899	0.666814	-6.910743	L
C-CA--0.170400	0	4.380495	-1.606556	-6.068146	L
C-CA--0.107200	0	3.979648	-0.595415	-6.958986	L
H-H-0.271900	0	8.744982	-2.068790	-4.496963	L
H-H1-0.097800	0	8.599484	0.675449	-5.674068	L
H-HC-0.029500	0	7.065814	1.139286	-3.708992	L
H-HC-0.029500	0	7.130301	-0.567169	-3.314640	L
H-HA-0.133000	0	6.097950	1.883167	-5.950697	L
H-HA-0.133000	0	5.724645	-2.147413	-4.469557	L
H-HA-0.143000	0	4.280935	1.449683	-7.585140	L
H-HA-0.143000	0	3.893669	-2.570541	-6.093678	L
H-HA-0.129700	0	3.183201	-0.782729	-7.665813	L

N-N--0.254800	0	9.946367	1.771397	-3.699850	L		
C-CT--0.026600	-1	10.810859	2.265136	-2.634333	L		
C-C-0.589600	0	10.178458	2.014908	-1.256575	L		
O-O--0.574800	0	8.985887	1.703425	-1.176915	L		
C-CT--0.007000	0	10.968361	3.765238	-2.916122	L		
C-CT-0.018900	0	9.649221	4.133702	-3.592950	L		
C-CT-0.019200	0	9.338367	2.886451	-4.420201	L		
H-H1-0.064100	0	11.780792	1.772166	-2.691454	L		
H-HC-0.025300	0	11.791217	3.919740	-3.615553	L		
H-HC-0.025300	0	11.128181	4.349032	-2.008291	L		
H-HC-0.021300	0	9.744737	5.023077	-4.216613	L		
H-HC-0.021300	0	8.874385	4.277708	-2.837240	L		
H-H1-0.039100	0	8.259841	2.776881	-4.524199	L		
H-H1-0.039100	0	9.800868	2.973659	-5.404160	L		
N-N--0.254800	0	10.944140	2.132630	-0.161494	L		
C-CT--0.026600	-1	10.391084	2.119116	1.185800	L		
C-C-0.589600	0	9.340411	3.204362	1.421762	L		
O-O--0.574800	0	9.604040	4.384625	1.203806	L		
C-CT--0.007000	0	11.591257	2.300778	2.126017	L		
C-CT-0.018900	0	12.778351	1.817413	1.296483	L		
C-CT-0.019200	0	12.393733	2.237725	-0.119123	L		
H-H1-0.064100	0	9.949406	1.137763	1.364819	L		
H-HC-0.025300	0	11.475068	1.718310	3.040534	L		
H-HC-0.025300	0	11.733542	3.356072	2.366091	L		
H-HC-0.021300	0	12.844012	0.729275	1.348455	L		
H-HC-0.021300	0	13.713586	2.277537	1.616301	L		
H-H1-0.039100	0	12.689070	3.273207	-0.293759	L		
H-H1-0.039100	0	12.871010	1.574604	-0.841451	L		
N-N--0.4157	0	8.171356	2.779341	1.890702	L		
C-CT-0.0188	-1	7.013790	3.606538	2.185849	L	H-HC	334
C-C-0.5973	0	6.152111	3.012879	3.297917	L		
O-O--0.5679	0	6.179183	1.804796	3.532152	L		
C-CT--0.515368	0	6.194596	3.723299	0.872799	H		
C-CM-0.287903	0	5.605443	2.441558	0.363329	H		
N-N2--0.582625	0	6.224363	1.661895	-0.600374	H		
C-CM--0.036893	0	4.498609	1.707034	0.720803	H		
C-CM-0.243454	0	5.503254	0.525048	-0.778110	H		
N-N2--0.523998	0	4.451918	0.517604	0.020503	H		
H-H-0.2719	0	8.032327	1.782218	1.969870	L		
H-H1-0.0881	0	7.345954	4.601856	2.496225	L		
H-HC-0.162301	0	6.850397	4.179068	0.116635	H		
H-HC-0.188340	0	5.398207	4.456219	1.051984	H		
H-H-0.339448	0	7.128865	1.871287	-1.022818	H		
H-H4-0.185957	0	3.739911	1.956988	1.446788	H		
H-H5-0.211295	0	5.775464	-0.262838	-1.465035	H		
N-N--0.415700	0	5.371558	3.872617	3.940240	L		
C-CT-0.014300	-1	4.551323	3.591465	5.109363	L		
C-C-0.597300	0	5.204860	2.640682	6.122628	L		
O-O--0.567900	0	4.681221	1.588628	6.493674	L		
C-CT--0.204100	0	3.083681	3.348854	4.736485	L		
C-C-0.713000	0	2.197208	4.191372	5.636887	L		
N-N--0.919100	0	1.703028	3.669110	6.724895	L		
O-O--0.593100	0	1.978447	5.368747	5.412507	L		
H-H-0.271900	0	5.500950	4.859368	3.709879	L		

H-H1-0.104800	0	4.550868	4.549707	5.634279	L		
H-HC-0.079700	0	2.844372	2.295368	4.834859	L		
H-HC-0.079700	0	2.900021	3.657962	3.708181	L		
H-H-0.419600	0	1.068941	4.231297	7.250110	L		
H-H-0.419600	0	1.751646	2.654241	6.813391	L		
N-N--0.4157	0	6.392273	3.072477	6.560837	L		
C-CT--0.1490	-1	7.298513	2.332596	7.417705	L		
H-H-0.2719	0	6.703253	3.945857	6.158365	L		
H-H1-0.0976	0	8.052702	2.998728	7.836183	L		
H-H1-0.0976	0	7.785899	1.545601	6.839028	L		
H-H1-0.0976	0	6.739598	1.858871	8.227828	L		
C-CT--0.3662	-1	-2.244745	-2.743127	9.535945	L		
C-C-0.5972	0	-2.366394	-3.837326	8.511083	L		
O-O--0.5679	0	-2.896293	-4.904268	8.809513	L		
H-HC-0.1123	0	-1.191613	-2.516516	9.690817	L		
H-HC-0.1123	0	-2.693866	-3.076868	10.468891	L		
H-HC-0.1123	0	-2.760618	-1.856037	9.173162	L		
N-N--0.347900	0	-1.847066	-3.574781	7.315237	L		
C-CT--0.240000	-1	-1.739936	-4.541982	6.216605	L		
C-C-0.734100	0	-0.326335	-4.581860	5.647990	L		
O-O--0.589400	0	0.494147	-3.708772	5.915720	L		
C-CT--0.009400	0	-2.820310	-4.245671	5.154994	L		
C-CT-0.018700	0	-2.591745	-2.946952	4.370754	L	H-HC	379
C-CT--0.464524	0	-3.654506	-2.760450	3.281151	H		
C-CT--0.195159	0	-3.544850	-1.433879	2.538816	H		
N-N3--0.748751	0	-2.304848	-1.326132	1.698564	H		
H-H-0.274700	0	-1.461009	-2.643393	7.175015	L		
H-H1-0.142600	0	-1.936443	-5.537432	6.615278	L		
H-HC-0.036200	0	-3.793830	-4.196970	5.645798	L		
H-HC-0.036200	0	-2.857404	-5.065544	4.444826	L		
H-HC-0.010300	0	-1.605860	-2.963760	3.906286	L		
H-HC-0.010300	0	-2.652870	-2.109423	5.057835	L		
H-HC-0.173903	0	-4.640582	-2.792397	3.754071	H		
H-HC-0.148535	0	-3.605984	-3.604606	2.579720	H		
H-HP-0.236066	0	-3.553555	-0.567892	3.198684	H		
H-HP-0.172509	0	-4.382501	-1.330681	1.838556	H		
H-H-0.437293	0	-2.125474	-0.330533	1.393975	H		
H-H-0.366736	0	-2.429879	-1.871506	0.834277	H		
H-H-0.419451	0	-1.415956	-1.619727	2.157245	H		
N-N--0.415700	0	-0.032105	-5.611288	4.866703	L		
C-CT--0.025200	-1	1.280762	-5.807970	4.261321	L		
C-C-0.597300	0	1.153740	-6.524538	2.916339	L		
O-O--0.567900	0	1.742378	-7.578674	2.684636	L		
H-H-0.271900	0	-0.776915	-6.261853	4.634802	L		
H-H1-0.069800	0	1.904691	-6.408222	4.923585	L		
H-H1-0.069800	0	1.770314	-4.848377	4.091143	L		
N-N--0.415700	0	0.333099	-5.941368	2.046858	L		
C-CT--0.025200	-1	-0.231567	-6.541924	0.841723	L		
C-C-0.597300	0	-1.379710	-5.673928	0.393970	L		
O-O--0.567900	0	-2.279403	-5.440822	1.192964	L		
H-H-0.271900	0	-0.109308	-5.092153	2.372327	L		
H-H1-0.069800	0	-0.603818	-7.541167	1.067470	L		
H-H1-0.069800	0	0.533148	-6.597113	0.066419	L		
N-N--0.4157	0	-1.328260	-5.198048	-0.850478	L		

C-CT--0.0014	-1	-2.160722	-4.110895	-1.370437	L
C-C-0.5973	0	-3.625389	-4.184848	-0.919085	L
O-O--0.5679	0	-4.186276	-3.227795	-0.395072	L
C-CT--0.0152	0	-2.057025	-4.076913	-2.909476	L
C-CA--0.0011	0	-0.667248	-4.290448	-3.485221	L
C-CA--0.1906	0	0.355812	-3.353615	-3.242780	L
C-CA--0.1906	0	-0.401928	-5.431228	-4.269678	L
C-CA--0.2341	0	1.641746	-3.559226	-3.778826	L
C-CA--0.2341	0	0.881603	-5.638363	-4.809650	L
C-C-0.3226	0	1.907410	-4.699590	-4.563682	L
O-OH--0.5579	0	3.141378	-4.898036	-5.099209	L
H-H-0.2719	0	-0.495763	-5.390640	-1.389243	L
H-H1-0.0876	0	-1.742362	-3.185997	-0.979952	L
H-HC-0.0295	0	-2.422316	-3.117020	-3.267826	L
H-HC-0.0295	0	-2.720061	-4.836960	-3.323349	L
H-HA-0.1699	0	0.159334	-2.466201	-2.649695	L
H-HA-0.1699	0	-1.192533	-6.137826	-4.475371	L
H-HA-0.1656	0	2.425225	-2.841295	-3.590862	L
H-HA-0.1656	0	1.075845	-6.505078	-5.419652	L
H-HO-0.3992	0	3.146325	-5.667186	-5.666186	L
N-N--0.415700	0	-4.226640	-5.364185	-1.090686	L
C-CT--0.024900	-1	-5.559689	-5.655093	-0.583596	L
C-C-0.597300	0	-5.687082	-5.581710	0.936265	L
O-O--0.567900	0	-5.069317	-6.355833	1.677080	L
C-CT-0.211700	0	-6.026475	-7.046236	-1.015367	L
O-OH--0.654600	-1	-7.278786	-7.356302	-0.418600	L
H-H-0.271900	0	-3.656645	-6.098711	-1.469225	L
H-H1-0.084300	0	-6.246986	-4.930212	-1.020063	L
H-H1-0.035200	0	-5.290373	-7.785951	-0.698435	L
H-H1-0.035200	0	-6.114759	-7.078455	-2.101357	L
H-HO-0.427500	-1	-7.536045	-8.229209	-0.725433	L
N-N--0.415700	0	-6.655010	-4.774870	1.364952	L
C-CT-0.033700	-1	-7.155084	-4.678601	2.725279	L
C-C-0.597300	0	-7.522854	-6.001086	3.403169	L
O-O--0.567900	0	-7.492196	-6.093279	4.628735	L
C-CT--0.182500	0	-8.337730	-3.709522	2.724805	L
H-H-0.271900	0	-7.100743	-4.182582	0.670668	L
H-H1-0.082300	0	-6.366235	-4.235595	3.332665	L
H-HC-0.060300	0	-8.040082	-2.748464	2.301156	L
H-HC-0.060300	0	-8.679044	-3.558265	3.744311	L
H-HC-0.060300	0	-9.156429	-4.128475	2.145770	L
N-N--0.415700	0	-7.885491	-7.017047	2.619543	L
C-CT--0.025200	-1	-8.277793	-8.324493	3.130613	L
C-C-0.597300	0	-7.309378	-9.459203	2.826738	L
O-O--0.567900	0	-7.707466	-10.622179	2.852831	L
H-H-0.271900	0	-7.845626	-6.857927	1.619059	L
H-H1-0.069800	0	-9.240451	-8.587879	2.693679	L
H-H1-0.069800	0	-8.412478	-8.288381	4.211896	L
N-N--0.415700	0	-6.039631	-9.145479	2.574975	L
C-CT--0.024900	-1	-4.960750	-10.131506	2.606390	L
C-C-0.597300	0	-4.272079	-10.091134	3.964744	L
O-O--0.567900	0	-3.962246	-9.014812	4.484827	L
C-CT-0.211700	0	-3.958092	-9.832489	1.486515	L
O-OH--0.654600	-1	-3.082657	-10.931545	1.287403	L

H-H-0.271900	0	-5.792748	-8.159575	2.572878	L		
H-H1-0.084300	0	-5.365385	-11.129227	2.435902	L		
H-H1-0.035200	0	-3.386465	-8.934505	1.726841	L		
H-H1-0.035200	0	-4.507951	-9.657592	0.560919	L		
H-HO-0.427500	-1	-2.542860	-10.743237	0.516164	L		
N-N--0.4157	0	-4.063619	-11.268578	4.552868	L		
C-CT--0.1490	-1	-3.520733	-11.414950	5.892075	L		
H-H-0.2719	0	-4.336536	-12.087919	4.033952	L		
H-H1-0.0976	0	-3.295508	-12.461352	6.097749	L		
H-H1-0.0976	0	-4.241854	-11.049539	6.626543	L		
H-H1-0.0976	0	-2.605312	-10.826232	5.989079	L		
C-CT--0.3662	-1	-12.148229	5.071702	-0.649710	L		
C-C-0.5972	0	-10.719272	5.257430	-0.211502	L		
O-O--0.5679	0	-10.404908	4.992137	0.943399	L		
H-HC-0.1123	0	-12.542128	6.022359	-1.002163	L		
H-HC-0.1123	0	-12.733478	4.723175	0.199543	L		
H-HC-0.1123	0	-12.181219	4.327339	-1.443164	L		
N-N--0.415700	0	-9.870606	5.702796	-1.134067	L		
C-CT--0.025200	-1	-8.437235	5.864905	-0.905897	L		
C-C-0.597300	0	-7.617976	5.793471	-2.169984	L		
O-O--0.567900	0	-8.118735	6.142715	-3.246078	L		
H-H-0.271900	0	-10.181264	5.865985	-2.079339	L		
H-H1-0.069800	0	-8.118819	5.055356	-0.258784	L		
H-H1-0.069800	0	-8.237721	6.813708	-0.409930	L		
N-N--0.415700	0	-6.369764	5.351398	-2.004525	L	H-H1	489
C-CT--0.530209	-1	-5.354334	5.291208	-3.053168	H		
C-C-0.568093	0	-4.662006	3.940441	-2.965834	H		
O-O--0.528105	0	-4.694036	3.270275	-1.931463	H		
C-CT-0.298500	0	-4.383413	6.491661	-2.958037	L	H-H1	489
C-CT--0.319200	0	-5.111926	7.811542	-3.224115	L		
C-CT--0.319200	0	-3.675029	6.569841	-1.603436	L		
H-H-0.271900	0	-6.079961	5.022822	-1.085731	L		
H-H1-0.184096	0	-5.870969	5.384009	-4.016116	H		
H-HC--0.029700	0	-3.624284	6.385203	-3.731060	L		
H-HC-0.079100	0	-5.819566	8.015705	-2.422427	L		
H-HC-0.079100	0	-5.645826	7.746510	-4.170844	L		
H-HC-0.079100	0	-4.383945	8.620022	-3.274966	L		
H-HC-0.079100	0	-4.395276	6.685757	-0.795580	L		
H-HC-0.079100	0	-2.984042	7.410051	-1.594409	L		
H-HC-0.079100	0	-3.108524	5.651950	-1.433020	L		
N-N--0.610103	0	-3.979962	3.556392	-4.073217	H		
C-CT--0.314060	-1	-4.243468	3.998804	-5.430312	H		
C-C-0.597300	0	-5.356751	3.173837	-6.068160	L	H-H1	505
O-O--0.567900	0	-5.482491	1.967577	-5.855602	L		
C-CT--0.110200	0	-2.951010	3.914577	-6.280246	L	H-H1	505
C-CT-0.353100	0	-1.957395	5.081325	-6.064764	L		
C-CT--0.412100	0	-1.080788	4.960330	-4.818068	L		
C-CT--0.412100	0	-1.009853	5.179797	-7.265411	L		
H-H-0.398207	0	-3.436247	2.706300	-3.971572	H		
H-H1-0.165130	0	-4.559187	5.041636	-5.431586	H		
H-HC-0.045700	0	-3.250958	3.929517	-7.328013	L		
H-HC-0.045700	0	-2.448027	2.964580	-6.104698	L		
H-HC--0.036100	0	-2.517559	6.015591	-6.012000	L		
H-HC-0.100000	0	-0.462761	4.066457	-4.878965	L		

H-HC-0.100000	0	-1.707840	4.875992	-3.929622	L		
H-HC-0.100000	0	-0.449196	5.840133	-4.710244	L		
H-HC-0.100000	0	-0.436491	4.257847	-7.355412	L		
H-HC-0.100000	0	-0.326008	6.015805	-7.120837	L		
H-HC-0.100000	0	-1.583107	5.345027	-8.175688	L		
N-N--0.415700	0	-6.146920	3.824739	-6.916997	L		
C-CT--0.038900	-1	-7.336216	3.227923	-7.536112	L		
C-C-0.597300	0	-7.090985	1.891686	-8.226757	L		
O-O--0.567900	0	-7.972867	1.036089	-8.272429	L		
C-CT-0.365400	-1	-7.974968	4.151999	-8.597264	L		
O-OH--0.676100	-1	-7.913640	5.509233	-8.149373	L		
C-CT--0.243800	0	-9.453708	3.862059	-8.854814	L		
H-H-0.271900	0	-6.023418	4.823089	-6.998468	L		
H-H1-0.100700	0	-8.074892	3.086488	-6.754108	L		
H-H1-0.004300	0	-7.420678	4.076108	-9.533749	L		
H-HC-0.064200	0	-9.569735	2.858526	-9.263670	L		
H-HC-0.064200	0	-9.848639	4.576495	-9.576866	L		
H-HC-0.064200	0	-10.016078	3.932562	-7.922956	L		
H-HO-0.410200	-1	-8.811994	5.843935	-8.113091	L		
N-N--0.415700	0	-5.914207	1.755483	-8.834722	L		
C-CT--0.025200	-1	-5.529531	0.599683	-9.631301	L		
C-C-0.597300	0	-4.637027	-0.408976	-8.937344	L		
O-O--0.567900	0	-4.141440	-1.311511	-9.612896	L		
H-H-0.271900	0	-5.226214	2.467873	-8.641219	L		
H-H1-0.069800	0	-4.996777	0.947930	-10.514953	L		
H-H1-0.069800	0	-6.418603	0.068523	-9.970945	L		
N-N--0.415700	0	-4.425344	-0.238071	-7.627524	L	H-H1	545
C-CT--0.539097	-1	-3.482857	-1.010239	-6.829082	H		
C-C-0.572106	0	-4.212923	-1.475975	-5.569499	H		
O-O--0.533001	0	-4.924405	-2.474844	-5.636640	H		
C-CT-0.130300	0	-2.146908	-0.243746	-6.626128	L	H-H1	545
C-CT--0.043000	0	-1.543774	0.277288	-7.952591	L		
C-CT--0.320400	0	-1.168560	-1.208252	-5.933168	L		
C-CT--0.066000	0	-0.230928	1.055905	-7.792162	L		
H-H-0.271900	0	-4.889173	0.533535	-7.155994	L		
H-H1-0.180646	0	-3.254900	-1.924429	-7.387139	H		
H-HC-0.018700	0	-2.324018	0.612482	-5.976890	L		
H-HC-0.023600	0	-2.253591	0.960012	-8.418137	L		
H-HC-0.023600	0	-1.380283	-0.560698	-8.631268	L		
H-HC-0.088200	0	-0.963576	-2.064069	-6.574538	L		
H-HC-0.088200	0	-1.585449	-1.559949	-4.991109	L		
H-HC-0.088200	0	-0.237919	-0.701057	-5.691858	L		
H-HC-0.018600	0	-0.356050	1.839650	-7.045464	L		
H-HC-0.018600	0	0.035154	1.512828	-8.745306	L		
H-HC-0.018600	0	0.576474	0.388948	-7.491359	L		
N-N--0.611145	0	-4.075608	-0.718882	-4.446630	H		
C-CT--0.109296	-1	-4.643963	-1.112497	-3.166014	H		
C-C-0.597300	0	-6.171156	-1.142131	-3.181817	L	H-H1	564
O-O--0.567900	0	-6.800349	-2.084188	-2.684255	L		
C-CT--0.388038	0	-4.127217	-0.289846	-2.006450	H		
H-H-0.369964	0	-3.450558	0.076434	-4.431266	H		
H-H1-0.178541	0	-4.384480	-2.159977	-2.977167	H		
H-H1-0.157964	0	-4.728321	-0.569904	-1.136116	H		
H-H1-0.179833	0	-4.231096	0.787428	-2.160685	H		

H-HS-0.319500	0	-2.234797	0.182342	-0.682205	H
N-N--0.415700	0	-6.763724	-0.140597	-3.818899	L
C-CT--0.087500	-1	-8.202025	-0.074434	-4.058870	L
C-C-0.597300	0	-8.690924	-1.222612	-4.928919	L
O-O--0.567900	0	-9.637157	-1.916995	-4.557323	L
C-CT-0.298500	0	-8.598580	1.290494	-4.641156	L
C-CT--0.319200	0	-10.115522	1.422001	-4.834820	L
C-CT--0.319200	0	-8.149397	2.462596	-3.754523	L
H-H-0.271900	0	-6.129873	0.555394	-4.204482	L
H-H1-0.096900	0	-8.702750	-0.175922	-3.098541	L
H-HC--0.029700	0	-8.121466	1.378701	-5.608805	L
H-HC-0.079100	0	-10.624478	1.271934	-3.882431	L
H-HC-0.079100	0	-10.467819	0.682802	-5.553945	L
H-HC-0.079100	0	-10.351893	2.413611	-5.219688	L
H-HC-0.079100	0	-8.602996	2.394005	-2.767698	L
H-HC-0.079100	0	-8.424111	3.412329	-4.212623	L
H-HC-0.079100	0	-7.065244	2.459126	-3.635059	L
N-N--0.415700	0	-8.053868	-1.434837	-6.079093	L
C-CT--0.025200	-1	-8.413516	-2.491713	-7.021212	L
C-C-0.597300	0	-8.322402	-3.893362	-6.431441	L
O-O--0.567900	0	-9.219693	-4.710384	-6.628099	L
H-H-0.271900	0	-7.255336	-0.845709	-6.278855	L
H-H1-0.069800	0	-7.747018	-2.439897	-7.881874	L
H-H1-0.069800	0	-9.434743	-2.330006	-7.365687	L
N-N--0.415700	0	-7.239812	-4.167816	-5.705934	L
C-CT--0.025200	-1	-7.016082	-5.433453	-5.018265	L
C-C-0.597300	0	-7.948117	-5.696593	-3.846735	L
O-O--0.567900	0	-8.283397	-6.848727	-3.576926	L
H-H-0.271900	0	-6.524931	-3.444974	-5.636363	L
H-H1-0.069800	0	-5.992878	-5.450310	-4.644505	L
H-H1-0.069800	0	-7.132785	-6.249513	-5.731473	L
N-N--0.415700	0	-8.334763	-4.640329	-3.135496	L
C-CT--0.038900	-1	-9.384107	-4.696705	-2.115562	L
C-C-0.597300	0	-10.755498	-4.989092	-2.715289	L
O-O--0.567900	0	-11.498251	-5.824116	-2.198553	L
C-CT-0.365400	0	-9.418486	-3.392315	-1.304575	L
O-OH--0.676100	0	-8.178964	-3.222026	-0.655517	L
C-CT--0.243800	0	-10.500579	-3.400205	-0.225358	L
H-H-0.271900	0	-7.942282	-3.737037	-3.379406	L
H-H1-0.100700	0	-9.155807	-5.510305	-1.427228	L
H-H1-0.004300	0	-9.588540	-2.543635	-1.967266	L
H-HC-0.064200	0	-11.482562	-3.294188	-0.685080	L
H-HC-0.064200	0	-10.342532	-2.568013	0.460214	L
H-HC-0.064200	0	-10.465607	-4.338358	0.326647	L
H-HO-0.410200	0	-7.592349	-2.768225	-1.294570	L
N-N--0.4157	0	-11.106494	-4.282598	-3.791100	L
C-CT--0.1490	-1	-12.338961	-4.476316	-4.526921	L
H-H-0.2719	0	-10.440702	-3.598609	-4.140222	L
H-H1-0.0976	0	-12.416405	-3.723233	-5.312095	L
H-H1-0.0976	0	-13.190906	-4.392744	-3.850820	L
H-H1-0.0976	0	-12.335294	-5.468754	-4.980708	L
N-N2--0.511415	0	1.999821	-0.010339	-1.634197	H
C-CA-0.057801	0	0.836701	0.654112	-1.573357	H
C-CA-0.063496	0	0.259271	1.246339	-2.698806	H

C-CA--0.109122	0	0.942766	1.167904	-3.915969	H
C-CA--0.164490	0	2.159168	0.508186	-3.968805	H
C-CA-0.098392	0	2.646649	-0.078441	-2.807933	H
C-C-0.541263	0	-1.095190	1.886025	-2.744997	H
O-O--0.574884	0	-1.655363	1.947078	-3.834845	H
H-H4-0.220889	0	0.366610	0.702587	-0.594352	H
H-HA-0.160434	0	0.496155	1.617695	-4.793836	H
H-HA-0.142191	0	2.724912	0.445432	-4.889793	H
H-H4-0.174502	0	3.574285	-0.639220	-2.801309	H
H-H-0.353801	0	-1.413473	2.073061	-0.760589	H
Zn-ZN-0.974537	0	2.854607	-1.136707	0.034459	H
O-OW--0.834000	0	-2.130435	-7.249891	3.494314	L
H-HW-0.417000	0	-2.567627	-6.669346	2.851603	L
H-HW-0.417000	0	-2.854761	-7.794894	3.843129	L
O-OW--0.783903	0	1.315433	-2.601686	-0.121103	H
H-HW-0.401253	0	0.948882	-2.498552	0.793489	H
H-HW-0.424111	0	1.924207	-3.382277	-0.176565	H
O-OW--0.834000	0	-3.693900	-8.455165	7.232887	L
H-HW-0.417000	0	-3.750604	-7.511029	7.400780	L
H-HW-0.417000	0	-3.814038	-8.530614	6.274051	L
O-OW--0.834000	0	7.162768	-0.009489	1.787711	L
H-HW-0.417000	0	6.859343	0.404991	2.615873	L
H-HW-0.417000	0	6.371991	0.078065	1.231502	L
O-OW--0.834000	0	7.053622	-4.962412	4.772815	L
H-HW-0.417000	0	6.102698	-5.014210	5.000249	L
H-HW-0.417000	0	7.253695	-5.846488	4.463057	L
O-OW--0.834000	0	8.634580	-0.919635	-0.256030	L
H-HW-0.417000	0	8.159449	-0.547219	0.514715	L
H-HW-0.417000	0	8.841579	-0.130118	-0.776754	L
O-OW--0.834000	0	12.643642	-1.068486	-2.001739	L
H-HW-0.417000	0	11.788840	-0.818318	-2.392728	L
H-HW-0.417000	0	12.648603	-2.031583	-2.112997	L
O-OW--0.834000	0	8.287255	-3.292341	0.978875	L
H-HW-0.417000	0	8.499184	-2.521284	0.412278	L
H-HW-0.417000	0	7.554529	-2.957938	1.506618	L
O-OW--0.834000	0	4.362781	-4.906888	5.169246	L
H-HW-0.417000	0	4.095841	-4.521460	6.026686	L
H-HW-0.417000	0	4.054785	-4.243133	4.541631	L
S-SH--0.326142	0	-2.375790	-0.736484	-1.666862	H
O-OW--0.834000	0	-1.638684	2.435323	-1.672633	H

TS5

C-CT--0.366200	-1	-10.309951	3.920915	4.014481	L
C-C-0.597200	0	-8.979827	3.269528	3.770742	L
O-O--0.567900	0	-8.404572	2.698437	4.690405	L
H-HC-0.112300	0	-11.052743	3.459970	3.366426	L
H-HC-0.112300	0	-10.230279	4.982544	3.787033	L
H-HC-0.112300	0	-10.589093	3.783489	5.056679	L
N-N--0.415700	0	-8.517555	3.358780	2.530906	L
C-CT--0.059700	-1	-7.343712	2.648442	2.016700	L
C-C-0.597300	0	-6.197799	3.631492	1.838846	L
O-O--0.567900	0	-6.362490	4.644334	1.164895	L
C-CT-0.130300	0	-7.693531	1.908272	0.701473	L
C-CT--0.043000	0	-8.895761	0.948954	0.898909	L

```
C-CT--0.320400   0   -6.476722    1.114856    0.196792 L
C-CT--0.066000   0   -9.411883    0.306467   -0.393452 L
H-H-0.271900     0   -9.051539    3.924315    1.879272 L
H-H1-0.086900    0   -7.034384    1.897520    2.743584 L
H-HC-0.018700    0   -7.960084    2.649770   -0.053908 L
H-HC-0.023600    0   -9.735708    1.496185    1.325037 L
H-HC-0.023600    0   -8.620133    0.159940    1.599821 L
H-HC-0.088200    0   -6.203221    0.333780    0.906313 L
H-HC-0.088200    0   -5.621410    1.775606    0.050360 L
H-HC-0.088200    0   -6.693025    0.671205   -0.773058 L
H-HC-0.018600    0   -9.629814    1.078440   -1.130270 L
H-HC-0.018600    0  -10.329067   -0.241435   -0.180347 L
H-HC-0.018600    0   -8.675528   -0.390156   -0.791142 L
N-N--0.516300    0   -5.077790    3.338272    2.495936 L
C-CT-0.038100   -1   -3.776896    3.982324    2.375400 L H-HC 30
C-C-0.536600     0   -3.703797    5.509368    2.180213 L
O-O--0.581900    0   -2.710827    6.046327    1.690615 L
C-CT--0.547145   0   -3.027303    3.265739    1.227948 H
C-C-0.532653     0   -2.377907    1.964722    1.605542 H
O-O2--0.588507   0   -2.527579    1.413479    2.699350 H
O-O2--0.625559   0   -1.602075    1.418167    0.681544 H
H-H-0.293600     0   -5.051621    2.449304    2.976888 L
H-H1-0.088000    0   -3.222978    3.790884    3.294838 L
H-HC-0.128435    0   -2.204392    3.897021    0.861849 H
H-HC-0.183556    0   -3.670952    3.155100    0.346847 H
N-N--0.415700    0   -4.690154    6.260086    2.645949 L
C-CT--0.025200  -1   -4.652183    7.701171    2.493424 L
C-C-0.597300     0   -3.758532    8.351223    3.536694 L
O-O--0.567900    0   -4.237516    9.017178    4.456088 L
H-H-0.271900     0   -5.535465    5.767119    2.888368 L
H-H1-0.069800    0   -5.660323    8.096574    2.607379 L
H-H1-0.069800    0   -4.293790    7.966436    1.497288 L
N-N--0.415700    0   -2.454489    8.140728    3.392480 L
C-CT--0.038900  -1   -1.428043    8.565539    4.330290 L
C-C-0.597300     0   -0.513492    9.639566    3.730028 L
O-O--0.567900    0   -0.605163    9.998381    2.554911 L
C-CT-0.365400    0   -0.591254    7.411699    4.926587 L
O-OH--0.676100   0    0.608006    7.237365    4.214327 L
C-CT--0.243800   0   -1.306009    6.069757    5.041381 L
H-H-0.271900     0   -2.191877    7.542606    2.611415 L
H-H1-0.100700    0   -1.931279    9.034732    5.173746 L
H-H1-0.004300    0   -0.311105    7.712904    5.936558 L
H-HC-0.064200    0   -2.252390    6.200693    5.564026 L
H-HC-0.064200    0   -0.678162    5.380231    5.600603 L
H-HC-0.064200    0   -1.488426    5.650544    4.052672 L
H-HO-0.410200    0    1.094496    6.480656    4.607077 L
N-N--0.415700    0    0.399397   10.157811    4.555510 L
C-CT--0.025200  -1    1.440132   11.091642    4.138946 L
C-C-0.597300     0    2.342559   10.600678    3.016013 L
O-O--0.567900    0    2.680813   11.376849    2.127375 L
H-H-0.271900     0    0.457458    9.760362    5.477753 L
H-H1-0.069800    0    2.074334   11.327587    4.992291 L
H-H1-0.069800    0    0.968024   12.014561    3.801532 L
N-N--0.516300    0    2.735886    9.330706    3.072512 L
```

C-CT-0.038100	-1	3.644412	8.731374	2.096010	L
C-C-0.536600	0	3.014130	8.439879	0.747426	L
O-O--0.581900	0	3.746749	8.142056	-0.189702	L
C-CT--0.030300	0	4.228534	7.418482	2.648877	L
C-C-0.799400	-1	5.575538	7.620370	3.324851	L
O-O2--0.801400	-1	6.155993	8.724286	3.240691	L
O-O2--0.801400	-1	6.035647	6.668167	3.987293	L
H-H-0.293600	0	2.359133	8.725038	3.787875	L
H-H1-0.088000	0	4.447110	9.437593	1.881204	L
H-HC--0.012200	0	4.384314	6.702340	1.840289	L
H-HC--0.012200	0	3.525851	6.963145	3.349987	L
N-N--0.415700	0	1.692323	8.527588	0.634143	L
C-CT--0.002400	-1	0.972659	8.316322	-0.614681	L
C-C-0.597300	0	0.373720	9.607068	-1.175318	L
O-O--0.567900	0	0.356949	9.814844	-2.388644	L
C-CT--0.034300	0	-0.088470	7.223340	-0.432743	L
C-CA-0.011800	0	0.442373	5.905649	0.108834	L
C-CA--0.125600	0	1.084081	4.980708	-0.735114	L
C-CA--0.125600	0	0.260357	5.587769	1.464669	L
C-CA--0.170400	0	1.522020	3.746339	-0.219193	L
C-CA--0.170400	0	0.672479	4.347683	1.977164	L
C-CA--0.107200	0	1.298033	3.422480	1.128796	L
H-H-0.271900	0	1.172323	8.728262	1.475702	L
H-H1-0.097800	0	1.662504	7.948909	-1.373583	L
H-HC-0.029500	0	-0.554194	7.029530	-1.399802	L
H-HC-0.029500	0	-0.868305	7.594247	0.234151	L
H-HA-0.133000	0	1.203569	5.201183	-1.784664	L
H-HA-0.133000	0	-0.254448	6.286495	2.098406	L
H-HA-0.143000	0	1.986498	3.018262	-0.863993	L
H-HA-0.143000	0	0.467921	4.093496	3.005743	L
H-HA-0.129700	0	1.570881	2.448726	1.501767	L
N-N--0.415700	0	-0.080873	10.504596	-0.298070	L
C-CT--0.149000	-1	-0.475166	11.850526	-0.663171	L
H-H-0.271900	0	-0.074873	10.244682	0.683032	L
H-H1-0.097600	0	-0.863083	12.367606	0.214897	L
H-H1-0.097600	0	-1.246656	11.808951	-1.433887	L
H-H1-0.097600	0	0.390489	12.389818	-1.050593	L
C-CT--0.366200	-1	7.983068	8.430931	-0.185824	L
C-C-0.597200	0	7.521318	7.629893	-1.380801	L
O-O--0.567900	0	8.342894	7.280017	-2.224696	L
H-HC-0.112300	0	7.530834	9.419742	-0.216378	L
H-HC-0.112300	0	9.067136	8.514230	-0.206904	L
H-HC-0.112300	0	7.679411	7.921335	0.729527	L
N-N--0.415700	0	6.211709	7.381782	-1.475688	L
C-CT--0.051800	-1	5.559479	6.850421	-2.680441	L
C-C-0.597300	0	4.919167	7.975826	-3.493036	L
O-O--0.567900	0	5.305606	8.244714	-4.630904	L
C-CT--0.110200	0	4.617310	5.673311	-2.342010	L
C-CT-0.353100	0	4.565772	4.619474	-3.469213	L
C-CT--0.412100	0	3.796791	3.378987	-3.029007	L
C-CT--0.412100	0	3.897335	5.115899	-4.753768	L
H-H-0.271900	0	5.606955	7.704555	-0.727495	L
H-H1-0.092200	0	6.346885	6.446462	-3.317926	L
H-HC-0.045700	0	3.613745	6.035748	-2.117628	L

H-HC-0.045700	0	4.996491	5.177658	-1.448138	L
H-HC--0.036100	0	5.584239	4.307060	-3.704493	L
H-HC-0.100000	0	2.735648	3.608474	-2.937223	L
H-HC-0.100000	0	4.171266	3.023554	-2.070674	L
H-HC-0.100000	0	3.926841	2.588561	-3.765453	L
H-HC-0.100000	0	2.917019	5.535532	-4.524268	L
H-HC-0.100000	0	3.777257	4.289968	-5.453881	L
H-HC-0.100000	0	4.515306	5.874794	-5.228663	L
N-N--0.415700	0	3.971229	8.658868	-2.865396	L
C-CT--0.149000	-1	3.352364	9.879891	-3.312443	L
H-H-0.271900	0	3.770829	8.344789	-1.923018	L
H-H1-0.097600	0	4.065747	10.474300	-3.884699	L
H-H1-0.097600	0	3.011006	10.454923	-2.450420	L
H-H1-0.097600	0	2.493433	9.643754	-3.942845	L
C-CT--0.366200	-1	-8.721471	2.384235	8.124356	L
C-C-0.597200	0	-7.843359	1.178030	7.947823	L
O-O--0.567900	0	-7.517213	0.502882	8.917577	L
H-HC-0.112300	0	-9.641646	2.236291	7.562439	L
H-HC-0.112300	0	-8.197639	3.261654	7.748068	L
H-HC-0.112300	0	-8.945621	2.510429	9.181419	L
N-N--0.415700	0	-7.455996	0.922103	6.706379	L
C-CT--0.038900	-1	-6.661219	-0.236988	6.296034	L
C-C-0.597300	0	-5.388648	0.233503	5.614524	L
O-O--0.567900	0	-5.410729	0.627245	4.448659	L
C-CT-0.365400	0	-7.473682	-1.201792	5.418476	L
O-OH--0.676100	0	-8.028636	-0.538138	4.308842	L
C-CT--0.243800	0	-8.624032	-1.818424	6.219764	L
H-H-0.271900	0	-7.790633	1.538338	5.969271	L
H-H1-0.100700	0	-6.365240	-0.800177	7.180766	L
H-H1-0.004300	0	-6.818754	-2.000165	5.067244	L
H-HC-0.064200	0	-8.226992	-2.352092	7.082896	L
H-HC-0.064200	0	-9.181210	-2.514890	5.599148	L
H-HC-0.064200	0	-9.307656	-1.039960	6.556850	L
H-HO-0.410200	0	-7.331519	0.031517	3.953129	L
N-N--0.415700	0	-4.305586	0.249630	6.395496	L
C-CT--0.059700	-1	-3.107954	1.057613	6.155887	L
C-C-0.597300	0	-1.863866	0.178527	6.071676	L
O-O--0.567900	0	-1.601227	-0.620376	6.975429	L
C-CT-0.130300	0	-2.971857	2.153432	7.238151	L
C-CT--0.043000	0	-4.229406	3.044578	7.396342	L
C-CT--0.320400	0	-1.740331	3.028467	6.962603	L
C-CT--0.066000	0	-4.681688	3.799933	6.137372	L
H-H-0.271900	0	-4.385244	-0.154568	7.314442	L
H-H1-0.086900	0	-3.214148	1.558828	5.192145	L
H-HC-0.018700	0	-2.811267	1.659764	8.198452	L
H-HC-0.023600	0	-4.036661	3.778314	8.179809	L
H-HC-0.023600	0	-5.060038	2.430014	7.742796	L
H-HC-0.088200	0	-1.755499	3.369850	5.927303	L
H-HC-0.088200	0	-0.832865	2.445323	7.119729	L
H-HC-0.088200	0	-1.720840	3.884994	7.635192	L
H-HC-0.018600	0	-3.888817	4.453872	5.778358	L
H-HC-0.018600	0	-5.555039	4.406904	6.376303	L
H-HC-0.018600	0	-4.954846	3.100238	5.350534	L
N-N--0.516300	0	-1.121214	0.347229	4.978998	L

C-CT-0.038100	-1	0.186972	-0.231541	4.720715	L	H-HC	17
C-C-0.536600	0	1.111186	-0.074767	5.934426	L		
O-O--0.581900	0	1.312361	1.016772	6.460522	L		
C-CT--0.520019	0	0.716746	0.414418	3.428570	H		
C-C-0.636404	0	0.962004	-0.538280	2.269564	H		
O-O2--0.608036	0	0.333517	-1.638060	2.181665	H		
O-O2--0.608131	0	1.808314	-0.109335	1.425263	H		
H-H-0.293600	0	-1.505568	0.937839	4.246530	L		
H-H1-0.088000	0	0.043107	-1.300647	4.558430	L		
H-HC-0.132300	0	1.661984	0.918021	3.643140	H		
H-HC-0.188993	0	0.027884	1.188300	3.075883	H		
N-N--0.415700	0	1.665028	-1.184160	6.391673	L		
C-CT-0.033700	-1	2.572815	-1.229408	7.514911	L		
C-C-0.597300	0	3.792407	-2.050238	7.111631	L		
O-O--0.567900	0	3.841826	-3.275586	7.267352	L		
C-CT--0.182500	0	1.824293	-1.785997	8.734301	L		
H-H-0.271900	0	1.369656	-2.062364	5.979858	L		
H-H1-0.082300	0	2.918440	-0.223650	7.761718	L		
H-HC-0.060300	0	0.985398	-1.131440	8.974094	L		
H-HC-0.060300	0	2.500427	-1.834401	9.587563	L		
H-HC-0.060300	0	1.447035	-2.786061	8.514632	L		
N-N--0.415700	0	4.762583	-1.355823	6.529425	L		
C-CT-0.018800	-1	5.967376	-1.940046	5.984914	L	H-HC	20
C-C-0.597300	0	7.165411	-1.789328	6.931583	L		
O-O--0.567900	0	7.212460	-0.939337	7.823290	L		
C-CT--0.517456	0	6.219073	-1.311261	4.612389	H		
C-CM-0.287351	0	5.341002	-1.790657	3.515394	H		
N-N2--0.586994	0	5.479224	-3.045461	2.938386	H		
C-CM--0.020338	0	4.356463	-1.183159	2.787176	H		
C-CM-0.242458	0	4.605352	-3.135793	1.900621	H		
N-N2--0.505070	0	3.914800	-2.017118	1.780140	H		
H-H-0.271900	0	4.658027	-0.346716	6.468020	L		
H-H1-0.088100	0	5.820607	-3.006939	5.849119	L		
H-HC-0.166355	0	7.271862	-1.483999	4.344782	H		
H-HC-0.174481	0	6.112964	-0.231482	4.742984	H		
H-H-0.335795	0	6.132430	-3.760339	3.251098	H		
H-H4-0.152777	0	3.959853	-0.186956	2.911353	H		
H-H5-0.209475	0	4.502866	-4.002321	1.262180	H		
N-N--0.415700	0	8.170117	-2.627972	6.685071	L		
C-CT--0.149000	-1	9.439225	-2.557862	7.375954	L		
H-H-0.271900	0	8.017809	-3.306870	5.945788	L		
H-H1-0.097600	0	10.111951	-3.327360	6.996140	L		
H-H1-0.097600	0	9.281346	-2.706149	8.445590	L		
H-H1-0.097600	0	9.886431	-1.574613	7.218853	L		
C-CT--0.366200	-1	8.357870	-9.388373	0.928827	L		
C-C-0.597200	0	8.735846	-9.164159	-0.522947	L		
O-O--0.567900	0	9.770493	-8.562616	-0.784299	L		
H-HC-0.112300	0	8.277427	-10.453968	1.130083	L		
H-HC-0.112300	0	7.418645	-8.881615	1.143108	L		
H-HC-0.112300	0	9.136716	-8.957816	1.558086	L		
N-N--0.254800	0	7.915593	-9.622426	-1.485183	L		
C-CT--0.026600	-1	8.156446	-9.387001	-2.911057	L		
C-C-0.589600	0	8.009600	-7.914290	-3.294107	L		
O-O--0.574800	0	8.722801	-7.419489	-4.163855	L		

C-CT--0.007000	0	7.146100	-10.278834	-3.640446	L	
C-CT-0.018900	0	5.987549	-10.400427	-2.649236	L	
C-CT-0.019200	0	6.682158	-10.372053	-1.286920	L	
H-H1-0.064100	0	9.167734	-9.700328	-3.173441	L	
H-HC-0.025300	0	7.587243	-11.263434	-3.802697	L	
H-HC-0.025300	0	6.824483	-9.848685	-4.590250	L	
H-HC-0.021300	0	5.424618	-11.322712	-2.795752	L	
H-HC-0.021300	0	5.330473	-9.534429	-2.743548	L	
H-H1-0.039100	0	6.036601	-9.895369	-0.547876	L	
H-H1-0.039100	0	6.924382	-11.388969	-0.975343	L	
N-N--0.516300	0	7.086265	-7.216931	-2.631581	L	
C-CT-0.039700	-1	6.832276	-5.790681	-2.805478	L	
C-C-0.536600	0	8.091184	-4.924591	-2.678710	L	
O-O--0.581900	0	8.265683	-3.943238	-3.404690	L	
C-CT-0.056000	0	5.734078	-5.354118	-1.830618	L	H-HC 24
C-CT--0.515956	0	5.200520	-3.970007	-2.209997	H	
C-C-0.587828	0	4.112040	-3.433901	-1.289629	H	
O-O2--0.600319	0	4.065967	-2.152299	-1.240837	H	
O-O2--0.589127	0	3.348265	-4.221356	-0.685078	H	
H-H-0.293600	0	6.551318	-7.692912	-1.924435	L	
H-H1-0.110500	0	6.455489	-5.639108	-3.817398	L	
H-HC--0.017300	0	6.129902	-5.332977	-0.814058	L	
H-HC--0.017300	0	4.912432	-6.070883	-1.876331	L	
H-HC-0.167759	0	4.791215	-4.022630	-3.226068	H	
H-HC-0.135230	0	6.021958	-3.245804	-2.227538	H	
N-N--0.415700	0	9.001565	-5.328197	-1.795044	L	
C-CT--0.024900	-1	10.241686	-4.628163	-1.481390	L	
C-C-0.597300	0	11.263249	-4.520696	-2.606705	L	
O-O--0.567900	0	12.245564	-3.790540	-2.473912	L	
C-CT-0.211700	0	10.881959	-5.263172	-0.240825	L	
O-OH--0.654600	0	9.925543	-5.452280	0.791701	L	
H-H-0.271900	0	8.790713	-6.134852	-1.224035	L	
H-H1-0.084300	0	9.976413	-3.603427	-1.216629	L	
H-H1-0.035200	0	11.681289	-4.615119	0.121587	L	
H-H1-0.035200	0	11.307872	-6.231546	-0.507736	L	
H-HO-0.427500	0	9.376997	-4.640101	0.872885	L	
N-N--0.415700	0	11.021919	-5.205138	-3.722967	L	
C-CT-0.033700	-1	11.771676	-5.043563	-4.961951	L	
C-C-0.597300	0	10.939370	-4.487631	-6.122043	L	
O-O--0.567900	0	11.372815	-4.500081	-7.273399	L	
C-CT--0.182500	0	12.397854	-6.405562	-5.295226	L	
H-H-0.271900	0	10.206491	-5.807519	-3.730095	L	
H-H1-0.082300	0	12.587681	-4.336041	-4.808643	L	
H-HC-0.060300	0	13.014088	-6.743027	-4.461154	L	
H-HC-0.060300	0	13.024054	-6.315506	-6.183493	L	
H-HC-0.060300	0	11.611300	-7.137893	-5.484267	L	
N-N--0.415700	0	9.759477	-3.970313	-5.797461	L	
C-CT--0.025200	-1	8.855701	-3.289557	-6.701832	L	
C-C-0.597300	0	8.807161	-1.785276	-6.484028	L	
O-O--0.567900	0	8.825913	-1.014015	-7.440450	L	
H-H-0.271900	0	9.480513	-4.026294	-4.824125	L	
H-H1-0.069800	0	7.849191	-3.681222	-6.557876	L	
H-H1-0.069800	0	9.145543	-3.474188	-7.736705	L	
N-N--0.415700	0	8.744664	-1.377702	-5.219619	L	

```
C-CT--0.002400  -1    8.575198    0.008246   -4.800359 L
C-C-0.597300     0    9.695190    0.443114   -3.847959 L
O-O--0.567900    0   10.323840   -0.407890   -3.209780 L
C-CT--0.034300   0    7.192364    0.131345   -4.134464 L
C-CA-0.011800    0    6.038487   -0.118161   -5.088050 L
C-CA--0.125600   0    5.616332    0.899266   -5.964992 L
C-CA--0.125600   0    5.413196   -1.378703   -5.130905 L
C-CA--0.170400   0    4.586137    0.652566   -6.890561 L
C-CA--0.170400   0    4.378879   -1.623050   -6.051673 L
C-CA--0.107200   0    3.971089   -0.611218   -6.938408 L
H-H-0.271900     0    8.745842   -2.084169   -4.493354 L
H-H1-0.097800    0    8.599605    0.658126   -5.675280 L
H-HC-0.029500    0    7.072942    1.125824   -3.705437 L
H-HC-0.029500    0    7.137799   -0.579952   -3.308684 L
H-HA-0.133000    0    6.087948    1.870817   -5.935703 L
H-HA-0.133000    0    5.734073   -2.164104   -4.462464 L
H-HA-0.143000    0    4.263543    1.436162   -7.561425 L
H-HA-0.143000    0    3.894472   -2.588262   -6.076786 L
H-HA-0.129700    0    3.171605   -0.799253   -7.641604 L
N-N--0.254800    0    9.950532    1.756760   -3.703888 L
C-CT--0.026600  -1   10.814521    2.251927   -2.638600 L
C-C-0.589600     0   10.180601    2.006656   -1.260759 L
O-O--0.574800    0    8.986892    1.699258   -1.181214 L
C-CT--0.007000   0   10.974442    3.751032   -2.924206 L
C-CT-0.018900    0    9.655986    4.119721   -3.602245 L
C-CT-0.019200    0    9.343282    2.870828   -4.426367 L
H-H1-0.064100    0   11.783795    1.757472   -2.693684 L
H-HC-0.025300    0   11.797673    3.902454   -3.623870 L
H-HC-0.025300    0   11.134992    4.336898   -2.017837 L
H-HC-0.021300    0    9.752988    5.007344   -4.228172 L
H-HC-0.021300    0    8.881263    4.266903   -2.847038 L
H-H1-0.039100    0    8.264576    2.762052   -4.529425 L
H-H1-0.039100    0    9.805237    2.955261   -5.410824 L
N-N--0.254800    0   10.946687    2.124497   -0.165856 L
C-CT--0.026600  -1   10.394538    2.113367    1.181788 L
C-C-0.589600     0    9.344679    3.199290    1.417867 L
O-O--0.574800    0    9.610105    4.379517    1.201832 L
C-CT--0.007000   0   11.595408    2.294590    2.121177 L
C-CT-0.018900    0   12.780980    1.807076    1.292016 L
C-CT-0.019200    0   12.396647    2.226020   -0.124062 L
H-H1-0.064100    0    9.952172    1.132564    1.362124 L
H-HC-0.025300    0   11.478450    1.714202    3.036919 L
H-HC-0.025300    0   11.740002    3.350073    2.359006 L
H-HC-0.021300    0   12.844104    0.718870    1.345728 L
H-HC-0.021300    0   13.717427    2.265542    1.610662 L
H-H1-0.039100    0   12.694506    3.260464   -0.300549 L
H-H1-0.039100    0   12.872139    1.560509   -0.845353 L
N-N--0.415700    0    8.174435    2.775198    1.885073 L
C-CT-0.018800   -1    7.018115    3.604717    2.178920 L H-HC 33
C-C-0.597300     0    6.152526    3.012527    3.289023 L
O-O--0.567900    0    6.177581    1.804608    3.524324 L
C-CT--0.515368   0    6.202300    3.723782    0.863812 H
C-CM-0.287903    0    5.607928    2.444563    0.353795 H
N-N2--0.582625   0    6.219491    1.664504   -0.614401 H
```

C-CM--0.036893	0	4.500270	1.713407	0.714729	H	
C-CM-0.243454	0	5.493027	0.530941	-0.791527	H	
N-N2--0.523998	0	4.445873	0.525685	0.012637	H	
H-H-0.271900	0	8.033103	1.778226	1.962184	L	
H-H1-0.088100	0	7.351829	4.599182	2.490365	L	
H-HC-0.162301	0	6.861788	4.175975	0.108735	H	
H-HC-0.188340	0	5.408977	4.460795	1.040151	H	
H-H-0.339448	0	7.123233	1.871091	-1.039689	H	
H-H4-0.185957	0	3.746151	1.965849	1.444409	H	
H-H5-0.211295	0	5.755957	-0.255588	-1.483506	H	
N-N--0.415700	0	5.371721	3.873560	3.929331	L	
C-CT-0.014300	-1	4.555547	3.596648	5.102377	L	
C-C-0.597300	0	5.209283	2.646871	6.116243	L	
O-O--0.567900	0	4.683874	1.596721	6.490599	L	
C-CT--0.204100	0	3.084462	3.358296	4.739337	L	
C-C-0.713000	0	2.205584	4.197516	5.650257	L	
N-N--0.919100	0	1.723636	3.671705	6.742095	L	
O-O--0.593100	0	1.981193	5.374752	5.430570	L	
H-H-0.271900	0	5.502112	4.859699	3.696996	L	
H-H1-0.104800	0	4.560396	4.556356	5.624629	L	
H-HC-0.079700	0	2.842323	2.305251	4.835719	L	
H-HC-0.079700	0	2.894705	3.672619	3.713897	L	
H-H-0.419600	0	1.094450	4.233059	7.274082	L	
H-H-0.419600	0	1.769846	2.656359	6.826526	L	
N-N--0.415700	0	6.397631	3.078003	6.552334	L	
C-CT--0.149000	-1	7.301901	2.340471	7.413178	L	
H-H-0.271900	0	6.709817	3.949850	6.147425	L	
H-H1-0.097600	0	8.054024	3.008132	7.832946	L	
H-H1-0.097600	0	7.792077	1.553207	6.837247	L	
H-H1-0.097600	0	6.741082	1.867421	8.222376	L	
C-CT--0.366200	-1	-2.244495	-2.725469	9.540677	L	
C-C-0.597200	0	-2.368274	-3.819757	8.516120	L	
O-O--0.567900	0	-2.899334	-4.885719	8.816019	L	
H-HC-0.112300	0	-1.190445	-2.508717	9.703198	L	
H-HC-0.112300	0	-2.703781	-3.053951	10.470549	L	
H-HC-0.112300	0	-2.749205	-1.833956	9.173020	L	
N-N--0.347900	0	-1.849334	-3.559333	7.319401	L	
C-CT--0.240000	-1	-1.740671	-4.530886	6.224752	L	
C-C-0.734100	0	-0.327870	-4.571440	5.654426	L	
O-O--0.589400	0	0.491791	-3.696354	5.917581	L	
C-CT--0.009400	0	-2.823504	-4.246078	5.164158	L	
C-CT-0.018700	0	-2.592160	-2.961426	4.358564	L	H-HC 37
C-CT--0.464524	0	-3.669686	-2.789520	3.282263	H	
C-CT--0.195159	0	-3.593816	-1.459721	2.545150	H	
N-N3--0.748751	0	-2.362729	-1.315421	1.695421	H	
H-H-0.274700	0	-1.462338	-2.628732	7.176349	L	
H-H1-0.142600	0	-1.933321	-5.524730	6.629279	L	
H-HC-0.036200	0	-3.794221	-4.185240	5.659232	L	
H-HC-0.036200	0	-2.868520	-5.077067	4.467595	L	
H-HC-0.010300	0	-1.612640	-2.991302	3.881162	L	
H-HC-0.010300	0	-2.639377	-2.113106	5.033702	L	
H-HC-0.173903	0	-4.649329	-2.836021	3.766493	H	
H-HC-0.148535	0	-3.616864	-3.630193	2.577839	H	
H-HP-0.236066	0	-3.617648	-0.601086	3.214715	H	

H-HP-0.172509	0	-4.439195	-1.369000	1.853029	H
H-H-0.437293	0	-2.207470	-0.318958	1.462533	H
H-H-0.366736	0	-2.494197	-1.803116	0.794377	H
H-H-0.419451	0	-1.455606	-1.616875	2.123901	H
N-N--0.415700	0	-0.033259	-5.604060	4.877443	L
C-CT--0.025200	-1	1.279322	-5.802388	4.271957	L
C-C-0.597300	0	1.151443	-6.519267	2.927300	L
O-O--0.567900	0	1.741426	-7.572664	2.695471	L
H-H-0.271900	0	-0.777602	-6.256204	4.648558	L
H-H1-0.069800	0	1.902721	-6.403041	4.934347	L
H-H1-0.069800	0	1.770057	-4.843505	4.101311	L
N-N--0.415700	0	0.328980	-5.937882	2.058129	L
C-CT--0.025200	-1	-0.233344	-6.541874	0.853700	L
C-C-0.597300	0	-1.385576	-5.681578	0.402616	L
O-O--0.567900	0	-2.290806	-5.459463	1.198187	L
H-H-0.271900	0	-0.114706	-5.088766	2.382088	L
H-H1-0.069800	0	-0.600628	-7.542695	1.080611	L
H-H1-0.069800	0	0.531965	-6.595074	0.078837	L
N-N--0.415700	0	-1.331493	-5.202668	-0.840824	L
C-CT--0.001400	-1	-2.160958	-4.113854	-1.363104	L
C-C-0.597300	0	-3.625863	-4.185380	-0.909669	L
O-O--0.567900	0	-4.186518	-3.229571	-0.382851	L
C-CT--0.015200	0	-2.057975	-4.090658	-2.903003	L
C-CA--0.001100	0	-0.667825	-4.304790	-3.477834	L
C-CA--0.190600	0	0.352977	-3.363677	-3.242802	L
C-CA--0.190600	0	-0.399700	-5.451054	-4.253359	L
C-CA--0.234100	0	1.639502	-3.570539	-3.776984	L
C-CA--0.234100	0	0.884321	-5.659371	-4.791695	L
C-C-0.322600	0	1.907912	-4.716354	-4.552906	L
O-OH--0.557900	0	3.142383	-4.916174	-5.086781	L
H-H-0.271900	0	-0.494961	-5.390254	-1.375095	L
H-H1-0.087600	0	-1.734424	-3.195029	-0.970876	L
H-HC-0.029500	0	-2.428195	-3.136648	-3.271166	L
H-HC-0.029500	0	-2.717933	-4.857012	-3.310031	L
H-HA-0.169900	0	0.154234	-2.472323	-2.656321	L
H-HA-0.169900	0	-1.188455	-6.161370	-4.453142	L
H-HA-0.165600	0	2.421475	-2.849821	-3.593662	L
H-HA-0.165600	0	1.080701	-6.530434	-5.394755	L
H-HO-0.399200	0	3.150292	-5.692353	-5.644023	L
N-N--0.415700	0	-4.228464	-5.363987	-1.082056	L
C-CT--0.024900	-1	-5.560883	-5.654507	-0.573462	L
C-C-0.597300	0	-5.688826	-5.579659	0.946394	L
O-O--0.567900	0	-5.075410	-6.355954	1.688505	L
C-CT-0.211700	0	-6.028732	-7.045867	-1.003382	L
O-OH--0.654600	-1	-7.281016	-7.354361	-0.405313	L
H-H-0.271900	0	-3.658938	-6.098452	-1.461363	L
H-H1-0.084300	0	-6.248247	-4.930048	-1.010514	L
H-H1-0.035200	0	-5.292863	-7.785628	-0.686007	L
H-H1-0.035200	0	-6.117665	-7.079323	-2.089269	L
H-HO-0.427500	-1	-7.538794	-8.227689	-0.710508	L
N-N--0.415700	0	-6.654845	-4.769882	1.373711	L
C-CT-0.033700	-1	-7.155791	-4.670810	2.733513	L
C-C-0.597300	0	-7.529433	-5.990453	3.413595	L
O-O--0.567900	0	-7.507860	-6.077362	4.639784	L

C-CT--0.182500	0	-8.334323	-3.696720	2.730952	L	
H-H-0.271900	0	-7.098457	-4.177333	0.678321	L	
H-H1-0.082300	0	-6.366199	-4.230811	3.341389	L	
H-HC-0.060300	0	-8.032271	-2.737382	2.306558	L	
H-HC-0.060300	0	-8.676038	-3.543039	3.749952	L	
H-HC-0.060300	0	-9.154180	-4.112789	2.151478	L	
N-N--0.415700	0	-7.886613	-7.009578	2.631694	L	
C-CT--0.025200	-1	-8.280719	-8.315251	3.145686	L	
C-C-0.597300	0	-7.312023	-9.451615	2.849455	L	
O-O--0.567900	0	-7.709087	-10.614642	2.887296	L	
H-H-0.271900	0	-7.840362	-6.854480	1.630826	L	
H-H1-0.069800	0	-9.241923	-8.579845	2.706303	L	
H-H1-0.069800	0	-8.419349	-8.275572	4.226339	L	
N-N--0.415700	0	-6.043442	-9.139293	2.590362	L	
C-CT--0.024900	-1	-4.964755	-10.125258	2.624980	L	
C-C-0.597300	0	-4.276927	-10.082585	3.983689	L	
O-O--0.567900	0	-3.966584	-9.005485	4.501855	L	
C-CT-0.211700	0	-3.961155	-9.828512	1.505283	L	
O-OH--0.654600	-1	-3.087105	-10.928920	1.307566	L	
H-H-0.271900	0	-5.796550	-8.153375	2.579493	L	
H-H1-0.084300	0	-5.369463	-11.123198	2.455931	L	
H-H1-0.035200	0	-3.388530	-8.930988	1.744877	L	
H-H1-0.035200	0	-4.510344	-9.653835	0.579245	L	
H-HO-0.427500	-1	-2.547170	-10.742393	0.535990	L	
N-N--0.415700	0	-4.069200	-11.259060	4.574001	L	
C-CT--0.149000	-1	-3.525618	-11.403375	5.913126	L	
H-H-0.271900	0	-4.342742	-12.079142	4.056593	L	
H-H1-0.097600	0	-3.302657	-12.449781	6.121220	L	
H-H1-0.097600	0	-4.245326	-11.034463	6.647230	L	
H-H1-0.097600	0	-2.608781	-10.816505	6.007922	L	
C-CT--0.366200	-1	-12.142925	5.076131	-0.660020	L	
C-C-0.597200	0	-10.713639	5.260173	-0.222269	L	
O-O--0.567900	0	-10.399285	4.995557	0.932786	L	
H-HC-0.112300	0	-12.535643	6.027132	-1.012875	L	
H-HC-0.112300	0	-12.728440	4.728855	0.189561	L	
H-HC-0.112300	0	-12.177151	4.331398	-1.453078	L	
N-N--0.415700	0	-9.864728	5.703639	-1.145446	L	
C-CT--0.025200	-1	-8.431443	5.866603	-0.917582	L	
C-C-0.597300	0	-7.612622	5.793250	-2.181824	L	
O-O--0.567900	0	-8.113229	6.141159	-3.258379	L	
H-H-0.271900	0	-10.175576	5.866155	-2.090784	L	
H-H1-0.069800	0	-8.111903	5.058925	-0.268488	L	
H-H1-0.069800	0	-8.232454	6.816711	-0.423901	L	
N-N--0.415700	0	-6.364716	5.350987	-2.015628	L	H-H1 48
C-CT--0.530209	-1	-5.348815	5.286983	-3.063668	H	
C-C-0.568093	0	-4.652401	3.939586	-2.969479	H	
O-O--0.528105	0	-4.671957	3.285030	-1.922310	H	
C-CT-0.298500	0	-4.377620	6.487915	-2.971506	L	H-H1 48
C-CT--0.319200	0	-5.105529	7.806832	-3.243755	L	
C-CT--0.319200	0	-3.670164	6.571933	-1.616443	L	
H-H-0.271900	0	-6.074705	5.024099	-1.096360	L	
H-H1-0.184096	0	-5.864416	5.376796	-4.027486	H	
H-HC--0.029700	0	-3.617595	6.378145	-3.743237	L	
H-HC-0.079100	0	-5.814636	8.013910	-2.444121	L	

H-HC-0.079100	0	-5.637739	7.738374	-4.191185	L	
H-HC-0.079100	0	-4.377453	8.615123	-3.296188	L	
H-HC-0.079100	0	-4.391059	6.691753	-0.809722	L	
H-HC-0.079100	0	-2.978566	7.411757	-1.610659	L	
H-HC-0.079100	0	-3.103998	5.654776	-1.440940	L	
N-N--0.610103	0	-3.985505	3.543508	-4.077772	H	
C-CT--0.314060	-1	-4.238673	3.989440	-5.438339	H	
C-C-0.597300	0	-5.350597	3.165314	-6.079558	L	H-H1 50
O-O--0.567900	0	-5.476149	1.958680	-5.869277	L	
C-CT--0.110200	0	-2.938036	3.887910	-6.273237	L	H-H1 50
C-CT-0.353100	0	-1.930601	5.040143	-6.043704	L	
C-CT--0.412100	0	-1.072729	4.901921	-4.785537	L	
C-CT--0.412100	0	-0.965729	5.126237	-7.231338	L	
H-H-0.398207	0	-3.385830	2.728105	-3.952816	H	
H-H1-0.165130	0	-4.547329	5.034741	-5.447657	H	
H-HC-0.045700	0	-3.225275	3.907101	-7.324525	L	
H-HC-0.045700	0	-2.449824	2.931212	-6.089576	L	
H-HC--0.036100	0	-2.477126	5.982734	-5.996473	L	
H-HC-0.100000	0	-0.482115	3.989017	-4.831778	L	
H-HC-0.100000	0	-1.711466	4.845604	-3.903381	L	
H-HC-0.100000	0	-0.415219	5.762481	-4.676624	L	
H-HC-0.100000	0	-0.401455	4.198137	-7.312939	L	
H-HC-0.100000	0	-0.274342	5.954560	-7.078311	L	
H-HC-0.100000	0	-1.524441	5.297328	-8.149570	L	
N-N--0.415700	0	-6.141683	3.815611	-6.927356	L	
C-CT--0.038900	-1	-7.331813	3.216455	-7.542776	L	
C-C-0.597300	0	-7.088156	1.879052	-8.231963	L	
O-O--0.567900	0	-7.971083	1.024476	-8.276970	L	
C-CT-0.365400	-1	-7.969973	4.138915	-8.605690	L	
O-OH--0.676100	-1	-7.907836	5.496953	-8.160357	L	
C-CT--0.243800	0	-9.449010	3.849788	-8.862544	L	
H-H-0.271900	0	-6.020839	4.814311	-7.008169	L	
H-H1-0.100700	0	-8.069761	3.077145	-6.759720	L	
H-H1-0.004300	0	-7.415844	4.060948	-9.542104	L	
H-HC-0.064200	0	-9.565975	2.845786	-9.269962	L	
H-HC-0.064200	0	-9.843527	4.563481	-9.585556	L	
H-HC-0.064200	0	-10.011130	3.922065	-7.930671	L	
H-HO-0.410200	-1	-8.805989	5.832267	-8.124735	L	
N-N--0.415700	0	-5.911502	1.741057	-8.839753	L	
C-CT--0.025200	-1	-5.526655	0.583175	-9.632946	L	
C-C-0.597300	0	-4.631316	-0.421721	-8.937508	L	
O-O--0.567900	0	-4.134475	-1.324641	-9.611631	L	
H-H-0.271900	0	-5.222999	2.452889	-8.646193	L	
H-H1-0.069800	0	-4.996347	0.929166	-10.518947	L	
H-H1-0.069800	0	-6.415585	0.049446	-9.968909	L	
N-N--0.415700	0	-4.419307	-0.247927	-7.628110	L	H-H1 54
C-CT--0.539097	-1	-3.481041	-1.022699	-6.827632	H	
C-C-0.572106	0	-4.213314	-1.478635	-5.565172	H	
O-O--0.533001	0	-4.932816	-2.472652	-5.630452	H	
C-CT-0.130300	0	-2.139645	-0.264766	-6.626450	L	H-H1 54
C-CT--0.043000	0	-1.535644	0.250327	-7.954830	L	
C-CT--0.320400	0	-1.167634	-1.236799	-5.935208	L	
C-CT--0.066000	0	-0.214429	1.015519	-7.799776	L	
H-H-0.271900	0	-4.883708	0.523824	-7.157108	L	

```
H-H1-0.180646    0   -3.259758   -1.940828   -7.381912 H
H-HC-0.018700    0   -2.307816    0.592743   -5.975778 L
H-HC-0.023600    0   -2.240436    0.939809   -8.418121 L
H-HC-0.023600    0   -1.382820   -0.589442   -8.633781 L
H-HC-0.088200    0   -0.971525   -2.094822   -6.576415 L
H-HC-0.088200    0   -1.585621   -1.584317   -4.992018 L
H-HC-0.088200    0   -0.231968   -0.737793   -5.696209 L
H-HC-0.018600    0   -0.329597    1.801578   -7.054208 L
H-HC-0.018600    0    0.053398    1.468138   -8.754478 L
H-HC-0.018600    0    0.587021    0.340877   -7.500237 L
N-N--0.611145    0   -4.071275   -0.721380   -4.443083 H
C-CT--0.109296  -1   -4.642328   -1.117342   -3.164407 H
C-C-0.597300     0   -6.169666   -1.141901   -3.176638 L H-H1 56
O-O--0.567900    0   -6.800440   -2.079834   -2.673180 L
C-CT--0.388038   0   -4.114851   -0.301501   -2.003464 H
H-H-0.369964     0   -3.433024    0.064876   -4.419522 H
H-H1-0.178541    0   -4.389645   -2.168085   -2.980189 H
H-H1-0.157964    0   -4.732685   -0.554859   -1.136687 H
H-H1-0.179833    0   -4.175224    0.777164   -2.169036 H
H-HS-0.319500    0   -2.152932    0.187439   -0.788331 H
N-N--0.415700    0   -6.761244   -0.143447   -3.819044 L
C-CT--0.087500  -1   -8.199731   -0.078813   -4.059341 L
C-C-0.597300     0   -8.688457   -1.229006   -4.926745 L
O-O--0.567900    0   -9.634900   -1.922364   -4.553730 L
C-CT-0.298500    0   -8.596433    1.284604   -4.645174 L
C-CT--0.319200   0  -10.113769    1.416249   -4.835717 L
C-CT--0.319200   0   -8.144456    2.459531   -3.763576 L
H-H-0.271900     0   -6.126201    0.549222   -4.208956 L
H-H1-0.096900    0   -8.700526   -0.177995   -3.098850 L
H-HC--0.029700   0   -8.121457    1.369371   -5.614229 L
H-HC-0.079100    0  -10.620514    1.270308   -3.881512 L
H-HC-0.079100    0  -10.468235    0.674343   -5.550976 L
H-HC-0.079100    0  -10.350491    2.406429   -5.224018 L
H-HC-0.079100    0   -8.594228    2.393484   -2.774833 L
H-HC-0.079100    0   -8.421379    3.407846   -4.223258 L
H-HC-0.079100    0   -7.059776    2.456969   -3.648329 L
N-N--0.415700    0   -8.051667   -1.443677   -6.076582 L
C-CT--0.025200  -1   -8.412593   -2.501546   -7.017126 L
C-C-0.597300     0   -8.322060   -3.902441   -6.425444 L
O-O--0.567900    0   -9.219297   -4.719642   -6.621607 L
H-H-0.271900     0   -7.252001   -0.856183   -6.276828 L
H-H1-0.069800    0   -7.746510   -2.451335   -7.878199 L
H-H1-0.069800    0   -9.433900   -2.339621   -7.361251 L
N-N--0.415700    0   -7.240031   -4.175946   -5.698749 L
C-CT--0.025200  -1   -7.017002   -5.440349   -5.008588 L
C-C-0.597300     0   -7.949227   -5.700400   -3.836520 L
O-O--0.567900    0   -8.285622   -6.851742   -3.564713 L
H-H-0.271900     0   -6.525609   -3.452579   -5.629619 L
H-H1-0.069800    0   -5.993808   -5.457113   -4.634793 L
H-H1-0.069800    0   -7.134268   -6.257785   -5.720128 L
N-N--0.415700    0   -8.335015   -4.642508   -3.127139 L
C-CT--0.038900  -1   -9.384673   -4.696694   -2.107357 L
C-C-0.597300     0  -10.756054   -4.989685   -2.706826 L
O-O--0.567900    0  -11.499286   -5.823417   -2.188690 L
```

C-CT-0.365400	0	-9.418892	-3.390855	-1.298571	L
O-OH--0.676100	0	-8.179909	-3.219911	-0.648625	L
C-CT--0.243800	0	-10.501744	-3.395985	-0.220076	L
H-H-0.271900	0	-7.942291	-3.739821	-3.372722	L
H-H1-0.100700	0	-9.156827	-5.509140	-1.417505	L
H-H1-0.004300	0	-9.587981	-2.543260	-1.962886	L
H-HC-0.064200	0	-11.483466	-3.291530	-0.680707	L
H-HC-0.064200	0	-10.344374	-2.561746	0.463166	L
H-HC-0.064200	0	-10.466816	-4.332542	0.334671	L
H-HO-0.410200	0	-7.592923	-2.766121	-1.287492	L
N-N--0.415700	0	-11.106611	-4.284941	-3.783918	L
C-CT--0.149000	-1	-12.339316	-4.479065	-4.519224	L
H-H-0.271900	0	-10.440416	-3.601914	-4.134160	L
H-H1-0.097600	0	-12.416000	-3.727745	-5.306159	L
H-H1-0.097600	0	-13.191131	-4.393008	-3.843274	L
H-H1-0.097600	0	-12.336735	-5.472571	-4.970682	L
N-N2--0.511415	0	2.009814	0.005678	-1.668114	H
C-CA-0.057801	0	0.852721	0.678971	-1.573849	H
C-CA-0.063496	0	0.232767	1.268640	-2.675571	H
C-CA--0.109122	0	0.872741	1.180485	-3.913742	H
C-CA--0.164490	0	2.089214	0.520737	-4.005945	H
C-CA-0.098392	0	2.617811	-0.065810	-2.862648	H
C-C-0.541263	0	-1.139631	1.897826	-2.632519	H
O-O--0.574884	0	-1.752690	1.899129	-3.713989	H
H-H4-0.220889	0	0.418517	0.738153	-0.580894	H
H-HA-0.160434	0	0.391040	1.620460	-4.778154	H
H-HA-0.142191	0	2.621711	0.453176	-4.946599	H
H-H4-0.174502	0	3.545731	-0.626441	-2.887537	H
H-H-0.353801	0	-1.493707	1.973623	-0.399163	H
Zn-ZN-0.974537	0	2.843295	-1.105754	-0.010546	H
O-OW--0.834000	0	-2.127490	-7.250029	3.506267	L
H-HW-0.417000	0	-2.560481	-6.671440	2.858747	L
H-HW-0.417000	0	-2.854821	-7.790818	3.855253	L
O-OW--0.783903	0	1.312872	-2.584515	-0.136233	H
H-HW-0.401253	0	0.929464	-2.477829	0.768936	H
H-HW-0.424111	0	1.923851	-3.364638	-0.181093	H
O-OW--0.834000	0	-3.689329	-8.439219	7.246963	L
H-HW-0.417000	0	-3.747179	-7.494679	7.412077	L
H-HW-0.417000	0	-3.812002	-8.517858	6.288665	L
O-OW--0.834000	0	7.150639	-0.013483	1.778573	L
H-HW-0.417000	0	6.848588	0.407860	2.603845	L
H-HW-0.417000	0	6.357825	0.066349	1.224060	L
O-OW--0.834000	0	7.059743	-4.944573	4.764192	L
H-HW-0.417000	0	6.109752	-5.004555	4.992968	L
H-HW-0.417000	0	7.261762	-5.820138	4.432173	L
O-OW--0.834000	0	8.638001	-0.919135	-0.254848	L
H-HW-0.417000	0	8.153855	-0.548423	0.511173	L
H-HW-0.417000	0	8.840709	-0.129414	-0.776990	L
O-OW--0.834000	0	12.649258	-1.083193	-2.004184	L
H-HW-0.417000	0	11.794612	-0.832580	-2.395239	L
H-HW-0.417000	0	12.652002	-2.046712	-2.111778	L
O-OW--0.834000	0	8.295706	-3.288593	0.986870	L
H-HW-0.417000	0	8.508143	-2.519695	0.417496	L
H-HW-0.417000	0	7.562681	-2.951897	1.512550	L

O-OW--0.834000	0	4.367125	-4.899308	5.157620	L
H-HW-0.417000	0	4.094675	-4.514794	6.013727	L
H-HW-0.417000	0	4.066518	-4.232884	4.529038	L
S-SH--0.326142	0	-2.380829	-0.807949	-1.657839	H
O-OW--0.834000	0	-1.590852	2.398068	-1.534032	H

PC$_c$

C-CT--0.366200	-1	-10.314696	3.925855	4.004262	L
C-C-0.597200	0	-8.984570	3.273910	3.762220	L
O-O--0.567900	0	-8.411929	2.701503	4.682570	L
H-HC-0.112300	0	-11.056666	3.465756	3.354682	L
H-HC-0.112300	0	-10.234093	4.987619	3.777846	L
H-HC-0.112300	0	-10.595598	3.787701	5.045900	L
N-N--0.415700	0	-8.519635	3.364285	2.523318	L
C-CT--0.059700	-1	-7.346516	2.651521	2.010554	L
C-C-0.597300	0	-6.198729	3.631529	1.827865	L
O-O--0.567900	0	-6.358893	4.638785	1.144625	L
C-CT-0.130300	0	-7.697079	1.909489	0.696418	L
C-CT--0.043000	0	-8.890821	0.941171	0.899451	L
C-CT--0.320400	0	-6.476809	1.126772	0.182323	L
C-CT--0.066000	0	-9.415267	0.305674	-0.393095	L
H-H-0.271900	0	-9.052241	3.930491	1.871236	L
H-H1-0.086900	0	-7.037281	1.901710	2.738629	L
H-HC-0.018700	0	-7.973997	2.649897	-0.056325	L
H-HC-0.023600	0	-9.729718	1.479789	1.338403	L
H-HC-0.023600	0	-8.603016	0.148705	1.591507	L
H-HC-0.088200	0	-6.185629	0.354516	0.894596	L
H-HC-0.088200	0	-5.631147	1.796726	0.021711	L
H-HC-0.088200	0	-6.698102	0.672480	-0.781367	L
H-HC-0.018600	0	-9.640789	1.081625	-1.123543	L
H-HC-0.018600	0	-10.329354	-0.245958	-0.176475	L
H-HC-0.018600	0	-8.680127	-0.386467	-0.800683	L
N-N--0.516300	0	-5.080986	3.341544	2.489219	L
C-CT-0.038100	-1	-3.779822	3.984909	2.372298	L H-HC 30
C-C-0.536600	0	-3.710707	5.509867	2.152819	L
O-O--0.581900	0	-2.726157	6.045514	1.644767	L
C-CT--0.547145	0	-3.006901	3.238743	1.265443	H
C-C-0.532653	0	-2.405048	1.951394	1.733620	H
O-O2--0.588507	0	-2.599088	1.431412	2.820036	H
O-O2--0.625559	0	-1.568256	1.347463	0.868982	H
H-H-0.293600	0	-5.054975	2.456347	2.978475	L
H-H1-0.088000	0	-3.245600	3.819762	3.308530	L
H-HC-0.128435	0	-2.163649	3.851511	0.913970	H
H-HC-0.183556	0	-3.621811	3.110157	0.365286	H
N-N--0.415700	0	-4.689461	6.262044	2.633203	L
C-CT--0.025200	-1	-4.654516	7.704026	2.487000	L
C-C-0.597300	0	-3.759086	8.351479	3.529132	L
O-O--0.567900	0	-4.236979	9.021007	4.446487	L
H-H-0.271900	0	-5.526003	5.768961	2.903441	L
H-H1-0.069800	0	-5.663057	8.096986	2.605877	L
H-H1-0.069800	0	-4.299955	7.974414	1.490915	L
N-N--0.415700	0	-2.455502	8.136185	3.386609	L
C-CT--0.038900	-1	-1.432224	8.568936	4.326851	L
C-C-0.597300	0	-0.517667	9.642804	3.726413	L

O-O--0.567900	0	-0.608121	9.999850	2.550696	L
C-CT-0.365400	0	-0.595293	7.416847	4.926937	L
O-OH--0.676100	0	0.604434	7.242143	4.215589	L
C-CT--0.243800	0	-1.310331	6.074541	5.041126	L
H-H-0.271900	0	-2.192577	7.536849	2.606398	L
H-H1-0.100700	0	-1.938017	9.039012	5.168269	L
H-H1-0.004300	0	-0.316537	7.719323	5.936909	L
H-HC-0.064200	0	-2.254252	6.204854	5.568335	L
H-HC-0.064200	0	-0.681020	5.382591	5.595815	L
H-HC-0.064200	0	-1.498232	5.657947	4.052107	L
H-HO-0.410200	0	1.091930	6.486591	4.609506	L
N-N--0.415700	0	0.393978	10.162233	4.552617	L
C-CT--0.025200	-1	1.436657	11.094353	4.137041	L
C-C-0.597300	0	2.338875	10.602130	3.014493	L
O-O--0.567900	0	2.676014	11.376888	2.124192	L
H-H-0.271900	0	0.450381	9.765784	5.475406	L
H-H1-0.069800	0	2.070749	11.328860	4.990873	L
H-H1-0.069800	0	0.966385	12.018215	3.799663	L
N-N--0.516300	0	2.733200	9.332578	3.073099	L
C-CT-0.038100	-1	3.642714	8.732345	2.098039	L
C-C-0.536600	0	3.014087	8.439802	0.748915	L
O-O--0.581900	0	3.747584	8.140537	-0.187035	L
C-CT--0.030300	0	4.225879	7.419753	2.652598	L
C-C-0.799400	-1	5.572270	7.621749	3.329710	L
O-O2--0.801400	-1	6.153035	8.725497	3.245481	L
O-O2--0.801400	-1	6.031464	6.669880	3.993266	L
H-H-0.293600	0	2.357475	8.727921	3.789867	L
H-H1-0.088000	0	4.445788	9.438265	1.883678	L
H-HC--0.012200	0	4.382183	6.702831	1.844816	L
H-HC--0.012200	0	3.522369	6.965266	3.353412	L
N-N--0.415700	0	1.692538	8.528059	0.633958	L
C-CT--0.002400	-1	0.973857	8.316082	-0.615318	L
C-C-0.597300	0	0.377670	9.607251	-1.177799	L
O-O--0.567900	0	0.361932	9.813898	-2.391325	L
C-CT--0.034300	0	-0.091479	7.227712	-0.431446	L
C-CA-0.011800	0	0.435552	5.911661	0.116796	L
C-CA--0.125600	0	1.083019	4.984942	-0.720608	L
C-CA--0.125600	0	0.247132	5.598854	1.472873	L
C-CA--0.170400	0	1.520139	3.753298	-0.197895	L
C-CA--0.170400	0	0.659394	4.361806	1.992302	L
C-CA--0.107200	0	1.290426	3.434611	1.150315	L
H-H-0.271900	0	1.171997	8.729992	1.474881	L
H-H1-0.097800	0	1.663303	7.945333	-1.372996	L
H-HC-0.029500	0	-0.556902	7.031394	-1.398219	L
H-HC-0.029500	0	-0.870974	7.603873	0.232881	L
H-HA-0.133000	0	1.208344	5.202106	-1.770155	L
H-HA-0.133000	0	-0.271896	6.299373	2.101200	L
H-HA-0.143000	0	1.988909	3.023784	-0.837702	L
H-HA-0.143000	0	0.451483	4.111163	3.021142	L
H-HA-0.129700	0	1.563662	2.463194	1.528780	L
N-N--0.415700	0	-0.077573	10.505419	-0.301558	L
C-CT--0.149000	-1	-0.473218	11.850540	-0.667661	L
H-H-0.271900	0	-0.072411	10.246124	0.679710	L
H-H1-0.097600	0	-0.862369	12.367631	0.209860	L

H-H1-0.097600	0	-1.244106	11.807640	-1.438900	L
H-H1-0.097600	0	0.392075	12.390668	-1.054726	L
C-CT--0.366200	-1	7.983813	8.429589	-0.178837	L
C-C-0.597200	0	7.523528	7.630818	-1.375743	L
O-O--0.567900	0	8.345620	7.283186	-2.220042	L
H-HC-0.112300	0	7.526662	9.416295	-0.204551	L
H-HC-0.112300	0	9.067403	8.518364	-0.201842	L
H-HC-0.112300	0	7.684575	7.915029	0.735187	L
N-N--0.415700	0	6.214295	7.381665	-1.471171	L
C-CT--0.051800	-1	5.562653	6.847958	-2.675102	L
C-C-0.597300	0	4.922469	7.971849	-3.489995	L
O-O--0.567900	0	5.306926	8.236971	-4.629399	L
C-CT--0.110200	0	4.618890	5.673738	-2.332676	L
C-CT-0.353100	0	4.572740	4.613393	-3.453280	L
C-CT--0.412100	0	3.798219	3.378447	-3.008726	L
C-CT--0.412100	0	3.913791	5.102795	-4.745387	L
H-H-0.271900	0	5.609292	7.703050	-0.722598	L
H-H1-0.092200	0	6.349970	6.441759	-3.311295	L
H-HC-0.045700	0	3.614291	6.037704	-2.115522	L
H-HC-0.045700	0	4.993709	5.183720	-1.433869	L
H-HC--0.036100	0	5.592042	4.297180	-3.679722	L
H-HC-0.100000	0	2.737029	3.610596	-2.924491	L
H-HC-0.100000	0	4.166367	3.028725	-2.046019	L
H-HC-0.100000	0	3.932346	2.583274	-3.738867	L
H-HC-0.100000	0	2.932635	5.525392	-4.525199	L
H-HC-0.100000	0	3.797330	4.272631	-5.441091	L
H-HC-0.100000	0	4.536263	5.857848	-5.220619	L
N-N--0.415700	0	3.976494	8.657697	-2.862379	L
C-CT--0.149000	-1	3.356831	9.877457	-3.311466	L
H-H-0.271900	0	3.777254	8.345857	-1.919049	L
H-H1-0.097600	0	4.070049	10.471463	-3.884341	L
H-H1-0.097600	0	3.014700	10.453579	-2.450480	L
H-H1-0.097600	0	2.498420	9.639688	-3.941953	L
C-CT--0.366200	-1	-8.731032	2.391495	8.116861	L
C-C-0.597200	0	-7.860282	1.179798	7.941998	L
O-O--0.567900	0	-7.544583	0.499550	8.911652	L
H-HC-0.112300	0	-9.647749	2.252576	7.547035	L
H-HC-0.112300	0	-8.197876	3.266633	7.748485	L
H-HC-0.112300	0	-8.962568	2.514735	9.172666	L
N-N--0.415700	0	-7.467044	0.925943	6.702092	L
C-CT--0.038900	-1	-6.669290	-0.231302	6.292482	L
C-C-0.597300	0	-5.399518	0.245032	5.610471	L
O-O--0.567900	0	-5.430091	0.653226	4.450007	L
C-CT-0.365400	0	-7.478876	-1.198188	5.414737	L
O-OH--0.676100	0	-8.033506	-0.536140	4.303959	L
C-CT--0.243800	0	-8.628836	-1.816370	6.215482	L
H-H-0.271900	0	-7.794187	1.546050	5.964894	L
H-H1-0.100700	0	-6.371304	-0.792974	7.177480	L
H-H1-0.004300	0	-6.822048	-1.995535	5.064876	L
H-HC-0.064200	0	-8.231579	-2.347538	7.080055	L
H-HC-0.064200	0	-9.183552	-2.515246	5.595497	L
H-HC-0.064200	0	-9.314767	-1.039018	6.550411	L
H-HO-0.410200	0	-7.338506	0.040146	3.954203	L
N-N--0.415700	0	-4.313199	0.251997	6.387384	L

C-CT--0.059700	-1	-3.115619	1.062511	6.155408	L	
C-C-0.597300	0	-1.871737	0.182897	6.071249	L	
O-O--0.567900	0	-1.609686	-0.612974	6.978427	L	
C-CT-0.130300	0	-2.983074	2.151848	7.244640	L	
C-CT--0.043000	0	-4.241131	3.042114	7.403965	L	
C-CT--0.320400	0	-1.750872	3.028852	6.978229	L	
C-CT--0.066000	0	-4.687036	3.808067	6.149068	L	
H-H-0.271900	0	-4.387678	-0.166083	7.300334	L	
H-H1-0.086900	0	-3.221129	1.570863	5.196962	L	
H-HC-0.018700	0	-2.825616	1.652562	8.202545	L	
H-HC-0.023600	0	-4.051873	3.769340	8.194307	L	
H-HC-0.023600	0	-5.073589	2.425104	7.741468	L	
H-HC-0.088200	0	-1.762414	3.375048	5.944462	L	
H-HC-0.088200	0	-0.844272	2.444521	7.135489	L	
H-HC-0.088200	0	-1.733930	3.881860	7.655262	L	
H-HC-0.018600	0	-3.893194	4.466843	5.801319	L	
H-HC-0.018600	0	-5.563131	4.411044	6.388018	L	
H-HC-0.018600	0	-4.953481	3.115301	5.353825	L	
N-N--0.516300	0	-1.127843	0.347773	4.978746	L	
C-CT-0.038100	-1	0.180615	-0.228206	4.724692	L	H-HC 17
C-C-0.536600	0	1.096586	-0.072818	5.945257	L	
O-O--0.581900	0	1.294265	1.018069	6.474726	L	
C-CT--0.520019	0	0.733809	0.419458	3.441941	H	
C-C-0.636404	0	0.912159	-0.506728	2.244978	H	
O-O2--0.608036	0	0.256294	-1.590468	2.150141	H	
O-O2--0.608131	0	1.751692	-0.083893	1.392272	H	
H-H-0.293600	0	-1.518048	0.926276	4.239272	L	
H-H1-0.088000	0	0.042117	-1.297207	4.556308	L	
H-HC-0.132300	0	1.718801	0.841005	3.659487	H	
H-HC-0.188993	0	0.105044	1.260293	3.135078	H	
N-N--0.415700	0	1.654280	-1.181297	6.399900	L	
C-CT-0.033700	-1	2.563204	-1.224755	7.522133	L	
C-C-0.597300	0	3.782959	-2.046612	7.121546	L	
O-O--0.567900	0	3.831472	-3.271918	7.277993	L	
C-CT--0.182500	0	1.815207	-1.778453	8.743188	L	
H-H-0.271900	0	1.361862	-2.059128	5.985283	L	
H-H1-0.082300	0	2.908976	-0.218509	7.766719	L	
H-HC-0.060300	0	0.976617	-1.123095	8.981925	L	
H-HC-0.060300	0	2.491726	-1.825187	9.596222	L	
H-HC-0.060300	0	1.437423	-2.778819	8.525792	L	
N-N--0.415700	0	4.755382	-1.352702	6.542666	L	
C-CT-0.018800	-1	5.959299	-1.937039	5.996311	L	H-HC 20
C-C-0.597300	0	7.158798	-1.783227	6.940355	L	
O-O--0.567900	0	7.206841	-0.930851	7.829765	L	
C-CT--0.517456	0	6.204910	-1.308400	4.622279	H	
C-CM-0.287351	0	5.311747	-1.774684	3.531149	H	
N-N2--0.586994	0	5.428686	-3.032075	2.955300	H	
C-CM--0.020338	0	4.331775	-1.154521	2.806259	H	
C-CM-0.242458	0	4.549682	-3.110341	1.920729	H	
N-N2--0.505070	0	3.873738	-1.982739	1.801696	H	
H-H-0.271900	0	4.651010	-0.343622	6.480652	L	
H-H1-0.088100	0	5.812741	-3.004153	5.862071	L	
H-HC-0.166355	0	7.253364	-1.492575	4.345324	H	
H-HC-0.174481	0	6.112490	-0.227688	4.756454	H	

H-H-0.335795	0	6.080263	-3.751744	3.259542	H	
H-H4-0.152777	0	3.952423	-0.151411	2.930724	H	
H-H5-0.209475	0	4.434626	-3.974926	1.281578	H	
N-N--0.415700	0	8.162877	-2.623064	6.696011	L	
C-CT--0.149000	-1	9.429500	-2.554646	7.391566	L	
H-H-0.271900	0	8.010718	-3.303232	5.957885	L	
H-H1-0.097600	0	10.102435	-3.325326	7.014546	L	
H-H1-0.097600	0	9.267447	-2.702244	8.460676	L	
H-H1-0.097600	0	9.878795	-1.572150	7.235711	L	
C-CT--0.366200	-1	8.353880	-9.389071	0.947629	L	
C-C-0.597200	0	8.734023	-9.166893	-0.503912	L	
O-O--0.567900	0	9.770227	-8.567777	-0.764670	L	
H-HC-0.112300	0	8.271533	-10.454330	1.149884	L	
H-HC-0.112300	0	7.415176	-8.880644	1.160250	L	
H-HC-0.112300	0	9.132589	-8.959068	1.577440	L	
N-N--0.254800	0	7.913994	-9.624362	-1.466711	L	
C-CT--0.026600	-1	8.156673	-9.390118	-2.892474	L	
C-C-0.589600	0	8.011646	-7.917509	-3.276733	L	
O-O--0.574800	0	8.726594	-7.423460	-4.145489	L	
C-CT--0.007000	0	7.146199	-10.281457	-3.622303	L	
C-CT-0.018900	0	5.986328	-10.401000	-2.632369	L	
C-CT-0.019200	0	6.679293	-10.372117	-1.269223	L	
H-H1-0.064100	0	9.167929	-9.704628	-3.153556	L	
H-HC-0.025300	0	7.586469	-11.266666	-3.783220	L	
H-HC-0.025300	0	6.826176	-9.851749	-4.572843	L	
H-HC-0.021300	0	5.422653	-11.322853	-2.778744	L	
H-HC-0.021300	0	5.330213	-9.534448	-2.728240	L	
H-H1-0.039100	0	6.033472	-9.893791	-0.531468	L	
H-H1-0.039100	0	6.919750	-11.389031	-0.956272	L	
N-N--0.516300	0	7.087448	-7.219547	-2.616234	L	
C-CT-0.039700	-1	6.833096	-5.793471	-2.790651	L	
C-C-0.536600	0	8.091472	-4.926928	-2.662883	L	
O-O--0.581900	0	8.266424	-3.946178	-3.389508	L	
C-CT-0.056000	0	5.733879	-5.358374	-1.814922	L	H-HC 24
C-CT--0.515956	0	5.193972	-3.977300	-2.196162	H	
C-C-0.587828	0	4.112897	-3.435668	-1.269160	H	
O-O2--0.600319	0	4.088710	-2.154965	-1.203282	H	
O-O2--0.589127	0	3.333676	-4.217878	-0.676648	H	
H-H-0.293600	0	6.551592	-7.695267	-1.909577	L	
H-H1-0.110500	0	6.456348	-5.641972	-3.802592	L	
H-HC--0.017300	0	6.131101	-5.333465	-0.799036	L	
H-HC--0.017300	0	4.914467	-6.077926	-1.857186	L	
H-HC-0.167759	0	4.775258	-4.037461	-3.208050	H	
H-HC-0.135230	0	6.014314	-3.252460	-2.224790	H	
N-N--0.415700	0	9.000909	-5.330015	-1.777975	L	
C-CT--0.024900	-1	10.241278	-4.630775	-1.463564	L	
C-C-0.597300	0	11.262452	-4.521578	-2.588956	L	
O-O--0.567900	0	12.242167	-3.787739	-2.457356	L	
C-CT-0.211700	0	10.882025	-5.267877	-0.224375	L	
O-OH--0.654600	0	9.926154	-5.456137	0.808661	L	
H-H-0.271900	0	8.788942	-6.135859	-1.206254	L	
H-H1-0.084300	0	9.976299	-3.606402	-1.197146	L	
H-H1-0.035200	0	11.682658	-4.621329	0.137851	L	
H-H1-0.035200	0	11.306130	-6.236695	-0.492543	L	

H-HO-0.427500	0	9.380587	-4.642018	0.891619	L
N-N--0.415700	0	11.024011	-5.209280	-3.703816	L
C-CT-0.033700	-1	11.775007	-5.048703	-4.942176	L
C-C-0.597300	0	10.945634	-4.489446	-6.102724	L
O-O--0.567900	0	11.384858	-4.493252	-7.251929	L
C-CT--0.182500	0	12.397027	-6.412310	-5.276549	L
H-H-0.271900	0	10.210539	-5.814314	-3.710205	L
H-H1-0.082300	0	12.593228	-4.344006	-4.787671	L
H-HC-0.060300	0	13.011885	-6.752536	-4.442583	L
H-HC-0.060300	0	13.023821	-6.323326	-6.164508	L
H-HC-0.060300	0	11.608270	-7.142012	-5.466598	L
N-N--0.415700	0	9.761688	-3.979455	-5.781289	L
C-CT--0.025200	-1	8.861290	-3.295239	-6.686381	L
C-C-0.597300	0	8.818699	-1.790673	-6.468867	L
O-O--0.567900	0	8.837769	-1.020002	-7.425823	L
H-H-0.271900	0	9.478338	-4.040622	-4.809537	L
H-H1-0.069800	0	7.853039	-3.682614	-6.542946	L
H-H1-0.069800	0	9.151031	-3.481401	-7.721004	L
N-N--0.415700	0	8.758696	-1.381831	-5.204484	L
C-CT--0.002400	-1	8.579350	0.003836	-4.787327	L
C-C-0.597300	0	9.686402	0.439670	-3.821453	L
O-O--0.567900	0	10.301381	-0.410438	-3.168916	L
C-CT--0.034300	0	7.183767	0.124760	-4.148991	L
C-CA-0.011800	0	6.055322	-0.160967	-5.123180	L
C-CA--0.125600	0	5.652750	0.824976	-6.044152	L
C-CA--0.125600	0	5.444445	-1.429123	-5.148884	L
C-CA--0.170400	0	4.655868	0.539748	-6.994902	L
C-CA--0.170400	0	4.442306	-1.711559	-6.094003	L
C-CA--0.107200	0	4.053551	-0.730825	-7.023337	L
H-H-0.271900	0	8.759858	-2.087422	-4.477345	L
H-H1-0.097800	0	8.617502	0.652275	-5.662775	L
H-HC-0.029500	0	7.044336	1.126910	-3.744538	L
H-HC-0.029500	0	7.121161	-0.570538	-3.310277	L
H-HA-0.133000	0	6.116520	1.800652	-6.030988	L
H-HA-0.133000	0	5.753656	-2.191941	-4.449490	L
H-HA-0.143000	0	4.349426	1.298991	-7.700377	L
H-HA-0.143000	0	3.968887	-2.682453	-6.105587	L
H-HA-0.129700	0	3.279143	-0.947980	-7.745860	L
N-N--0.254800	0	9.950240	1.752455	-3.686898	L
C-CT--0.026600	-1	10.816741	2.248461	-2.624547	L
C-C-0.589600	0	10.179743	2.011530	-1.246497	L
O-O--0.574800	0	8.983907	1.711750	-1.167003	L
C-CT--0.007000	0	10.984335	3.745354	-2.916934	L
C-CT-0.018900	0	9.669046	4.117231	-3.599447	L
C-CT-0.019200	0	9.350948	2.865503	-4.417208	L
H-H1-0.064100	0	11.783378	1.748612	-2.677057	L
H-HC-0.025300	0	11.809712	3.889484	-3.615604	L
H-HC-0.025300	0	11.145902	4.334550	-2.012908	L
H-HC-0.021300	0	9.771864	5.000840	-4.230099	L
H-HC-0.021300	0	8.893808	4.272711	-2.846432	L
H-H1-0.039100	0	8.271806	2.761817	-4.521211	L
H-H1-0.039100	0	9.815225	2.941625	-5.401253	L
N-N--0.254800	0	10.945829	2.125921	-0.151415	L
C-CT--0.026600	-1	10.392535	2.112428	1.195465	L

C-C-0.589600	0	9.343347	3.198620	1.433393	L	
O-O--0.574800	0	9.611272	4.379507	1.223948	L	
C-CT--0.007000	0	11.592829	2.290022	2.136262	L	
C-CT-0.018900	0	12.777957	1.800606	1.307617	L	
C-CT-0.019200	0	12.396120	2.222496	-0.108278	L	
H-H1-0.064100	0	9.949137	1.131645	1.373295	L	
H-HC-0.025300	0	11.473666	1.708954	3.051288	L	
H-HC-0.025300	0	11.739652	3.344923	2.375284	L	
H-HC-0.021300	0	12.838208	0.712167	1.359895	L	
H-HC-0.021300	0	13.715261	2.256216	1.627832	L	
H-H1-0.039100	0	12.697505	3.256161	-0.283312	L	
H-H1-0.039100	0	12.870180	1.556307	-0.829845	L	
N-N--0.415700	0	8.170748	2.774141	1.894573	L	
C-CT-0.018800	-1	7.015308	3.605076	2.187931	L	H-HC 33
C-C-0.597300	0	6.147366	3.013821	3.296743	L	
O-O--0.567900	0	6.169775	1.805757	3.531368	L	
C-CT--0.515368	0	6.201881	3.728136	0.872130	H	
C-CM-0.287903	0	5.602968	2.451145	0.362952	H	
N-N2--0.582625	0	6.213573	1.665446	-0.601147	H	
C-CM--0.036893	0	4.490794	1.726673	0.722747	H	
C-CM-0.243454	0	5.482198	0.535409	-0.777912	H	
N-N2--0.523998	0	4.432820	0.537572	0.023734	H	
H-H-0.271900	0	8.026963	1.777123	1.966612	L	
H-H1-0.088100	0	7.350561	4.598481	2.501098	L	
H-HC-0.162301	0	6.864077	4.177287	0.117647	H	
H-HC-0.188340	0	5.411464	4.468459	1.047481	H	
H-H-0.339448	0	7.118288	1.868386	-1.026167	H	
H-H4-0.185957	0	3.736133	1.985184	1.449630	H	
H-H5-0.211295	0	5.742950	-0.254475	-1.466719	H	
N-N--0.415700	0	5.367492	3.875907	3.936782	L	
C-CT-0.014300	-1	4.549535	3.599366	5.108690	L	
C-C-0.597300	0	5.202052	2.649744	6.123417	L	
O-O--0.567900	0	4.676622	1.599268	6.496634	L	
C-CT--0.204100	0	3.078639	3.360152	4.742737	L	
C-C-0.713000	0	2.197196	4.202653	5.648141	L	
N-N--0.919100	0	1.700851	3.677022	6.733492	L	
O-O--0.593100	0	1.982279	5.381948	5.429517	L	
H-H-0.271900	0	5.499473	4.862007	3.705143	L	
H-H1-0.104800	0	4.553154	4.559082	5.630901	L	
H-HC-0.079700	0	2.833903	2.307614	4.841009	L	
H-HC-0.079700	0	2.891650	3.671606	3.715948	L	
H-H-0.419600	0	1.069880	4.240291	7.261265	L	
H-H-0.419600	0	1.742226	2.661503	6.818899	L	
N-N--0.415700	0	6.389626	3.081301	6.561268	L	
C-CT--0.149000	-1	7.293103	2.344130	7.423309	L	
H-H-0.271900	0	6.701946	3.953343	6.156921	L	
H-H1-0.097600	0	8.041736	3.012886	7.847524	L	
H-H1-0.097600	0	7.787619	1.559869	6.847012	L	
H-H1-0.097600	0	6.730879	1.867253	8.229298	L	
C-CT--0.366200	-1	-2.256622	-2.718573	9.543564	L	
C-C-0.597200	0	-2.375810	-3.815000	8.520905	L	
O-O--0.567900	0	-2.903254	-4.882372	8.822480	L	
H-HC-0.112300	0	-1.203358	-2.499290	9.707793	L	
H-HC-0.112300	0	-2.717314	-3.046077	10.473059	L	

```
H-HC-0.112300     0   -2.762177   -1.828756    9.172909 L
N-N--0.347900     0   -1.857532   -3.554613    7.323929 L
C-CT--0.240000   -1   -1.749513   -4.526211    6.229351 L
C-C-0.734100      0   -0.337186   -4.565698    5.657762 L
O-O--0.589400     0    0.483783   -3.692623    5.923168 L
C-CT--0.009400    0   -2.835245   -4.240569    5.171214 L
C-CT-0.018700     0   -2.610381   -2.956322    4.361755 L H-HC 37
C-CT--0.464524    0   -3.707939   -2.778659    3.306438 H
C-CT--0.195159    0   -3.627373   -1.468348    2.533966 H
N-N3--0.748751    0   -2.434192   -1.398132    1.622916 H
H-H-0.274700      0   -1.473521   -2.622970    7.178950 L
H-H1-0.142600     0   -1.941198   -5.520114    6.634046 L
H-HC-0.036200     0   -3.804080   -4.180343    5.670014 L
H-HC-0.036200     0   -2.882869   -5.072404    4.475745 L
H-HC-0.010300     0   -1.639907   -2.993437    3.866553 L
H-HC-0.010300     0   -2.639122   -2.107221    5.036424 L
H-HC-0.173903     0   -4.676813   -2.796946    3.813634 H
H-HC-0.148535     0   -3.688894   -3.634411    2.617476 H
H-HP-0.236066     0   -3.592752   -0.592070    3.178426 H
H-HP-0.172509     0   -4.498874   -1.372704    1.875527 H
H-H-0.437293      0   -2.345659   -0.468827    1.183337 H
H-H-0.366736      0   -2.571504   -2.039160    0.829171 H
H-H-0.419451      0   -1.493062   -1.574217    2.059670 H
N-N--0.415700     0   -0.042222   -5.598398    4.881107 L
C-CT--0.025200   -1    1.272371   -5.799556    4.280688 L
C-C-0.597300      0    1.147053   -6.518543    2.936758 L
O-O--0.567900     0    1.735752   -7.573325    2.707855 L
H-H-0.271900      0   -0.786955   -6.249506    4.650776 L
H-H1-0.069800     0    1.892660   -6.400061    4.946110 L
H-H1-0.069800     0    1.764825   -4.841590    4.109936 L
N-N--0.415700     0    0.327411   -5.937134    2.065020 L
C-CT--0.025200   -1   -0.236686   -6.540933    0.861246 L
C-C-0.597300      0   -1.385078   -5.674472    0.411065 L
O-O--0.567900     0   -2.287120   -5.442008    1.207692 L
H-H-0.271900      0   -0.114943   -5.086611    2.387607 L
H-H1-0.069800     0   -0.608550   -7.539775    1.089364 L
H-H1-0.069800     0    0.528341   -6.597695    0.086366 L
N-N--0.415700     0   -1.331128   -5.200233   -0.833879 L
C-CT--0.001400   -1   -2.161385   -4.113954   -1.359227 L
C-C-0.597300      0   -3.625408   -4.186286   -0.905840 L
O-O--0.567900     0   -4.182560   -3.231075   -0.375065 L
C-CT--0.015200    0   -2.059071   -4.094833   -2.899321 L
C-CA--0.001100    0   -0.670564   -4.319332   -3.474077 L
C-CA--0.190600    0    0.356188   -3.384373   -3.241030 L
C-CA--0.190600    0   -0.409180   -5.470152   -4.245184 L
C-CA--0.234100    0    1.642035   -3.602099   -3.772299 L
C-CA--0.234100    0    0.874322   -5.689365   -4.780521 L
C-C-0.322600      0    1.904091   -4.752688   -4.543205 L
O-OH--0.557900    0    3.138709   -4.963610   -5.072476 L
H-H-0.271900      0   -0.496558   -5.393163   -1.369341 L
H-H1-0.087600     0   -1.737545   -3.191043   -0.972103 L
H-HC-0.029500     0   -2.423071   -3.138858   -3.268753 L
H-HC-0.029500     0   -2.723996   -4.857614   -3.304955 L
H-HA-0.169900     0    0.162994   -2.490226   -2.657039 L
```

H-HA-0.169900	0	-1.202316	-6.176115	-4.443131	L
H-HA-0.165600	0	2.428653	-2.886553	-3.589589	L
H-HA-0.165600	0	1.065747	-6.564192	-5.379739	L
H-HO-0.399200	0	3.144273	-5.746197	-5.620730	L
N-N--0.415700	0	-4.229367	-5.363886	-1.079847	L
C-CT--0.024900	-1	-5.562480	-5.653434	-0.572334	L
C-C-0.597300	0	-5.691657	-5.577253	0.947333	L
O-O--0.567900	0	-5.077012	-6.351563	1.690452	L
C-CT-0.211700	0	-6.030025	-7.045141	-1.001507	L
O-OH--0.654600	-1	-7.283130	-7.352842	-0.404987	L
H-H-0.271900	0	-3.661689	-6.098262	-1.462159	L
H-H1-0.084300	0	-6.249247	-4.929140	-1.010607	L
H-H1-0.035200	0	-5.294764	-7.784745	-0.682354	L
H-H1-0.035200	0	-6.117440	-7.079686	-2.087481	L
H-HO-0.427500	-1	-7.540745	-8.226315	-0.709906	L
N-N--0.415700	0	-6.658827	-4.768013	1.373015	L
C-CT-0.033700	-1	-7.160824	-4.667308	2.732258	L
C-C-0.597300	0	-7.535358	-5.986373	3.412970	L
O-O--0.567900	0	-7.514351	-6.072943	4.639158	L
C-CT--0.182500	0	-8.339351	-3.693283	2.727185	L
H-H-0.271900	0	-7.102657	-4.176514	0.676919	L
H-H1-0.082300	0	-6.371595	-4.226509	3.340283	L
H-HC-0.060300	0	-8.036422	-2.734043	2.303188	L
H-HC-0.060300	0	-8.682968	-3.539317	3.745438	L
H-HC-0.060300	0	-9.158136	-4.109495	2.146330	L
N-N--0.415700	0	-7.892747	-7.005652	2.631332	L
C-CT--0.025200	-1	-8.286923	-8.311263	3.145527	L
C-C-0.597300	0	-7.319194	-9.447637	2.845577	L
O-O--0.567900	0	-7.718287	-10.610216	2.874371	L
H-H-0.271900	0	-7.846058	-6.850887	1.630458	L
H-H1-0.069800	0	-9.249380	-8.574865	2.708293	L
H-H1-0.069800	0	-8.422619	-8.272364	4.226582	L
N-N--0.415700	0	-6.048963	-9.135649	2.594051	L
C-CT--0.024900	-1	-4.970745	-10.122253	2.629621	L
C-C-0.597300	0	-4.284035	-10.078746	3.988899	L
O-O--0.567900	0	-3.975416	-9.001217	4.507208	L
C-CT-0.211700	0	-3.966241	-9.826658	1.510476	L
O-OH--0.654600	-1	-3.091808	-10.927127	1.314784	L
H-H-0.271900	0	-5.801163	-8.149950	2.589369	L
H-H1-0.084300	0	-5.375791	-11.120142	2.461125	L
H-H1-0.035200	0	-3.393941	-8.928810	1.749643	L
H-H1-0.035200	0	-4.514662	-9.653064	0.583786	L
H-HO-0.427500	-1	-2.550990	-10.741201	0.543683	L
N-N--0.415700	0	-4.076060	-11.254942	4.579723	L
C-CT--0.149000	-1	-3.535472	-11.398547	5.920163	L
H-H-0.271900	0	-4.347711	-12.075377	4.061896	L
H-H1-0.097600	0	-3.309779	-12.444398	6.128091	L
H-H1-0.097600	0	-4.258194	-11.032377	6.652679	L
H-H1-0.097600	0	-2.620699	-10.808915	6.017749	L
C-CT--0.366200	-1	-12.142308	5.078439	-0.672988	L
C-C-0.597200	0	-10.714084	5.264755	-0.232679	L
O-O--0.567900	0	-10.401738	5.004063	0.923804	L
H-HC-0.112300	0	-12.534972	6.028121	-1.029430	L
H-HC-0.112300	0	-12.729374	4.733240	0.176379	L

H-HC-0.112300	0	-12.174377	4.331338	-1.463908	L	
N-N--0.415700	0	-9.863617	5.705422	-1.155702	L	
C-CT--0.025200	-1	-8.430390	5.868016	-0.926979	L	
C-C-0.597300	0	-7.610688	5.778445	-2.189636	L	
O-O--0.567900	0	-8.112988	6.110663	-3.270442	L	
H-H-0.271900	0	-10.172832	5.863305	-2.102379	L	
H-H1-0.069800	0	-8.114229	5.066790	-0.268786	L	
H-H1-0.069800	0	-8.230020	6.823183	-0.443742	L	
N-N--0.415700	0	-6.360069	5.343972	-2.019282	L	H-H1 48
C-CT--0.530209	-1	-5.345526	5.286422	-3.069307	H	
C-C-0.568093	0	-4.613106	3.954761	-2.965912	H	
O-O--0.528105	0	-4.593720	3.325255	-1.902309	H	
C-CT-0.298500	0	-4.396546	6.506743	-2.995830	L	H-H1 48
C-CT--0.319200	0	-5.147519	7.808114	-3.288659	L	
C-CT--0.319200	0	-3.689196	6.625983	-1.643252	L	
H-H-0.271900	0	-6.066277	5.030122	-1.096500	L	
H-H1-0.184096	0	-5.865554	5.354613	-4.032310	H	
H-HC--0.029700	0	-3.634916	6.398446	-3.766268	L	
H-HC-0.079100	0	-5.858918	8.016262	-2.491342	L	
H-HC-0.079100	0	-5.680049	7.714769	-4.233813	L	
H-HC-0.079100	0	-4.433764	8.627980	-3.355932	L	
H-HC-0.079100	0	-4.411585	6.750891	-0.838593	L	
H-HC-0.079100	0	-3.008696	7.475042	-1.653783	L	
H-HC-0.079100	0	-3.110477	5.720436	-1.450567	L	
N-N--0.610103	0	-3.961185	3.548813	-4.080066	H	
C-CT--0.314060	-1	-4.233033	3.987138	-5.441930	H	
C-C-0.597300	0	-5.348529	3.161647	-6.075417	L	H-H1 50
O-O--0.567900	0	-5.477007	1.956315	-5.860303	L	
C-CT--0.110200	0	-2.942030	3.894687	-6.293077	L	H-H1 50
C-CT-0.353100	0	-1.949404	5.065128	-6.092976	L	
C-CT--0.412100	0	-1.077971	4.964304	-4.840709	L	
C-CT--0.412100	0	-0.997017	5.144253	-7.291053	L	
H-H-0.398207	0	-3.313277	2.766968	-3.952380	H	
H-H1-0.165130	0	-4.547373	5.031143	-5.450255	H	
H-HC-0.045700	0	-3.243357	3.897619	-7.340576	L	
H-HC-0.045700	0	-2.437353	2.947183	-6.107720	L	
H-HC--0.036100	0	-2.509939	6.000008	-6.057216	L	
H-HC-0.100000	0	-0.463655	4.066654	-4.881866	L	
H-HC-0.100000	0	-1.707661	4.903202	-3.952639	L	
H-HC-0.100000	0	-0.441370	5.842470	-4.749058	L	
H-HC-0.100000	0	-0.421230	4.222253	-7.362510	L	
H-HC-0.100000	0	-0.315292	5.984017	-7.158564	L	
H-HC-0.100000	0	-1.566534	5.292183	-8.206670	L	
N-N--0.415700	0	-6.136010	3.810937	-6.928237	L	
C-CT--0.038900	-1	-7.324008	3.213412	-7.549265	L	
C-C-0.597300	0	-7.078607	1.875249	-8.236027	L	
O-O--0.567900	0	-7.960889	1.020016	-8.280364	L	
C-CT-0.365400	-1	-7.960818	4.135316	-8.613468	L	
O-OH--0.676100	-1	-7.898903	5.493628	-8.168937	L	
C-CT--0.243800	0	-9.439441	3.845788	-8.872084	L	
H-H-0.271900	0	-6.011124	4.808780	-7.013360	L	
H-H1-0.100700	0	-8.064382	3.074048	-6.768479	L	
H-H1-0.004300	0	-7.405457	4.056631	-9.549086	L	
H-HC-0.064200	0	-9.555699	2.841220	-9.278337	L	

```
H-HC-0.064200    0   -9.832940    4.558542   -9.596577 L
H-HC-0.064200    0  -10.002950    3.919243   -7.941139 L
H-HO-0.410200   -1   -8.797028    5.829141   -8.134517 L
N-N--0.415700    0   -5.901350    1.736780   -8.842498 L
C-CT--0.025200  -1   -5.517074    0.578440   -9.635765 L
C-C-0.597300     0   -4.628206   -0.430311   -8.937412 L
O-O--0.567900    0   -4.135930   -1.337656   -9.608886 L
H-H-0.271900     0   -5.213214    2.449395   -8.650603 L
H-H1-0.069800    0   -4.981653    0.923651  -10.518994 L
H-H1-0.069800    0   -6.406422    0.048252   -9.976231 L
N-N--0.415700    0   -4.414795   -0.253721   -7.628601 L H-H1 54
C-CT--0.539097  -1   -3.474858   -1.026041   -6.827190 H
C-C-0.572106     0   -4.208790   -1.479190   -5.563818 H
O-O--0.533001    0   -4.926452   -2.475180   -5.628885 H
C-CT-0.130300    0   -2.135190   -0.265434   -6.629481 L H-H1 54
C-CT--0.043000   0   -1.528215    0.232425   -7.963306 L
C-CT--0.320400   0   -1.165090   -1.229080   -5.924049 L
C-CT--0.066000   0   -0.213679    1.010137   -7.816131 L
H-H-0.271900     0   -4.877092    0.520401   -7.159591 L
H-H1-0.180646    0   -3.253253   -1.945679   -7.378811 H
H-HC-0.018700    0   -2.307025    0.599669   -5.989920 L
H-HC-0.023600    0   -2.234993    0.909341   -8.441645 L
H-HC-0.023600    0   -1.366447   -0.617322   -8.627629 L
H-HC-0.088200    0   -0.968951   -2.095754   -6.553586 L
H-HC-0.088200    0   -1.583869   -1.564017   -4.976737 L
H-HC-0.088200    0   -0.229166   -0.727951   -5.691424 L
H-HC-0.018600    0   -0.341876    1.814890   -7.093019 L
H-HC-0.018600    0    0.059589    1.439848   -8.779826 L
H-HC-0.018600    0    0.589887    0.349452   -7.492116 L
N-N--0.611145    0   -4.076405   -0.715591   -4.445250 H
C-CT--0.109296  -1   -4.640165   -1.118091   -3.165138 H
C-C-0.597300     0   -6.167934   -1.141589   -3.175129 L H-H1 56
O-O--0.567900    0   -6.799020   -2.077768   -2.669367 L
C-CT--0.388038   0   -4.107727   -0.309898   -2.002758 H
H-H-0.369964     0   -3.420831    0.056495   -4.417047 H
H-H1-0.178541    0   -4.385884   -2.169905   -2.988172 H
H-H1-0.157964    0   -4.719937   -0.571612   -1.133128 H
H-H1-0.179833    0   -4.180515    0.769637   -2.157366 H
H-HS-0.319500    0   -1.976627    0.458942   -1.346472 H
N-N--0.415700    0   -6.758678   -0.144481   -3.820765 L
C-CT--0.087500  -1   -8.196399   -0.079455   -4.064676 L
C-C-0.597300     0   -8.683852   -1.230164   -4.931983 L
O-O--0.567900    0   -9.630047   -1.923845   -4.559072 L
C-CT-0.298500    0   -8.591490    1.284130   -4.650809 L
C-CT--0.319200   0  -10.108215    1.415564   -4.846157 L
C-CT--0.319200   0   -8.142654    2.457937   -3.765953 L
H-H-0.271900     0   -6.122617    0.544654   -4.215437 L
H-H1-0.096900    0   -8.699357   -0.178139   -3.105201 L
H-HC--0.029700   0   -8.113431    1.369850   -5.618183 L
H-HC-0.079100    0  -10.618072    1.268425   -3.893789 L
H-HC-0.079100    0  -10.460116    0.674358   -5.563410 L
H-HC-0.079100    0  -10.343951    2.406120   -5.234117 L
H-HC-0.079100    0   -8.596200    2.390237   -2.778978 L
H-HC-0.079100    0   -8.418810    3.406503   -4.225563 L
```

H-HC-0.079100	0	-7.058248	2.456329	-3.646897	L
N-N--0.415700	0	-8.046251	-1.445296	-6.081255	L
C-CT--0.025200	-1	-8.406490	-2.504039	-7.021142	L
C-C-0.597300	0	-8.316710	-3.904506	-6.428338	L
O-O--0.567900	0	-9.213912	-4.721650	-6.624741	L
H-H-0.271900	0	-7.247310	-0.857080	-6.282139	L
H-H1-0.069800	0	-7.739698	-2.454657	-7.881717	L
H-H1-0.069800	0	-9.427475	-2.342282	-7.366318	L
N-N--0.415700	0	-7.235401	-4.177732	-5.700430	L
C-CT--0.025200	-1	-7.013686	-5.441830	-5.009193	L
C-C-0.597300	0	-7.946853	-5.700506	-3.837575	L
O-O--0.567900	0	-8.283245	-6.851617	-3.564919	L
H-H-0.271900	0	-6.520740	-3.454644	-5.631364	L
H-H1-0.069800	0	-5.990761	-5.459091	-4.634712	L
H-H1-0.069800	0	-7.131041	-6.259695	-5.720236	L
N-N--0.415700	0	-8.333438	-4.641934	-3.129527	L
C-CT--0.038900	-1	-9.384393	-4.695853	-2.111040	L
C-C-0.597300	0	-10.754924	-4.989389	-2.712240	L
O-O--0.567900	0	-11.498434	-5.823518	-2.195186	L
C-CT-0.365400	0	-9.420294	-3.390200	-1.302144	L
O-OH--0.676100	0	-8.182391	-3.219259	-0.650306	L
C-CT--0.243800	0	-10.504754	-3.396258	-0.225239	L
H-H-0.271900	0	-7.941155	-3.739247	-3.375538	L
H-H1-0.100700	0	-9.157351	-5.508194	-1.420780	L
H-H1-0.004300	0	-9.588673	-2.542482	-1.966492	L
H-HC-0.064200	0	-11.485840	-3.291632	-0.687182	L
H-HC-0.064200	0	-10.348524	-2.562593	0.458932	L
H-HC-0.064200	0	-10.470471	-4.333172	0.328973	L
H-HO-0.410200	0	-7.594680	-2.764436	-1.287756	L
N-N--0.415700	0	-11.104475	-4.284677	-3.789679	L
C-CT--0.149000	-1	-12.336345	-4.479187	-4.526289	L
H-H-0.271900	0	-10.438075	-3.601499	-4.139180	L
H-H1-0.097600	0	-12.412252	-3.728204	-5.313615	L
H-H1-0.097600	0	-13.188916	-4.392959	-3.851309	L
H-H1-0.097600	0	-12.333195	-5.472887	-4.977323	L
N-N2--0.511415	0	2.084375	-0.011345	-1.760652	H
C-CA-0.057801	0	0.945468	0.693581	-1.694224	H
C-CA-0.063496	0	0.353734	1.294168	-2.804833	H
C-CA--0.109122	0	1.005270	1.182853	-4.032649	H
C-CA--0.164490	0	2.204427	0.485643	-4.101856	H
C-CA-0.098392	0	2.704282	-0.107943	-2.948300	H
C-C-0.541263	0	-1.021779	1.925071	-2.724667	H
O-O--0.574884	0	-1.648787	1.958970	-3.807310	H
H-H4-0.220889	0	0.492662	0.773698	-0.712214	H
H-HA-0.160434	0	0.547266	1.631733	-4.905957	H
H-HA-0.142191	0	2.747203	0.394385	-5.034551	H
H-H4-0.174502	0	3.617322	-0.692888	-2.957961	H
H-H-0.353801	0	-1.463127	1.829437	-0.020211	H
Zn-ZN-0.974537	0	2.832594	-1.069740	-0.035960	H
O-OW--0.834000	0	-2.136430	-7.244736	3.510040	L
H-HW-0.417000	0	-2.569496	-6.661524	2.866822	L
H-HW-0.417000	0	-2.864599	-7.783638	3.860055	L
O-OW--0.783903	0	1.310937	-2.552233	-0.149018	H
H-HW-0.401253	0	0.902190	-2.438993	0.743136	H

H-HW-0.424111	0	1.916466	-3.337527	-0.177706	H
O-OW--0.834000	0	-3.715819	-8.436787	7.255232	L
H-HW-0.417000	0	-3.763697	-7.491653	7.420328	L
H-HW-0.417000	0	-3.831832	-8.513526	6.295981	L
O-OW--0.834000	0	7.139622	-0.015964	1.790140	L
H-HW-0.417000	0	6.838958	0.411143	2.613069	L
H-HW-0.417000	0	6.344063	0.055031	1.238556	L
O-OW--0.834000	0	7.045671	-4.935450	4.766743	L
H-HW-0.417000	0	6.097161	-4.998784	5.000480	L
H-HW-0.417000	0	7.246209	-5.806518	4.422279	L
O-OW--0.834000	0	8.643767	-0.913549	-0.234469	L
H-HW-0.417000	0	8.151645	-0.542532	0.526329	L
H-HW-0.417000	0	8.843351	-0.125762	-0.760404	L
O-OW--0.834000	0	12.639631	-1.080430	-1.985004	L
H-HW-0.417000	0	11.779163	-0.832941	-2.365078	L
H-HW-0.417000	0	12.645246	-2.043758	-2.094303	L
O-OW--0.834000	0	8.307922	-3.285496	1.004191	L
H-HW-0.417000	0	8.519920	-2.514516	0.437399	L
H-HW-0.417000	0	7.570664	-2.953157	1.526393	L
O-OW--0.834000	0	4.354652	-4.895514	5.170240	L
H-HW-0.417000	0	4.084052	-4.513171	6.027947	L
H-HW-0.417000	0	4.052064	-4.227253	4.544431	L
S-SH--0.326142	0	-2.361108	-0.785926	-1.678212	H
O-OW--0.834000	0	-1.449178	2.275034	-1.577830	H
